# Spatially deterministic nucleation of 2D semiconductors by etching flux

**DOI:** 10.1038/s41586-026-11095-1

**Published:** 2026-10-07

**Authors:** Jeongwon Park, Jiyun Kim, Sumin Kang, Jieun Oh, Youngmin Sunwoo, Saeyoung Oh, Seunghye Shin, Sera Yang, Seonghwan Jo, Jaehyun Lee, Donghyeop Lee, Minsu Kim, Changhun Eom, Seohyun Jeong, Chan Lim, Gunho Moon, Mingu Kang, Min-gyu Kim, Junhyeok Kim, Chaejeong Yun, Gichang Noh, Donggeon Park, Dongyoung Kim, Seongdae Kwon, Chengyun Hong, Hyeongjin Lim, Minseok Choi, Yongjoon Lee, Joonghoon Choi, Junhyuk Tak, Guanning Shao, Seung Jae Kwak, Tae Soo Kim, Jeehwan Kim, YongJoo Kim, Young Joon Hong, Sung-Yool Choi, Heejun Yang, Jonghwan Kim, Byungjo Kim, Woo-Hee Kim, Chang-Soo Lee, Kibum Kang

**Affiliations:** 1 Department of Materials Science and Engineering, Korea Advanced Institute of Science and Technology (KAIST), Daejeon, Republic of Korea; 2 TDS Innovation Inc., Daejeon, Republic of Korea; 3 Department of Materials Science and Chemical Engineering, BK21 FOUR ERICA-ACE Center, Hanyang University, Ansan, Republic of Korea; 4 Graduate School of Semiconductor Materials and Devices Engineering, Ulsan National Institute of Science and Technology (UNIST), Ulsan, Republic of Korea; 5 Graduate School of Semiconductor Technology, Korea Advanced Institute of Science and Technology (KAIST), Daejeon, Republic of Korea; 6 Center for Van der Waals Quantum Solids, Institute for Basic Science (IBS), Pohang, Republic of Korea; 7 Department of Materials Science and Engineering, Pohang University of Science and Technology, Pohang, Republic of Korea; 8 Department of Physics, Korea Advanced Institute of Science and Technology (KAIST), Daejeon, Republic of Korea; 9 KAIST InnoCORE PRISM-AI Center, Korea Advanced Institute of Science and Technology (KAIST), Daejeon, Republic of Korea; 10 School of Electrical Engineering, Korea Advanced Institute of Science and Technology (KAIST), Daejeon, Republic of Korea; 11 Department of Nano Science and Technology, Sungkyunkwan University (SKKU), Suwon, Republic of Korea; 12 Department of Materials Science and Engineering, Korea University, Seoul, Republic of Korea; 13 Department of Nanotechnology and Advanced Materials Engineering, Sejong University, Seoul, Republic of Korea; 14 School of Chemical and Biological Engineering and Institute of Chemical Processes, Seoul National University (SNU), Seoul, Republic of Korea; 15 Department of Mechanical Engineering, Massachusetts Institute of Technology, Cambridge, MA USA; 16 Department of Physics, Pohang University of Science and Technology, Pohang, Republic of Korea

**Keywords:** Two-dimensional materials, Two-dimensional materials, Electronic devices, Synthesis and processing, Synthesis and processing

## Abstract

Nucleation control is fundamental to semiconductor deposition, governing when, where and how crystalline materials form. Conventional strategies can regulate nucleation density and, through area-selective growth, confine deposition to designated regions^1–13^. However, they generally do not determine where an individual nucleus forms within the growth regions, leaving even single-nucleation events spatially stochastic and limiting deterministic construction of crystalline materials. Here we report etching-flux-mediated single-centred nucleation of two-dimensional (2D) semiconductors to deterministically localize a single nucleation event. The etching flux released from the barrier suppresses nuclei near the pattern boundary, leaving a single surviving nucleus at the pattern centre, and systematic experiments elucidate the mechanism and establish nucleation design rules for zero, single and multiple nucleation regimes. Etching-flux-mediated single-centred nucleation enables single-crystal molybdenum disulfide growth at the 10-μm scale, field-effect mobilities of up to 117 cm^2^ V^−1^ s^−1^ and large-area uniformity with process compatibility. This in-plane chemical-flux strategy realizes spatially programmed growth, demonstrating nucleation–growth decoupling for line-shaped single crystals, multiple transistors integrated within a large common crystal, and aligned 2D lateral heterostructures for self-aligned contacts. These capabilities open a path towards advanced 2D electronic integration and expand semiconductor deposition from controlling where materials grow to controlling where and how crystals can begin to form.

## Abstract (web summary)

Etching-flux-mediated single-centred nucleation of two-dimensional semiconductors is used to deterministically localize a single nucleation event, expanding semiconductor deposition from controlling where materials grow to controlling where and how crystals can begin to form.

## Main

Attaining highly localized and precise control of nucleation has emerged as a critical requirement in modern semiconductor processing, as such precision fundamentally governs uniformity and device performance. Area-selective growth (ASG) is a promising approach that deposits target materials on the desired regions, driven by a reaction-rate difference between growth and barrier regions. Over the past few decades, ASG has evolved through various approaches, including selective epitaxial growth of silicon–germanium stressors and III–V semiconductors^1–3^, selective metallization such as tungsten gap filling and cobalt capping^4,5^, and area-selective atomic layer deposition (AS-ALD) for oxide spacers and nitride barriers^6,7^. Recently, the ASG technique has been extended to two-dimensional (2D) transition-metal dichalcogenides (TMDs), which is a promising next-generation semiconductor, via selective chemical vapour deposition (CVD)^8–13^.

In 2D TMD ASG, selectivity can be realized by using heterogeneous surface patterning with a barrier region through mechanisms similar to those in three-dimensional (3D) bulk ASG processes. Recently, single-crystal 2D TMDs have been demonstrated through confined growth; however, the achievable crystal size remains limited^8,9^. As the entire growth region generally serves as a nucleation-active region (NAR) in conventional ASG, nucleation events occur stochastically at random sites within the growth region. Such stochastic nucleation behaviour constrains the attainable single-crystal domain size and hinders reliable spatial control, indicating the need for an advanced ASG strategy that enables large single-crystal growth and aligned heterostructure assembly.

To address these limitations, we developed an etching-flux-mediated single-centred nucleation (EF-SCN) strategy that introduces a paradigm for nucleation control in ASG systems. Unlike conventional 2D TMD ASG, which relies solely on out-of-plane selectivity through heterogeneous surface systems with different surface binding energies, EF-SCN achieves in-plane spatial control of nucleation by creating a non-uniform nucleation probability within the growth region. Oxygen released from an oxide barrier produces a lateral etching flux that suppresses nucleation, defining the NAR at the geometric centre of each pattern. This flux-driven mechanism enables deterministic nucleation and establishes a physical framework in which the in-plane chemical flux regulates spatial nucleation probability.

From a practical standpoint, EF-SCN also provides substantial advantages and novel device configurations. The deterministic nucleation at a centre point enables effective nucleation suppression and single-crystal size scaling, producing single-crystal molybdenum disulfide (MoS_2_) at the 10-μm scale. For process compatibility, EF-SCN achieves a single-crystal yield exceeding 99% and uniform selectivity over a large area, and can be applied to low-temperature growth at 430 °C. The EF-SCN-grown single-crystal MoS_2_ exhibits a high field-effect mobility of 117 cm^2^ V^−1^ s^−1^, and a large single crystal realizes the integration of multiple field-effect transistors (FETs) within a single channel with a mobility relative standard deviation (RSD) of 7.8%. Moreover, precise positional and orientational alignment of 2D lateral heterostructures enables a distinctive demonstration of self-aligned ALD contact FETs. Collectively, EF-SCN establishes a selective-growth framework that achieves deterministic nucleation and spatially programmed growth, providing a pathway towards functional single-crystal architectures and advanced 2D electronic integration.

## Etching-flux-mediated single-centred nucleation

**Fig. 1:**
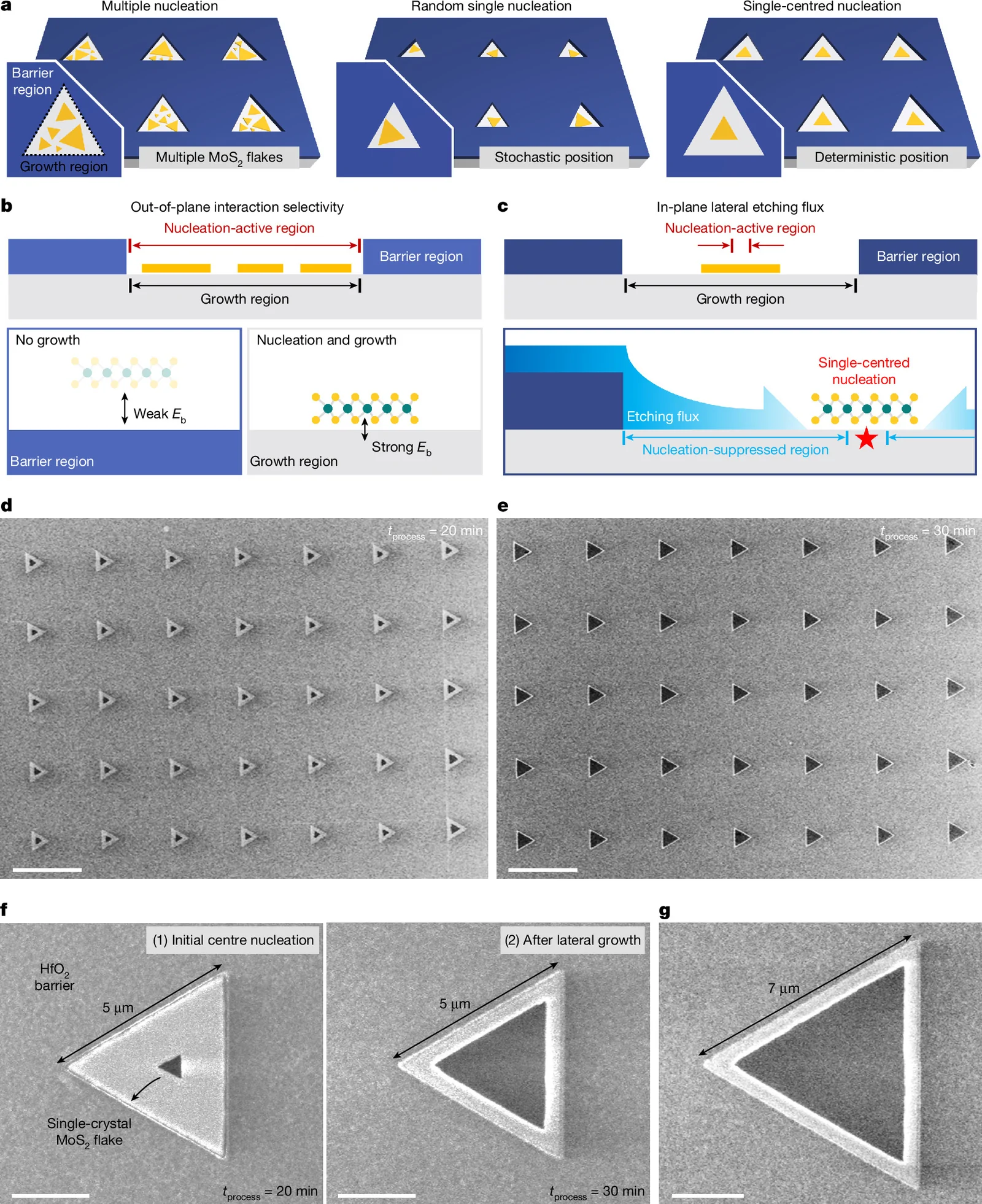
Two-dimensional monolayer MoS_2_ EF-SCN. **a**, Schematic of 2D MoS_2_ selective growth based on a nucleation barrier: multiple nucleation (left), single nucleation with stochastic position (middle) and single-centred nucleation (right). **b**,**c**, Schematics of MoS_2_ selective-growth mechanisms based on a binding energy (*E*_b_) difference (**b**) and a lateral etching-flux barrier (**c**). **d**,**e**, SEM images of the 2D MoS_2_ EF-SCN at the initial stage (**d**) and the fully grown stage (**e**) at the array level. The black regions are single-crystal MoS_2_ flakes and the darker grey region is the HfO_2_ barrier. **f**,**g**, SEM images of 2D MoS_2_ single-centred nucleation and lateral growth (**f**) and large-size single-crystal growth (**g**). Scale bars, 15 μm (**d**,**e**); 2 μm (**f**,**g**).

Figure 1a illustrates conventional 2D TMD ASG using a nucleation barrier. In this scheme, nucleation is intentionally suppressed in the barrier region, leading to selective deposition within the growth region (Fig. 1a, left), especially at the corners, edges or terrace sites of the pattern^8–11^ (Supplementary Fig. 1a). Inherently, multiple nucleation tends to arise because the NAR (red regions in Supplementary Fig. 1a) does not form as a single unified site within the growth region, regardless of nucleation site type. Reducing the growth-region size can mitigate this issue, enabling single-crystal growth of 2D TMD even through the conventional ASG approach (Fig. 1a, middle). However, the nucleation still occurs stochastically within the growth region (red stars in Supplementary Fig. 1a), requiring advanced nucleation strategies for deterministic positioning. Figure 1 shows single-centred nucleation, where a single-crystal TMD is exclusively formed at the centre of the growth region (Fig. 1a, right).

Figure 1b,c shows schematics comparing conventional 2D TMD ASG and the EF-SCN mechanism. In conventional ASG, TMD exhibits weaker binding energy on the barrier region and stronger binding energy on the growth region; therefore, out-of-plane interaction selectivity between the two regions is the main principle^8,14^ (Fig. 1b). Consequently, the entire growth region serves as an NAR with a uniform nucleation probability, leading to nucleation at stochastic positions within the growth region (Supplementary Fig. 1b). In contrast, EF-SCN uses a barrier that releases an etching flux capable of inducing 2D TMD desorption (Fig. 1c). The area close to the etching-flux barrier becomes a nucleation-suppressed region (NSR), and only the central point remains a valid NAR. This in-plane lateral etching flux induces the maximum nucleation probability at the centre of the growth region, resulting in the deterministic single-centred nucleation (Supplementary Fig. 1c). As 2D materials are bound to the substrate via weak van der Waals interaction, their nucleation is expected to be sensitively controlled by the in-plane etching flux.

**Extended Data Fig. 1:**
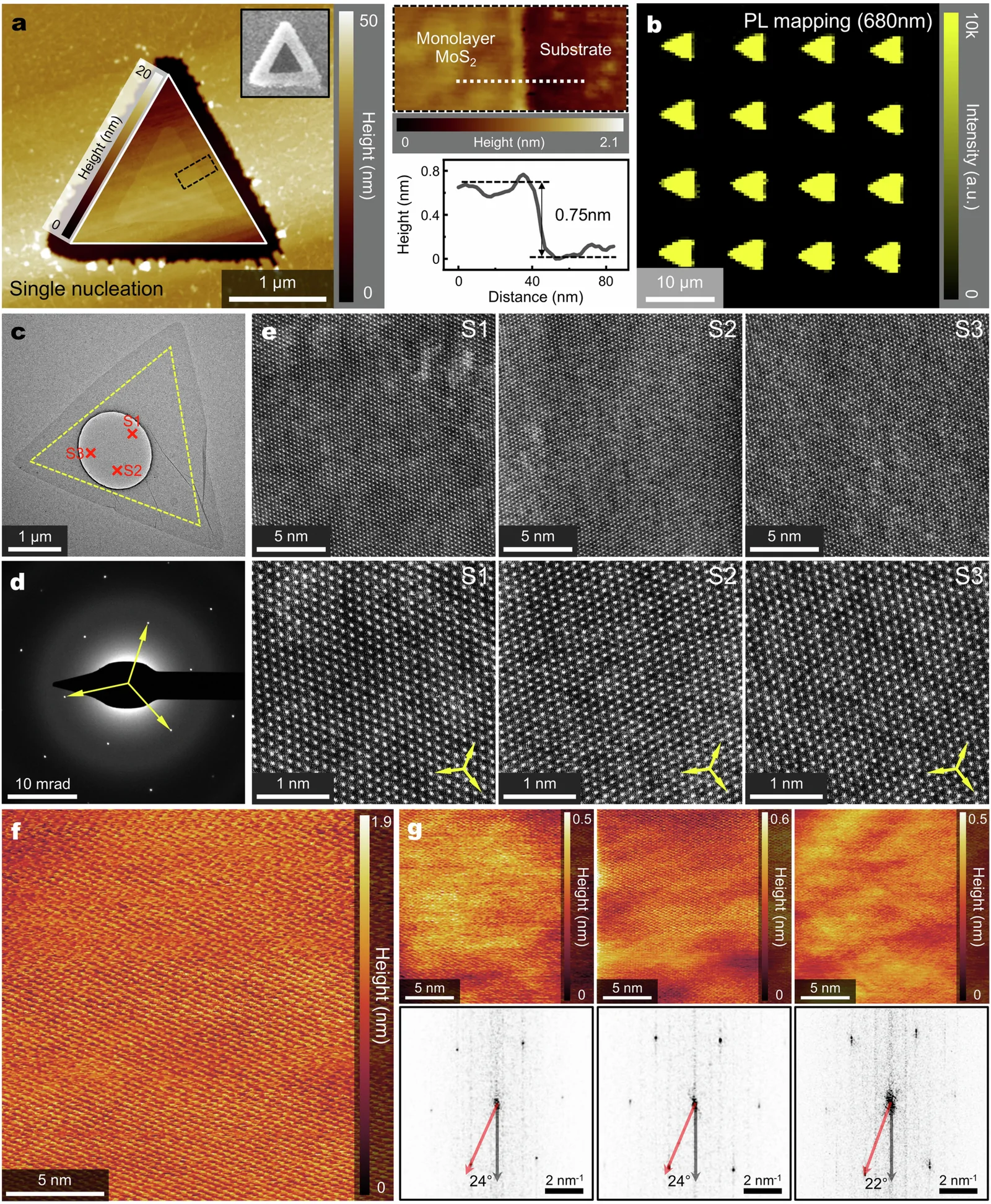
Characterizations of single-crystal MoS_2_ flake by EF-SCN. **a**, AFM image of MoS_2_ single-centred nucleation with the corresponding SEM inset image and height profile. **b**, Photoluminescence (PL) mapping image of a MoS_2_ EF-SCN array. **c**, TEM image of a single-crystal MoS_2_ flake fully suspended over a TEM grid hole. **d**, Diffraction pattern obtained from the flake in **c**. **e**, HAADF-STEM images acquired at S1, S2, and S3 positions marked in **c**, showing consistent atomic lattice orientation across the flake. **f**, Representative atomic-resolution AFM image of single-crystal MoS_2_ flake by EF-SCN. **g**, AFM images and corresponding FFT patterns of single-crystal MoS_2_. Source data

Figure 1d,e shows representative scanning electron microscope (SEM) images of EF-SCN for 2D monolayer MoS_2_. The MoS_2_ (black area) was grown by metal–organic chemical vapour deposition (MOCVD), and the patterned substrate consisted of a miscut sapphire growth region (light-grey triangular area) and a hafnium oxide (HfO_2_) etching-flux barrier region (dark-grey area). Uniform MoS_2_-centred nucleation across the array (Fig. 1d and Supplementary Fig. 2a) and fully filled single-crystal growth (Fig. 1e) were observed. As shown in Fig. 1f, single-centred nucleation emerges at the initial stage and is followed by lateral growth to form a single-crystal MoS_2_ flake. Figure 1g shows a large single-crystal MoS_2_ flake (approximately 7 μm), demonstrating the effective size scaling through the non-uniform nucleation probability distribution in the EF-SCN system. In addition, atomic force microscopy (AFM) confirms a monolayer thickness of 0.75 nm (Extended Data Fig. 1a). Supplementary Fig. 2b shows that EF-SCN is also applicable to square barrier patterns, and the photoluminescence spectra and mapping image describe uniform selective MoS_2_ growth (Supplementary Fig. 2c and Extended Data Fig. 1b).

**Fig. 2:**
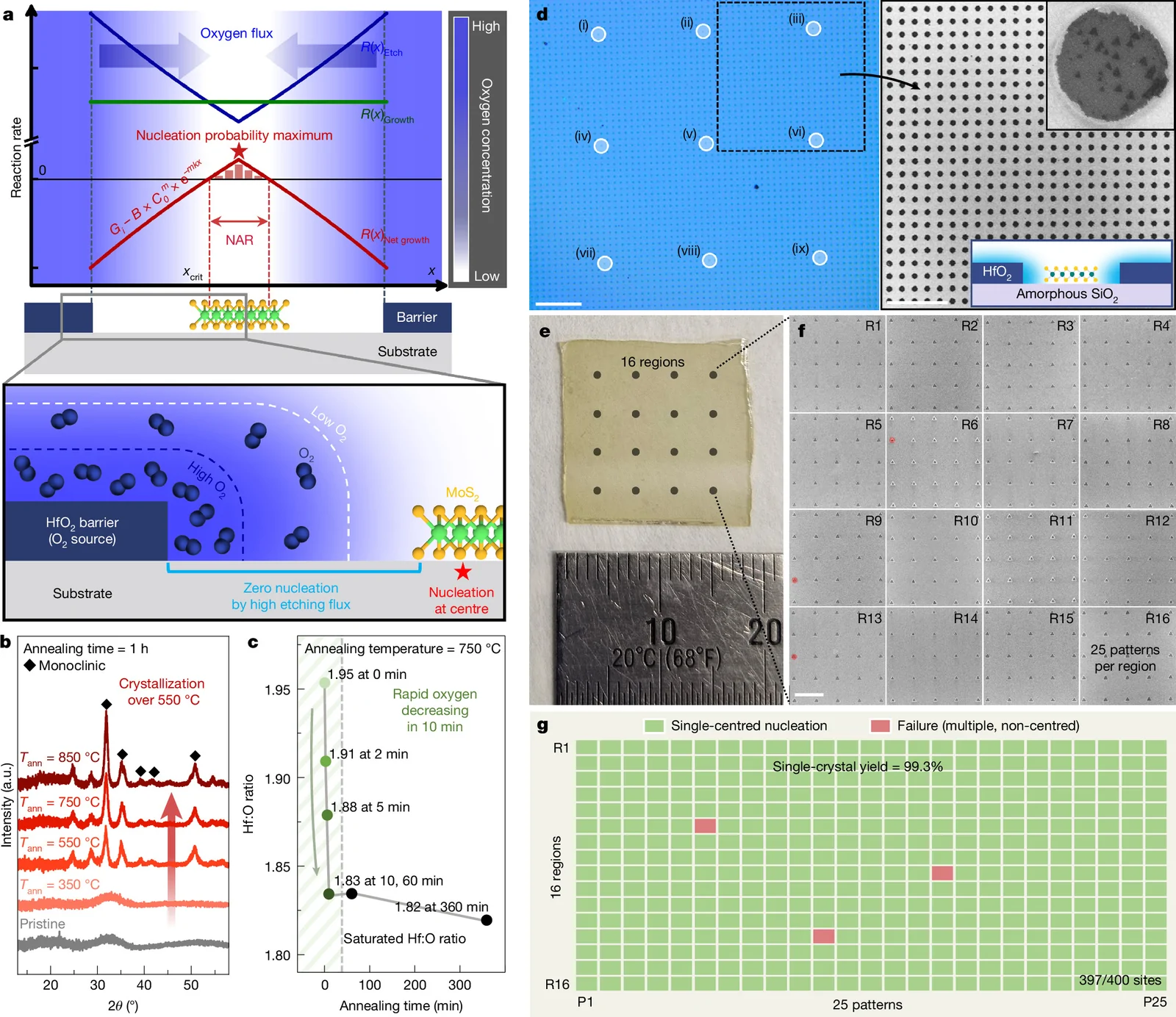
Etching-flux barrier mechanism and process compatibility of the EF-SCN system. **a**, Schematic (bottom) and reaction-rate graph (top) of the 2D MoS_2_ single-centred nucleation. The blue gradient region represents the oxygen concentration, and the blue, green and red lines indicate the MoS_2_ etching, growth and net growth rate for nucleation, respectively. The schematic below shows oxygen release from the etching-flux barrier. **b**, Grazing incidence X-ray diffraction spectra of HfO_2_ at different annealing temperatures (*T*_ann_). **c**, Atomic ratios of HfO_2_ measured by XPS after different annealing times. **d**, Optical microscope image (left) and SEM images (right) showing single-crystal MoS_2_ growth by EF-SCN on an amorphous SiO_2_ substrate. (i)–(ix) indicate nine arbitrarily selected regions in the image. **e**–**g**, Photograph (**e**), SEM images (**f**) and single-crystal mapping (**g**) of large-scale MoS_2_ EF-SCN consisting of 16 regions (R1–R16), each containing 25 patterns (P1–P25) (a total of 400 sites). Scale bars, 50 μm (**d**(left)); 30 μm (**d**(right)); 20 μm (**f**). Source data

**Extended Data Fig. 2:**
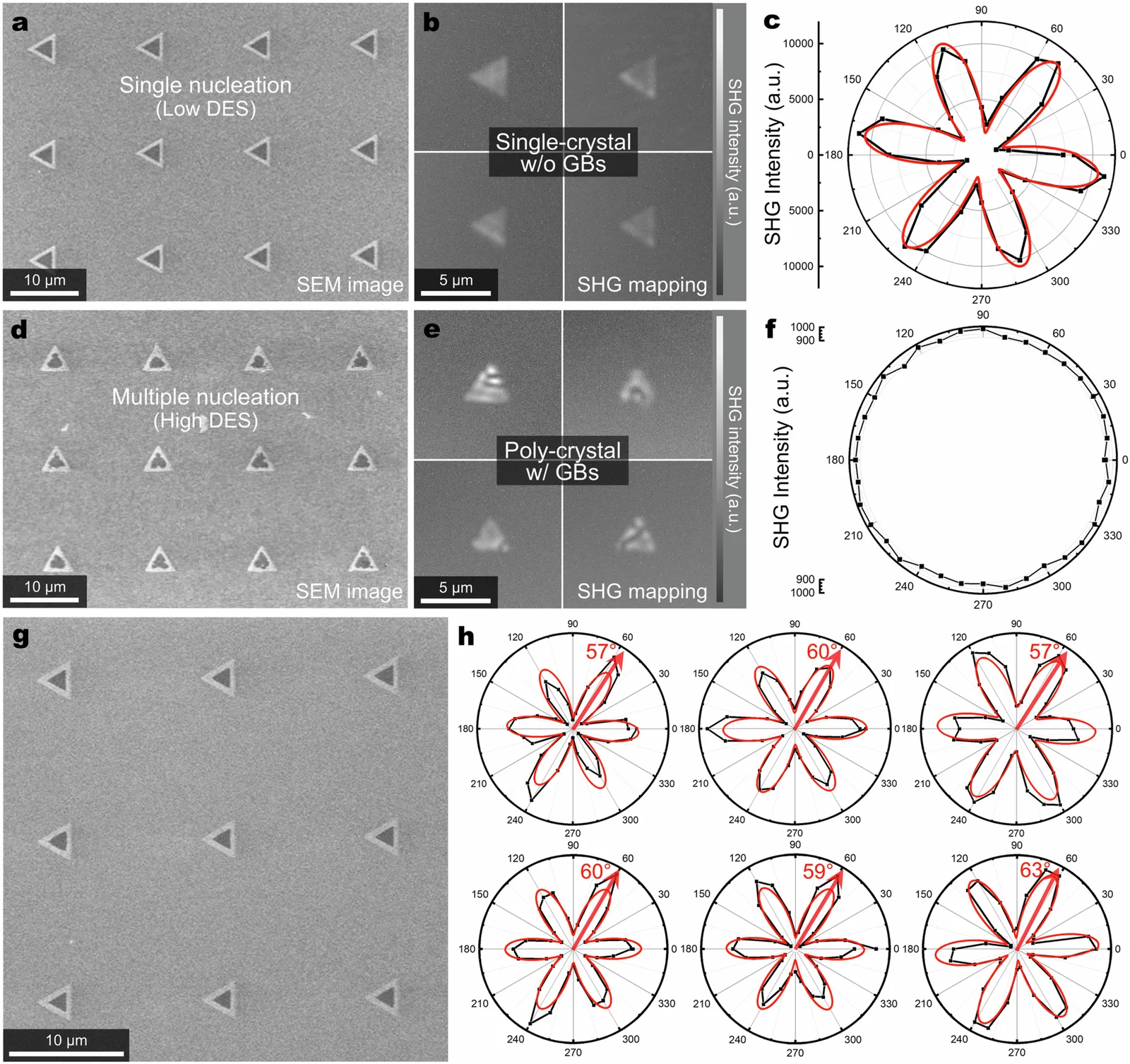
SHG mapping and angle-resolved SHG patterns of single-crystal MoS_2_ flake by EF-SCN. **a**–**c**, SEM image (**a**), SHG mapping image (**b**), and angle-resolved SHG patterns (**c**) of single-crystal MoS_2_ flakes under low-DES condition. **d**–**f**, SEM image (**d**), SHG mapping image (**e**), and angle-resolved SHG patterns (**f**) of polycrystalline MoS_2_ domains under high-DES condition. **g**,**h**, SEM image (**g**) and corresponding angle-resolved SHG patterns (**h**) of single-crystal MoS_2_.

Confirming that the MoS_2_ flakes grown by EF-SCN are single-crystalline is essential; therefore, rigorous structural and optical characterizations were performed. SEM images of a typical 2D TMD morphology (Supplementary Figs. 2 and 3), transmission electron microscopy (TEM) results such as a diffraction pattern and high-angle annular dark-field scanning transmission electron microscopy (HAADF-STEM) images (Extended Data Fig. 1c–e), atomic-resolution AFM (Extended Data Fig. 1f,g), and second-harmonic-generation intensity mapping and angle-resolved patterns (Extended Data Fig. 2) consistently verified that the EF-SCN-grown MoS_2_ flakes are single-crystalline rather than polycrystalline. Detailed analyses are provided in Supplementary Note 1. Figure 2 further elucidates the mechanism of the etching-flux barrier, a key component of the EF-SCN system, as well as its processing compatibility.

## EF-SCN mechanism and process compatibility

Figure 2 shows the release of oxygen from the HfO_2_ etching-flux barrier and its impact on MoS_2_ nucleation. Supplementary Table 1 summarizes the transition temperatures of four representative oxides frequently used as substrates in 2D TMD CVD processes. Sapphire, amorphous Al_2_O_3_ (a-Al_2_O_3_) and amorphous SiO_2_ (a-SiO_2_) undergo thermal decomposition or crystallization at approximately 1,100 °C, 900 °C and 800 °C, respectively^15–23^. In contrast, amorphous HfO_2_ (a-HfO_2_) undergoes thermal decomposition and oxygen reduction at approximately 500 °C, with fast crystallization and oxygen self-diffusion^24–27^. X-ray diffraction of HfO_2_ annealed for 1 hour under vacuum revealed monoclinic phase peaks above 550 °C, consistent with its crystallization (Fig. 2b). At the typical EF-SCN temperature of 750 °C, X-ray photoelectron spectroscopy (XPS) showed that the oxygen content decreased rapidly from HfO_1.95_ to HfO_1.83_ within 10 minutes and remained saturated (Fig. 2c). These results suggest that the HfO_2_ barrier can effectively release oxygen during the MoS_2_ MOCVD process owing to its low thermal stability. As oxygen is known to induce an etching effect on MoS_2_ growth, we examined whether oxygen released from the HfO_2_ barrier is sufficient to suppress MoS_2_ nucleation^28–31^.

**Extended Data Fig. 3:**
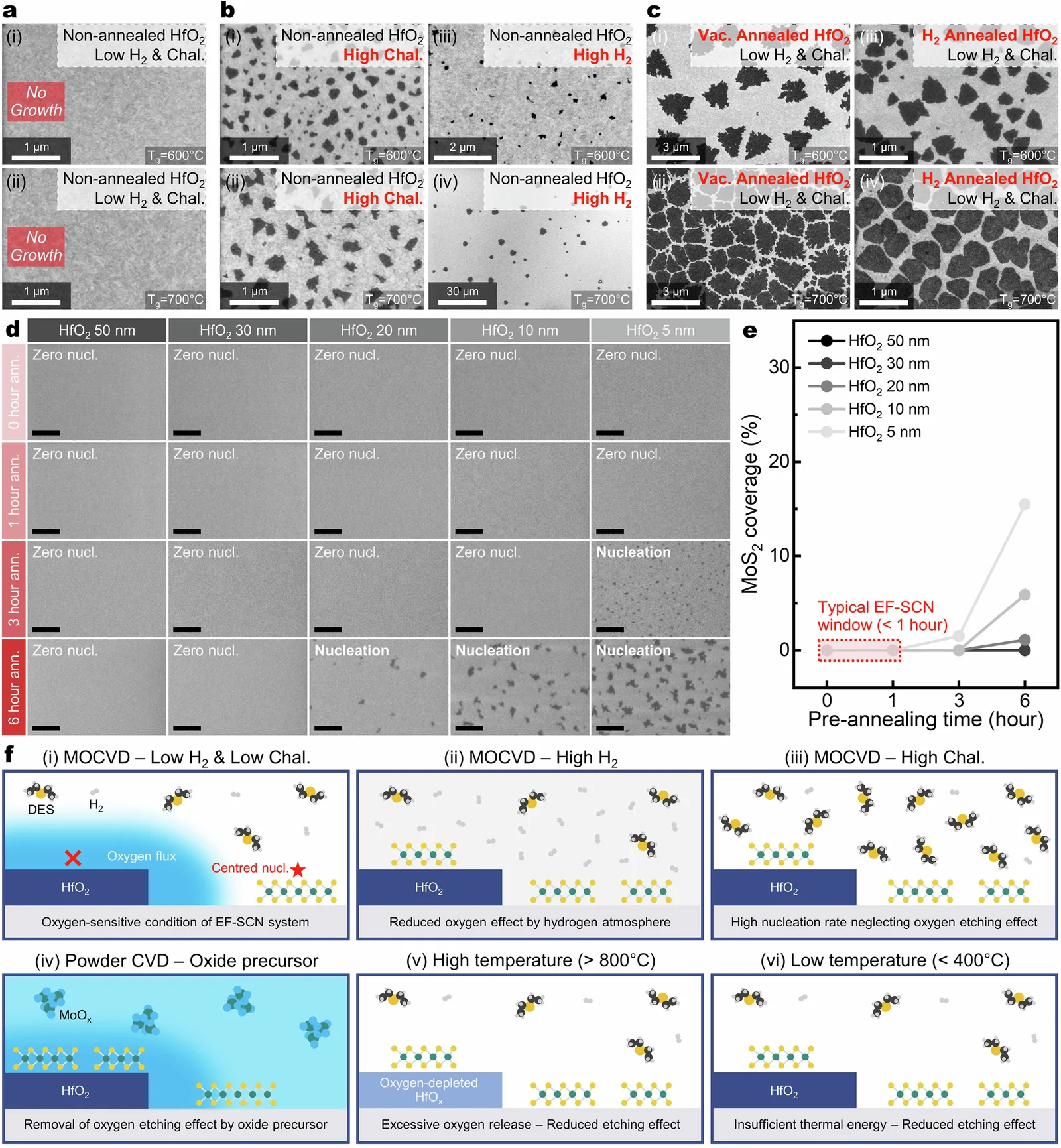
Valid conditions for HfO_2_ to act as an etching-flux barrier. **a**–**c**, SEM images of MoS_2_ grown on HfO_2_ under low-H_2_ and low-chalcogen conditions (**a**), under high-H_2_ or high-chalcogen conditions (**b**), and on annealed HfO_2_ under low-H_2_ and low-chalcogen conditions (**c**). **d**, SEM images of MoS_2_ nucleation as a function of HfO_2_ thickness and in-situ pre-annealing time. Scale bars, 500 nm. **e**, Corresponding graph of MoS_2_ coverage extracted from the SEM images in **d**. **f**, Schematic of MoS_2_ growth on an HfO_2_ surface with different conditions. Source data

Supplementary Fig. 4 experimentally demonstrates that among the compared oxides, only HfO_2_ suppresses MoS_2_ nucleation under identical MOCVD conditions. In addition, Supplementary Fig. 5 shows that MoS_2_ can be completely etched away by oxygen released from the HfO_2_ barrier. Importantly, compared with previous reports of MoS_2_ growth on HfO_2_ surfaces, the EF-SCN MOCVD environment is more sensitive to oxygen-induced etching, which can be attributed to the precursor type^8,32^. More detailed discussions of the MoS_2_ nucleation behaviour on the various oxides and oxygen-sensitive growth conditions for valid EF-SCN are provided in Supplementary Note 2, together with Extended Data Fig. 3 and Supplementary Table 2.

We confirmed that HfO_2_ acts as an etching-flux barrier by releasing oxygen, thereby enabling deterministic control over the nucleation site of 2D MoS_2_. Supplementary Fig. 6 presents a model describing the oxygen flux, oxygen concentration and MoS_2_ nucleation rate within the growth region, with detailed derivations and assumptions provided in Supplementary Note 3 and Supplementary Table 3. According to Fick’s first law, the oxygen concentration within the growth region at a given time can be expressed as:

1 $${C}_{{{\rm{O}}}_{2}}(x)=\frac{A}{{Dk}}\exp (-{kx})$$

where *A* is the oxygen flux constant along the *x* direction, *D* is the oxygen diffusivity and *k* is the oxygen removal constant along the *z* direction. Considering the temporal decrease in oxygen concentration released from HfO_2_, the net growth reaction rate for MoS_2_ nucleation can be described as:

2 $${R(x,t)}_{\text{Net growth}}={G}_{i}-B\times {C}_{0}^{m}\times \exp (-{mkx})\times {f(t)}^{m}$$

in which *B* is the rate constant of MoS_2_ etching reactions, *C*_0_ is the oxygen concentration at *x* = 0, *m* is the reaction order with respect to oxygen, *G*_*i*_ is the constant growth reaction rate and *f*(*t*) is the time-dependent oxygen-decay term.

Figure 2a and Supplementary Fig. 6c illustrate the overall EF-SCN mechanism by incorporating the nucleation-rate model with the oxygen-release phenomenon. As the distance from the etching-flux barrier increases, the net nucleation rate (red line) increases, producing a non-uniform nucleation probability distribution with a maximum at the centre of the growth region. The central region where *R*(*x*)_Net growth_ > 0 forms an NAR, whereas the region near the barrier edge where *R*(*x*)_Net growth_ = 0 forms an NSR. The distance from the barrier edge at which *R*(*x*)_Net growth_ = 0 is defined as the critical length (*x*_crit_). Consistent with this model, the SEM image in Supplementary Fig. 6d shows that the region near the HfO_2_ barrier becomes the NSR without MoS_2_ nucleation, and MoS_2_ flakes are observed in the central NAR. This result demonstrates that the etching flux released from HfO_2_ controls the nucleation position even within the growth region.

The EF-SCN system, established through the above nucleation model, offers broad compatibility with various processing schemes based on its effective nucleation-control capability. As shown in Supplementary Fig. 7, effective single-crystal growth is achieved across a wide range of pattern densities (0.009–0.068 patterns per μm^2^). Furthermore, EF-SCN is applicable not only to crystalline substrates but also to amorphous substrates. Figure 2d shows optical microscope and SEM images of uniform single-crystal growth over a 400-μm area on amorphous SiO_2_. At nine randomly selected pattern sites, the bilayer MoS_2_ flakes shown in Supplementary Fig. 8 exhibit consistent orientations within each site, confirming that the underlying monolayer MoS_2_ is single-crystalline, although the crystal orientations vary randomly among different sites owing to the amorphous substrate.

**Extended Data Fig. 4:**
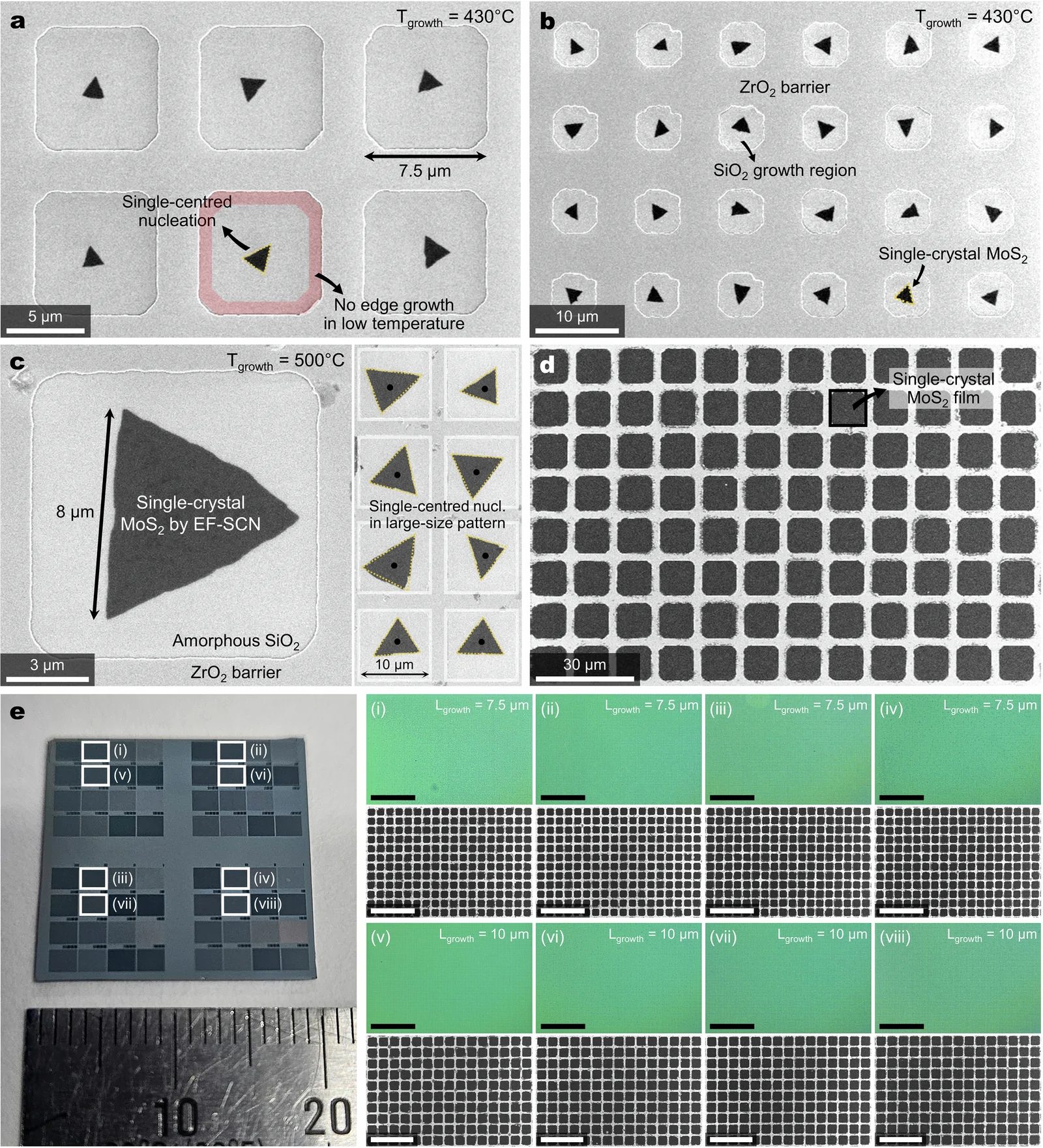
Low-temperature EF-SCN using a ZrO_2_ barrier. **a**,**b**, SEM images of single-crystal MoS_2_ grown at 430 °C by low-temperature MOCVD. The ZrO_2_ barrier suppresses MoS_2_ nucleation at the pattern edge by the etching-flux, resulting in well-defined single-centred nucleation within the amorphous SiO_2_ growth region. **c**,**d**, SEM images demonstrating effective EF-SCN at 500 °C in 10 μm patterns, showing both large single-crystal MoS_2_ flake (**c**) and a continuous film (**d**) growth. **e**, Wafer-scale demonstration of low-temperature EF-SCN on a 2 cm substrate. The photograph, together with OM and SEM images collected from eight representative locations, confirms uniform single-crystal growth across the substrate. Black scale bars, 500 μm; white scale bars, 50 μm.

Importantly, Fig. 2e–g shows single-crystal yield and uniformity on a large scale. Across 16 regions on a 2-cm substrate, 400 sites were fabricated with 25 patterns per region, achieving a single-crystal yield exceeding 99%. Extended Data Fig. 4 shows that engineering the etching-flux barrier material can extend EF-SCN to low-temperature processing. As discussed in Supplementary Note 2, the HfO_2_ barrier releases sufficient etching flux at 750 °C, whereas its reduced oxygen-releasing capability below 500 °C may hinder effective EF-SCN. Therefore, we used zirconium dioxide (ZrO_2_), which is known to have a lower crystallization temperature than HfO_2_, as an alternative etching-flux barrier^33,34^.

Extended Data Fig. 4a,b shows SEM images of MoS_2_ grown by EF-SCN at 430 °C on a SiO_2_ substrate patterned with a ZrO_2_ barrier. Despite the low-temperature process, uniform centred nucleation of well-defined triangular single-crystalline MoS_2_ domains is observed at the array level. Extended Data Fig. 4c,d shows the growth of large single-crystal at 500 °C, including an 8-μm single-crystal MoS_2_ flake and single-crystalline MoS_2_ films fully filling the 10-μm regions. Furthermore, Extended Data Fig. 4e shows the large-area uniformity of low-temperature EF-SCN. The photograph and the corresponding optical microscope and SEM images show uniform selective growth and single-crystalline MoS_2_ films across a 2-cm substrate. These results indicate that EF-SCN provides compatibility with low-temperature semiconductor processing and scalability towards large-area integration. Additional discussions on oxygen depletion from the etching-flux barrier and its impact are provided in Supplementary Note 4, along with Supplementary Fig. 9.

## Nucleation design rule

**Fig. 3:**
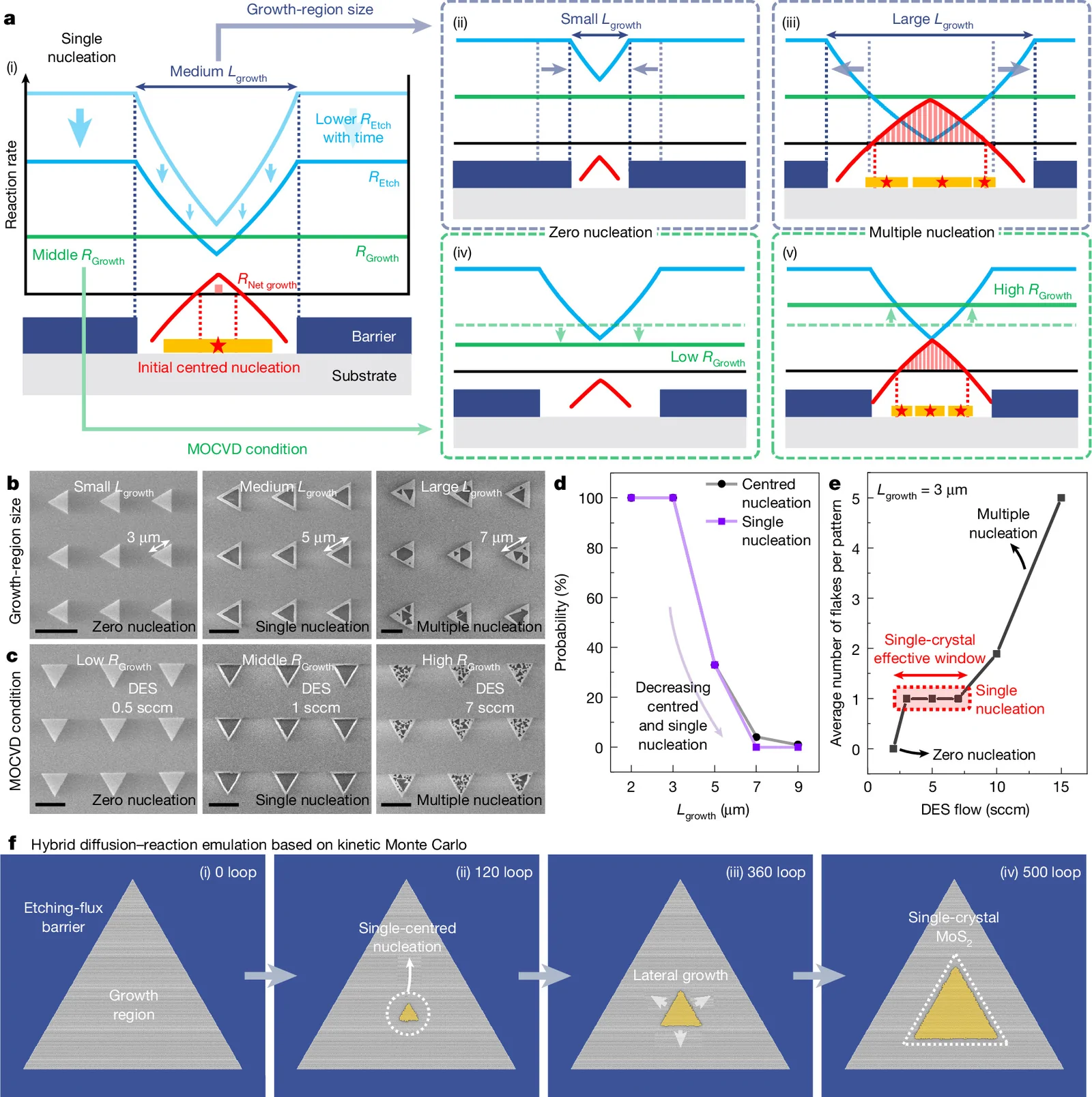
Nucleation design rule of 2D MoS_2_ EF-SCN. **a**, Schematic of zero–single–multiple nucleation control. On the basis of the single-nucleation case (i), zero and multiple nucleation can be controlled by the growth-region size in (ii) and (iii), and the MoS_2_ MOCVD conditions in (iv) and (v). **b**,**c**, SEM images of zero–single–multiple nucleation behaviour, depending on growth-region size (**b**) and MoS_2_ MOCVD conditions (**c**). **d**, MoS_2_ single (purple) and centred (black) nucleation probabilities depending on growth-region size. **e**, The average number of MoS_2_ flakes per pattern as a function of DES flow at *L*_growth_ = 3 μm. **f**, Simulation images illustrating single-centred nucleation and lateral-growth behaviour based on a hybrid diffusion–reaction emulation model, presented in a sequential loop. Scale bars, 5 μm (**b**,**c**). Source data

**Extended Data Fig. 5:**
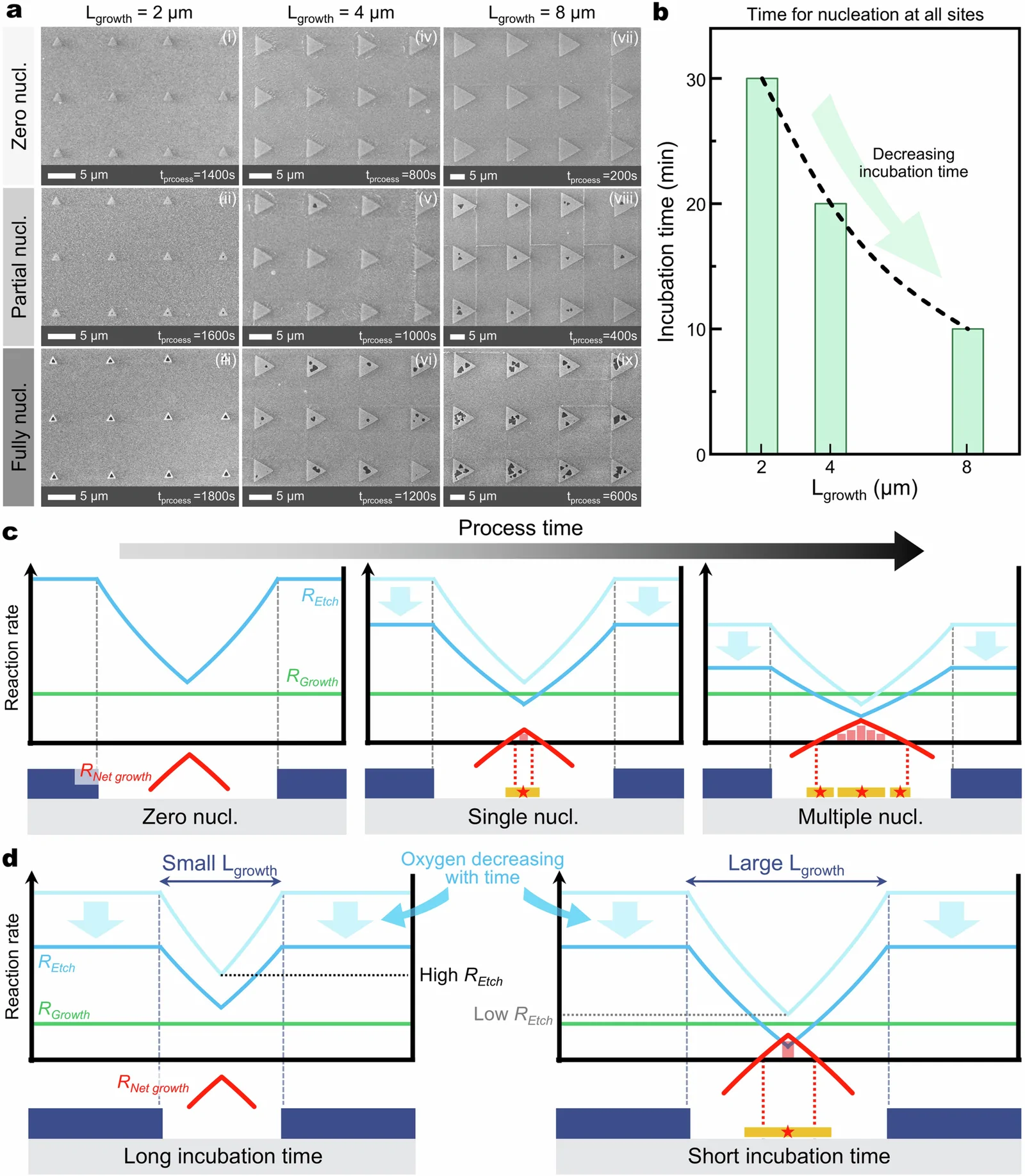
2D MoS_2_ nucleation behaviour with time. **a**, SEM images of MoS_2_ nucleation onset with process time. **b**, Incubation time of MoS_2_ nucleation with growth region size. **c**, Schematic of nucleation rate in the growth region with time. **d**, Comparison of MoS_2_ incubation times between small and large growth regions. Source data

Unlike conventional ASG approaches, EF-SCN achieves spatially programmed growth through a flux-mediated mechanism, enabling its nucleation behaviour to be systematically described and controlled within a physical framework. Figure 3 establishes a nucleation design rule for zero, single and multiple nucleation by varying the process time (*t*_process_), growth-region size and MOCVD conditions. As shown in Fig. 3a(i), single nucleation occurs when a NAR forms at the centre of the growth region at a specific *t*_process_. Extended Data Fig. 5a shows time-dependent MoS_2_ nucleation for different growth-region sizes (*L*_growth_) under low molybdenum hexacarbonyl (MHC) flow (2.5 sccm) and high diethyl sulfide (DES) flow (5 sccm). As *t*_process_ increases, oxygen release and the nucleation etching rate decrease, expanding the NAR and increasing nucleation (Extended Data Fig. 5c). The MoS_2_ incubation time also decreases with increasing *L*_growth_ (Extended Data Fig. 5b), because the lower etching rate at the centre of larger growth regions enables earlier NAR formation (Extended Data Fig. 5d). In summary, the nucleation-rate model of EF-SCN was confirmed by analysing the NAR formation with *t*_process_ and comparing the incubation time with *L*_growth_. Subsequently, a nucleation design rule is proposed by controlling *L*_growth_ and MOCVD conditions.

**Extended Data Fig. 6:**
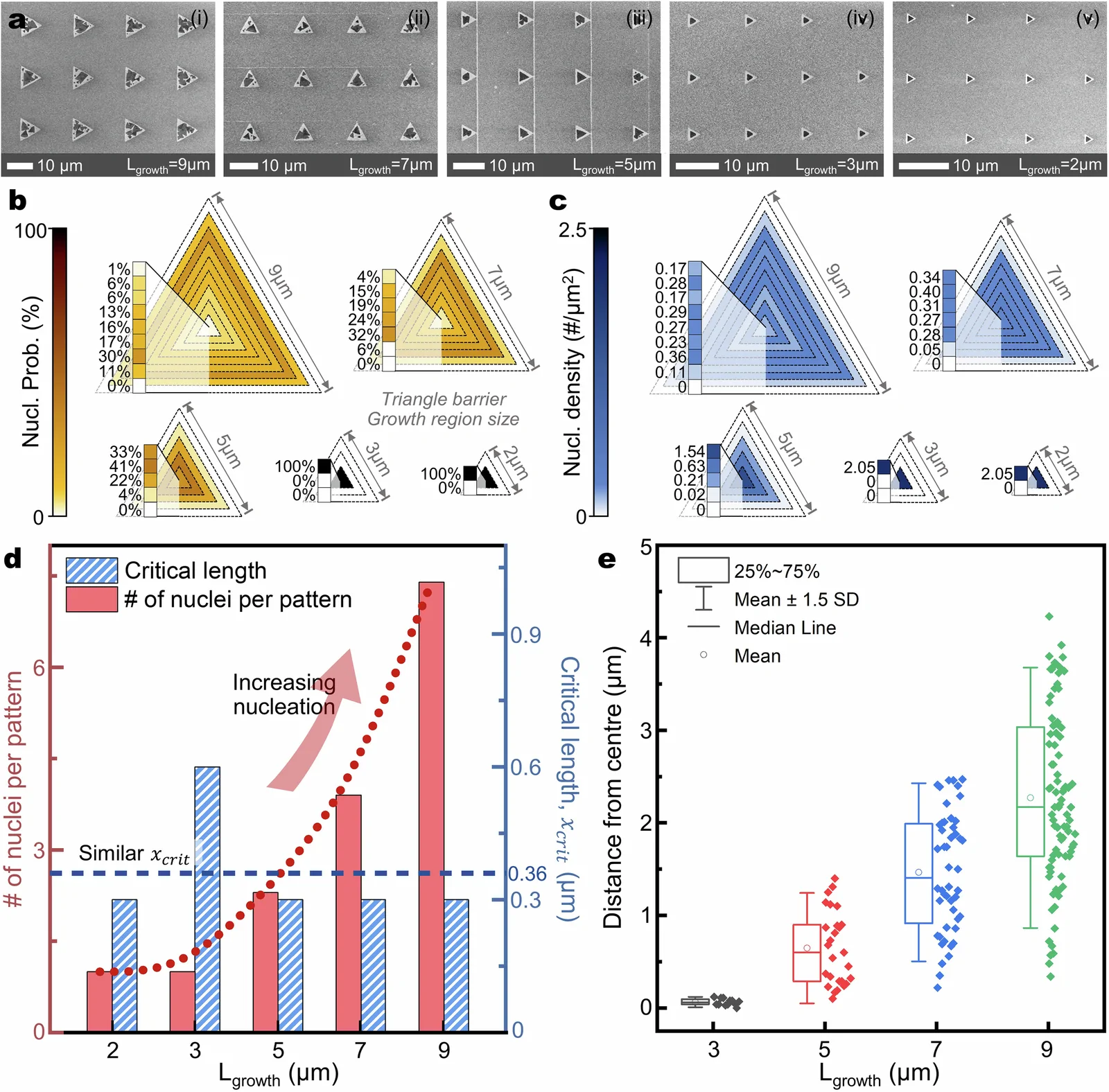
2D MoS_2_ nucleation behaviour with growth region size. **a**, SEM images of MoS_2_ nucleation with different growth region sizes. **b**,**c**, Contour maps showing the spatial nucleation probability (**b**) and density (**c**) of MoS_2_ for various growth region sizes. **d**, Bar graphs showing the number of nuclei per pattern (left, red-filled bars) and the critical length from the barrier edge (right, blue-hatched bars) versus growth region size. **e**, Box plots showing the distribution of distances from the pattern centre to the incentre of MoS_2_ flakes as a function of growth region size. In the plot, n = 12, 27, 48, and 88 for L_growth_ = 3, 5, 7, and 9 μm, respectively; box limits, 25th–75th percentiles; whiskers, mean ± 1.5 standard deviations; centre line, median; open circle, mean. Source data

In terms of growth-region sizes under fixed *t*_process_, Figure 3a(i)–(iii) shows that a smaller *L*_growth_ prevents NAR formation and yields zero nucleation, whereas a larger *L*_growth_ broadens the NAR and yields multiple nucleation. Figure 3b directly shows zero, single and multiple nucleation at *L*_growth_ values of 3 μm, 5 μm and 7 μm, respectively. Extended Data Fig. 6a–c spatially visualizes the nucleation positions within the growth region. Although the nucleation probability peaks near the HfO_2_ edge because the subunit area increases with distance from the centre (Extended Data Fig. 6b), nucleation-density mapping reveals saturation in the central area and distinct single-centred nucleation at *L*_growth_ values of 2 μm and 3 μm (Extended Data Fig. 6c). Figure 3d further shows that the probabilities of single and centred nucleation decrease markedly with increasing *L*_growth_, as determined from the data in Extended Data Fig. 6a. The critical length remains nearly constant, whereas the number and spatial spread of nuclei increase as the NAR expands (Extended Data Fig. 6d,e). Further details of this size dependence are provided in Supplementary Note 5.

Figure 3a(i),(iv),(v) illustrates the NAR and nucleation behaviour with different MOCVD conditions (that is, *R*_Growth_) under identical *t*_process_. Compared with the single-nucleation case, a lower *R*_Growth_ prevents NAR formation and leads to zero nucleation; whereas a higher *R*_Growth_ broadens the NAR width and leads to multiple nucleation. Because chalcogen flow critically affects MoS_2_ nucleation density, we investigated the effect of DES flow. Figure 3c shows zero, single and multiple nucleation at DES flows of 0.5 sccm, 1 sccm and 7 sccm, respectively. For *L*_growth_ = 3 μm, Fig. 3e and Supplementary Fig. 10 show zero nucleation at 2 sccm and multiple nucleation at 10–15 sccm, defining 3–7 sccm as an effective window for single-crystal MoS_2_ growth. Supplementary Figs. 11 and 12 further show that the threshold DES flow increases as *L*_growth_ decreases and distinguish DES flow as the primary control of nucleation density and rate, whereas MHC flow primarily controls lateral growth. Thus, effective single-crystal growth requires relatively low DES flow and high MHC flow. Supplementary Note 5 further details the distinct roles of DES and MHC flows, and the valid single-crystal growth strategy is discussed in Supplementary Note 6 with Supplementary Figs. 13 and 14.

**Extended Data Fig. 7:**
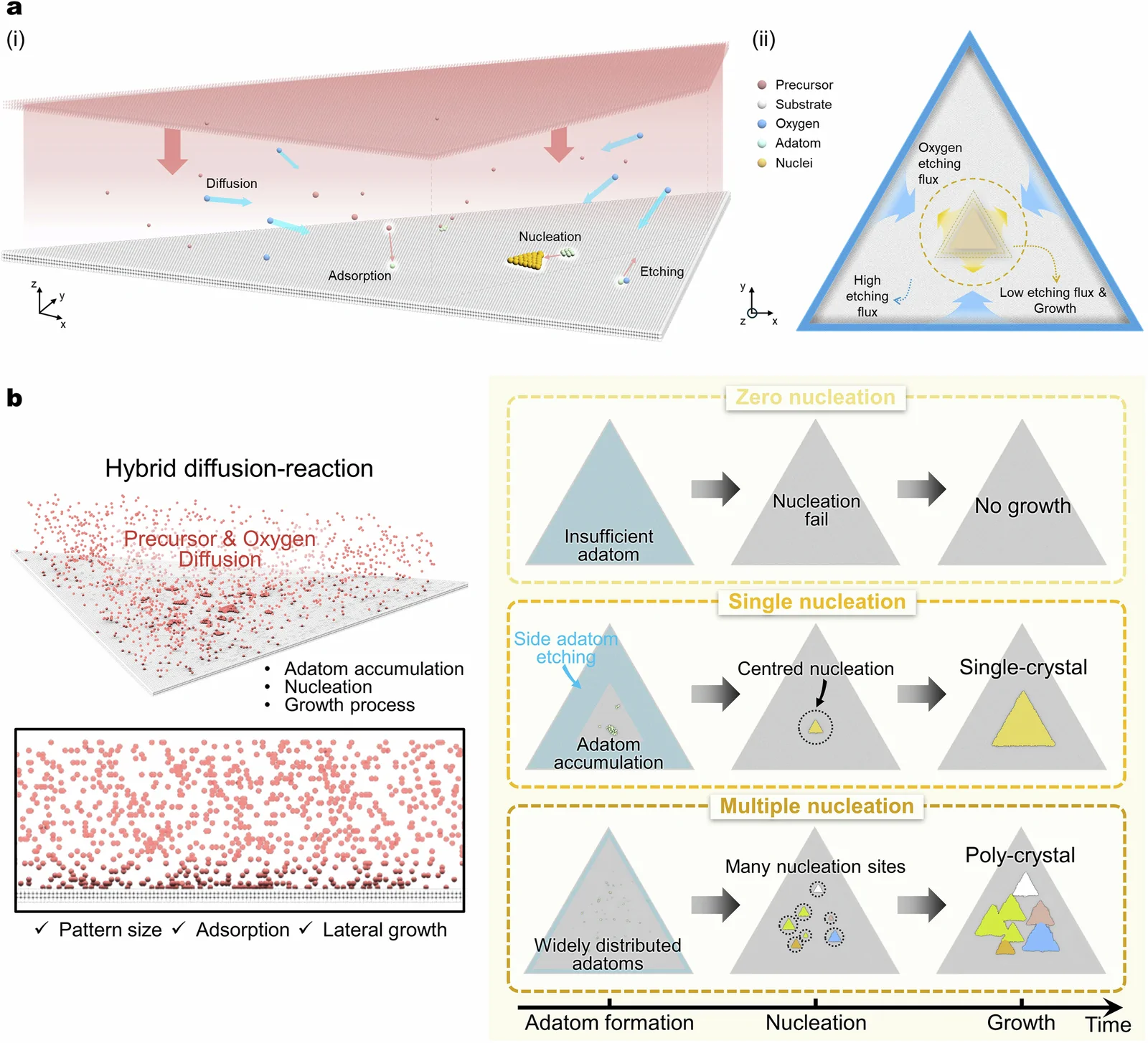
Hybrid diffusion-reaction process emulation of the EF-SCN nucleation regimes. **a**, Schematic of the emulation framework: orthogonal view of the triangular growth template with physical processes (i) and top view of the oxygen and precursor flux directions (ii). **b**, Time evolution of the zero-, single-, and multiple-nucleation outcomes.

To further examine whether spatially separated precursor supply and etching flux can generate EF-SCN behaviour, we performed kinetic Monte Carlo (kMC)-based hybrid diffusion–reaction emulation. In Extended Data Fig. 7, precursor species are supplied from above and HfO_2_-derived oxygen etchants from the side walls, with adsorption, nucleation, lateral growth and etching implemented as minimal reaction rules through a cellular-automaton-based scheme^35–37^. Using template size, lateral-growth probability and adsorption probability as counterparts to *L*_growth_, MHC flow and DES flow, the emulation reproduced the zero-, single- and multiple-nucleation regimes. Under the single-nucleation condition, Fig. 3f shows centre nucleation followed by lateral growth, consistent with a centre-localized NAR; the temporal evolution is shown in Supplementary Videos 1 and 2. Supplementary Figs. 15 and 16 further reproduce the parameter-dependent regimes and NAR/NSR distribution. Overall, the emulation supports the experimentally established design rule, with detailed assumptions and parameters provided in Supplementary Note 7.

**Fig. 4:**
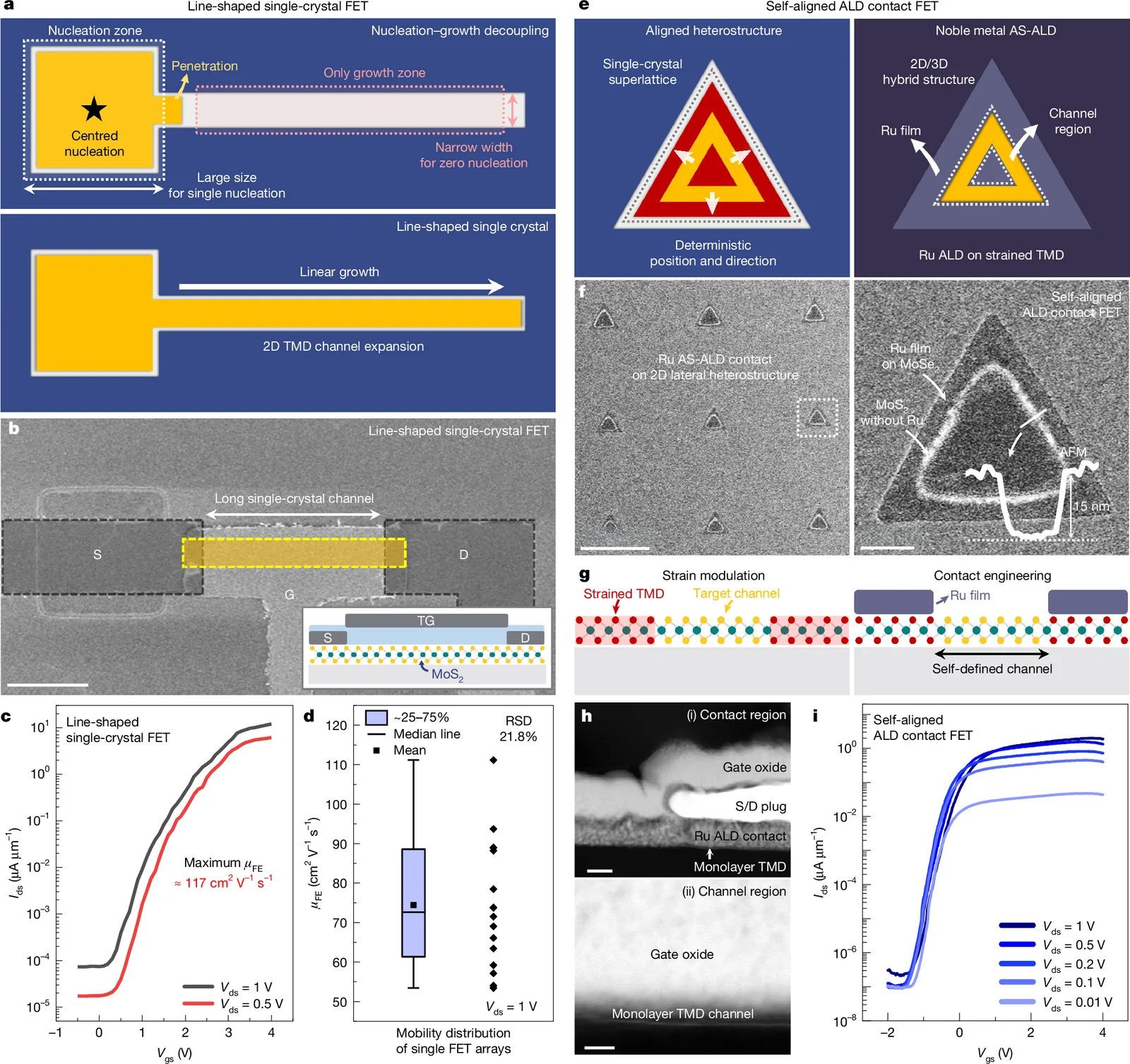
Two-dimensional TMD single-crystal architectures based on the EF-SCN method. **a**, Schematic of the nucleation–growth decoupling strategy for line-shaped single-crystal 2D MoS_2_ growth. **b**, SEM image of a line-shaped single-crystal MoS_2_ single FET. The source (S), drain (D) and gate (G) electrodes are indicated, and the inset image shows a cross-sectional view of the top gate (TG) device. **c**, Transfer curves of the line-shaped single FET. *I*_ds_, *V*_gs_, *V*_ds_ and *μ*_FE_ denote the drain–source current, gate–source voltage, drain–source voltage and field-effect mobility, respectively. **d**, Statistical distribution and box plot of the field-effect mobility for an array of 16 single FETs. In the plot, box limits are the 25th–75th percentiles, the centre line is the median, and the square symbol is the mean. RSD is the relative standard deviation (21.8%). **e**–**g**, Top-view schematic (**e**), SEM images (**f**) and cross-sectional schematic (**g**) of Ru AS-ALD on aligned 2D MoS_2_–MoSe_2_ lateral heterostructures for self-aligned ALD contacts. The inset graph in **f** shows the AFM height profile along the white line. **h**,**i**, HAADF-STEM images (**h**) and transfer curves (**i**) of self-aligned ALD contact FETs. Scale bars, 3 μm (**b**); 10 μm (**f**(left)); 1 μm (**f**(right)); 20 nm (**h**(top)); 5 nm (**h**(bottom)). Source data

Together, Figs. 1–3 establish EF-SCN as a deterministic and process-compatible platform for controlling the position, dimensions and nucleation mode of single-crystal 2D TMD. Building on this spatial control, Fig. 4 shows functional single-crystal architectures, including multiple FETs formed through nucleation-growth decoupling and self-aligned ALD contact FETs based on programmed lateral heterostructures.

## Spatially programmed single-crystal architectures

**Extended Data Fig. 8:**
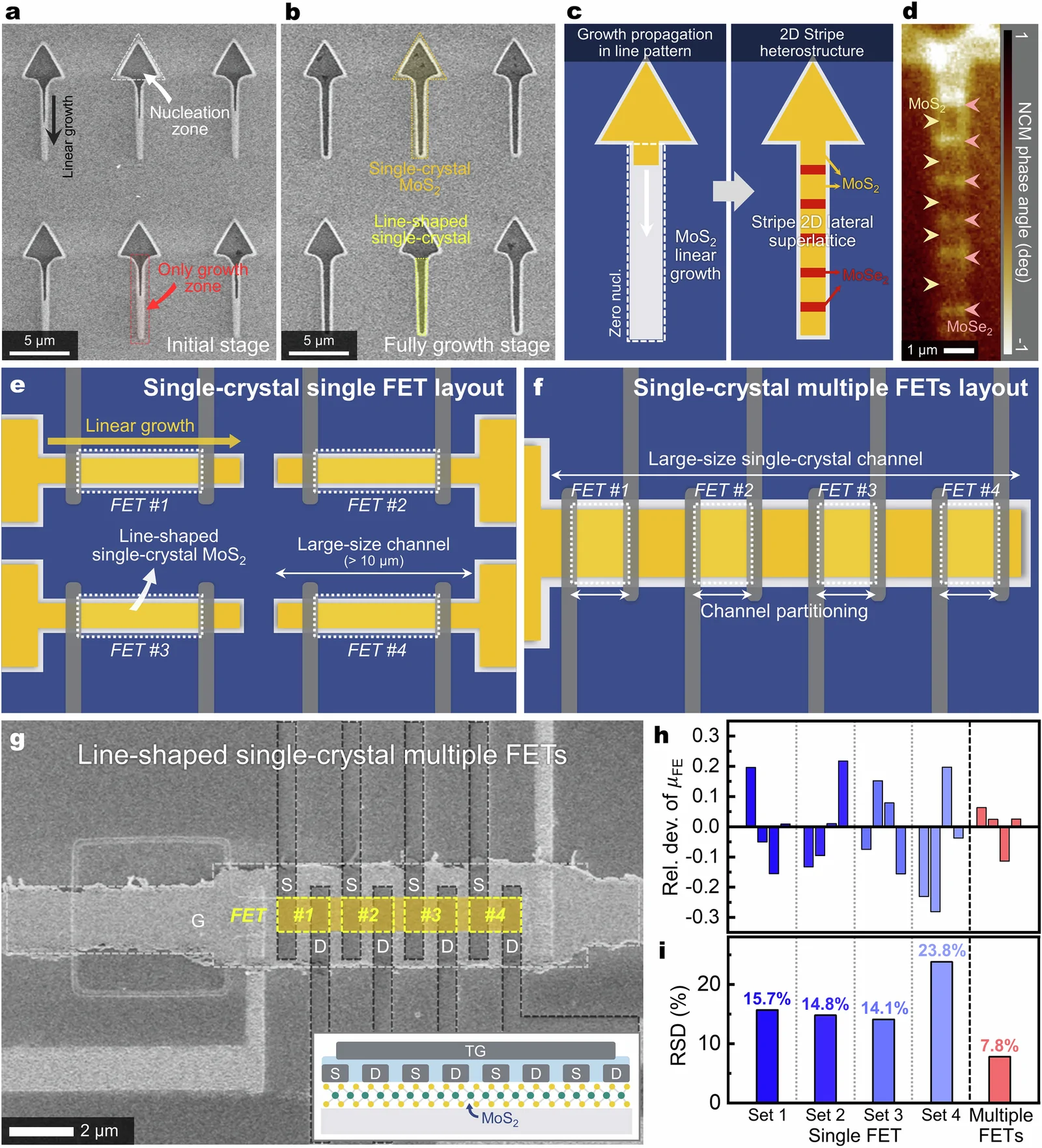
Nucleation-growth decoupling strategy and single-crystal multiple FETs. **a**,**b**, SEM images showing line-shaped single-crystal MoS_2_ growth via the nucleation-growth decoupling strategy at the initial stage (**a**) and the fully grown stage (**b**). **c**,**d**, Schematic (**c**) and NCM phase mapping image by AFM (**d**) of a stripe 2D MoS_2_-MoSe_2_ lateral heterostructure. **e**,**f**, Schematics illustrating the layouts of a single-crystal single FET (**e**) and single-crystal multiple FETs (**f**) layouts. **g**, SEM image of single-crystal MoS_2_ multiple FETs. **h**,**i**, Relative mobility deviation (**h**) and relative standard deviation (**i**) of single FET arrays and multiple FETs. Source data

Figure 4a schematically illustrates the nucleation-growth decoupling strategy for 2D MoS_2_ with programmed shape. The EF-SCN system allows control over single, multiple and zero nucleation, all tunable by *L*_growth_. Therefore, by integrating a large-size nucleation zone with a small-size only growth zone where nucleation is suppressed, the positions of nucleation and growth can be spatially decoupled. The SEM image in Extended Data Fig. 8a confirmed that a MoS_2_ flake nucleated in the nucleation zone penetrated the only growth zone, resulting in a line-shaped single-crystal MoS_2_ (Extended Data Fig. 8b). We further extended the strategy to fabricate a single-crystal stripe 2D MoS_2_–MoSe_2_ heterostructure (Extended Data Fig. 8c), which is confirmed by non-contact-mode phase mapping (Extended Data Fig. 8d). Collectively, these observations demonstrate that the nucleation–growth decoupling strategy is not limited to enlarging crystal size but also enables deliberate shape engineering of single-crystal 2D TMD beyond the conventional triangular morphology.

**Extended Data Fig. 9:**
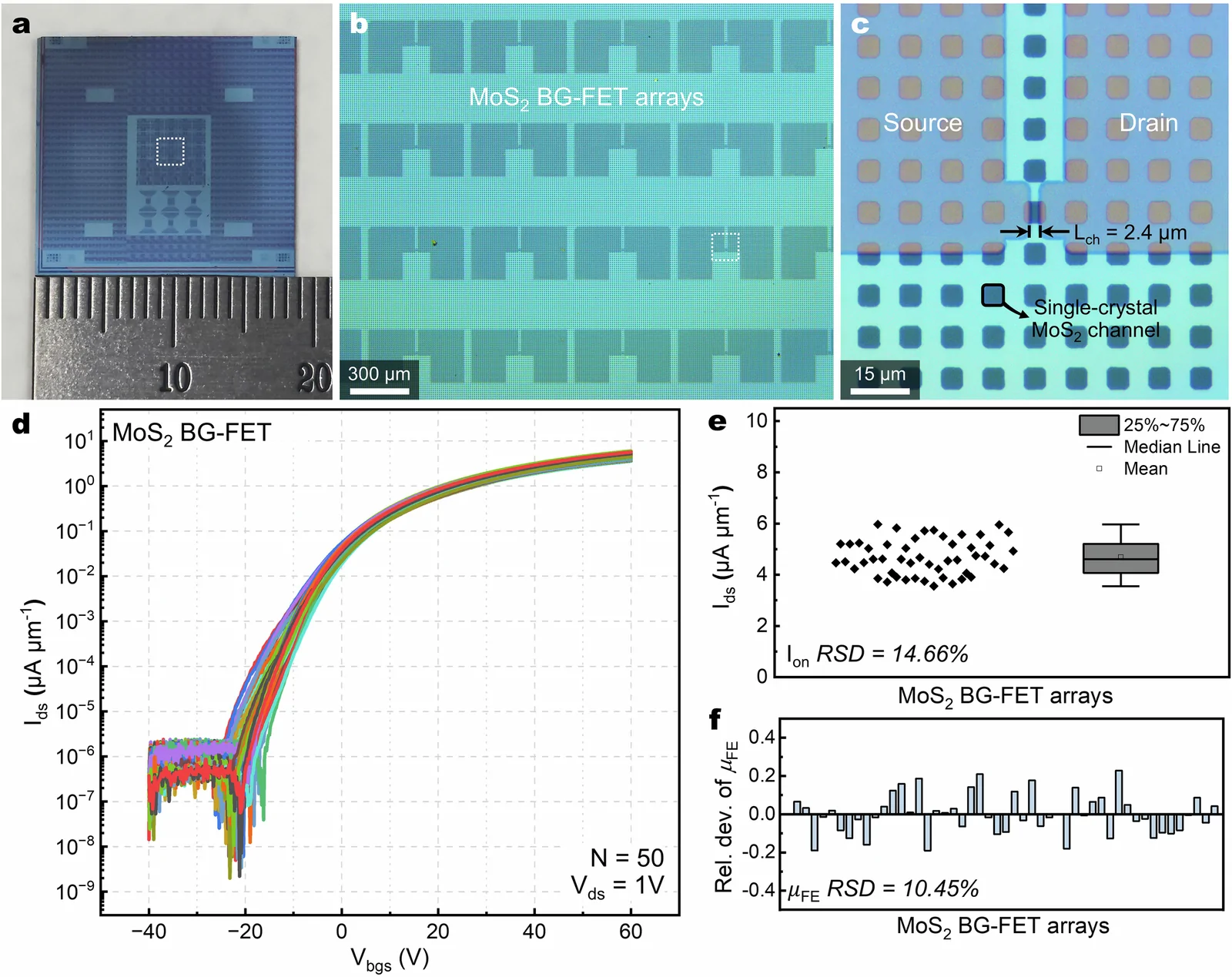
Electrical characteristics and device-to-device uniformity of single-crystal MoS_2_ BG-FET arrays by EF-SCN. **a**, Photograph of the chip containing the MoS_2_ BG-FET arrays. **b**,**c**, OM images of the device arrays shown in **a** at higher magnifications. **d**, Transfer curves of 50 MoS_2_ BG-FETs measured at V_ds_ = 1 V. **e**,**f**, Distribution of the on-current density (**e**) and relative deviations of the field-effect mobility (**f**) for 50 devices. In the box plot, box limits, 25th–75th percentiles; centre line, median; open square, mean; RSD, relative standard deviation of on-current density (14.66%). Source data

To evaluate the quality and device potential of the line-shaped 2D TMD channel, we fabricated FETs with the structure shown in Fig. 4b and Extended Data Fig. 8e with an average channel length of 6.8 μm and width of 0.83 μm, using 10-μm-long single-crystal MoS_2_. As shown in the transfer curve in Fig. 4c, the line-shaped single FET exhibits typical n-type behaviour, with an on-current density of 12 μA μm^−1^. Importantly, a high field-effect mobility of 117 cm^2^ V^−1^ s^−1^ was achieved, surpassing previously reported values for selectively grown MoS_2_ FETs, indicating the excellent electrical properties of single-crystal MoS_2_ grown by EF-SCN^8,9^. In Fig. 4d, the mobility distribution across a total of 16 single FET arrays reveals an average mobility of 74 cm^2^ V^−1^ s^−1^ with an RSD of 21.8%, demonstrating a performance variation comparable to that of previous 2D TMD single-crystal FETs. In addition, to evaluate the large-area uniformity of the EF-SCN system, we analysed the performance of 50 back-gated FETs fabricated using single-crystal MoS_2_ channels grown across a 2-cm substrate. As shown in Extended Data Fig. 9, the devices exhibited RSDs of 10.45% in mobility and 14.66% in on-current density, supporting the superior uniformity of the EF-SCN system over a large area. Further details are provided in Supplementary Note 8.

Meanwhile, the long single-crystal channels synthesized via the nucleation–growth decoupling strategy render high-density and low-variation device layouts feasible within a confined footprint. We therefore propose single-crystal multiple FETs, in which multiple transistors are defined along a single channel (Extended Data Fig. 8f). As shown in Extended Data Fig. 8g, the single-crystal multiple FETs consist of 4 active channels, each with a length of 0.31 μm and a width of 0.87 μm, integrated within a 10-μm-long single-crystal MoS_2_. Comparing the relative deviation and RSD of field-effect mobility, single-crystal single FETs (4 sets, 16 devices) showed an overall RSD of 21.8%, whereas single-crystal multiple FETs (4 devices) showed a markedly lower RSD of 7.8%, which is a 65% reduction in variation (Extended Data Fig. 8h,i). This indicates that fabricating multiple channels within a single crystal enables lower variation than defining a single channel in a single crystal, suggesting that the single-crystal scaling capability of EF-SCN holds strong potential for low-variation device architectures.

**Extended Data Fig. 10:**
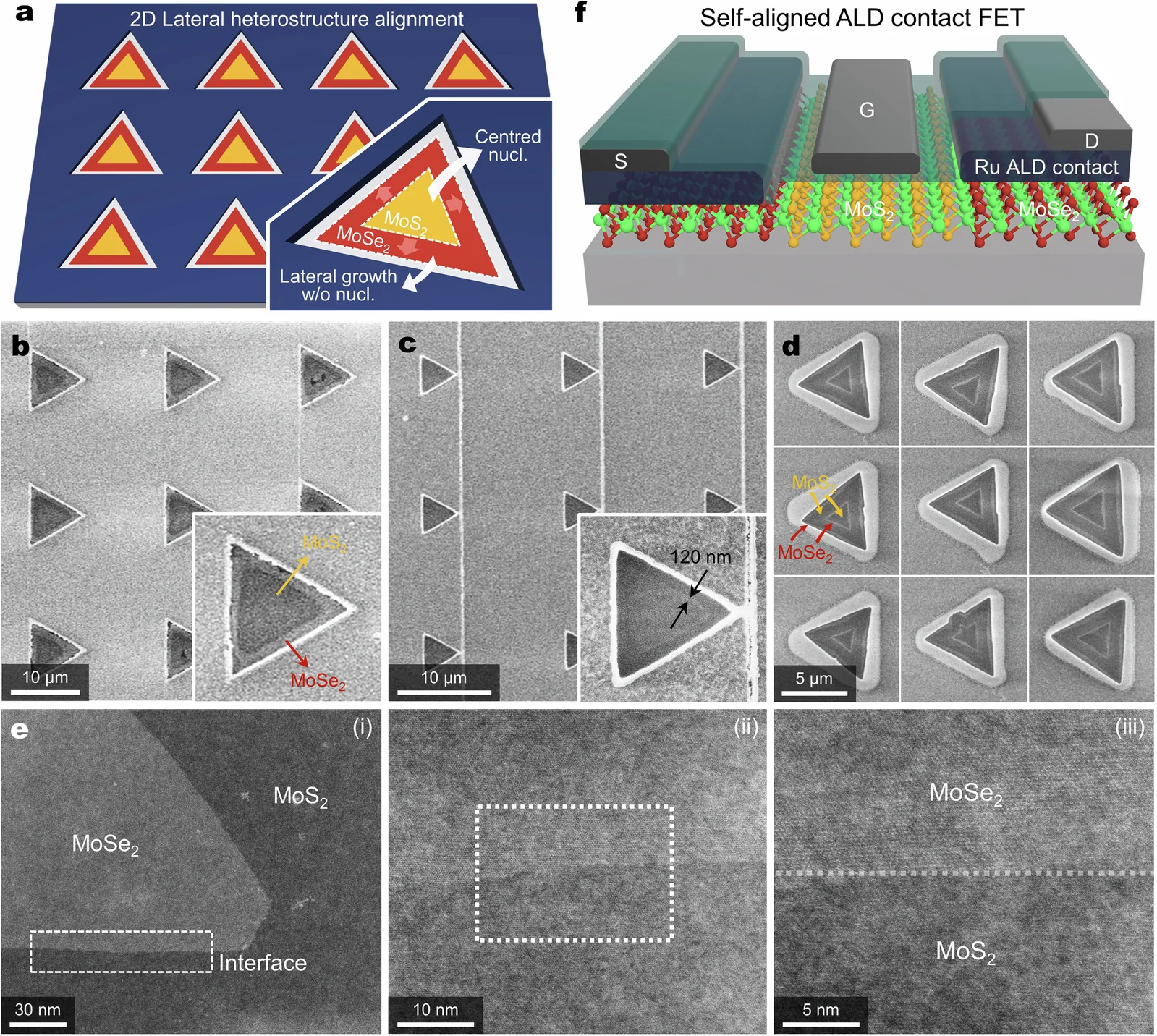
Alignment of 2D TMD lateral heterostructure for self-aligned ALD contact FET. **a**–**d**, Schematic (**a**) and SEM images (**b**–**d**) of aligned 2D MoS_2_-MoSe_2_ lateral heterostructure by the EF-SCN method. **e**, HAADF-STEM images of the MoS_2_-MoSe_2_ lateral heterostructure interface with an intermixing width of approximately 1–2 nm. **f**, Schematic of self-aligned ALD contact FET with Ru AS-ALD on 2D MoS_2_-MoSe_2_ lateral heterostructure.

Conventional selective-growth approaches, although effective in producing high-quality single-crystal TMDs, are inherently limited to applications using homogeneous channel or vertical heterostructure formation, leaving in-plane lateral compositional control less accessible^8,9^. However, lateral heterojunctions are important for device engineering, as they enable direct modulation of electronic properties along the channel direction. In this regard, the EF-SCN system offers a distinct route to the in-plane alignment of lateral heterostructures through precise spatial regulation of nucleation and lateral growth, as shown in Extended Data Fig. 10a–d. We successfully synthesized 2D MoS_2_–MoSe_2_ lateral superlattices with precise positional and orientational alignment at the array level. As shown in the HAADF-STEM image in Extended Data Fig. 10e, these lateral heterostructures exhibit sharp interfaces with an intermixing width of less than 2 nm. Such spatially programmed growth introduces contact engineering strategies via strain modulation at the 2D TMD heterojunctions, enabling selective metal contact formation via ALD on the MoSe_2_ region in MoS_2_–MoSe_2_ lateral heterostructures^38^ (Extended Data Fig. 10f).

Figure 4e presents a schematic illustrating the fabrication of self-aligned ALD contact FETs. Ruthenium (Ru) ALD on the MoS_2_–MoSe_2_ lateral heterostructures grown by EF-SCN selectively deposits on the MoSe_2_ region owing to local strain distribution (Fig. 4g). As shown in Fig. 4f, a well-aligned 2D/3D hybrid structure is achieved at the array level, and AFM measurements confirm the formation of 15-nm-thick Ru film. Using this Ru film as a contact, self-aligned ALD contact FETs were fabricated with a conformal Ru layer at the contact region on the TMD surface where monolayer MoS_2_ is preserved in the channel region, as verified by HAADF-STEM (Fig. 4h). The device with a channel width of 2.5 μm and length of 1.4 μm exhibited a typical n-type behaviour under drain voltages ranging from 0.01 V to 1 V (Fig. 4i). The maximum electrical performances include a field-effect mobility of 4.3 cm^2^ V^−1^ s^−1^ (*V*_ds_ = 0.01 V), an on/off ratio exceeding 10^7^, an on-current density of 2.0 μA μm^−1^ and a subthreshold swing of 170 mV dec^−1^. Despite the harsh conditions of Ru ALD (oxygen exposure at 220 °C) expected to degrade electrical properties of the channel, the device delivers meaningful performance considering previous reports on direct ALD contact-based 2D TMD FETs^39,40^.

Collectively, these results demonstrate that the deterministic nucleation control and precise crystal orientation alignment of EF-SCN facilitate expansion towards advanced device applications that remain inaccessible with conventional approaches (see Supplementary Note 9 and Supplementary Table 4 for a detailed comparison with conventional confined growth). This is evidenced by high-performance and low-variation FETs fabricated through large-size, shape-controlled single-crystal growth, as well as self-aligned ALD contact FETs enabled by spatially controlled lateral heterostructures. Therefore, the EF-SCN system serves as a foundational platform for advanced 2D material devices, ranging from homogeneous single-crystal channel applications such as high-density logic circuits to heterostructure-based applications, including spatial doping for rectification, heterojunction design for optoelectronics, strain modulation in 2D materials, edge contacts via lateral growth and anisotropic 2D materials^41–48^.

## Conclusion

We demonstrate that the EF-SCN system establishes a physical framework for selective growth by enabling deterministic control over nucleation position for effective single-crystal growth and multifunctional 2D TMD architectures. Unlike conventional 2D TMD ASG, reactive species released from the etching-flux barrier create a non-uniform nucleation probability distribution and localize a single-nucleation site at the pattern centre, enabling spatially controlled MoS_2_ single crystals up to 10 μm. The platform also exhibits broad process compatibility, including low-temperature growth, amorphous substrates and large-scale implementation. Nucleation-rate modelling further establishes a quantitative design rule for zero, single and multiple nucleation as functions of growth-region size, process time and MOCVD conditions. Extending this concept, nucleation–growth decoupling produces line-shaped single-crystal MoS_2_ and programmable stripe heterostructures, enabling field-effect mobilities up to 117 cm^2^ V^−1^ s^−1^ and multiple FETs within a single crystal with low device-to-device variation. Precise positional and orientational control also enables self-aligned ALD contact FETs through spatially programmed 2D/3D heterostructures with Ru AS-ALD. More broadly, the in-plane regulation of nucleation and growth provides a route to strain modulation, contact engineering and other complementary-metal–oxide–semiconductor-relevant functions within a selective-growth sequence. These capabilities highlight EF-SCN as a versatile platform for translating channel design, stressor formation and doping strategies into the 2D materials regime.

## Methods

### MOCVD for 2D TMD EF-SCN

The monolayer MoS_2_ was grown in a home-made horizontal hot-wall quartz tube, with MHC (Sigma-Aldrich), DES (Sigma-Aldrich), hydrogen (H_2_), potassium chloride (KCl) (Sigma-Aldrich) and argon carrier gas. The flow rate was controlled by the mass flow controller. The MOCVD process was conducted at 430–800 °C and 1–20 torr for 200–3,000 s. The EF-SCN behaviour was controlled by adjusting the MOCVD conditions, with DES flow rates of 0.5–20 sccm and MHC flow rates of 2–5 sccm, depending on the nucleation type. The monolayer MoS_2_–MoSe_2_ lateral superlattice was grown by alternating supply of DES and dimethyl selenide (Alfa Aesar). The MoS_2_ MOCVD process was mainly carried out on *c*-plane sapphire (Madelab), *c*/*a* miscut sapphire (0.5–5°, MTI Korea) and amorphous SiO_2_ (Waferbiz) patterned with etching-flux barrier. To compare the MoS_2_ growth behaviour, not only *c*-plane sapphire but also a-Al_2_O_3_, a-SiO_2_ and a-HfO_2_ deposited on Si(100) by an electron-beam evaporator (Atech) were used as substrates.

### Etching-flux barrier pattern fabrication

The etching-flux barrier was mainly fabricated on a sapphire substrate using electron-beam lithography (VEGA3 LMH/ELPHY quantum lithography system). Poly(methyl methacrylate) (PMMA; 1000 HARP PMMA eB 0.7, KemLab) was spin-coated on the substrate at 4,000 rpm and baked at 180 °C for 3 min. Subsequently, a conductive protective coating solution (AR-PC 5092, Allresist) was spin-coated at 2,000 rpm and baked at 90 °C for 2 min. After patterning process by electron-beam lithography and removing the AR-PC with deionized water, the electron-beam-exposed region was developed by methyl isobutyl ketone (MIBK) and isopropyl alcohol (IPA) solution (MIBK/IPA 1:3 positive radiation resist developer, KAYAKU) for 15–20 s, followed by cleaning in IPA for 20 s. Finally, the etching-flux barrier was fabricated by depositing 5–20 nm of HfO_2_ (or ZrO_2_) using an electron-beam evaporator, after which a lift-off process in acetone at 70 °C for 30 min was conducted. For the large-scale demonstration, photolithography was used to fabricate the barrier patterns. AZ 5214E photoresist (Merck Performance Materials) was spin-coated at 4,000 rpm and baked at 115 °C for 60 s. The photoresist was then exposed for 10–14 s using a mask aligner (Midas MDA-600S) and developed in AZ 300 MIF solution (Merck Performance Materials) for 45 s. Subsequently, the barrier material was deposited using an electron-beam evaporator, followed by lift-off in dimethyl sulfoxide (Sigma-Aldrich).

### Characterization of 2D TMD EF-SCN

The 2D TMD EF-SCN results were mainly measured with a field-emission scanning electron microscope (FE-SEM, Hitachi S-4800). The MoS_2_ thickness was confirmed by AFM (NX10, Park Systems) in non-contact mode, and non-contact-mode phase mapping was also performed with the same instrument. The Raman and photoluminescence spectra of MoS_2_ were measured using a confocal Raman spectroscopy system (LabRAM ARAMIS, Horiba) equipped with an argon ion laser of 514.5 nm wavelength. Photoluminescence mapping was performed using a confocal Raman spectroscopy (XperRAM C, Nanobase) with a 532-nm laser, 2,400 grooves per mm grating and a reference peak at 680 nm (1.82 eV). HAADF-STEM images of single-crystal MoS_2_ and self-aligned ALD contact FETs were obtained using FEI Titan3 G2 60-300 (FEI) operated at 200 kV and 300 kV. Atomic-resolution AFM measurements were performed in ultrahigh vacuum (5.0 × 10^−10^ mbar) at room temperature using contact mode. In second-harmonic generation (SHG) measurement, the wide-field SHG images were obtained using a femtosecond laser system (Light Conversion PHAROS). The excitation pulses were generated using an optical parametric amplifier (ORPHEUS) providing a wavelength of 1,300 nm at a repetition rate of 100 kHz. The polarization-resolved SHG signals were measured using a femtosecond titanium:sapphire laser operating at a central wavelength of 800 nm with a repetition rate of 80 MHz. To generate contour mapping images for nucleation probability and density, SEM images were converted into a binary image using ImageJ. Then, each growth region was divided into evenly spaced subunit areas and flake centres were counted within each subunit area.

### Thermal annealing and characterization of etching-flux barrier

To investigate the oxygen emission from the HfO_2_ etching-flux barrier, 50-nm HfO_2_ deposited on Si(100) substrate by an electron-beam evaporator was thermally annealed at 750 °C for 2–360 min under vacuum with argon gas. The compositional analysis of HfO_2_ was evaluated by XPS (Nexsa G2, Thermo Fisher Scientific). The XPS measurements were operated at room temperature under high vacuum (<2 × 10^−8^ torr) in constant analyser energy mode with a pass energy of 50.0 eV, a step size of 0.05 eV and a beam diameter of 400 μm. The XPS peaks were fitted using Thermo Avantage software. To examine the phase and crystal structure changes of HfO_2_ depending on annealing temperature (350–850 °C), grazing incidence X-ray diffraction (Ultima IV, RIGAKU) measurement was performed with Cu-Kα radiation (*λ* = 1.541 Å). The X-ray diffraction data were analysed using PDXL software. For analysis of the MoS_2_ etching behaviour by HfO_2_, MoS_2_ flakes were first grown on Si/SiO_2_ (90 nm) substrates via MOCVD and subsequently dry-transferred onto sapphire substrate patterned with 20-nm HfO_2_ barrier. PMMA was spin-coated onto the MoS_2_-grown substrate at 8,000 rpm for 40 s and baked at 180 °C for 10 min. The PMMA/MoS_2_ flakes stack was dry-transferred onto a HfO_2_-patterned sapphire substrate using a thermal release tape and polydimethylsiloxane. PMMA was then removed using acetone, and the sample was rinsed with IPA. Finally, the transferred samples were thermally annealed at 750 °C for 1 h under vacuum with argon gas.

### Ruthenium metal AS-ALD

Ru films were deposited using a travelling-wave-type ALD with (ethylbenzyl)(1-ethyl-1,4-cyclohexadienyl)Ru(0) (EBECHRu, Hansol Chemical) as the precursor and O_2_ as the reactant gas. The EBECHRu precursor was stored in a stainless-steel canister and heated to 100 °C to obtain sufficient vapour pressure, and the delivery lines were maintained at 10–15 °C higher than the bubbler temperature to prevent condensation. The ALD process was conducted at a stage temperature of 220 °C, and each unit cycle consisted of an EBECHRu pulse (3 s), nitrogen (N_2_) purge (45 s), O_2_ pulse (3 s) and N_2_ purge (45 s). High-purity N_2_ gas (99.999%) with a flow rate of 200 sccm was used for purging residual gas molecules and by-products.

### Hybrid diffusion–reaction process emulator by kMC

Hybrid diffusion–reaction process emulation was used to test whether spatially separated precursor supply and oxygen etching flux can reproduce the zero–single–multiple nucleation behaviour of EF-SCN. The emulation domain was defined as a simplified triangular growth template on a hexagonal lattice, with a precursor reservoir positioned above the template and an oxygen reservoir placed along the template boundary to mimic HfO_2_-derived etching species. Diffusion and reaction steps were alternated using a cellular-automaton-based scheme^35–37^, in which the reaction step included the essential events required for EF-SCN: precursor adsorption, nucleus formation, lateral growth and oxygen-mediated etching.

Process-condition effects were represented by varying schematic parameters corresponding to template size, adsorption and nucleation probability, lateral-growth probability, and etching-flux supply. These parameters were used to capture the experimental effects of *L*_growth_, DES-controlled nucleation density and MHC-controlled lateral-growth rate. The resulting outcomes were classified as zero, single or multiple nucleation according to the number of surviving nuclei, and the spatial distribution of nucleation events was used to identify NAR and NSR formation. Complete lattice definitions, update rules, parameter values, seed statistics, and NAR and NSR quantification procedures are described in Supplementary Note 7.

### Device fabrication and electrical characterization

A top-gated FET based on single-crystal line-shaped MoS_2_ was fabricated using electron-beam lithography. A gold (Au) layer (40 nm) was deposited via electron-beam evaporation to form the source, drain and top-gate electrodes. The top-gate dielectric was prepared by depositing a 0.7-nm Si seed layer via electron-beam evaporation, followed by 8 nm HfO_2_ using ALD. The dielectric capacitance of 6.2 × 10^−7^ F cm^−2^ was confirmed by employing capacitance–voltage measurements on metal–insulator–metal capacitors. A back-gated FET array was fabricated using photolithography. Source and drain electrodes were formed by thermal evaporation of a 40-nm Au layer. For encapsulation, a 1-nm Si seed layer was first deposited by electron-beam evaporation, and a 10-nm HfO_2_ layer was subsequently deposited by plasma-enhanced ALD. For a 2D TMD FET with Ru AS-ALD contact, a source–drain isolation process was required to separate the continuous Ru film formed outside the superlattice region. The Ru layer was etched using inductively coupled plasma reactive ion etching with a mixed gas of O_2_ (24 sccm) and carbon tetrafluoride (CF_4_, 6 sccm), using PMMA as the etching mask. The source, drain and top-gate electrodes were deposited by electron-beam evaporation of Au (30 nm). The top-gate dielectric was prepared by depositing a 0.7-nm Si seed layer, followed by 30-nm HfO_2_. The dielectric capacitance of 8.5 × 10^−7^ F cm^−2^ was confirmed by using capacitance–voltage measurements on metal–insulator–metal capacitors. Electrical measurements of the back-gated FET array were performed at room temperature under vacuum using a semiconductor analyser (B1500A, Keysight), and those of the other devices were performed at room temperature under ambient conditions using a semiconductor analyser (4200A-SCS, Keithley).

## Online content

Any methods, additional references, Nature Portfolio reporting summaries, source data, extended data, supplementary information, acknowledgements, peer review information; details of author contributions and competing interests; and statements of data and code availability are available at https://doi.org/10.1038/s41586-026-11095-1.

## Supplementary information

Supplementary Information Supplementary Figs. 1–16, Tables 1–4, Notes 1–9 and References.

Peer Review File

Supplementary Data Source data for Supplementary Figs. 2, 4, 9, 11 and 14.

Supplementary Video 1 Time evolution of the size-dependent nucleation regimes in the kMC-based hybrid diffusion–reaction process emulation.

Supplementary Video 2 Time evolution of centred nucleation and shape-programmed growth in the kMC-based hybrid diffusion–reaction process emulation.

## Source data

Source Data Fig. 2

Source Data Fig. 3

Source Data Fig. 4

Source Data Extended Data Fig. 1

Source Data Extended Data Fig. 3

Source Data Extended Data Fig. 5

Source Data Extended Data Fig. 6

Source Data Extended Data Fig. 8

Source Data Extended Data Fig. 9

## Data Availability

Source data are provided with this paper. All other data supporting the results of this study are available in the paper and Supplementary Information.
