# Supplementary material for: Spatially deterministic nucleation of 2D semiconductors by etching flux
Source: Nature. 2026 Oct 7;658(8135):398–406. doi: 10.1038/s41586-026-11095-1 (PMC13645665; doi:10.1038/s41586-026-11095-1)
Supplement: Supplementary file 1 [file 41586_2026_11095_MOESM1_ESM.pdf]

nature portfolio

https://doi.org/10.1038/s41586-026-11095-1

Supplementary information

Spatially deterministic nucleation of 2D semiconductors by etching flux

In the format provided by the authors and unedited

Supplementary Information

I. Supplementary Figures

Supplementary Fig. 1	Classification scheme for ASG of 2D TMD
Supplementary Fig. 2	Single nucleation behavior of 2D MoS_2 EF-SCN
Supplementary Fig. 3	Relationship between MoS_2 epitaxial growth direction and HfO_2 barrier pattern direction
Supplementary Fig. 4	2D MoS_2 growth behaviors with a-HfO_2, a-SiO_2, and a-Al_2O_3 barrier
Supplementary Fig. 5	2D MoS_2 etching behavior by HfO_2 surface
Supplementary Fig. 6	Oxygen flux and nucleation rate in EF-SCN system
Supplementary Fig. 7	High density single-crystal growth by EF-SCN
Supplementary Fig. 8	2D MoS_2 EF-SCN on amorphous substrate
Supplementary Fig. 9	Impact of oxygen released from an etching-flux barrier on underlying substrate
Supplementary Fig. 10	Valid DES flow window for single-crystal MoS_2 growth
Supplementary Fig. 11	Threshold chalcogen flow for nucleation in EF-SCN system
Supplementary Fig. 12	2D MoS_2 growth behaviors with chalcogen and metal flows
Supplementary Fig. 13	Nucleation design rule for single-crystal MoS_2 growth
Supplementary Fig. 14	MoS_2 growth behaviors in large-size pattern
Supplementary Fig. 15	Parametric study of nucleation outcomes around the reference EF-SCN condition
Supplementary Fig. 16	NAR/NSR analysis of the oxygen-flux origin of single-centered nucleation

II. Supplementary Tables

Supplementary Table 1	Thermal decomposition and crystallization temperature of various substrates
Supplementary Table 2	MoS_2 growth conditions with oxygen flow
Supplementary Table 3	2D MoS_2 growth and etching reactions
Supplementary Table 4	Benchmark table of 2D monolayer MoS_2 ASG

III. Supplementary Videos

Supplementary Video 1	Time evolution of the size-dependent nucleation regimes in the kMC-based hybrid diffusion-reaction process emulation
Supplementary Video 2	Time evolution of centered nucleation and shape-programmed growth in the kMC-based hybrid diffusion-reaction process emulation

IV. Supplementary Notes

Supplementary Note 1	Rigorous verifications of single-crystallinity for EF-SCN MoS_2
Supplementary Note 2	Oxygen-sensitive conditions for valid EF-SCN
Supplementary Note 3	EF-SCN nucleation rate modeling with experimental results
Supplementary Note 4	Oxygen depletion in the etching-flux barrier
Supplementary Note 5	Additional discussions of L_{growth} and growth conditions
Supplementary Note 6	Strategies for effective single-crystal growth
Supplementary Note 7	Hybrid diffusion-reaction process emulation of EF-SCN
Supplementary Note 8	Large-scale device uniformity of EF-SCN method
Supplementary Note 9	Benchmark of 2D monolayer MoS_2 ASG

V. References

I. Supplementary Figures

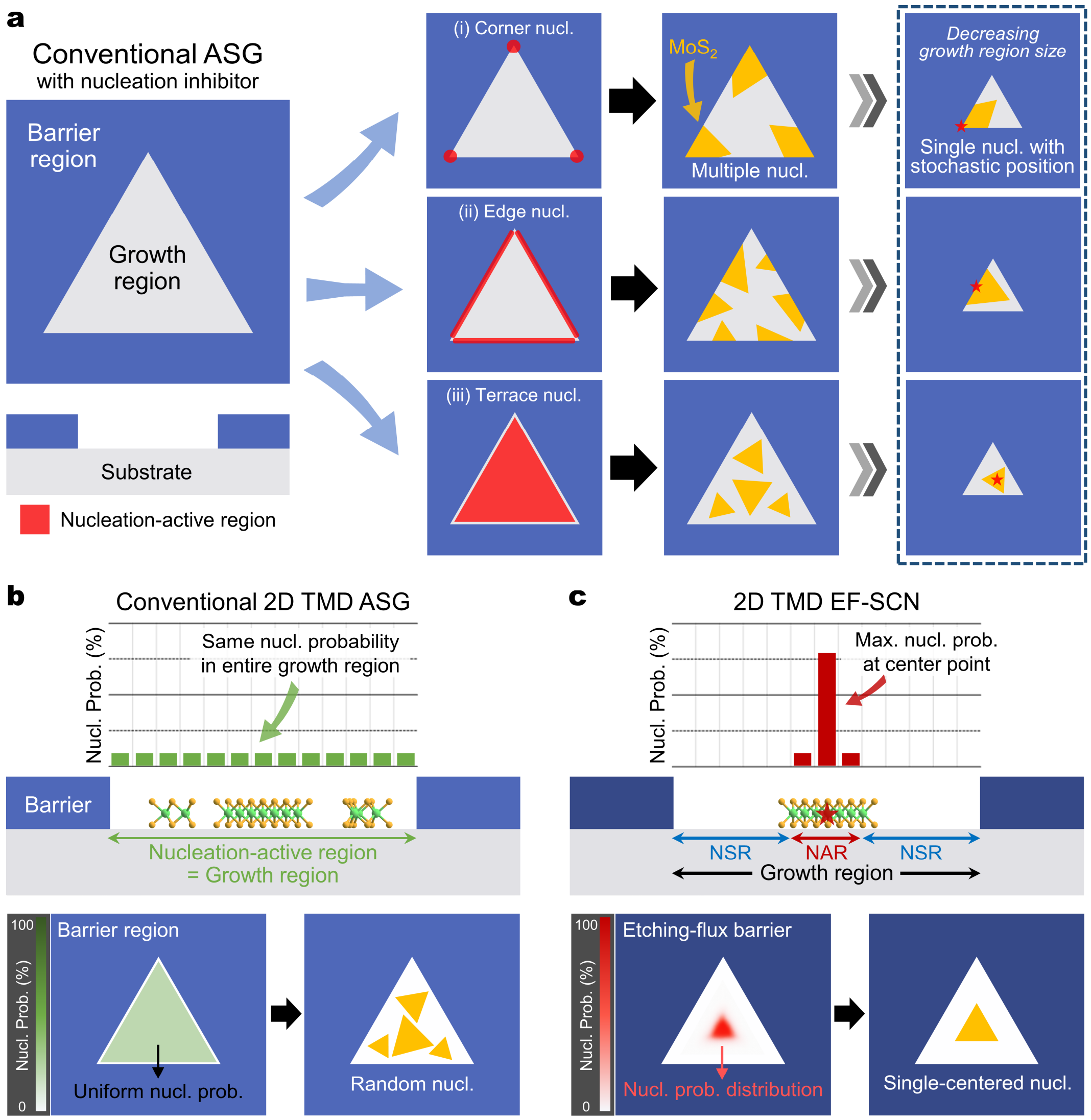

Supplementary Fig. 1 | Classification scheme for ASG of 2D TMD. a, Schematic of conventional 2D TMD area-selective growth (ASG) methods with nucleation barrier. The red area is nucleation-active region and MoS_2 nucleation can occur at the corner, edge, and terrace sites in the patterned substrate. **b**,**c**, Nucleation probability distribution of conventional 2D TMD ASG (**b**) and EF-SCN (**c**). The nucleation-active region (NAR) forms at the center of the growth region, whereas the nucleation-suppressed region (NSR) forms near the etching-flux barrier edge.

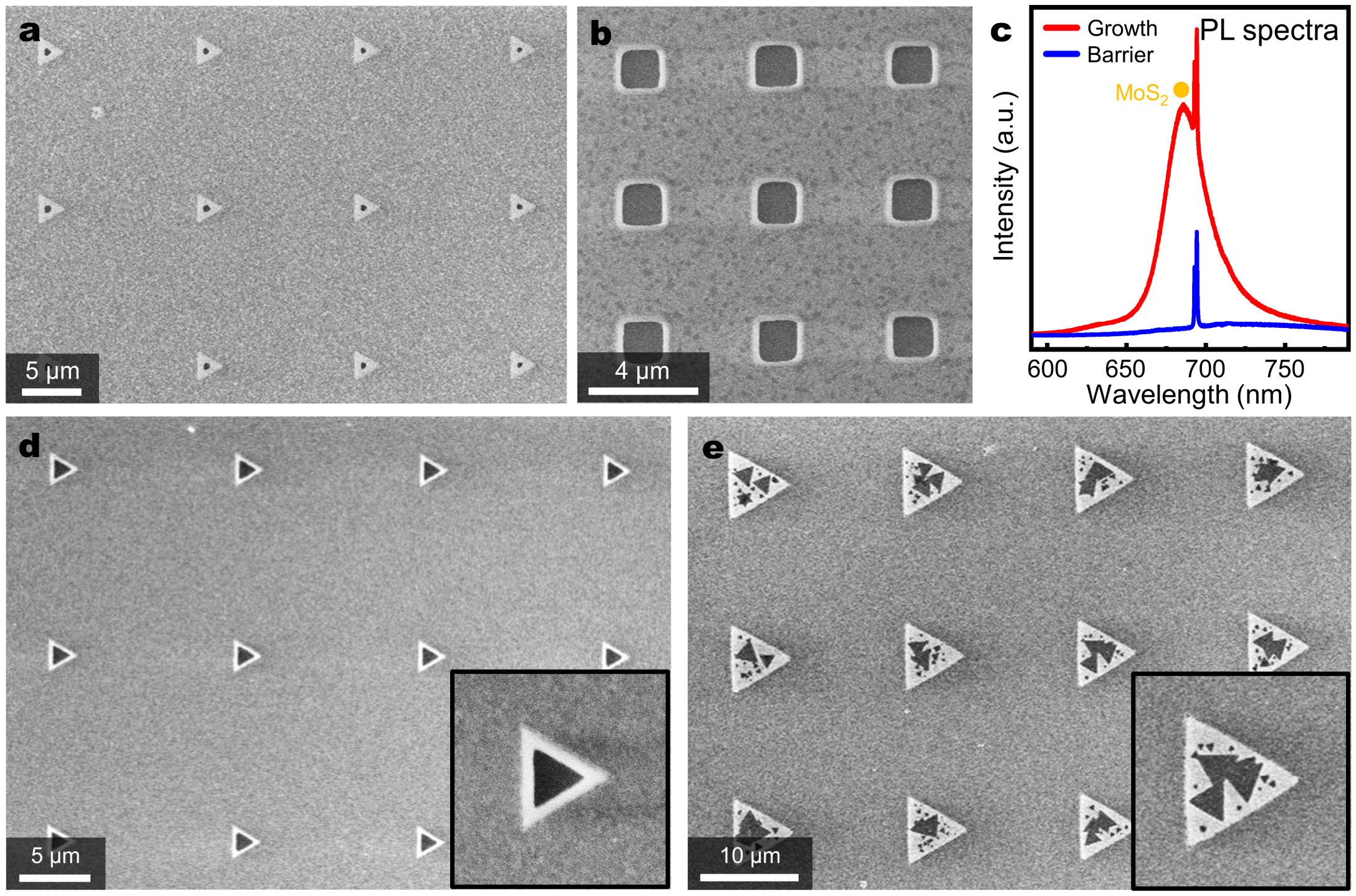

Supplementary Fig. 2 | Single nucleation behavior of 2D MoS_2 EF-SCN. a, SEM image of the initial nucleation stage at center point. **b**, SEM image of MoS_2 EF-SCN in square barrier patterns. **c**, Photoluminescence (PL) spectra of MoS_2 grown by EF-SCN. **d**,**e**, Representative SEM images of single-crystal MoS_2 flake (**d**) and poly-crystal MoS_2 flakes (**e**) by EF-SCN method.

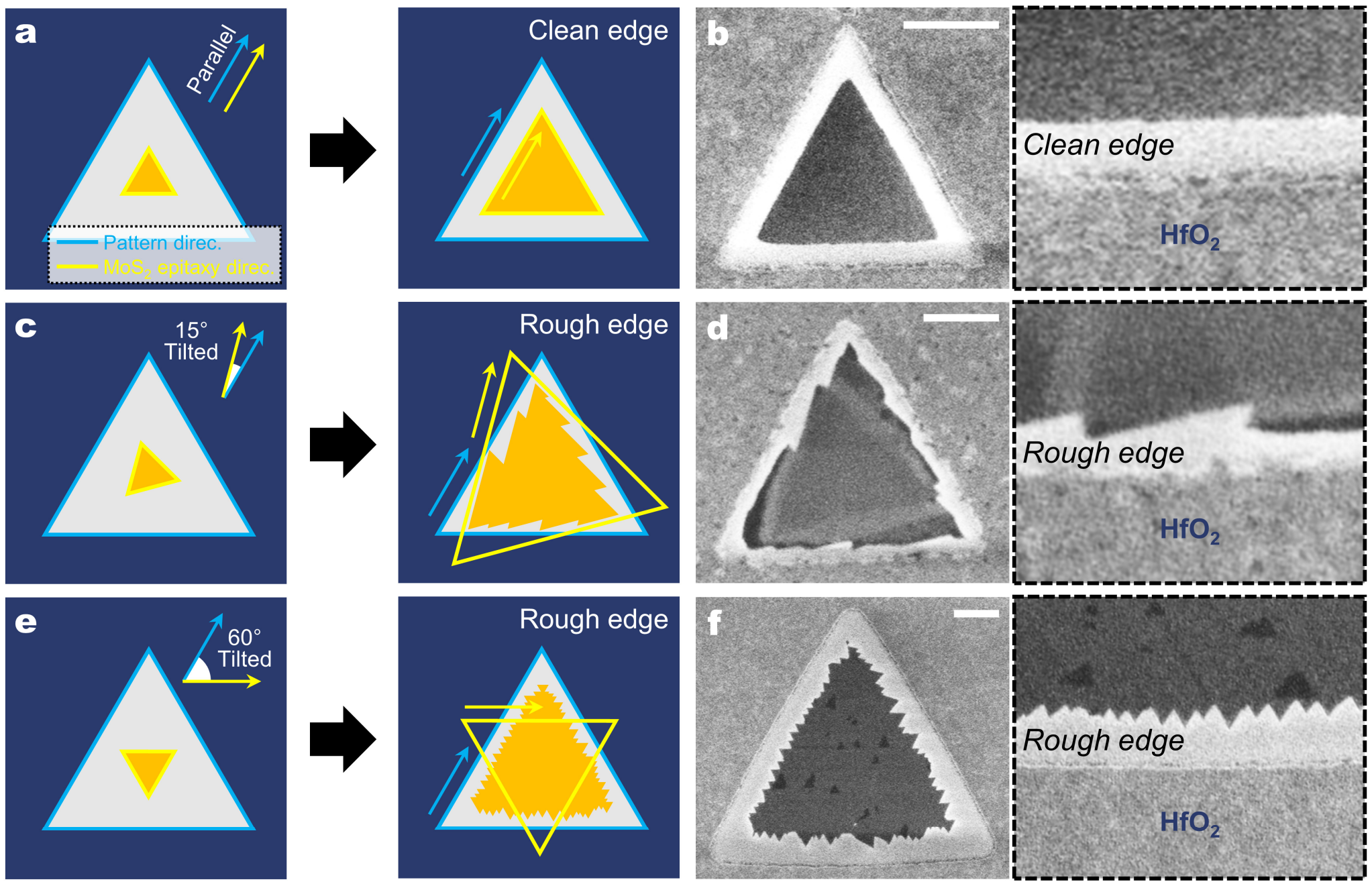

Supplementary Fig. 3 | Relationship between MoS_2 epitaxial growth direction and HfO_2 barrier pattern direction. a,b, Schematic (**a**) and SEM images (**b**) of MoS_2 growth when the epitaxial growth direction and the barrier pattern direction are parallel. **c–f**, Schematic and SEM images of MoS_2 growth at misalignment angles of 15° (**c, d**) and 60° (**e, f**) between the epitaxial growth direction and the barrier pattern direction. Scale bars, 1 μm.

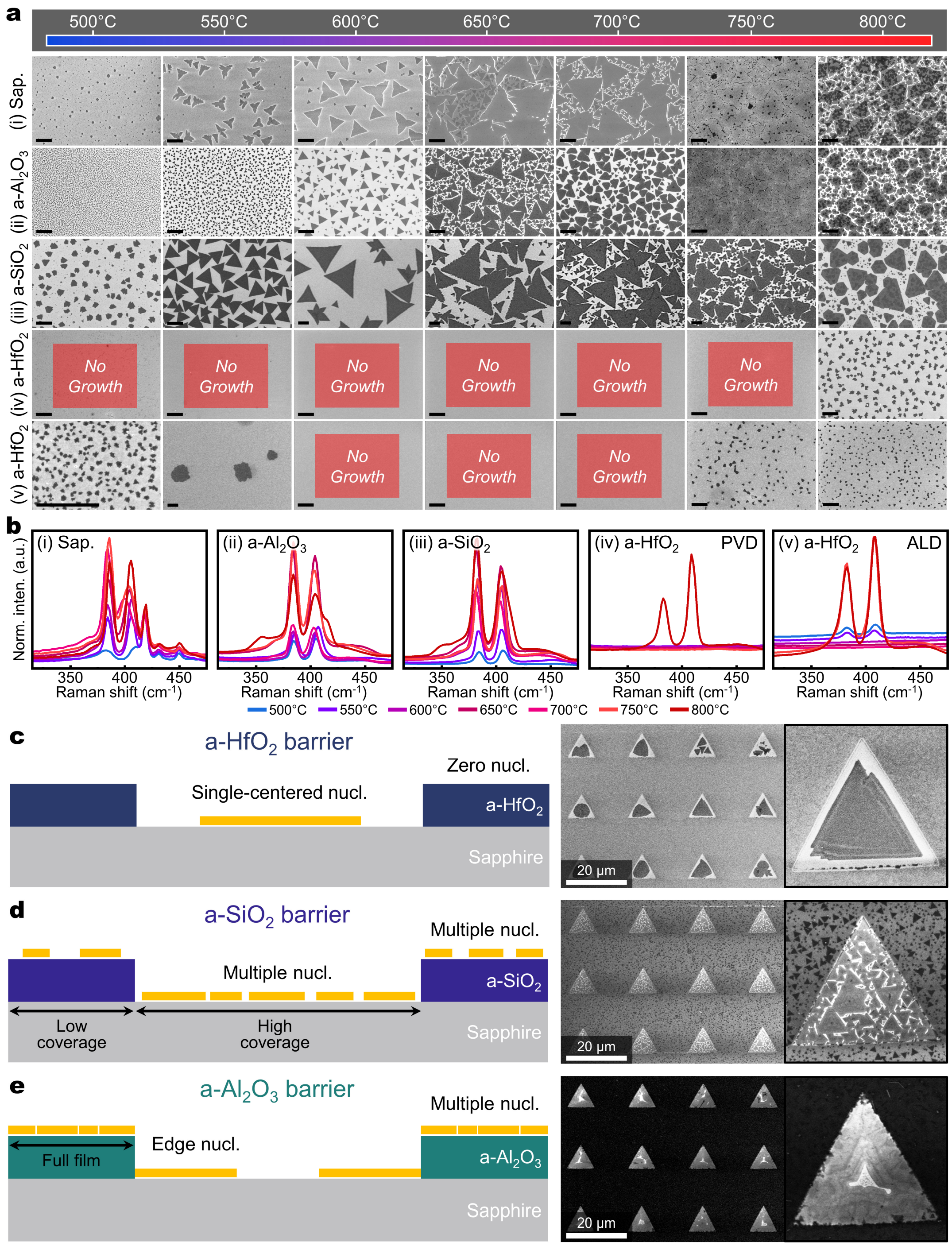

Supplementary Fig. 4 | 2D MoS_2 growth behaviors with a-HfO_2, a-SiO_2, and a-Al_2O_3 barrier. a,**b**, SEM images (**a**) and Raman spectra (**b**) of MoS_2 grown on various substrates (sapphire, a-Al_2O_3, a-SiO_2, PVD a-HfO_2, and ALD a-HfO_2) with MOCVD temperatures (500–800 °C). Scale bars, 3 μm. **c**–**e**, Schematic and SEM images of MoS_2-$MoSe_2$ lateral superlattice growth with a-HfO_2 barrier (**c**), a-SiO_2 barrier (**d**), and a-Al_2O_3 barrier (**e**).

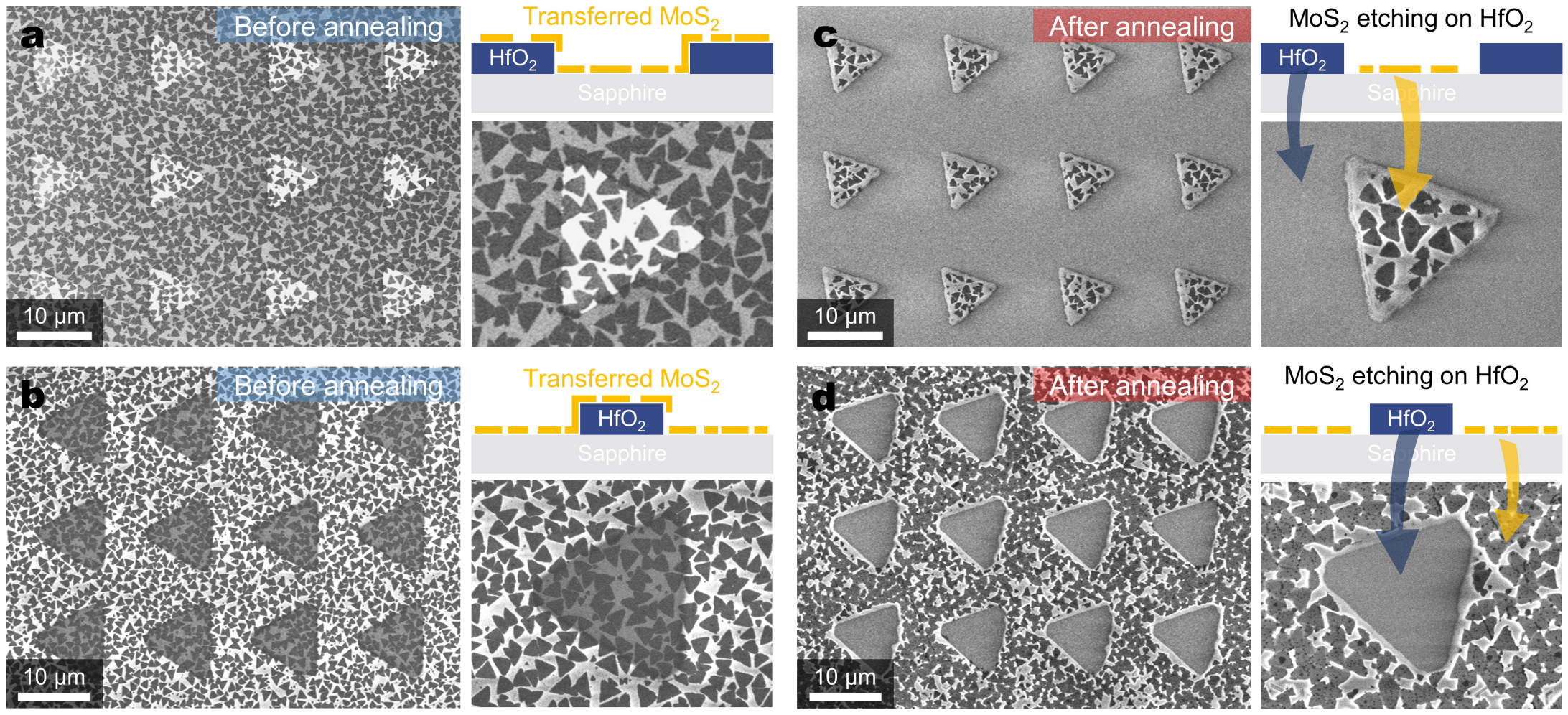

Supplementary Fig. 5 | 2D MoS_2 etching behavior by HfO_2 surface. a,**b**, SEM images of dry-transferred MoS_2 flakes on HfO_2 patterned substrate without annealing. **c**,**d**, SEM images of the transferred MoS_2 flakes after thermal annealing.

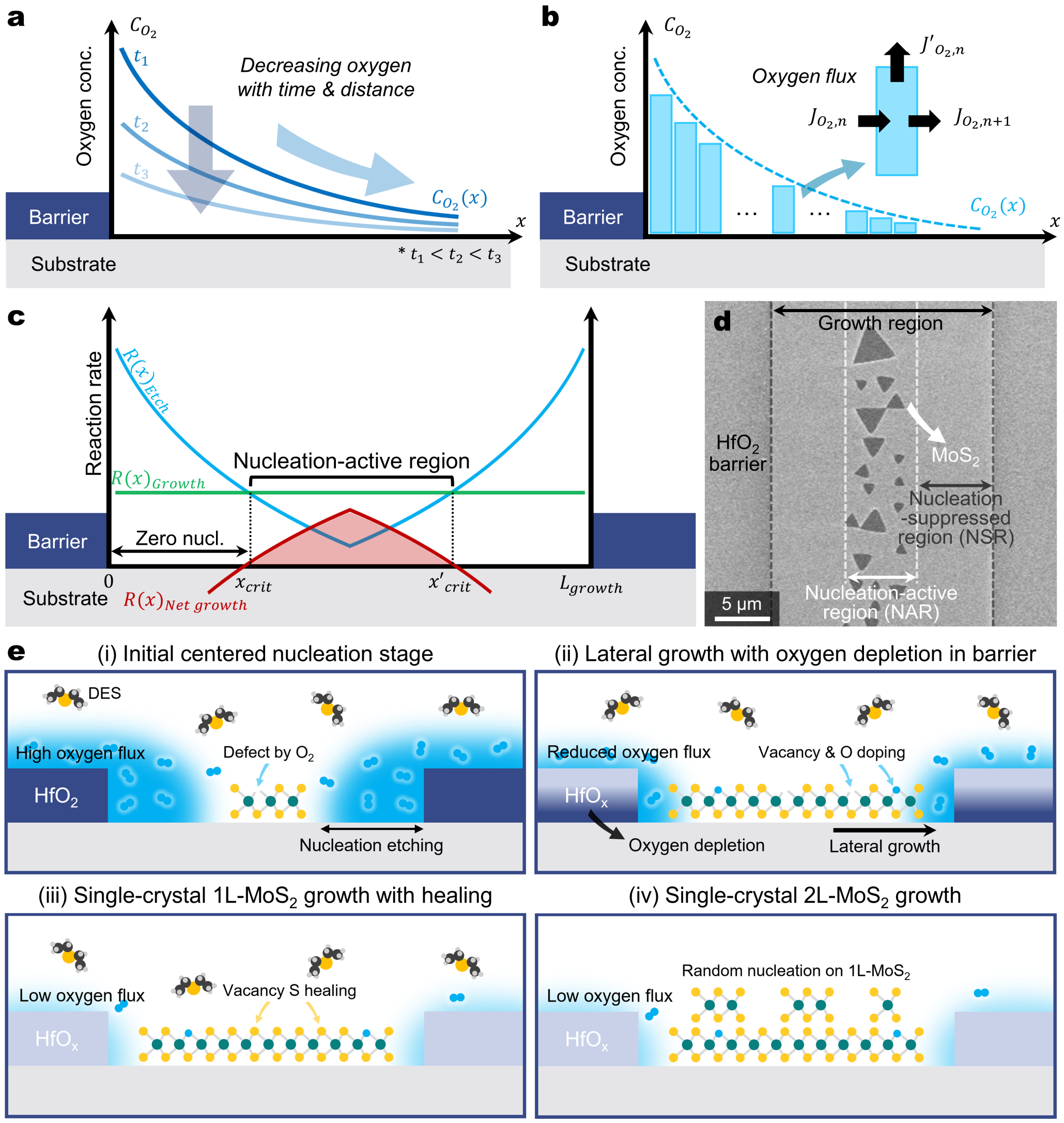

Supplementary Fig. 6 | Oxygen flux and nucleation rate in EF-SCN system. a,**b**, Schematic of oxygen concentration with time (**a**) and oxygen flux with distance (**b**) in the growth region. **c**, Schematic of nucleation rate distribution, nucleation-active region, and critical length in the EF-SCN system. **d**, SEM image of MoS_2 nucleation behavior in linear barrier pattern. **e**, Process schematic depicting centered nucleation, oxygen depletion in barrier, defect formation/healing, and vertical growth.

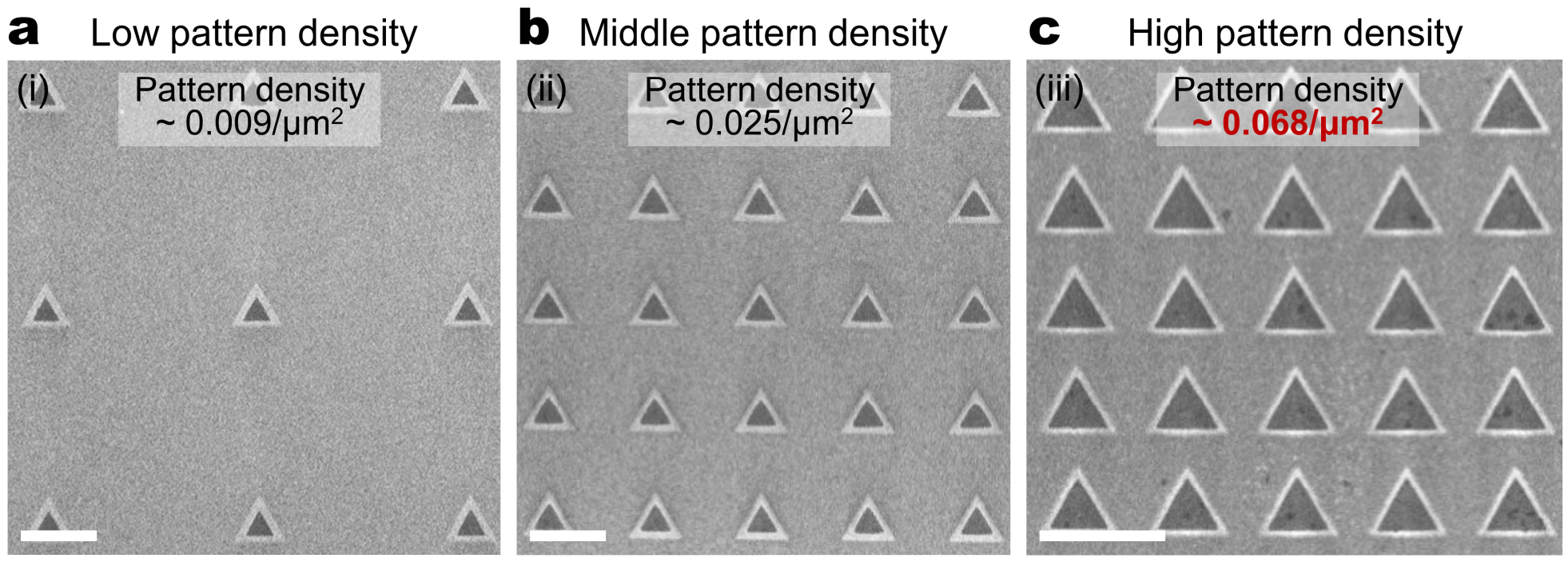

Supplementary Fig. 7 | High density single-crystal growth by EF-SCN. a–**c**, SEM images of MoS_2 EF-SCN behaviors in low (**a**), middle (**b**), and high (**c**) pattern density substrates. Scale bars, 5 μm.

a

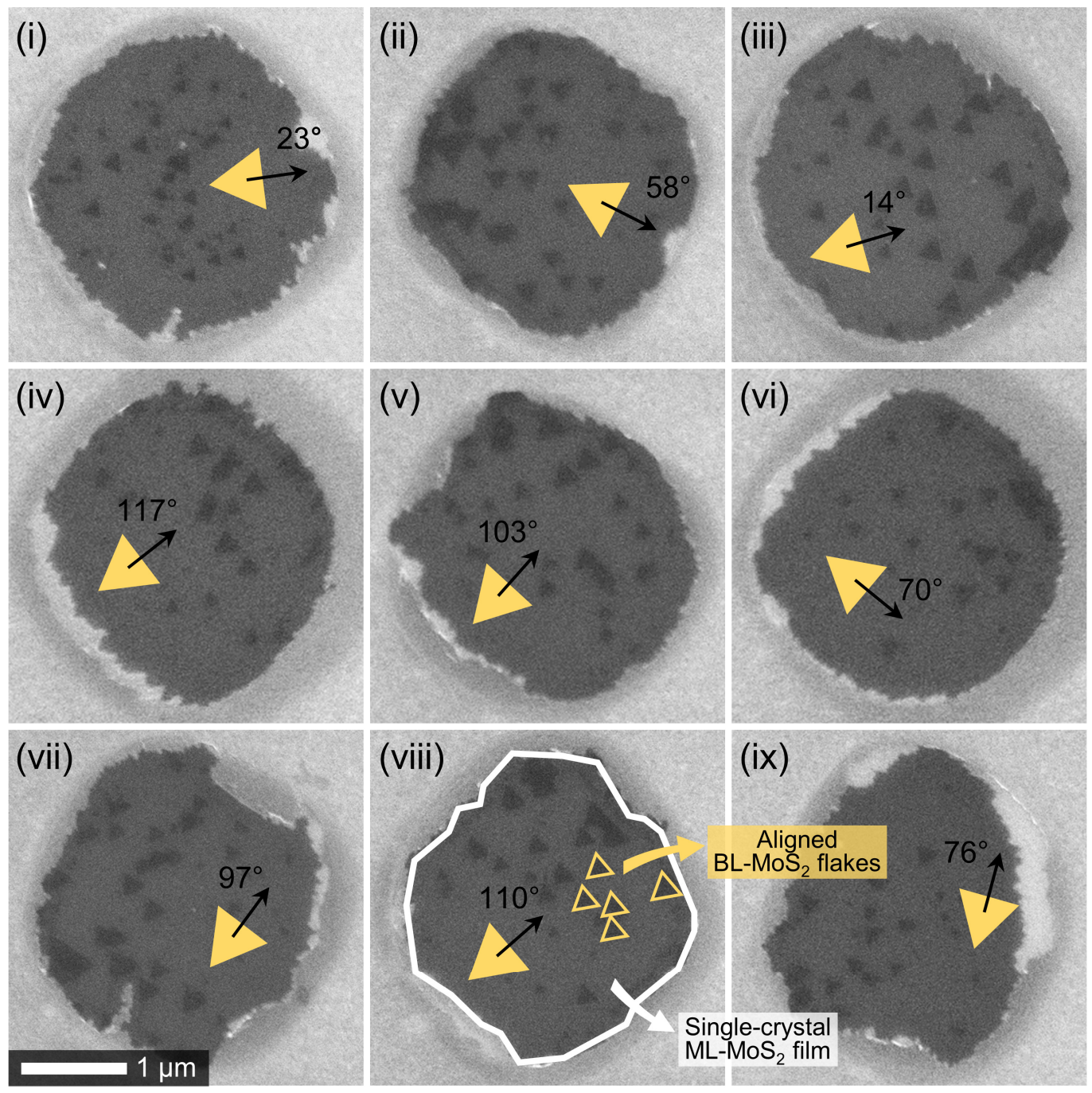

Supplementary Fig. 8 | 2D MoS_2 EF-SCN on amorphous substrate. a, SEM images of 2D MoS_2 EF-SCN on amorphous SiO_2 substrate, corresponding to the enlarged views of **Fig. 2d** (i-ix). Small bilayer MoS_2 flakes (dark contrast) are observed on the monolayer MoS_2 surface, exhibiting a consistent crystallographic orientation within each pattern site.

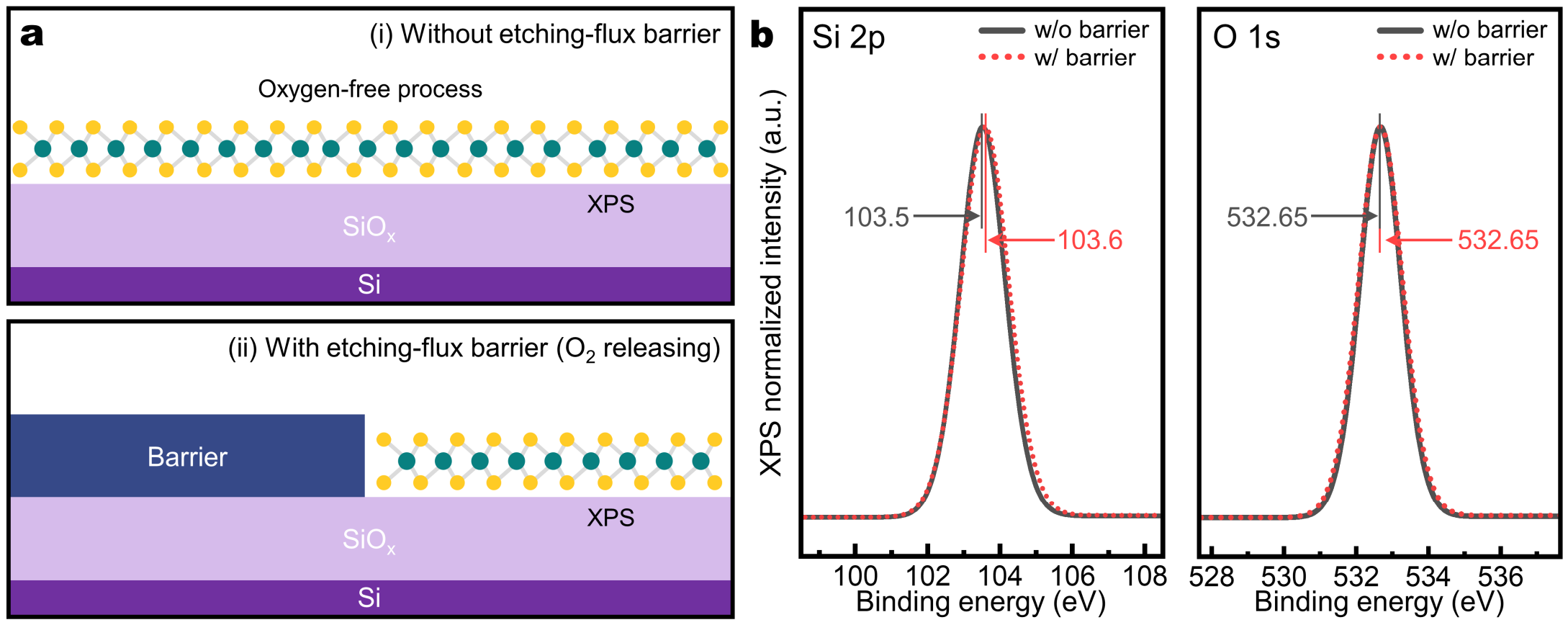

Supplementary Fig. 9 | Impact of oxygen released from an etching-flux barrier on underlying substrate. a,b, Schematic (**a**) and XPS analysis (**b**) comparing the quality of underlying dielectric layer (amorphous SiO_x) between conventional MoS_2 MOCVD without barrier and EF-SCN with barrier.

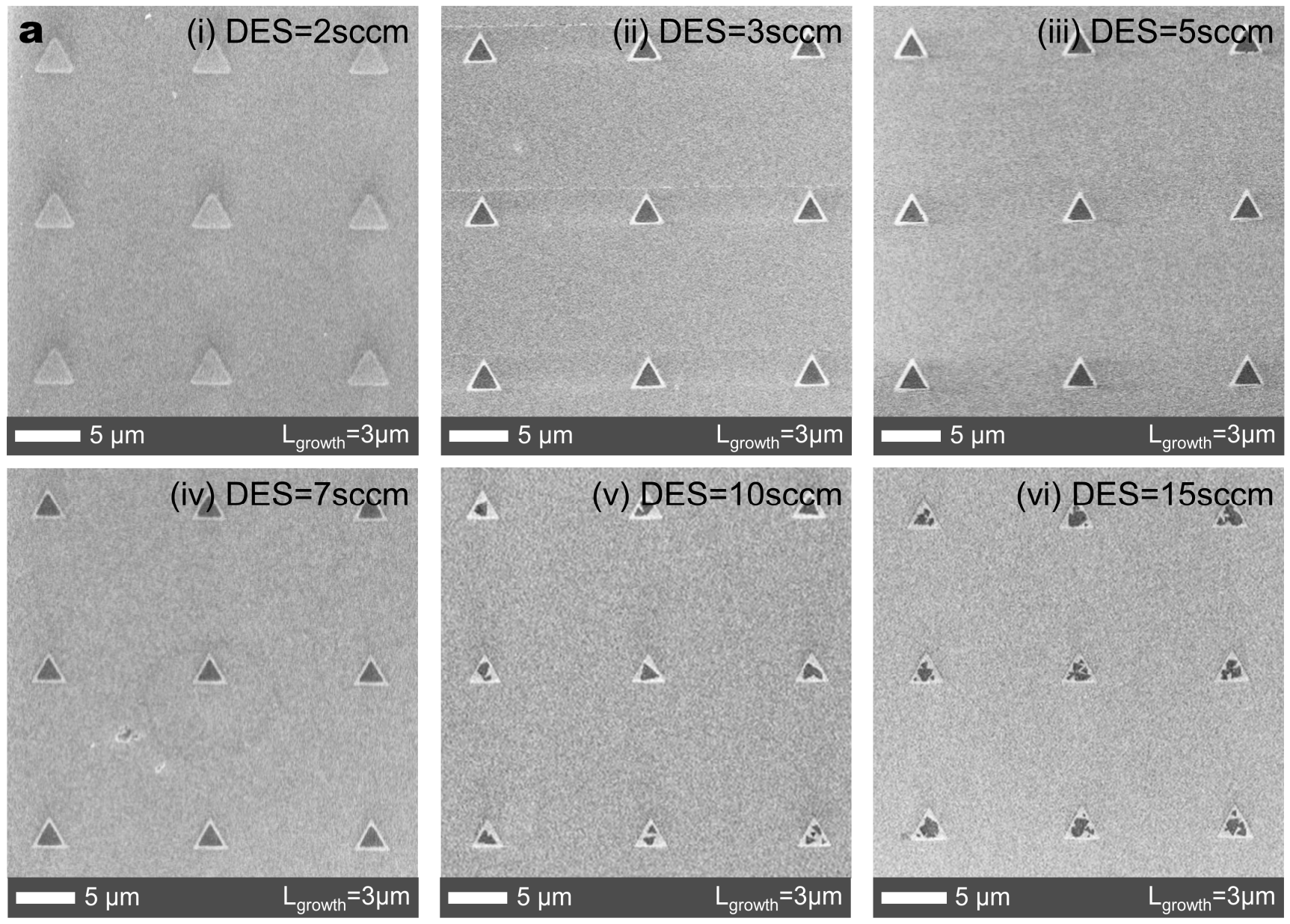

Supplementary Fig. 10 | Valid DES flow window for single-crystal MoS_2 growth. a, SEM images showing MoS_2 EF-SCN behavior with DES flow (2–15 sccm).

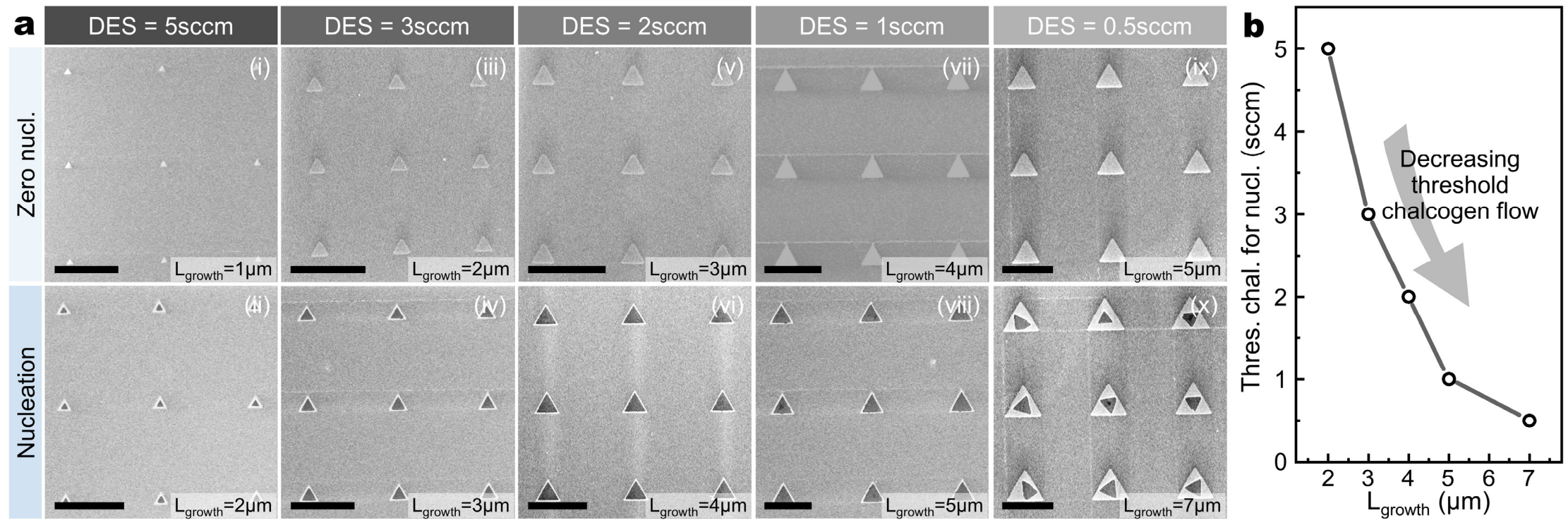

Supplementary Fig. 11 | Threshold chalcogen flow for nucleation in EF-SCN system. a, SEM images illustrating the onset of MoS_2 nucleation at different DES flow rates. Scale bars, 10 μm. **b**, Plot of threshold DES flow in EF-SCN process for valid MoS_2 nucleation.

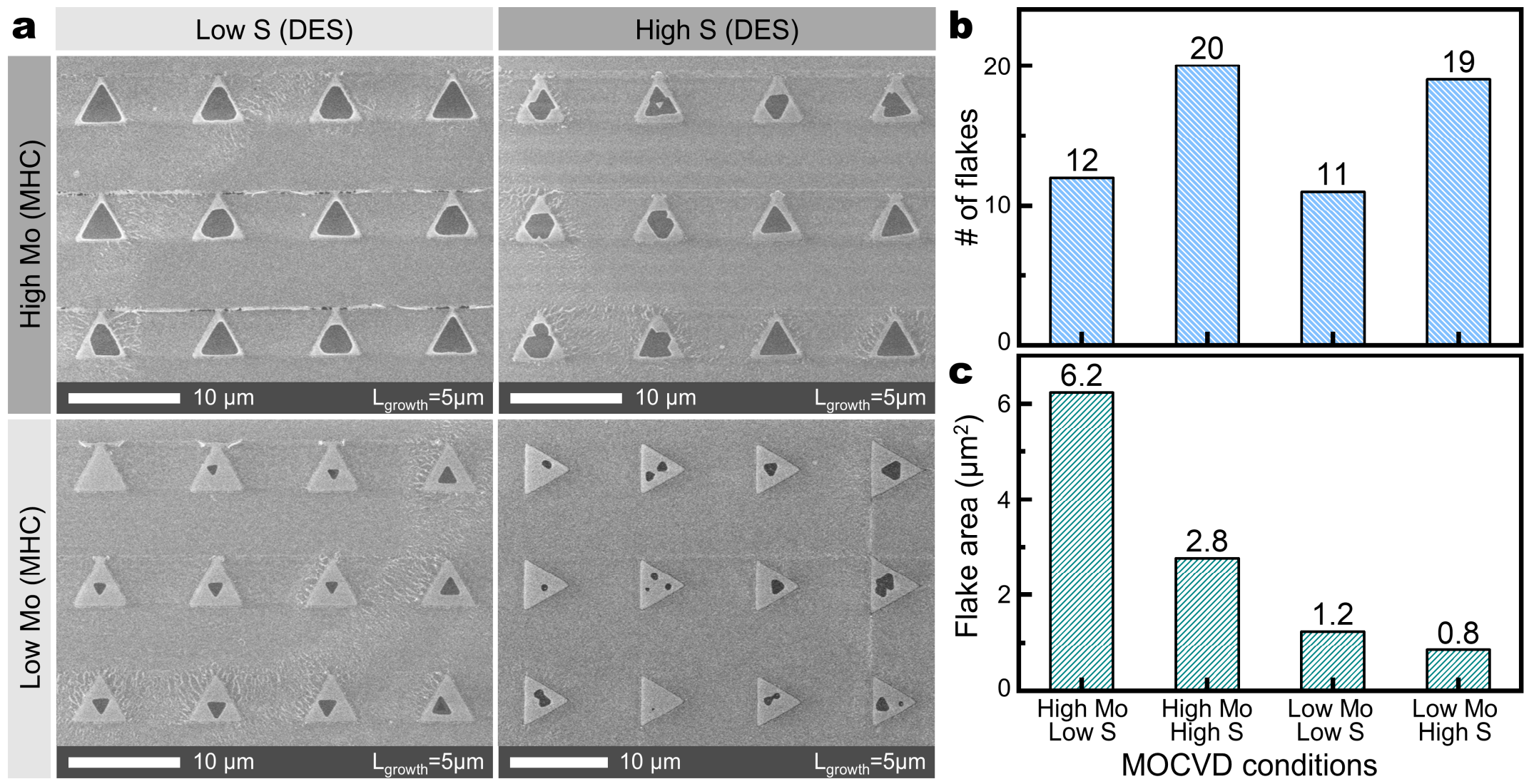

Supplementary Fig. 12 | 2D MoS_2 growth behaviors with chalcogen and metal flows. a, SEM images showing MoS_2 nucleation behavior under low/high sulfur (DES) and low/high Mo (MHC) conditions. **b**,**c**, Bar graphs of the number of flakes (**b**) and average flake area (**c**) with MOCVD conditions.

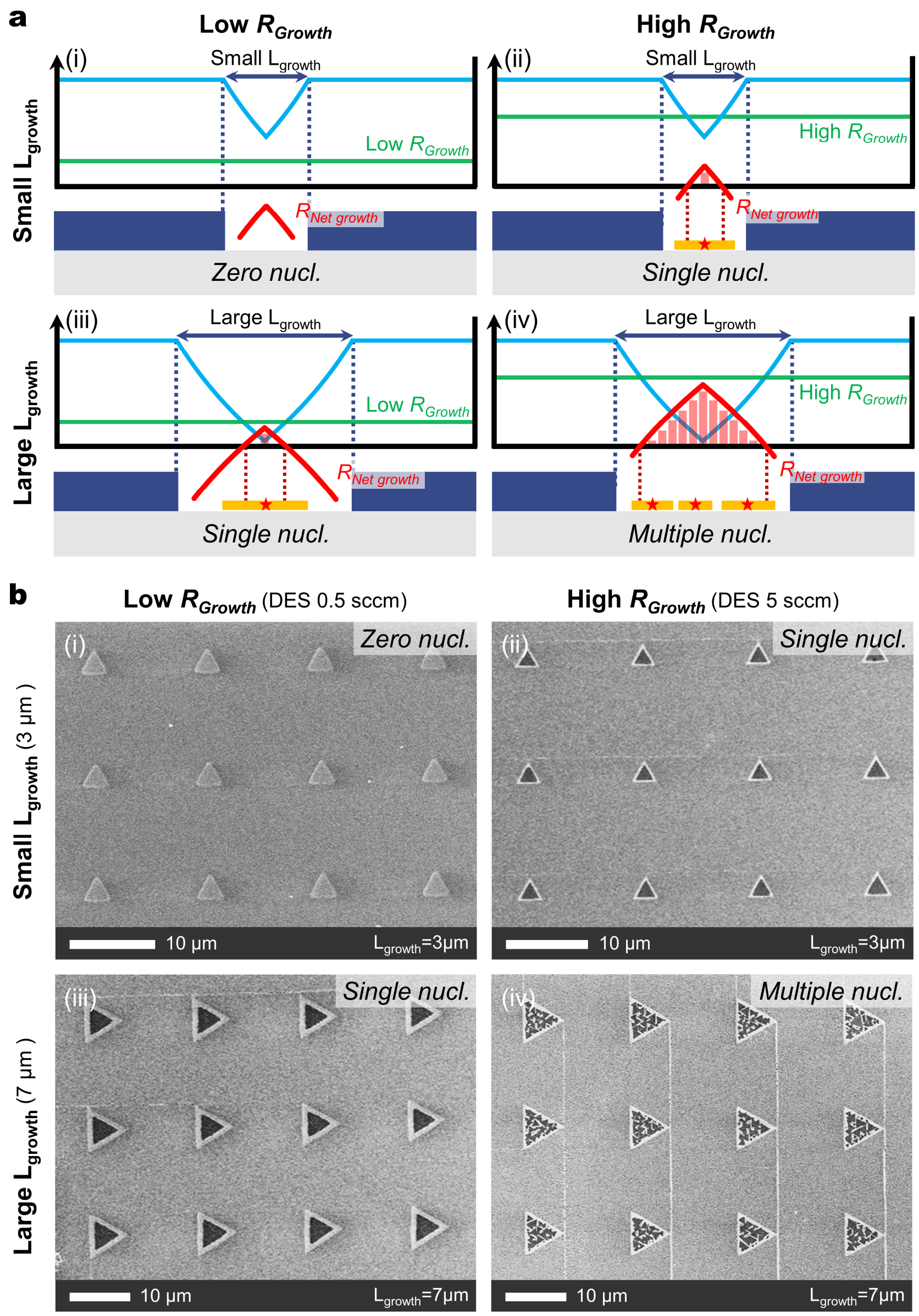

Supplementary Fig. 13 | Nucleation design rule for single-crystal MoS_2 growth. a,**b**, Schematic (**a**) and SEM images (**b**) for zero-single-multiple nucleation control by growth region size and MOCVD condition.

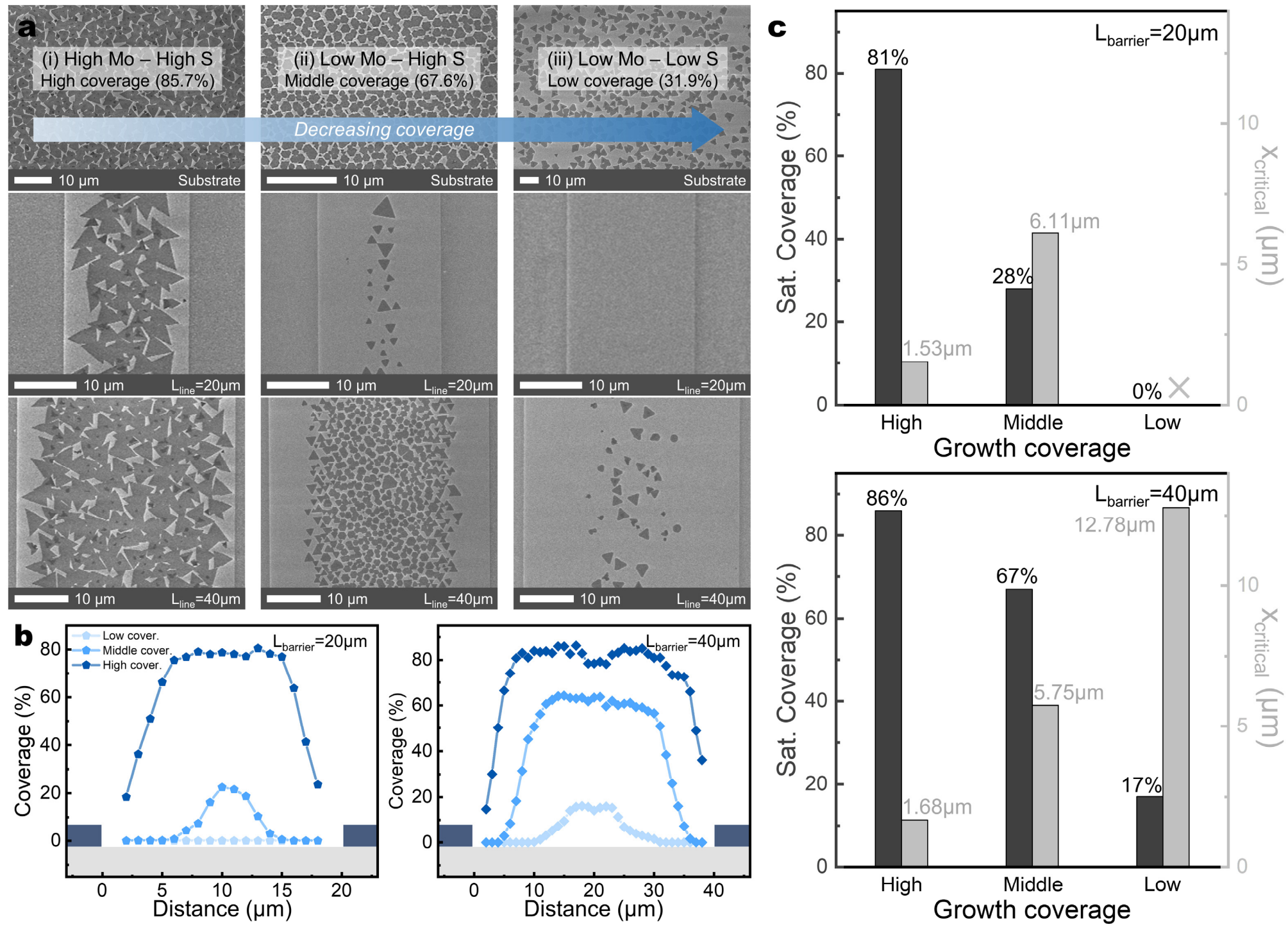

Supplementary Fig. 14 | MoS_2 growth behaviors in large-size pattern. a,b, SEM images (**a**) and coverage graphs (**b**) of MoS_2 growth in linear etching-flux barrier pattern with different growth conditions. **c**, Bar graphs of saturated growth coverage (dark gray) and critical length (light gray) of the results in (**a**).

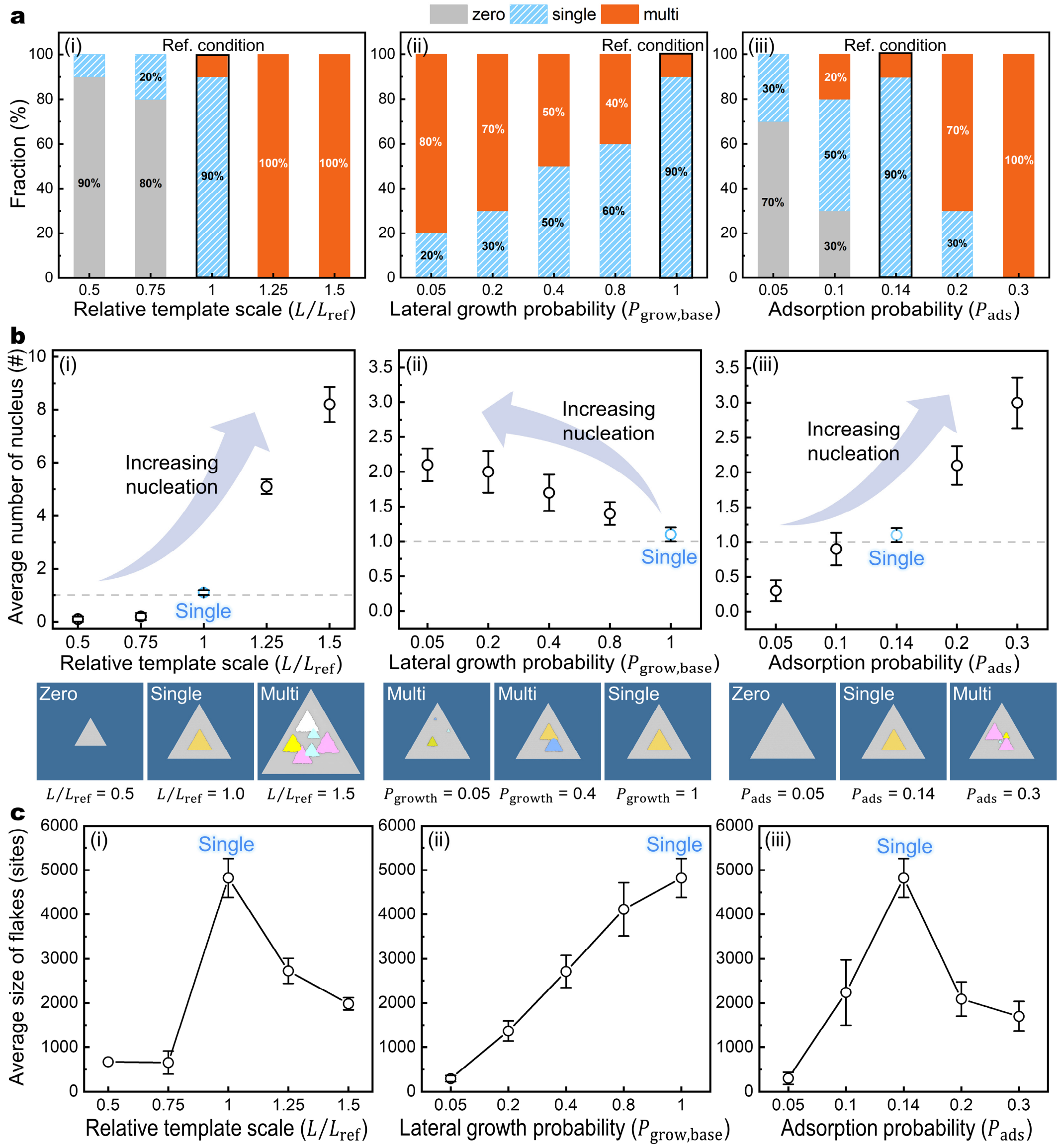

Supplementary Fig. 15 | Parametric study of nucleation outcomes around the reference EF-SCN condition. a, Fractions of zero, single, and multiple nucleation under three sweeps of relative template scale (i), maximum lateral growth probability (ii), and adsorption probability (iii). **b**,**c**, Average number of nuclei (**b**) and average flake size (**c**) for the same three sweeps.

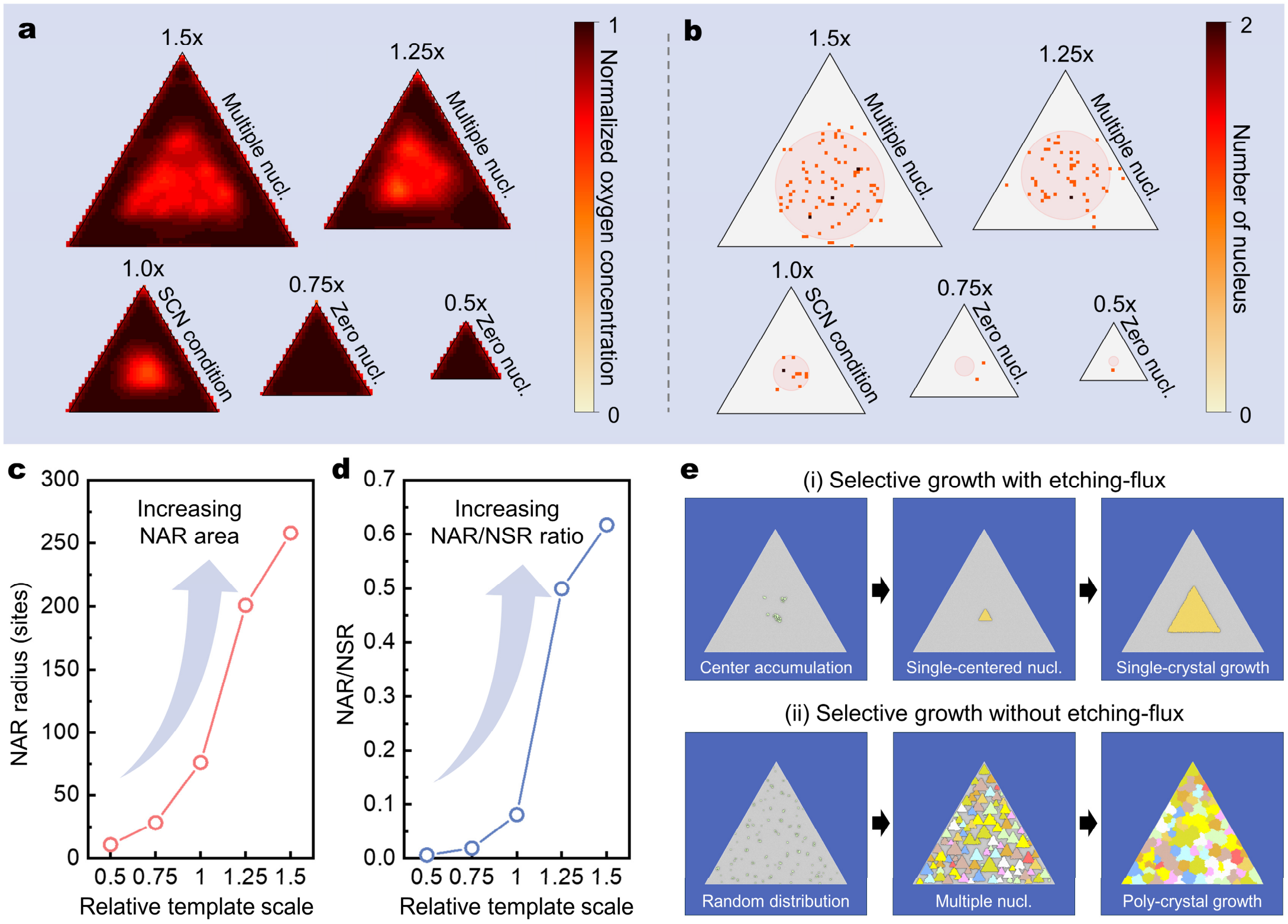

Supplementary Fig. 16 | NAR/NSR analysis of the oxygen-flux origin of single-centered nucleation. a–d, Normalized cumulative oxygen concentration maps across relative template scales (**a**), pooled nucleus positions with the effective NAR boundary (**b**), effective NAR radius (r_{eff}) as a function of relative template scale (**c**), NAR/NSR ratio as a function of relative template scale (**d**). **e**, Reference EF-SCN condition with and without the boundary oxygen etching-flux under otherwise identical rules.

II. Supplementary Tables

Material	Crystallinity	Annealing	Transition		Ref
Sapphire	Crystalline	1100–1500 °C (N_2 & N_2+H_2 vacuum)	Decomposition (>1100 °C)	>1100 °C	[1]
	Crystalline	1100–1500 °C (He & H_2 vacuum)	Decomposition (>1300 °C)		[2], [3]
a-Al_2O_3	Amorphous (Sputter)	600–1200 °C (Air)	Crystallization (~900 °C)	~900 °C	[4]
	Amorphous (ALD)	1000–1200 °C (N_2 vacuum)	Decomposition (~1150 °C)		[5]
	Amorphous (ALD)	800–1000 °C (Vacuum)	Decomposition (~900 °C)		[6]
a-SiO_2	Amorphous (Thermal)	-	Decomposition (>800 °C)	>800 °C	[7]
	Amorphous (Thermal)	1050–1100 °C (Vacuum)	Void formation (~1050 °C)		[8]
	Amorphous (Thermal)	760–850 °C (Vacuum)	Decomposition (>800 °C)		[9]
a-HfO_2	Amorphous (Plasma ALD)	400–600 °C (N_2 vacuum)	Decomposition (~500 °C)	~500 °C	[10]
	Amorphous (Sputter)	200–600 °C (Vacuum)	Roughening (~600 °C)		[11]

Supplementary Table 1 | Thermal decomposition and crystallization temperature of various substrates. Transition temperatures of sapphire, a-Al_2O_3, a-SiO_2, and a-HfO_2 during thermal annealing[1-11].

Type	Precursor	Temperature	Ar flow	O_2 flow	$O_2/Ar+O_2$	Ref
Powder CVD	**MoO_3**, S	910 °C	275 sccm	~8 sccm	**2.83%**	[12]
	**MoO_3**, S	750–850 °C	100 sccm	~2 sccm	**1.96%**	[13]
	**MoO_3**, S	625 °C	20 sccm	~0.2 sccm	**0.99%**	[14]
MOCVD	**MHC**, DES	600 °C	400 sccm	~0.1 sccm	**0.025%**	[15]

Supplementary Table 2 | MoS_2 growth conditions with oxygen flow. Precursors, growth temperature, and oxygen fraction conditions for effective MoS_2 growth[12-15].

	MoS_2 growth reactions (MoS₂ in product)	MoS_2 etching reactions (MoS₂ in reactant)
No O_2 in reactant	$Mo(CO)_6 + 2(C_2H_5)_2S \rightarrow MoS_2 + 6CO + 4C_2H_4 + 2H_2$	$MoS_2 + SO_2 \rightarrow MoO_2 + S$
	$Mo(CO)_6 + 3(C_2H_5)_2S + 2KCl \rightarrow MoS_2 + 6CO + 6C_2H_4 + K_2S + 2HCl + 2H_2$	$MoS_2 + 2H_2 \rightarrow Mo + 2H_2S$
	$K_2MoO_4 + 3(C_2H_5)_2S \rightarrow MoS_2 + K_2O + 3H_2O + 6C_2H_4 + S$	$MoS_2 + 2CO \rightarrow Mo + 2COS$
	$MoO_3 + 3(C_2H_5)_2S \rightarrow MoS_2 + 3H_2O + 6C_2H_4 + S$	$MoS_2 + CH_4 \rightarrow Mo + CS_2 + 2H_2$
O_2 in reactant	$Mo(CO)_6 + 2(C_2H_5)_2S + 3O_2 \rightarrow MoS_2 + 6CO_2 + 2C_2H_4 + H_2$	$2MoS_2 + 7O_2 \rightarrow 2MoO_3 + 4SO_2$
	$K_2MoO_4 + 3(C_2H_5)_2S + O_2 \rightarrow MoS_2 + K_2O + 3H_2O + 6C_2H_4 + SO_2$	$MoS_2 + 3O_2 \rightarrow MoO_3 + 2SO_2$
	$K_2MoO_4 + 2S + O_2 \rightarrow MoS_2 + K_2SO_4$	$MoS_2 + O_2 \rightarrow MoO_2 + SO_2$
	$MoO_3 + 3(C_2H_5)_2S + 19O_2 \rightarrow MoS_2 + SO_2 + 12CO_2 + 15H_2O$	$MoS_2 + 4KCl + 5O_2 \rightarrow MoO_2 + 2K_2SO_4 + 2Cl_2$

Supplementary Table 3 | 2D MoS_2 growth and etching reactions. Representative reactions for MoS_2 growth and etching with MOCVD system.

<table>
<tr><th colspan="2"></th><th>Seed growth [16-19]</th><th colspan="2">Confined growth [20,21]</th><th colspan="2">EF-SCN [Our work]</th></tr>
<tr><td colspan="2">Selectivity origin</td><td>Surface difference
(Nucleation promoter)</td><td colspan="2">Surface difference
(Nucleation inhibitor)</td><td colspan="2">Etching-flux</td></tr>
<tr><td rowspan="3">A</td><td>TMD growth temperature (°C)</td><td>650–750</td><td>750 [20]</td><td>385 [21]</td><td>750</td><td>430</td></tr>
<tr><td>TMD growth method</td><td>Powder CVD
or Sulfurization</td><td colspan="2">Powder CVD</td><td colspan="2">MOCVD</td></tr>
<tr><td>Mobility ($cm^2 V^{-1} s^{-1}$)</td><td>5–50</td><td>62 [20]</td><td>56 [21]</td><td colspan="2">117</td></tr>
<tr><td rowspan="5">B</td><td>Nucleation initiation</td><td>Not spatially deterministic</td><td colspan="2">Not spatially deterministic</td><td colspan="2">Deterministic at center</td></tr>
<tr><td>Effective single-crystal size (μm)</td><td>Limited single domain</td><td>1–2 [20]</td><td>0.2–0.5 [21]</td><td colspan="2">10</td></tr>
<tr><td>Orientation alignment</td><td>Not controlled</td><td colspan="2">Not controlled</td><td colspan="2">Possible</td></tr>
<tr><td rowspan="2">Advanced applications
beyond basic FET</td><td rowspan="2">-</td><td colspan="2">Valleytronics device [20]
with vertical heterostructure</td><td colspan="2">Self-aligned ALD contact FETs
with aligned lateral heterostructure</td></tr>
<tr><td colspan="2">CFET [21]
with single-crystal 3D integration</td><td colspan="2">Single-crystal multiple FETs
with large-size single-crystal</td></tr>
</table>

Supplementary Table 4 | Benchmark table of 2D monolayer MoS_2 ASG. Comparison between seed growth[16-19], confined growth[20,21], and EF-SCN method.

III. Supplementary Videos

Supplementary Video 1 | Time evolution of the size-dependent nucleation regimes in the kMC-based hybrid diffusion-reaction process emulation. **a**–**c**, Representative emulation results for the zero (**a**), single (**b**), and multiple-nucleation (**c**) regimes at relative template scales of 0.5×, 1.0×, and 1.5× respectively. Each frame advances one diffusion-reaction cycle (cycles 0–500); all panels share a common physical scale and a synchronized cycle clock.

Supplementary Video 2 | Time evolution of centered nucleation and shape-programmed growth in the kMC-based hybrid diffusion-reaction process emulation. **a**,**b**, Centered nucleation and subsequent lateral growth in triangular (**a**) and square (**b**) growth regions. The two regions were size-matched according to their inscribed-circle dimensions. **c**, Line-shaped growth through nucleation-growth decoupling in a template comprising a large nucleation zone and a narrow only growth zone. The square nucleation zone has the same lateral dimensions as the square growth region in **b**. A nucleus formed near the center of the nucleation zone expands into the only growth zone, where nucleation is suppressed but lateral growth is allowed.

IV. Supplementary Notes

Supplementary Note 1. Rigorous verifications of single-crystallinity for EF-SCN MoS_2

We performed various characterizations to confirm whether the MoS_2 flakes grown by EF-SCN method were indeed single-crystalline. As shown in Supplementary Fig. 2d, e, the characteristics of single and multiple nucleation can be readily distinguished by SEM images. To further confirm that the triangular MoS_2 flake observed in Fig. 1 is a single crystal, more rigorous characterizations were performed. Extended Data Fig. 1c–e present transmission electron microscopy (TEM) analyses of a MoS_2 flake grown by the EF-SCN method. The diffraction pattern obtained from a MoS_2 flake suspended over a TEM grid hole (Extended Data Fig. 1c) exhibits a typical hexagonal spot pattern of single-crystal MoS_2, rather than multiple spots or ring patterns (Extended Data Fig. 1d). Furthermore, as shown in Extended Data Fig. 1e, high-angle annular dark-field scanning transmission electron microscopy (HAADF-STEM) images acquired at the center (S1), edge (S2), and corner (S3) positions of the flake reveal consistent crystallographic orientation across all regions, aligned with the diffraction pattern. These results confirm that the entire flake region has a consistent crystallographic orientation, demonstrating its single-crystal nature. In addition, Extended Data Fig. 1f, g present atomic resolution AFM measurements, where a well-defined periodic hexagonal lattice characteristic of MoS_2 is clearly observed across the entire scanned area. AFM and corresponding fast Fourier transform (FFT) analyses performed at three arbitrary locations within a flake confirm identical crystallographic orientation. Together, these TEM and atomic resolution AFM results verify that the MoS_2 flakes grown by EF-SCN are single-crystalline and possess high structural quality.

Extended Data Fig. 2 shows second-harmonic generation (SHG) analyses of single-crystal MoS_2 grown by EF-SCN. In the case of single nucleation, a single triangular flake is observed in the SEM image, and the absence of grain boundaries results in uniform SHG intensity mapping, along with a well-defined six-petal pattern in angle-resolved SHG, characteristic of single-crystal MoS_2 (Extended Data Fig. 2a–c). In contrast, under multiple nucleation conditions, multiple domains and irregular flake morphologies are observed in the SEM image, accompanied by a significant intensity drop at grain boundaries in SHG mapping. The coexistence of domains with different orientations results in an isotropic SHG intensity distribution rather than a six-petal pattern (Extended Data Fig. 2d–f). The barrier patterns in EF-SCN are aligned with the epitaxial growth direction of MoS_2 on miscut sapphire. Accordingly, SHG measurements at six arbitrary positions within the single-crystal MoS_2 array consistently exhibit six-petal patterns with similar crystallographic orientations (Extended Data Fig. 2g, h). The small angular variations ($\pm 3°$) are attributed to slight misalignment arising from epitaxial growth at a relatively low temperature (750 °C). Furthermore, when the barrier pattern direction is misaligned with the epitaxial growth direction, rough edges aligned with the intrinsic crystallographic orientation are observed near the barrier, instead of the clean triangular flake typical of EF-SCN (Supplementary Fig. 3). These results indicate that the orientation of triangular single-crystal MoS_2 in EF-SCN is determined by the epitaxial growth direction rather than the barrier pattern geometry.

Supplementary Note 2. Oxygen-sensitive conditions for valid EF-SCN

The HfO_2 barrier used in the EF-SCN system enables a new selective growth strategy by releasing an oxygen etching-flux, which induces deterministic nucleation at the center of the growth region. Owing to the low thermal decomposition temperature of HfO_2, we confirmed that sufficient oxygen release occurs at 750 °C (Fig. 2b, c). In contrast to other thermally stable oxides listed in Supplementary Table 1, such as sapphire, a-Al_2O_3, and a-SiO_2, MoS_2 nucleation is selectively suppressed on HfO_2, enabling control over the nucleation position within the growth region (Supplementary Fig. 4).

Supplementary Fig. 4a, b present SEM images and Raman spectra showing the temperature-dependent growth behavior of MoS_2 (500–800 °C) on five different substrates (c-plane sapphire, a-Al_2O_3, a-SiO_2, a-HfO_2 by PVD and ALD) under the same MOCVD conditions. For sapphire, a-Al_2O_3, and a-SiO_2 substrates, MoS_2 growth was observed at all temperatures, with slight variations in coverage. However, for a-HfO_2, MoS_2 nucleation was clearly suppressed at temperatures between 500 and 750 °C. Supplementary Fig. 4c–e present the growth behavior of 2D TMD at 750 °C when a-HfO_2, a-SiO_2, and a-Al_2O_3 are used as barrier materials. Unlike a-HfO_2, where centered nucleation is induced by the etching-flux released from the barrier, a-SiO_2 exhibits random multiple nucleation within the growth region, while a-Al_2O_3 shows distinct edge nucleation. This indicates that a-SiO_2 and a-Al_2O_3 do not release sufficient oxygen during 2D TMD growth to induce an etching effect, whereas a-HfO_2 can suppress MoS_2 growth through etching-flux and enable spatially deterministic nucleation.

Moreover, Supplementary Fig. 5 shows SEM images of MoS_2 flakes dry-transferred onto an HfO_2-patterned sapphire substrate and subsequently annealed. Before annealing, MoS_2 flakes were observed on both HfO_2 and sapphire regions (Supplementary Fig. 5a, b); however, after annealing at 750 °C, only the flakes on sapphire remained, while those on HfO_2 were completely etched away (Supplementary Fig. 5c, d). These results verify that the oxygen released from HfO_2 induces a sufficiently effective etching effect on MoS_2 nucleation and growth behavior during the MOCVD process in EF-SCN.

Since this nucleation-position control mechanism, driven by the oxygen released from the etching-flux barrier, represents a completely new approach that has not been previously reported, it is necessary to clarify how the EF-SCN system differs from previous studies and to identify the key factors required for effective EF-SCN. We therefore address (I) previously reported TMD growth on HfO_2 and how the EF-SCN system differs from these earlier approaches, (II) the origin of the effective etching-flux behavior specific to the MOCVD process, and (III) the key growth conditions required for a valid EF-SCN system.

I. TMD growth on HfO_2 in previous studies

In previously reported studies on 2D TMD confined growth, HfO_2 was used as the growth region and SiO_2 as the barrier region to achieve selective growth of TMDs[20]. In addition, effective growth of MoS_2 on ALD-based HfO_2 films has also been reported[22]. A common feature of these studies is the use of high growth temperatures (700–900 °C) and powder CVD processes employing oxide precursors. Under such high-temperature conditions, oxygen can be sufficiently released from HfO_2; however, the use of oxide precursors is expected to significantly diminish the etching and nucleation suppression effects induced by the oxygen released from HfO_2. In contrast, in selective growth approaches that induce edge nucleation at low temperatures below 400 °C, the growth temperature of TMD is lower than the crystallization temperature of HfO_2, and thus insufficient oxygen is expected to be released[21]. Therefore, in conventional 2D TMD confined growth using HfO_2 as the growth region, the oxygen released from HfO_2 does not provide a sufficiently strong etching effect, and selective

growth is instead governed by the difference in TMD binding energy between the two surfaces.

II. Origin of the effective etching-flux behavior of HfO_2 in the EF-SCN system

EF-SCN employs a gas-precursor-based MOCVD process using molybdenum hexacarbonyl (MHC) and diethyl sulfide (DES), rather than an oxide-precursor-based powder CVD process. As shown in Supplementary Table 2, in powder CVD using molybdenum oxide precursors, effective MoS_2 growth has been reported even when the oxygen concentration reaches as high as ~1% during the growth process[12-14]. In contrast, in MOCVD using MHC precursors, even a small oxygen concentration of 0.025% induces an etching effect, and increasing the oxygen concentration to ~0.04% results in complete etching, preventing MoS_2 growth[15]. Therefore, because the EF-SCN system is based on an oxygen-sensitive MOCVD process, deterministic control of the nucleation position is expected to be achievable by the trace amounts of oxygen released from HfO_2.

III. Growth conditions for achieving an effective EF-SCN system

To demonstrate that oxygen-sensitive growth conditions are critical for the EF-SCN system, Extended Data Fig. 3 analyzes the valid conditions under which HfO_2 can act as an etching-flux barrier. Extended Data Fig. 3a shows SEM images of MoS_2 MOCVD growth on HfO_2 films deposited by e-beam evaporation at growth temperatures of 600 and 700 °C. Consistent with the typical EF-SCN results, MoS_2 growth is completely suppressed on HfO_2. In contrast, when the DES flow is greatly increased from 5 sccm to 20 sccm (Extended Data Fig. 3b(i) and 3b(ii)) or the H_2 flow is increased from 0 sccm to 800 sccm (Extended Data Fig. 3b(iii) and 3b(iv)), MoS_2 growth is observed even on the HfO_2 surface. In addition, MoS_2 growth on the HfO_2 surface is clearly observed when MOCVD is performed after vacuum-annealing HfO_2 at 1000 °C for 1 h (Extended Data Fig. 3c(i) and 3c(ii)) or after H_2 annealing (Extended Data Fig. 3c(iii) and 3c(iv)). These results indicate that even if oxygen is released from the etching-flux barrier, HfO_2 can act as a growth surface when the relative oxygen level is insufficient to suppress MoS_2 nucleation.

Extended Data Fig. 3d, e present the MoS_2 growth behavior on the HfO_2 surface as a function of the in-situ pre-annealing time and HfO_2 thickness. The growth temperature and growth duration were fixed at 750 °C and 30 min, respectively. After ramping the temperature to 750 °C, an in-situ pre-annealing step was introduced prior to the precursor injection for MoS_2 growth, allowing the duration of the oxygen-mediated nucleation-suppression effect to be evaluated different HfO_2 thicknesses. As shown in the SEM images in Extended Data Fig. 3d, no MoS_2 nucleation was observed on all HfO_2 surfaces with thicknesses ranging from 5 to 50 nm when the pre-annealing time was between 0 and 1 h. In contrast, MoS_2 growth was observed on the 5 nm HfO_2 surface after 3 h of pre-annealing, and on the 5–20 nm HfO_2 surfaces after 6 h of pre-annealing. These results indicate that a longer pre-annealing time and a thinner HfO_2 thickness accelerate the depletion of oxygen in the HfO_2 barrier, thereby weakening its ability to suppress MoS_2 nucleation. Extended Data Fig. 3e shows the MoS_2 coverage extracted from the SEM images in Extended Data Fig. 3d. Considering that the EF-SCN process is typically completed within 1 h, HfO_2 barriers with a thickness of 5 nm or greater are expected to retain sufficient nucleation-suppression capacity throughout the whole EF-SCN process for single-crystal growth.

Extended Data Fig. 3f shows a schematic illustrating the valid growth conditions under which HfO_2 can act as an etching-flux barrier. As shown in Extended Data Fig. 3f(i), the EF-SCN system requires MOCVD conditions with low H_2 and low chalcogen flow, under which oxygen released from HfO_2 can be used for nucleation control. In contrast, when the H_2 flow or chalcogen flow is high, the relative effect of oxygen is reduced by the hydrogen atmosphere

(Extended Data Fig. 3f(ii)) or the high TMD nucleation rate (Extended Data Fig. 3f(iii)), resulting in reduced selectivity. As noted above, when molybdenum oxide-based precursors are used, the oxygen released from HfO_2 is expected to lose its TMD etching effect (Extended Data Fig. 3f(iv)). In addition, if the growth temperature is too high, oxygen can be rapidly depleted (Extended Data Fig. 3f(v)), whereas if the growth temperature is too low, insufficient oxygen is released (Extended Data Fig. 3f(vi)); in both cases, HfO_2 is expected to fail to act as an etching-flux barrier.

These valid conditions for EF-SCN can be further expanded by using other barrier materials instead of HfO_2. As shown in Extended Data Fig. 4, when a ZrO_2 barrier with a lower crystallization temperature than HfO_2 is used, EF-SCN can be achieved even below 500 °C[23-27]. In addition, as shown by PVD-HfO_2 and ALD-HfO_2 in Supplementary Fig. 4a, the nucleation suppression effect can vary depending on the deposition method of the etching-flux barrier. Taken together, these findings suggest that the EF-SCN strategy is broadly extendable: by tailoring the barrier material and its deposition conditions, diverse nucleation control scenarios can be realized beyond the HfO_2-based system demonstrated here.

Supplementary Note 3. EF-SCN nucleation rate modeling with experimental results

Etching-flux mediated single-centered nucleation (EF-SCN) is a growth method that employs an etching-flux barrier to achieve single nucleation of 2D transition metal dichalcogenide (TMD) at a deterministic site. Importantly, in the EF-SCN system, the nucleation-active region (NAR) is formed exclusively at the center of the growth region rather than across the entire surface, necessitating the development of a model to explain this unique behavior. In this note, we provide a theoretical explanation of the oxygen flux, oxygen concentration, and MoS_2 nucleation rate in the growth region.

I. Conceptual differences: nucleation vs growth

Before discussing the EF-SCN model in detail, we need to clarify the terms 'nucleation' and 'growth'. 'Nucleation' refers to the initial stage in material formation where atoms gather to form a stable nucleus. In 2D TMD area-selective growth (ASG) and EF-SCN, selectivity is defined based on this nucleation stage. 'Growth' refers to the subsequent stage in which additional atoms attach to the existing nucleus, leading to expansion of the individual crystal. Although 'growth' can broadly describe material formation, in this manuscript it is distinguished from 'nucleation'. The reaction rates discussed below (R_{Growth}, R_{Etch}, and $R_{Net\ growth}$) are nucleation rates: R_{Growth} denotes the reaction rate in the direction of nucleation formation, and R_{Etch} denotes the reaction rate in the direction of nucleation removal (i.e., the subscript 'growth' in R_{Growth} does not indicate the above growth stage, but rather the reaction direction). $R_{Net\ growth}$ is defined as $R_{Growth} - R_{Etch}$, which corresponds to the net nucleation rate.

II. Oxygen flux and concentration equations

We confirmed that the HfO_2 etching-flux barrier releases oxygen, which can etch and desorb 2D TMD nuclei. Therefore, in the EF-SCN model, the most important step is to express the oxygen concentration emitted from the etching-flux barrier as a function of distance from the barrier edge into the growth region. Supplementary Fig. 6b schematically represents the oxygen concentration (C_{O_2}) released from the barrier as a function of distance. To derive the equation for $C_{O_2}(x)$, we first consider the oxygen flux (J_{O_2}) in a unit area. The oxygen flux equation in the nth unit area, starting from the barrier edge, can be written as:

$$J_{O_2,n}(x) = J'_{O_2,n}(z) + J_{O_2,n+1}(x) \qquad (1)$$

in which $J_{O_2,n}(x)$ is the oxygen in-flux into the nth unit area along the x-direction, $J'_{O_2,n}(z)$ is the oxygen out-flux diffusing out of the nth unit area along the z-direction, and $J_{O_2,n+1}(x)$ is the oxygen out-flux to the next n+1th unit area along the x-direction. Here, $J_{O_2,n}(x)$ and $J_{O_2,n+1}(x)$ are the oxygen fluxes along the x-direction that govern TMD nucleation in the growth region, and $J'_{O_2,n}(z)$ is the oxygen flux along the z-direction, which escapes from the system and does not affect growth. To simplify the oxygen flux model, it is assumed that the oxygen out-flux is proportional to the oxygen in-flux (Assumption #1).

$$J'_{O_2,n}(z) = kJ_{O_2,n}(x) \qquad (2)$$

in which k is the proportionality constant representing the fraction of oxygen flux removed in the z-direction. By using equations (1) and equation (2), $J_{O_2}(x)$ can be expressed as:

$$\frac{dJ_{O_2}(x)}{dx} = -J'_{O_2}(z) = -kJ_{O_2}(x) \qquad (3)$$

Assuming that the oxygen flux decays to zero as $x \to \infty$, i.e., $\lim_{x\to\infty} J_{O_2}(x) = 0$ (Assumption #2), the general solution for the oxygen flux is given by:

$$J_{O_2}(x) = A\exp(-kx) + p = A\exp(-kx) \quad (4)$$

where A is the oxygen flux constant in the x-direction. According to Fick's first law, the oxygen concentration and flux are related by:

$$J_{O_2}(x) = -D\frac{dC_{O_2}(x)}{dx} \quad (5)$$

in which D is the oxygen diffusivity in the x-direction. From equations (4) and equation (5), and assuming that the oxygen concentration also approaches zero as $x \to \infty$, i.e., $\lim_{x\to\infty} C_{O_2}(x) = 0$ (Assumption #3), the oxygen concentration equation can be given as:

$$C_{O_2}(x) = \frac{A}{Dk}\exp(-kx) + q = \frac{A}{Dk}\exp(-kx) = C_0\exp(-kx) \quad (6)$$

in which C_0 is the initial oxygen concentration at $x = 0$. Equation (6) is valid under steady-state conditions, i.e., when the system is time-independent, whereas the actual oxygen concentration decreases over time, as shown in Fig. 2c and Supplementary Fig. 6a. This temporal decay of oxygen is expressed as follows:

$$C_{O_2}(x,t) = C_0\exp(-kx) \times f(t) \quad (7)$$

where $f(t)$ is time-dependent decay function of the oxygen concentration. The reduction of oxygen released from HfO_2 depends on the extent of crystallization and oxygen self-diffusion in the etching-flux barrier. Accordingly, $f(t)$ is determined by parameters such as the temperature and pressure of the 2D TMD growth process, as well as the deposition method and thickness of HfO_2.

III. 2D TMD nucleation rate in the growth region

The 2D MoS_2 EF-SCN is realized by metal-organic chemical vapor deposition (MOCVD), in which numerous reactions occur simultaneously. Since it is challenging to consider all reaction pathways in the model, we first categorize the growth and etching reactions for MoS_2 nucleation. Supplementary Table 3 provides 16 representative reaction equations that can arise from 2D TMD CVD precursors such as $Mo(CO)_6$, $(C_2H_5)_2S$, KCl, O_2, and H_2. The growth reactions of MoS_2 nucleation (where MoS_2 is the product) can be divided into those without O_2 as a reactant and those with O_2 as a reactant. While $Mo(CO)_6$ is the main molybdenum precursor, at high temperatures above 700 °C, intermediate species such as K_2MoO_4 or MoO_x can also participate in the MoS_2 growth. Similarly, the etching reactions of MoS_2 nucleation (where MoS_2 is the reactant) can be divided into those without O_2 as a reactant and those with O_2 as a reactant. Among these reactions, we focus on the O_2-involved MoS_2 etching reactions, since oxygen released from the barrier plays a crucial role in the EF-SCN system; thus, these reaction rates are considered first in the analysis.

The rate of MoS_2 etching reactions involving O_2 can be expressed as:

$$R(x)_{Etch} = B[O_2]^m = B \times \frac{A^m}{(Dk)^m} exp(-mkx) = B \times {C_0}^m \times exp(-mkx) \quad (8)$$

$$R(x,t)_{Etch} = B \times {C_0}^m \times exp(-mkx) \times f(t)^m \quad (9)$$

in which B is the rate constant for O_2-involved MoS_2 etching reactions, and m is the reaction order with respect to oxygen. MoS_2 etching reactions not involving O_2 as a reactant are either independent of oxygen released from the etching-flux barrier (ex. etching by H_2, CH_4, etc.) or, if oxygen-containing species participate (ex. etching by SO_2, CO, etc.), can be subsumed into equation (8). Therefore, the nucleation etching rate induced by the etching-flux barrier inside the growth region can be summarized as equation (8).

On the other hand, the MoS_2 growth reaction rates must consider the specific features of 2D MoS_2 MOCVD. MoS_2 growth reactions not involving O_2 as a reactant are unaffected by oxygen released from the barrier and remain uniform across the growth region. However, the rate of MoS_2 growth reactions involving O_2 as a reactant may increase with oxygen concentration. Nevertheless, as shown in Supplementary Fig. 6d, oxygen in the MOCVD process

predominantly induces etching rather than promoting MoS_2 nucleation, and the extent to which the growth reaction rate is affected by oxygen is expected to be negligible compared with the variation in the etching reaction rate. Therefore, as a simplification, we assume that oxygen released from HfO_2 does not affect the MoS_2 growth reactions in the MOCVD process (Assumption #4). However, this assumption may not hold under certain conditions, such as excessively high or low CVD temperatures, variations in etching-flux barrier properties, and the type of CVD precursors. In our EF-SCN system, the growth reaction rate can be simply expressed as a constant throughout the growth region.

$$R(x)_{Growth} = G_i \ (constant) \quad (10)$$

Finally, using equations (8)-(10), the net nucleation rate is given by:

$$R(x)_{Net\ growth} = R(x)_{Growth} - R(x)_{Etch} = G_i - B \times {C_0}^m \times exp(-mkx) \quad (11)$$

$$R(x,t)_{Net\ growth} = G_i - B \times {C_0}^m \times exp(-mkx) \times f(t)^m \quad (12)$$

IV. Important values in the 2D TMD EF-SCN system

From equations (8)-(12), we can plot the reaction rates of nucleation, as shown in Supplementary Fig. 6c. The central area where the net nucleation rate, $R(x)_{Net\ growth}$, becomes greater than zero is defined as the nucleation-active region (NAR), while the region near the etching-flux barrier where $R(x)_{Net\ growth}$ becomes zero is defined as the nucleation-suppressed region (NSR). In the EF-SCN system, where the nucleation site is deterministically controlled, there are two important parameters for designing nucleation behavior. The first is the critical length, x_{crit}, which represents the distance from the barrier edge at which $R(x)_{Net\ growth}$ becomes zero. This can be expressed as:

$$x_{crit} = \frac{1}{mk} \ln\left(\frac{BC_0^m f(t)^m}{G_i}\right) \quad (13)$$

x_{crit} is independent of the growth region size (L_{growth}) but increases when $G_i(R(x)_{Growth})$ decreases. As shown in Extended Data Fig. 6d, x_{crit} remains constant regardless of L_{growth}. In contrast, Supplementary Fig. 14 demonstrates that x_{crit} is larger under low growth coverage conditions.

The second is the width of the NAR ($x_{crit} < x < x'_{crit}$), defined as the region where nucleation can occur. This is given by:

$$W_{NAR} = L_{growth} - \frac{2}{mk} \ln\left(\frac{BC_0^m f(t)^m}{G_i}\right) \quad (14)$$

W_{NAR} is governed by the three parameters described in Fig. 3. First, over time, $f(t)$ decreases, leading to a broadening of the NAR. Furthermore, increasing L_{growth} or G_i results in an increase in W_{NAR}. These predictions are consistent with experimental observations, including the temporal evolution of NAR formation and the onset of nucleation (Extended Data Fig. 5), the occurrence of multiple nucleation events with larger L_{growth} (Fig. 3b) and at higher diethyl sulfide (DES) flows (Fig. 3c).

Supplementary Note 4. Oxygen depletion in the etching-flux barrier

I. Finite oxygen release in the etching-flux barrier

The HfO_2 etching-flux barrier employed in this study is expected to release oxygen during its crystallization process, which is thermally activated under MoS_2 growth conditions. However, as crystallization progresses, the oxygen reservoir becomes gradually depleted, implying that the total oxygen supply is intrinsically finite. As evidenced by the XPS data in Fig. 2c, the oxygen content in HfO_2 decreases rapidly within the first 10 minutes at 750 °C and subsequently reaches a saturated state. This observation indicates that the oxygen flux is most pronounced during the initial centered nucleation stage and gradually diminishes thereafter. Consequently, the resulting single-crystal film is not expected to experience excessive or unintended etching effects.

Supplementary Fig. 8 provides experimental evidence for the time-dependent attenuation of the etching effect from the etching-flux barrier. As observed in the SEM images, once a single-crystal monolayer MoS_2 film has fully formed, subsequent bilayer nucleation no longer occurs at the central region but instead emerges at random locations on the monolayer surface. This clearly demonstrates that the oxygen flux released from HfO_2 becomes insufficient to enforce spatial selectivity of nucleation over time.

II. Potential impact of oxygen on the channel region

Extended Data Fig. 1e presents HAADF-STEM images of EF-SCN single-crystal MoS_2 transferred onto a TEM grid. Atomic-scale analysis reveals the presence of sulfur vacancy sites, manifested as reduced contrast at chalcogen positions. Importantly, the observed defect density is comparable to that reported for conventional CVD-grown MoS_2, suggesting that oxygen from the etching-flux barrier does not significantly degrade the MoS_2 film quality[28-30]. Moreover, excessive oxygen exposure could induce molybdenum vacancy sites; however, no molybdenum vacancies are observed in the HAADF-STEM analysis, indicating that the crystalline quality remains comparable to that of standard CVD-grown MoS_2.

III. Potential impact of oxygen on the underlying dielectric film

The amount of oxygen released from the etching-flux barrier in the EF-SCN system is expected to be extremely low compared to other studies in which oxygen is introduced into the gas phase for 2D TMD growth control. In fact, MoS_2 MOCVD research using oxygen gas reported that even an oxygen fraction of 0.04% can completely suppress MoS_2 growth[15]. In contrast, under the EF-SCN system, nucleation occurs at the center of the growth region, implying that the effective oxygen partial pressure is substantially lower than that reported in such studies. This suggests that the oxygen level is sufficiently low to avoid detrimental effects on the other regions.

The impact of this trace oxygen flux on the underlying dielectric film is expected to be minimal. In particular, for crystalline substrates such as sapphire, it is unlikely that such low-level oxygen exposure would induce measurable surface degradation, such as increased roughness. Therefore, we employed amorphous SiO_x, which is more susceptible to oxygen-induced changes than crystalline sapphire. As shown in Supplementary Fig. 9, MoS_2 was grown on amorphous SiO_x substrates using both oxygen-free MOCVD and EF-SCN with a ZrO_2 barrier. After growth, the MoS_2 films were removed via a transfer process, and XPS measurements were performed on the exposed SiO_x surface. For the MOCVD sample grown without the etching-flux barrier, we measured the SiO_x surface where MoS_2 had previously been present. In the case of the EF-SCN sample with the barrier, measurements were performed at a location 10 μm away from the barrier edge.

As shown in the Si 2p XPS spectra in Supplementary Fig. 9b, binding energy peaks are observed at 103.5eV without the barrier and at 103.6eV with the barrier. In addition, the O 1s XPS spectra exhibit an identical peak position at 532.65eV in both cases. These results indicate that the oxygen released from the etching-flux barrier does not have a detrimental effect on the underlying SiO_x surface. Furthermore, XPS analysis of the SiO_x film reveals atomic ratios of $SiO_{1.506}$ without the barrier and $SiO_{1.502}$ with the barrier, confirming that two films are compositionally equivalent.

IV. Time-dependent 2D TMD growth scenario in the EF-SCN system

Supplementary Fig. 6e presents a schematic that more clearly illustrates the growth behavior of EF-SCN. At the initial stage of MoS_2 growth, a high oxygen flux released from HfO_2 induces a strong nucleation-etching effect, driving selective nucleation at the center — the point farthest from the barrier (Supplementary Fig. 6e(i)).

As growth proceeds, the MoS_2 nucleated at the center fills the growth region through lateral growth, while oxygen depletion progresses in the HfO_2 barrier (Supplementary Fig. 6e(ii)). As a result, the oxygen flux gradually decreases, weakening its nucleation-suppression capability. During this stage, oxygen may induce processes such as sulfur vacancy formation or oxygen doping, which could influence the film quality.

With further growth time, only a trace amount of oxygen is released from HfO_x. At this stage, the oxygen flux remains sufficient to suppress MoS_2 nucleation on the barrier but is no longer strong enough to control nucleation positions within the growth region (Supplementary Fig. 6e(iii)). In addition, a continuous supply of MOCVD precursors facilitates partial healing of defect sites.

At the final stage, as the oxygen flux from HfO_x becomes insufficient to maintain spatial control, bilayer MoS_2 flakes nucleate at random locations on the monolayer MoS_2 surface (Supplementary Fig. 6e(iv)). Ultimately, after a sufficiently long duration, HfO_x may completely lose its ability to release oxygen, which could lead to undesired growth on the barrier. However, as shown in Extended Data Fig. 3c, MoS_2 growth on HfO_x requires extreme conditions such as thermal annealing at 1000 °C for 1 hour. Therefore, considering the typical EF-SCN growth duration and conditions, the selectivity window of the HfO_2 barrier is expected to be sufficiently wide.

Supplementary Note 5. Additional discussions of L_{growth} and growth conditions

Extended Data Fig. 6d displays the critical length (blue hatched bar) and the number of nuclei per pattern (red filled bar) as functions of L_{growth}. Even as L_{growth} increases, the critical length remains nearly constant, because the position where the net nucleation rate becomes zero does not change, as illustrated in Fig. 3a (i) and (iii). Conversely, as L_{growth} increases, the width of the NAR expands, resulting in a significant increase in the number of nuclei. Extended Data Fig. 6e shows the distribution of distances between the pattern center and the incenter of MoS_2 flakes across different growth region sizes. As the growth region size increases, both the mean distance from the pattern center and the spread of the distribution increase systematically. This result suggests that as L_{growth} increases, the NAR expands, leading to an increase in both the number and spatial spread of nucleation events. Moreover, the EF-SCN mechanism indicates that MOCVD conditions can alter the reaction rate and control nucleation behavior.

Supplementary Fig. 11 shows the threshold chalcogen flow required for MoS_2 nucleation as a function of L_{growth}. The graph reveals that a smaller L_{growth} requires a higher chalcogen flow for nucleation to occur, indicating that the DES flow critically controls R_{Growth} control. Supplementary Fig. 12 shows the nucleation and growth behavior under low and high DES flow conditions (3 and 5 sccm), and low and high MHC conditions (2.5 and 4 sccm). Under high DES conditions, the number of flakes is consistently about twice that observed under low DES conditions, suggesting that DES flow is the limiting factor governing nucleation density and rate in the EF-SCN system. In contrast, under high MHC conditions, the flake area is approximately 3–5 times larger than that under low MHC conditions, indicating that MHC flow is the limiting factor determining the lateral growth rate. From the perspective of MOCVD conditions, more effective single-crystal growth requires a combination of low DES flow, which enables single nucleation, and high MHC flow, which promotes a high lateral growth rate.

Supplementary Note 6. Strategies for effective single-crystal growth

Supplementary Fig. 13 presents a strategy for achieving single-crystal growth through the EF-SCN system. To attain effective single-crystal MoS_2 growth at a given L_{growth}, both an appropriate DES flow to enable single-centered nucleation and an MHC flow to ensure sufficiently rapid lateral growth are required. Supplementary Fig. 13a shows a schematic illustrating the principles of single nucleation formation for both small and large L_{growth}. In the small L_{growth} case, zero nucleation tends to occur due to the strong etching-flux; therefore, a high DES condition should be applied to induce single nucleation (Supplementary Fig. 13a (i) and (ii)). Conversely, in the large L_{growth} case, multiple nucleation easily occurs due to the insufficient etching effect at the central region. Thus, a low DES condition is required to achieve single nucleation (Supplementary Fig. 13a (iii) and (iv)). As shown in the SEM images of Supplementary Fig. 13b, zero-single-multiple nucleation were clearly observed and effective single-crystal growth was achieved for L_{growth} of 3 μm and 7 μm.

Achieving high-quality single-crystal growth within a defined growth region size ultimately requires two key conditions: a DES concentration that favors single nucleation events, and a sufficiently high MHC flow rate to ensure rapid lateral growth. Under these conditions, we discuss the effective range of the etching-flux effect achievable with the EF-SCN system, and the extent to which it enables the growth of large-size single crystals. Supplementary Fig. 14 presents the growth behavior observed under varying growth coverage conditions in linear etching-flux barrier patterns with line widths of 20 and 40 μm. Across both pattern widths, a decrease in growth coverage is accompanied by a progressive narrowing of the NAR, an increase in critical length, and a reduction in intra-pattern saturation coverage, consistent with trends established in small-size triangular pattern geometries. Furthermore, the observation of zero nucleation even at a line width of 20 μm under low coverage conditions suggests that, provided the lateral growth rate of MoS_2 can be sufficiently enhanced, the formation of large single crystals on the order of tens of micrometers should be attainable.

Supplementary Note 7. Hybrid diffusion-reaction process emulation of EF-SCN

To complement the nucleation-rate model described in Supplementary Note 3 and to interpret the stochastic growth sequence in the triangular template, we performed kMC-based hybrid diffusion-reaction process emulation. The emulation was used to explain how changes in experimental conditions lead to different nucleation outcomes based on local transport, adsorption, nucleation, lateral growth, and oxygen-mediated etching events. It is therefore not intended to determine absolute kinetic constants from first-principles energetics, but to explain how the spatial balance between precursor supply and oxygen etching-flux produces the NAR/NSR distribution and the zero-single-multiple nucleation regimes observed experimentally.

I. Emulation method

Extended Data Fig. 7 illustrates the emulation framework and the physical processes represented in the model. Extended Data Fig. 7a(i) shows the orthogonal view of the triangular growth template. Precursor species are supplied from the upper reservoir, oxygen etchants are supplied from the HfO_2 sidewall region, and the local surface events include adsorption, nucleation, lateral growth, and etching. Extended Data Fig. 7a(ii) shows the top-view flux geometry. Oxygen etching-flux propagates inward from the template boundary, whereas precursor species are supplied from above the growth region. Thus, the boundary region has a high etching-flux and the center region has a lower etching-flux. This spatial contrast is the origin of the NAR/NSR distribution used in the emulation.

Extended Data Fig. 7b shows how this framework is connected to the zero-, single-, and multiple-nucleation outcomes. In the zero-nucleation case, adatom formation is insufficient or etching is too strong, so nucleation fails and no growth occurs. In the single-nucleation case, side adatoms are etched near the boundary, while adatoms accumulate near the center and generate a centered nucleus. Subsequent lateral growth from this nucleus gives single-crystal growth. In the multiple-nucleation case, adatoms are widely distributed in the active region, many nucleation sites appear, and the final structure becomes poly-crystal-like. The three main control factors in this emulation are pattern size, adsorption probability, and lateral growth probability, which are related to L_{growth}, DES flow, and MHC flow, respectively.

The growth template was represented on a simple hexagonal lattice, and each lattice site was assigned one of six states: vacuum, substrate, oxygen etchant, gas-phase precursor, adsorbed adatom, or MoS_2 nucleus. The transport step was implemented using the SPPARKS on-lattice kMC framework[31] with Kawasaki spin-exchange moves[32], in which neighboring lattice sites attempt to exchange their site states with probabilities determined by pair interaction weights. The diffusion and reaction modules were coupled through an operator-split reaction-diffusion cycle[33,34].

During each outer cycle, oxygen and precursor species first diffuse from the sidewall and top reservoirs, respectively. Smaller pair interaction weights allow more frequent exchange and therefore faster effective transport. The key transport weights were w_{13} for vacuum-oxygen exchange and w_{14} for vacuum-precursor exchange, which control the oxygen flux and precursor flux, respectively. The updated configuration was then passed to the reaction module, which was implemented as an asynchronous stochastic cellular automaton. Sites were visited in randomized order, and accepted changes became visible to later events in the same cycle[35]. Similar on-lattice stochastic approaches have been used to describe TMD nucleation and morphology evolution[36,37].

The reaction module applies adsorption, nucleation, lateral growth, and etching, followed by replenishment of the precursor and oxygen reservoirs. Precursor species adjacent to substrate

sites convert into adatoms with probability P_{ads}, representing precursor sticking and DES-dependent effective nucleation density. Connected adatom clusters are converted into nuclei when their size exceeds the critical size N_c, consistent with the role of a critical cluster in vdW heteroepitaxy[38]. Once formed, all nuclei are assigned the same epitaxial orientation, $\varphi=\pi/2$, corresponding to the upright triangular direction compatible with the sapphire-guided growth direction, thereby preventing misorientation during coalescence in the emulation.

Lateral growth from a formed nucleus was described by an angular growth probability. This rule was used to preserve the triangular MoS_2 morphology associated with anisotropic edge stability and CVD shape evolution[39,40].

$$P_{grow}(\alpha) = P_{grow,base} \frac{cos(\pi/3)}{cos(((\alpha-\varphi)\ mod\ (2\pi/3))-\pi/3)} \quad (1)$$

Here, $P_{grow,base}$ is the maximum lateral growth probability and is related to the MHC-dependent lateral growth rate. After lateral growth, a Wulff-type reshape operation was applied to conserve the nucleus area while removing sites protruding outside the local triangular envelope. This step prevents artificial roughening caused by the coarse lattice representation and keeps the simulated domains consistent with the experimentally observed triangular morphology.

Oxygen-mediated etching was applied to precursors, adatoms, and formed nucleus sites adjacent to oxygen. All precursors in contact with oxygen were removed. For formed nucleus sites, etching was described using coordination-dependent probability, so that less-coordinated edge sites are more vulnerable than highly coordinated interior sites[41,42]. The etching probability for a formed nucleus site with in-plane coordination number n was written as

$$P_{etch}(n) = P_{ref}\ exp(-n\varepsilon) \quad (2)$$

Here, P_{ref} is the reference etching probability assigned to zero-coordinated nucleus sites, and ε controls how strongly local coordination suppresses etching. Adatoms were treated as zero-coordinated nucleus sites and were therefore etched with P_{ref}. Each etching event consumed both the oxygen species and the reacting species.

To represent finite oxygen release from the HfO_2 boundary and oxygen depletion during growth[43], oxygen consumed during etching was only partially replenished after each reaction stage according to

$$N_{supply} \sim Binomial(N_{consumed}, f_{rec}) \quad (3)$$

In contrast, the precursor reservoir was fully resupplied after each outer cycle to maintain continuous precursor influx into the growth template. Partial replenishment of the oxygen reservoir was used to reproduce oxygen depletion at the HfO_2 boundary. The numerical parameters used in the reference condition are summarized in below table.

Parameter	Symbol	Value
Reference etching probability for zero-coordinated nucleus sites	P_{ref}	0.9
Adatom etching treatment	—	same as zero-coordinated nucleus sites (P_{ref} = 0.9)
Coordination-dependent etching parameter	ε	0.6
Oxygen replenishment fraction	f_{rec}	0.7
Critical nucleus size	N_c	30
Oxygen diffusion weight	w_{13}	0.142
Precursor diffusion weight	w_{14}	0.155
Substrate-vacuum weight	w_{12}	2.0
Precursor-substrate weight	w_{24}	0.0001
Vacuum-adatom weight	w_{16}	5.0
Oxygen-nucleus weight	w_{3q}	0.32
Precursor-nucleus weight	w_{4q}	0.01
Reference template scale	L_{ref}	1.0x
Epitaxial orientation	φ	π/2
Diffusion-time parameter per cycle	M	500
Total reaction-diffusion cycles	N_{loops}	500
Reference adsorption probability	P_{ads}	0.14
Reference maximum growth probability	$P_{grow,base}$	1.0

Starting from the reference single-nucleation condition, three control parameters were varied independently: relative template scale L/L_{ref}, adsorption probability P_{ads}, and maximum lateral growth probability $P_{grow,base}$. The relative template scale was swept over 0.5x, 0.75x, 1.0x, 1.25x, and 1.5x; $P_{grow,base}$ over 0.05, 0.2, 0.4, 0.8, and 1.0; and P_{ads} over 0.05, 0.1, 0.14, 0.2, and 0.3. In each emulation cycle, diffusion was performed for 500 a.u., followed by reaction updates and reservoir replenishment. Each condition was calculated for 500 emulation cycles using 10 independent stochastic seeds. Final nucleation outcomes were classified by the number of surviving nuclei: zero surviving nuclei as zero nucleation, one surviving nucleus as single nucleation, and two or more surviving nuclei as multiple nucleation. Lattice configurations were rendered using OVITO[44].

For the NAR/NSR analysis, cumulative oxygen occupancy was obtained by summing oxygen occupancy over all frames and normalizing the accumulated oxygen signal. The effective NAR area was defined using both the nucleating-seed fraction and the spatial spread of nucleus positions:

$$A_{NAR} = P_{nuc}\pi r_{90}^2 \quad (4)$$

Here, P_{nuc} is the fraction of seeds that produced at least one nucleus, and r_{90} is the smallest centroid-centered radius enclosing 90% of pooled nucleus positions. For visualization, r_{eff} was used so that $\pi r_{eff}^2 = A_{NAR}$. The complementary nucleation-suppressed region was defined as

$$A_{NSR} = A_{template} - A_{NAR} \quad (5)$$

where $A_{template}$ is the total template area.

II. Emulation results and discussion

Fig. 3f shows the emulated single-centered nucleation sequence at representative outer cycles. At 0 loop, the growth region is initially empty. At 120 loops, a stable nucleus appears near the center of the triangular template. At 360 loops, the centered nucleus grows laterally and occupies a larger part of the template. At 500 loops, the template is filled by a single-crystal MoS_2 domain. This sequence is consistent with the EF-SCN process in which center nucleation is followed by lateral growth.

Supplementary Fig. 15a shows the fractions of zero, single, and multiple nucleation for the

three parameter sweeps. In the template-size sweep, smaller templates favor zero nucleation because the oxygen etching-flux reaches the central region strongly enough to suppress stable nucleus formation. At L/L_{ref} = 1.0, the single-nucleation fraction is maximized because the NAR is formed but remains spatially confined. Larger templates shift the system toward multiple nucleation because the oxygen-depleted central region expands into a broader active area. In the $P_{grow,base}$ sweep, low lateral growth probability promotes multiple nucleation because the first nucleus does not expand fast enough to capture nearby adatoms before additional clusters reach N_c. In the P_{ads} sweep, low adsorption probability reduces the rate of adatom formation, delaying cluster growth and often preventing clusters from reaching N_c within the fixed process time. In contrast, high adsorption probability rapidly increases the local adatom population within the NAR, allowing multiple clusters to exceed N_c before one nucleus can dominate the region.

Supplementary Fig. 15b shows the average number of surviving nuclei for the same parameter sweeps, providing a quantitative view of the zero-single-multiple transition. In the template-scale sweep, the average nucleus number remains below one for small templates, where oxygen etching suppresses stable nucleation. This value approaches one at the reference condition, and increases sharply for larger templates, where the oxygen-depleted region becomes broad enough for multiple adatom clusters to reach the critical size. In the $P_{grow,base}$ sweep, the average nucleus number decreases as lateral growth becomes faster, indicating that rapid growth of the first nucleus suppresses the formation of additional nuclei by consuming nearby adatoms. In the P_{ads} sweep, the average nucleus number is low when adsorption is insufficient, remains close to one near the reference condition, and increases again at high adsorption probability because excessive adatom formation allows multiple clusters to reach the critical size.

Supplementary Fig. 15c shows the corresponding average flake size. The average flake size is maximized near the single-centered condition. For small templates, excessive etching suppresses nucleation and limits lateral growth. For large templates, multiple nuclei share the same precursor supply and the average size per flake decreases. For low $P_{grow,base}$, each nucleus expands slowly and additional nuclei can form before the first nucleus dominates the template. At low P_{ads}, adatom formation is slow, so nucleation is delayed and the remaining time for lateral growth is shortened within the fixed process window. At high P_{ads}, multiple nuclei form within the NAR, and the available precursor supply is distributed among competing nuclei, reducing the average size of each flake. Thus, Supplementary Fig. 15 supports the design rule that efficient single-crystal growth requires both appropriate nucleation density and sufficiently fast lateral growth.

Supplementary Fig. 16a shows normalized cumulative oxygen concentration maps for different relative template scales. Across all scales, oxygen remains high near the template boundary and low near the center. This spatial profile arises because oxygen is supplied from the boundary-localized reservoir and is consumed during etching, producing higher oxygen occupancy near the template boundary and lower occupancy toward the center. Supplementary Fig. 16b compares the pooled nucleus positions with the effective NAR boundary. Nuclei are concentrated in the oxygen-depleted central region, showing that nucleation occurs preferentially where the etching-flux is weaker.

Supplementary Fig. 16c, d quantify this behavior by plotting the effective NAR radius and the NAR/NSR ratio as functions of relative template scale. Both values increase with template scale. Therefore, the single-centered regime corresponds to the condition where the NAR is generated but remains sufficiently confined near the center. When the template becomes larger, the NAR expands and supports several possible nucleation positions, leading to multiple nucleation.

Supplementary Fig. 16e compares selective growth with and without the boundary oxygen

etching-flux. With the etching-flux included, adatoms accumulate near the center, a single-centered nucleus forms, and lateral growth produces a single-crystal domain. Without the boundary etching-flux, adatoms distribute more uniformly across the template, multiple nuclei form, and the final structure becomes poly-crystal-like. This comparison shows that the central NAR is not generated only by triangular geometry or precursor supply. It is produced by the spatially non-uniform oxygen etching-flux supplied from the HfO_2 boundary.

Overall, the hybrid diffusion-reaction emulation provides a spatially resolved interpretation of the EF-SCN design rule. It reproduces the centered nucleation sequence, the zero-single-multiple transition, the dependence on template size and MOCVD-related parameters, and the expansion of the NAR with increasing template scale. Although the emulator does not resolve absolute atomistic reaction barriers, temperature-dependent rate constants, or an experimental length threshold, it shows how spatially separated precursor and oxygen fluxes organize stochastic nucleation events into the deterministic single-centered regime observed in EF-SCN.

III. Time-evolution emulation of nucleation and growth in EF-SCN system

Supplementary Video 1 presents the time evolution of the size-dependent zero-, single-, and multiple-nucleation regimes in the kMC-based hybrid diffusion-reaction process emulation. The three panels show representative results at relative template scales of 0.5×, 1.0×, and 1.5×, respectively, using a common physical scale and synchronized diffusion-reaction cycles from 0 to 500. In the smallest template, the boundary-derived oxygen etching-flux suppresses stable nucleus formation throughout the growth region, resulting in zero nucleation. At the reference template scale, a stable nucleus forms near the center and subsequently expands laterally to fill the template, reproducing the single-centered nucleation sequence. In the largest template, the expanded center-localized NAR accommodates multiple stable nuclei, which grow simultaneously and ultimately produce the multiple-nucleation regime. This temporal evolution directly visualizes how template size regulates the spatial extent of the NAR and determines the zero-single-multiple nucleation transition.

Supplementary Video 2 demonstrates centered nucleation and subsequent shape-programmed growth in growth regions with different geometries. Supplementary Video 2a, b show the temporal evolution of nucleation and lateral growth in triangular and square growth regions that are size-matched according to their inscribed-circle dimensions. In both geometries, nucleation initially occurs near the center, followed by lateral growth conforming to the template boundary, indicating that center-localized nucleation is not restricted to triangular patterns. Supplementary Video 2c further illustrates nucleation-growth decoupling in a line-shaped template consisting of a large nucleation zone connected to a narrow only-growth zone. A nucleus formed near the center of the nucleation zone expands into the only-growth zone, where additional nucleation is suppressed while lateral growth remains allowed. Consequently, a continuous line-shaped domain is formed from a single nucleation event. Together, these videos provide a time-resolved visualization of the EF-SCN design rule and demonstrate how spatial regulation of nucleation and lateral growth enables both deterministic nucleation control and programmable single-crystal geometries.

Supplementary Note 8. Large-scale device uniformity of EF-SCN method

I. Device performance uniformity in previously reported TMD growth methods

To establish a basis for evaluating the uniformity of single-crystal MoS_2 grown by EF-SCN, we benchmarked previously reported studies. Based on a survey of 11 publications, the device-to-device uniformity of polycrystalline/coalesced film-based devices showed a broad mobility RSD distribution ranging from 8.8% to 29.7%[45-47], with an on-current RSD of 9.1%[46]. For single-crystal epitaxial films, mobility RSD was generally around 15%[48-50], with the best-performing cases reported as low as 8%[51-53]. On-current RSD for these films was reported in the range of 16–20%[48,52,53].

For confined-growth approaches, which are most closely related to this work, reported mobility RSD ranged from 11.4% to 24.9%, and on-current RSD ranged from 12.9% to 25.2%[20,21]. The mobility RSD of 21.8% reported in the main text (Fig. 4), obtained from 16 devices on a 1 cm substrate, also falls within this typical range for confined-growth systems. However, we further conducted the following experiment to verify the uniformity of the EF-SCN growth method at a larger scale.

II. Uniformity of MoS_2 grown by EF-SCN on a 2 cm substrate

Extended Data Fig. 9a–c present a photograph and optical microscope (OM) images of back-gated field-effect transistor (BG-FET) arrays fabricated from single-crystal MoS_2 on a 2 cm SiO_2 substrate by EF-SCN. Each device had a channel length of 2.4 μm and a width of 5 μm. Source and drain electrodes were formed by depositing 40 nm of Au, followed by sequential deposition of a 1 nm Si seed layer and 10 nm HfO_2 for encapsulation. Extended Data Fig. 9d shows the transfer characteristics of the 50 devices measured at V_{ds} = 1 V. These devices exhibited an on-current RSD of 14.66% (mean 4.69 μA μm^{-1}) and a mobility RSD of 10.45% (mean 8.56 $cm^2\ V^{-1}\ s^{-1}$) (Extended Data Fig. 9e, f).

Both RSD values are comparable to those reported for confined-growth-based studies discussed above (on-current RSD 12.9–25.2%; mobility RSD 11.4–24.9%)[20,21], demonstrating that the EF-SCN growth method achieves excellent uniformity even at larger scales.

Supplementary Note 9. Benchmark of 2D monolayer MoS_2 ASG

Conventional 2D TMD ASG has been explored primarily through two approaches: seed growth[16-19] and confined growth[20,21]. In this note, we elaborate on the novelty and advantages of EF-SCN in terms of process compatibility and advanced single-crystal device implementation, based on the benchmarking presented in Supplementary Table 4.

A. Process compatibility and performance superiority

Most reported seed growth approaches employ high growth temperatures in the range of 650–750 °C, whereas confined growth based on edge nucleation has demonstrated effective single-crystal growth at a low temperature of 385 °C. In the EF-SCN system, we have an etching-flux barrier compatible with low-temperature processing, enabling successful single-crystal MoS_2 growth even at 500 °C. This result confirms that EF-SCN is a highly effective single-crystal growth strategy that can be applied to low temperature processes. Furthermore, it is anticipated that further optimization of the etching-flux barrier design will enable single-centered nucleation to remain effective at even lower process temperatures.

While most 2D TMD area-selective growth studies have used powder CVD, EF-SCN employs the MOCVD process, which is suitable for achieving oxygen-sensitive growth conditions. To validate the process compatibility and robustness of EF-SCN, we demonstrate high density single-crystal growth, establish a viable process window for deterministic single-crystal growth, and achieve large-scale uniformity and single-crystal growth on amorphous substrates.

In terms of device performance, single-crystal MoS_2 grown by seed growth and confined growth typically exhibits superior electrical properties compared to polycrystalline films, with reported mobilities of 50–60 $cm^2\ V^{-1}\ s^{-1}$. The EF-SCN system likewise enables high-quality single-crystal growth, yielding excellent FET performance, with a maximum field-effect mobility of 117 $cm^2\ V^{-1}\ s^{-1}$, as measured from FET arrays.

B. Exclusive capability for advanced single-crystal devices

Seed growth and confined growth rely on differences in TMD binding energy between the growth and non-growth regions, which inherently results in non-deterministic nucleation initiation sites. In seed growth, nucleation can occur across the entire seed edge and surface, making precise single-crystal control difficult and typically leading to polycrystalline growth. In confined growth, nucleation may occur across the entire terrace (or entire edge) region of the growth area, limiting effective single-crystal growth to sizes of 1–2 μm at high temperature and 0.2–0.5 μm at low temperatures. By contrast, EF-SCN enables nucleation to initiate deterministically at the center of the growth region, effectively suppressing undesired nucleation. As a result, large-size single-crystal growth can be achieved, reaching 7 μm in triangular patterns and 10 μm in line patterns. This scaling of single-crystal size goes beyond simply realizing arrays of individual single-crystal single FET, and instead enables single-crystal multiple FETs, significantly enhancing the utility of single-crystal TMDs.

Beyond nucleation site control, precise orientation alignment of single-crystals further enables expansion toward advanced device architectures that are not accessible with conventional approaches. Previous studies based on seed growth and confined growth have demonstrated basic FETs, CFETs, and valleytronic devices using single-crystal TMD and vertical heterostructures. In contrast, EF-SCN enables simultaneous control of both nucleation position and crystal orientation, allowing the formation of fully aligned lateral heterostructures. Such structures open new opportunities for device fabrication, such as self-aligned ALD contact FETs, and are expected to further enable advanced engineering of strain and doping in the channel region.

V. References

1 Kumagai, Y. et al. Formation of AlN on sapphire surfaces by high-temperature heating in a mixed flow of H_2 and N_2. *J. Cryst. Growth* **350**, 60–65 (2012).

2 Akiyama, K., Murakami, H., Kumagai, Y. & Koukitu, A. In situ gravimetric monitoring of decomposition rate on surface of (10-12) r-plane sapphire for high-temperature growth of nonpolar AlN. *Jpn. J. Appl. Phys.* **47**, 3434–3437 (2008).

3 Akiyama, K., Araki, T., Murakami, H., Kumagai, Y. & Koukitu, A. In situ gravimetric monitoring of decomposition rate on the surface of (0001) c-plane sapphire for the high temperature growth of AlN. *Phys. Status Solidi C* **4**, 2297–2300 (2007).

4 Eklund, P., Sridharan, M., Singh, G. & Bøttiger, J. Thermal stability and phase transformations of γ-/amorphous-Al_2O_3 thin films. *Plasma Process. Polym.* **6**, S907–S911 (2009).

5 Zhang, L., Jiang, H. C., Liu, C., Dong, J. W. & Chow, P. Annealing of Al_2O_3 thin films prepared by atomic layer deposition. *J. Phys. D: Appl. Phys.* **40**, 3707–3713 (2007).

6 Chang, H. S. et al. Thermal stability and decomposition of the HfO_2-Al_2O_3 laminate system. *Appl. Phys. Lett.* **84**, 28–30 (2004).

7 Smith, F. W. & Ghidini, G. Reaction of oxygen with Si (111) and (100): critical conditions for the growth of SiO_2. *J. Electrochem. Soc.* **129**, 1300–1306 (1982).

8 Liehr, M., Lewis, J. E. & Rubloff, G. W. Kinetics of high-temperature thermal decomposition of SiO_2 on Si(100). *J. Vac. Sci. Technol. A* **5**, 1559–1562 (1987).

9 Sasse, H.-E. & König, U. SiO diffusion during thermal decomposition of SiO_2. *J. Appl. Phys.* **67**, 6194–6196 (1990).

10 Zhang, X.-Y. et al. Temperature-dependent HfO_2/Si interface structural evolution and its mechanism. *Nanoscale Res. Lett.* **14**, 83 (2019).

11 Vinod, A., Rathore, M. S. & Rao, N. S. Effects of annealing on quality and stoichiometry of HfO_2 thin films grown by RF magnetron sputtering. *Vacuum* **155**, 339–344 (2018).

12 Zhang, X. *et al.* Low-defect density monolayer MoS_2 wafer by oxygen-assisted growth-repair strategy. *Adv. Sci.* **11**, 2408640 (2024).

13 Chen, W. *et al.* Oxygen-assisted chemical vapor deposition growth of large single-crystal and high-quality monolayer MoS_2. *J. Am. Chem. Soc.* **137**, 15632–15635 (2015).

14 Shen, P.-C. *et al.* Healing of donor defect states in monolayer molybdenum disulfide using oxygen-incorporated chemical vapour deposition. *Nat. Electron.* **5**, 28–36 (2022).

15 Kim, T. S. *et al.* Gas-phase alkali metal-assisted MOCVD growth of 2D transition metal dichalcogenides for large-scale precise nucleation control. *Small* **18**, 2106368 (2022).

16 Han, G. H. *et al.* Seeded growth of highly crystalline molybdenum disulphide monolayers at controlled locations. *Nat. Commun.* **6**, 6128 (2015).

17 Li, Y. *et al.* Site-specific positioning and patterning of MoS_2 monolayers: The role of Au seeding. *ACS Nano* **12**, 8970–8976 (2018).

18 Ryu, B. *et al.* A facile approach for site-selective and large-area growth of MoS_2 through heterogeneous nucleation. *Appl. Surf. Sci.* **607**, 155066 (2023).

19 Li, S. *et al.* Wafer-scale and deterministic patterned growth of monolayer MoS_2 via vapor–liquid–solid method. *Nanoscale* **11**, 16122–16129 (2019).

20 Kim, K. S. *et al.* Non-epitaxial single-crystal 2D material growth by geometric confinement. *Nature* **614**, 88–94 (2023).

21 Kim, K. S. *et al.* Growth-based monolithic 3D integration of single-crystal 2D semiconductors. *Nature* **636**, 615–621 (2024).

22 Zhao, M. *et al.* Growth of atomically thin MoS_2 flakes on high-κ substrates by chemical

vapor deposition. *J. Mater. Sci.* **53**, 4262–4273 (2018).
23 Hsain, H. A. *et al.* Compositional dependence of crystallization temperatures and phase evolution in hafnia–zirconia $(Hf_xZr_{1-x})O_2$ thin films. *Appl. Phys. Lett.* **116**, 192901 (2020).
24 Murdzek, J. A., George, S. M. Effect of crystallinity on thermal atomic layer etching of hafnium oxide, zirconium oxide, and hafnium zirconium oxide. *J. Vac. Sci. Technol. A* **38**, 022608 (2020).
25 Ushakov, S. V. *et al.* Crystallization in hafnia- and zirconia-based systems. *Phys. Status Solidi B* **241**, 2268–2278 (2004).
26 Ushakov, S. V., Brown, C. E., Navrotsky, A. Effect of La and Y on crystallization temperatures of hafnia and zirconia. *J. Mater. Res.* **19**, 693–700 (2004).
27 Kim, H., McIntyre, P. C., Saraswat, K. C. Microstructural evolution of ZrO_2–HfO_2 nanolaminate structures grown by atomic layer deposition. *J. Mater. Res.* **19**, 643–650 (2004).
28 Zhang, X. et al. Low-defect density monolayer MoS_2 wafer by oxygen-assisted growth-repair strategy. *Adv. Sci.* **11**, 2408640 (2024).
29 Lee, K. et al. STEM image analysis based on deep learning: Identification of vacancy defects and polymorphs of MoS_2. *Nano Lett.* **22**, 4677–4685 (2022).
30 van der Zande, A. M. et al. Grains and grain boundaries in highly crystalline monolayer molybdenum disulphide. *Nat. Mater.* **12**, 554–561 (2013).
31 Mitchell, J. A. et al. Parallel simulation via SPPARKS of on-lattice kinetic and Metropolis Monte Carlo models for materials processing. *Model. Simul. Mater. Sci. Eng.* **31**, 055001 (2023).
32 Kawasaki, K. Diffusion constants near the critical point for time-dependent Ising models. I. *Phys. Rev.* **145**, 224–230 (1966).
33 Lampoudi, S., Gillespie, D. T. & Petzold, L. R. The multinomial simulation algorithm for discrete stochastic simulation of reaction-diffusion systems. *J. Chem. Phys.* **130**, 094104 (2009).
34 Choi, T., Maurya, M. R., Tartakovsky, D. M. & Subramaniam, S. Stochastic operator-splitting method for reaction-diffusion systems. *J. Chem. Phys.* **137**, 184102 (2012).
35 Chopard, B. & Droz, M. *Cellular Automata Modeling of Physical Systems* (Cambridge Univ. Press, 1998).
36 Nie, Y. et al. A kinetic Monte Carlo simulation method of van der Waals epitaxy for atomistic nucleation-growth processes of transition metal dichalcogenides. *Sci. Rep.* **7**, 2977 (2017).
37 Aldana, S., Wang, L., Spiridon, I. A. & Zhang, H. Understanding substrate effects on 2D MoS_2 growth: a kinetic Monte Carlo approach. *Adv. Mater. Interfaces* **11**, 2400209 (2024).
38 Okada, M. et al. Microscopic mechanism of van der Waals heteroepitaxy in the formation of MoS_2/hBN vertical heterostructures. *ACS Omega* **5**, 31692–31699 (2020).
39 Schweiger, H., Raybaud, P., Kresse, G. & Toulhoat, H. Shape and edge sites modifications of MoS_2 catalytic nanoparticles induced by working conditions: a theoretical study. *J. Catal.* **207**, 76–87 (2002).
40 Wang, S. et al. Shape evolution of monolayer MoS_2 crystals grown by chemical vapor deposition. *Chem. Mater.* **26**, 6371–6379 (2014).
41 Farigliano, L. M., Paredes-Olivera, P. A. & Patrito, E. M. Initial steps of oxidative etching of MoS_2 basal plane induced by O_2. *J. Phys. Chem. C* **124**, 13177–13186 (2020).

42 Åhlgren, E. H. et al. Atomic-scale oxygen-mediated etching of 2D MoS_2 and $MoTe_2$. *Adv. Mater. Interfaces* **9**, 2200987 (2022).
43 Shin, D. et al. Oxygen tracer diffusion in amorphous hafnia films for resistive memory. *Mater. Horiz.* **11**, 2372–2381 (2024).
44 Stukowski, A. Visualization and analysis of atomistic simulation data with OVITO – the Open Visualization Tool. *Model. Simul. Mater. Sci. Eng.* **18**, 015012 (2010).
45 Dodda, A. et al. Active pixel sensor matrix based on monolayer MoS_2 phototransistor array. *Nat. Mater.* **21**, 1379–1387 (2022).
46 Seol, M. et al. High-throughput growth of wafer-scale monolayer transition metal dichalcogenide via vertical Ostwald ripening. *Adv. Mater.* **32**, 2003542 (2020).
47 Smithe, K. K. H. et al. Low variability in synthetic monolayer MoS_2 devices. *ACS Nano* **11**, 8456–8463 (2017).
48 Liu, L. et al. Uniform nucleation and epitaxy of bilayer molybdenum disulfide on sapphire. *Nature* **605**, 69–75 (2022).
49 Huang, L.-Y. et al. Wafer-scale single-crystal WSe_2 monolayers using substrate-passivation-driven epitaxy. *ACS Nano* **20**, 2104–2113 (2026).
50 Li, C. et al. Revisiting the epitaxial growth mechanism of 2D TMDC single crystals. *Adv. Mater.* **36**, 2404923 (2024).
51 Fu, W. et al. Evidence of air-induced surface transformation of atomic step-engineered sapphire in relation to epitaxial growth of 2D semiconductors. *Nat. Commun.* **16**, 8488 (2025).
52 Zou, X. et al. Robust epitaxy of single-crystal transition-metal dichalcogenides on lanthanum-passivated sapphire. *Science* **390**, eaea0849 (2025).
53 Li, T. et al. Epitaxial growth of wafer-scale molybdenum disulfide semiconductor single crystals on sapphire. *Nat. Nanotechnol.* **16**, 1201–1207 (2021).
