# Supplementary material for: Spatially deterministic nucleation of 2D semiconductors by etching flux
Source: Nature. 2026 Oct 7;658(8135):398–406. doi: 10.1038/s41586-026-11095-1 (PMC13645665; doi:10.1038/s41586-026-11095-1)
Supplement: Supplementary file 2 [file 41586_2026_11095_MOESM2_ESM.pdf]

nature portfolio

Peer Review File

Spatially deterministic nucleation of 2D semiconductors by etching flux

Corresponding Author: Professor Kibum Kang

Any redactions in this file are there to maintain patient confidentiality, the confidentiality of unpublished data, or to remove third-party material.

This file contains all reviewer reports in order by version, followed by all author rebuttals in order by version.

Version 0:

Reviewer comments:

Referee #1

(Remarks to the Author)
This manuscript presents a new concept for localized and precise control of nucleation sites in 2D semiconductor growth and provides compelling experimental validation of the approach. The EF-SCN mechanism enables direct single-crystal growth and spontaneous formation of aligned lateral heterostructures, which in my view represents a significant advancement of area-selected growth toward the industrial adoption (previous ASG cannot grow single-crystal materials). In addition, the precise control of nucleation (zero-one-multiple) is amazing and addresses a long-standing problem in 2D material growth. I believe this manuscirpt meets the originality and technical standard for Nature, and should be published without delay. The following should be taken as constructive suggestions to further improve the presentation and interpretation.
1.Although the EF-SCN mechanism is described using a continuum-based phenomenological model, nucleation is inherently a stochastic process. In this regard, the authors are encouraged to incorporate simulations such as kinetic Monte Carlo (kMC) to compare and validate the proposed growth model from a simulation perspective.
2.Based on the proposed growth model, performing kMC simulations in which process parameters, such as the amount of etching flux and growth region size, are systematically varied to reproduce zero-single-multiple nucleation modes, along with a quantitative comparison of the conditions defining each regime, would further strengthen the logic and validity of the authors' model.
3.In previous studies on confined growth of 2D TMDs (Nature 614, 2023), HfO_2 functioned as a growth region. In contrast, in this work, HfO_2 acts as both a barrier and a source of etching flux, as shown in Fig. 2, exhibiting a distinctly different behavior. Providing additional discussion on the mechanistic differences between these two cases would help clarify the growth behavior proposed by the authors.
4.In this study, HfO_2 deposited by e-beam evaporation is used as the barrier. The authors are encouraged to discuss whether HfO_2 deposited by ALD could play a similar role as an oxygen source under EF-SCN conditions, and how differences in deposition methods might influence the proposed mechanism.
5.Beyond HfO_2, it would be valuable to examine whether other oxide barriers, such as Al_2O_3 or SiO_2, can induce nucleation control effects similar to those observed in EF-SCN. If differences arise, a comparative discussion on how variations in material properties, such as melting point, bonding energy, and surface defect characteristics, affect the nucleation behavior is suggested.
6.In Extended Data Fig. 10e, the epitaxial growth direction is parallel to the triangular barrier pattern direction. If the epitaxial growth direction does not coincide with the pattern orientation, it would be informative to discuss whether MoS_2 flakes follow the imposed pattern or retain their intrinsic epitaxial orientation, and to include the expected or observed growth behavior under such misalignment conditions.
7.In the regime where many nucleation events occur due to increased pattern size, analyzing the evolution of nucleation distributions as a function of pattern size and growth conditions is expected to be particularly informative. The authors are encouraged to experimentally explore this regime and to include a discussion on the observed trends and underlying mechanisms.

Referee #2

(Remarks to the Author)
The authors present a strategy utilizing "etching-flux mediation" to spatially program the nucleation of 2D semiconductors at the center of patterned trenches. The concept of "geometric confinement" for wafer-scale single-domain growth was established by Kim et al. in 2023. That work solved the fundamental kinetic challenge of merging nuclei. In 2024, they also demonstrated the growth of single-crystal TMDs at 385 °C, enabling Back-End-of-Line compatible Monolithic 3D integration. In contrast, the current work relies on high-temperature growth (700 °C–800 °C) to activate the etching mechanism. In the context of the semiconductor roadmap, abandoning the low-temperature progress achieved in 2024 to return to high-temperature processing—merely to achieve "center nucleation"—seems to diverge from the industry's critical trajectory toward low-thermal-budget integration. Therefore, the scientific significance of this work should be clarified further.

Below are several fundamental concerns regarding the validity and practical utility of the proposed approach.

1. What is the specific, quantifiable advantage of "Center Nucleation" over the low-temperature "Edge Nucleation" strategy? If edge-nucleated crystals are already single-crystalline and BEOL-compatible, why should the industry adopt a high-temperature (>700 °C), oxygen-mediated process?
2. The proposed growth strategy exhibit extremely low nucleation density (~one nucleus per tens of square micrometers) and posing a risk of nucleation failure, which may be harmful than grain boundaries during large-area integration. What's more, slight variations in oxygen flow dynamics across a wafer could lead to over-etching or under-etching. Thus, what is the specific relationship between the growth outcomes and oxygen partial pressure? Furthermore, what is the wafer-scale uniformity of this method?
3. Continuous oxygen flux would induce unintentional etching, how does this affect the intrinsic material quality?
4. On a flat substrate, the single-crystallinity of TMDs could be easily confirmed by the triangular or hexagonal geometries due to the kinetic Wulff criterion (ACS Nano 2016, 10, 4330). However, in this space-confined strategy, the final triangular morphology of the TMD domains could be determined by anisotropic etching. Therefore, the authors should provide more effective characterizations for the single-crystallinity of the as-grown arrays, like TEM?
5. Does the high-temperature oxygen anneal degrade the surface roughness or interface trap density of the underlying dielectric? A roughened interface due to etching would render this method unsuitable for high-performance logic. Please provide more discussion.
6. To evaluate the reliability of this method, what is the statistical performance distribution of FET devices fabricated on the large-area TMD single-crystal arrays?
7. Regarding the spatially programmed lateral heterostructures, what is the atomic sharpness of the interface?
8. Can this oxygen-mediated strategy support vertical heterostructure growth without destroying the underlying TMD layer?

Referee #3

(Remarks to the Author)
I co-reviewed this manuscript with one of the reviewers who provided the listed reports.

Referee #4

(Remarks to the Author)
Area-selective growth has been widely developed in 2D and nanoscale electronics for precise control of high-quality materials with ideal device performance. Single crystal epitaxial growth of 2D materials has also become very important in further improvement of high-quality materials without twin boundaries and alloys. The research of ASG strategies such as selective chemical vapor deposition in 2D growth is meaningful in the study of 2D channel layers for mass production.
This manuscript contributes meaningfully to that direction by introducing an etching-flux mediated single-nucleation method. Oxygen-flux induced by HfO_2 has been applied in the restriction of MoS_2 growth near the nucleation barrier. MoS_2-$MoSe_2$ heterostructures and double-aligned 2D/3D hetero-patterns are realized. The approach yields line-shaped MoS_2, exhibiting excellent electrical performance with a maximum mobility of 72 cm2V-1s-1 and an on/off ratio above 108. In addition, a model has been proposed to describe the oxygen flux, oxygen concentration, and MoS_2 nucleation rate within the growth region, which further elaborate the etching behavior of EF-SCN.
In my evaluation, the current version of the manuscript shows moderate level of novelty since both oxygen etching behavior and selective growth methods have already been realized in previous studies. Some underlying mechanisms remain unclear that may require further evidence for more convincing conclusion. Below are the list of technical comments that should be addressed before further consideration of this manuscript.
List of technical comments:
1. One of the main conclusions in the manuscript is the single nucleation achieved under medium Lgrowth and middle Rgrowth. However, it is important to differentiate between single nucleation and multiple nucleation "film" within the triangular region in the nucleation site. Current results only show AFM height profiles to identify the grain boundary in multiple nucleation images. For polycrystalline films, the height profile can also be smooth if the grain boundary is in atomic scale. Would the oxygen etching-flux actually perform etching after the multiple nucleation film growth? Therefore, further evidence should be provided in order to clarify whether it is really single crystal through single nucleation growth.
2. For practical application, the process window is very important. It shows in Fig. 3b and Fig. 3c that only when Lgrowth = 3 μm and DES is 1 sccm, the claimed single nucleation can be effectively achieved. I would suggest to briefly describe whether it is possible to further enlarge the process window for larger tolerance. In addition, it is shown in Fig. 3f that the critical length becomes almost 0.6 μm when Lgrowth = 3 μm, which is different from what the authors stated "as Lgrowth increases, the critical length remains nearly constant."
3. In the nucleation probability profiles shown in Fig. 3. Error bar should be indicated and well-defined in the statistical

results.
4. It seems that the triangle orientation is randomly distributed in multiple nucleation scheme. It would be important to know whether the triangle orientation is uniform or not in single nucleation condition across the nucleation sites. This can also help to clarify whether it is single nucleation or multiple nucleation to form the film first then etched away by the oxygen flux.
5. Miscut sapphire substrates have been used for the epitaxial growth of 2D TMD including MoS_2. As mentioned in the above comment, the triangle orientation is randomly distributed in multiple nucleation scheme. It may be critical to understand whether the terrace of the substrate under specific miscut angle and and EBL patterned angle are aligned.
6. For the fully filled crystal line in Extended Data Fig. 10, it would also be important to use methods other than AFM to further confirmation of the single crystal MoS_2. Methods such as TEM would provide more definitive structural validation.

Referee #5

(Remarks to the Author)
I co-reviewed this manuscript with one of the reviewers who provided the listed reports.

Version 1:

Reviewer comments:

Referee #1

(Remarks to the Author)
I have reviewed the revised manuscript and the authors' point-by-point responses. I appreciate the authors' careful and comprehensive efforts to address the comments raised during the review process.

The authors have responded to my previous comments in a scientifically rigorous and satisfactory manner. In particular, the addition of simulation results strengthens the mechanistic support for the proposed nucleation control process. These simulations provide a clearer physical basis for understanding how the nucleation position can be deterministically guided, and they make the central mechanism of the manuscript more convincing.

I also appreciate that the authors have added several important datasets beyond the specific scope of my original questions. The additional single-crystal confirmation, the large-area single-crystal nucleation yield, the demonstration of etching-flux-mediated nucleation control, and the control of single-crystal coverage collectively support the robustness and process maturity of the proposed approach. These results improve the completeness of the manuscript and provide stronger evidence that the observed nucleation behavior is controllable and scalable.

The revised device data also broaden the significance of the manuscript. Compared with the original manuscript, the revised version not only shows improved mobility but also demonstrates multi-channel FETs fabricated within a single crystal. This provides a more concrete basis for considering the potential relevance of this growth strategy to future device architectures, including logic standard cells or NAND-related applications.

Taken together, the newly added datasets further strengthen an already compelling manuscript by improving the mechanistic foundation, demonstrating process robustness, supporting scalability, and clarifying the novelty of the work. In my view, the revised manuscript now presents an even more complete and scientifically convincing study.

I only have a few minor suggestions that may further improve the presentation of the revised manuscript. These suggestions are intended to help the authors highlight the newly added results more effectively.

1. The revised manuscript now includes many important additional datasets. Given the significance of some of the newly added results, the authors may consider reorganizing the figure structure. Some of the newly added data appear more central to the main message than the current Extended Data Fig. 1. Therefore, the authors may consider moving relatively less critical panels from Extended Data Fig. 1 to the Supplementary Information and placing some of the more important newly added datasets in the Extended Data figures.

2. In Figure 3, kMC is introduced for the first time. The authors may consider briefly defining the acronym as kinetic Monte Carlo at its first appearance. In addition, although the detailed description appears to be provided in the Supplementary Information, a short summary in the main text explaining how the kMC results support the experimental nucleation-control data would help readers better connect the simulation with the experimental observations.

3. The authors may also consider adding the simulation results as a GIF or other dynamic visualization in the Supplementary Information. This could help readers more intuitively understand the temporal evolution of the simulated nucleation process and further improve the clarity of the mechanistic explanation.

Referee #2

(Remarks to the Author)
The authors have answered most of my questions in the last reply. Although they have compared the proposed method with reported works and supplemented a substantial amount of data, the core contribution of this manuscript remains a

sophisticated process optimization rather than a fundamental conceptual breakthrough. The EF-SCN method offers an elegant technical approach for specific engineering applications; however, when viewed alongside recent achievements in wafer-scale single-crystal TMD growth (such as 2–8 inch step-guided epitaxy on sapphire), its distinct advantages regarding overall integration density or process simplicity are not entirely clear. Overall, upon a rigorous evaluation of the manuscript and the revised data, I am still concern about the core novelty and broader impact of this work.

There are several critical points remain further clarification.

1. The authors mention that the HfO_2 barrier eventually exhibits a "loss of oxygen-releasing ability". If the EF-SCN mechanism fundamentally relies on the lateral etching flux provided by HfO_2, the total available oxygen is finite and strictly governed by the volume/thickness of the barrier layer. A quantitative physical model or experimental data correlating the barrier thickness with its oxygen-releasing capacity and duration is required. It is important to understand the growth mechanism.
2. To demonstrate the high reliability and low variation, the authors fabricated "multiple FETs" on a single MoS_2 stripe and claimed a low RSD of 7.8%. This statistical approach is highly misleading. Devices fabricated by partitioning the same single-crystal stripe naturally share identical qualities. This only proves that a single crystal is locally uniform. They need to demonstrate the reliability and uniformity of the EF-SCN growth method across a large-area array. Furthermore, the variation observed among the single FETs from different sites does not currently reflect a very high level of uniformity.
3. The authors introduced a ZrO_2 barrier to achieve single-centered nucleation at low temperature. However, the intrinsic crystal quality appears to degrade significantly. Consequently, these results fail to convincingly demonstrate the superiority of this oxygen-mediated process over the previously established low-temperature "edge nucleation" methods.
4. Based on the current characterizations, the proposed method cannot clearly demonstrate an atomically sharp interface for the lateral heterostructures. In fact, the dynamic growth environment combined with continuous oxygen etching raises concerns about potential interdiffusion or alloying at the junction, which may yield inferior interface sharpness.
5. Looking at the broader implications of this work, the concepts of oxygen-assisted etching and geometrically confined selective growth of 2D materials have been previously established. While utilizing an amorphous oxide barrier as a localized, solid-state oxygen source is more like a "process optimization" rather than a fundamental breakthrough in underlying mechanisms. The authors must explicitly articulate the fundamental scientific novelty that elevates this work. What entirely new physical insight does this mechanism reveal that justifies its publication in Nature?

Referee #3

(Remarks to the Author)
I co-reviewed this manuscript with one of the reviewers who provided the listed reports.

Referee #4

(Remarks to the Author)
The authors present an interesting and well-executed study on the etching-flux mediated single-centered nucleation (EF-SCN) strategy for deterministic growth of 2D TMD semiconductors. The proposed approach effectively addresses the long-standing challenge of stochastic nucleation during 2D TMD growth by introducing a localized nucleation-active region through laterally diffusing oxygen etching-flux. The concept is moderately novel but highly significant for the controllable and scalable synthesis of high-quality 2D semiconductors.

After addressing the reviewers comments, the manuscript becomes well organized, clearly written, and supported by high-quality experimental data. The authors systematically establish the nucleation mechanism and process window through both experimental investigations and hybrid diffusion-reaction emulation, which strengthens the scientific rigor and reliability of the work. The demonstration of large-size single-crystal MoS_2 growth with excellent electrical performance, together with compatibility on large-scale, low-temperature, and amorphous substrates, highlights the practical relevance and technological importance of the proposed strategy.

In addition, the spatially programmed growth enabled by EF-SCN provides a compelling route toward advanced 2D device architectures, including multiple FETs within a single-crystal channel and self-aligned ALD-contact heterostructure devices. These demonstrations further emphasize the versatility and potential impact of the method for future integrated 2D electronics.

Overall, the methodology is sound, the statistical analysis is appropriately conducted, and the conclusions are well supported by the presented data. The authors have also adequately addressed the previous comments and concerns. I believe this work makes a valuable contribution to the field of 2D semiconductor growth and device integration, and I recommend publication in its present form.

Referee #5

(Remarks to the Author)
I co-reviewed this manuscript with one of the reviewers who provided the listed reports.

Version 2:

Reviewer comments:

Referee #2

(Remarks to the Author)
The authors have answered my questions, the work can be published.

Open Access This Peer Review File is licensed under a Creative Commons Attribution 4.0 International License, which permits use, sharing, adaptation, distribution and reproduction in any medium or format, as long as you give appropriate credit to the original author(s) and the source, provide a link to the Creative Commons license, and indicate if changes were made.
In cases where reviewers are anonymous, credit should be given to 'Anonymous Referee' and the source.
The images or other third party material in this Peer Review File are included in the article's Creative Commons license, unless indicated otherwise in a credit line to the material. If material is not included in the article's Creative Commons license and your intended use is not permitted by statutory regulation or exceeds the permitted use, you will need to obtain permission directly from the copyright holder.
To view a copy of this license, visit https://creativecommons.org/licenses/by/4.0/

Response Letter

Table of Contents

1. Overview of the Revision and Key Additions …………...………………………………………………….. 2

2. Point-by-Point Responses to Reviewer #1 Comments …………...……………………………………… 4

3. Point-by-Point Responses to Reviewer #2, #3 Comments ………………………………………………. 19

4. Point-by-Point Responses to Reviewer #4, #5 Comments ………………………………………………. 41

5. References ……………………………………………………………………………………………….. 59

1. Overview of the Revision and Key Additions

At the outset of this revision, we would like to sincerely express our gratitude to the editor and the reviewers for granting us the opportunity to revise our manuscript entitled *"Etching-flux mediated single-centered nucleation for spatially programmed growth of 2D semiconductors",* as well as for their insightful and constructive comments. The thoughtful suggestions from the reviewers have greatly encouraged us to further strengthen the manuscript, and we have addressed each point through additional experiments and demonstrations. We believe the revised manuscript presents the scientific interpretation and methodological novelty of the EF-SCN approach in a clearer and more comprehensive manner.

During the revision process, we received a total of **21 comments from five reviewers**. In response, we have not only carefully addressed each comment but also performed additional experiments and analyses to further validate the scientific rigor and practical potential of the EF-SCN method. We focused on strengthening three key aspects that were not fully developed in the original manuscript: (i) Rigorous verification of single-crystallinity, (ii) Process compatibility and scalability, and (iii) Demonstration of advanced applications that are distinct from conventional selective growth.

Before providing the point-by-point responses, we summarize the key additional results of this revision in Fig. R1 and outline the major advances achieved below.

(i) Single-crystal analysis

We first conducted a comprehensive and rigorous validation to confirm whether the MoS_2 flakes grown via EF-SCN are indeed single-crystalline. While the original manuscript presented limited analysis, in this revision, we employed various complementary techniques, including HAADF-STEM, atomic-resolution AFM, and second-harmonic generation (SHG), to systematically verify the single-crystallinity.

TEM analysis revealed identical diffraction patterns and lattice orientations across the entire flake, directly confirming the absence of grain boundaries. Atomic-resolution AFM further demonstrates a highly ordered and uniform hexagonal lattice over extended regions, with FFT data consistently indicating a single crystallographic orientation. In addition, SHG measurements exhibited spatially uniform intensity across the entire flake along with a clear six-fold symmetry, independently confirming that the EF-SCN-grown MoS_2 possesses high crystallinity and is indeed single-crystalline.

(ii) Process compatibility

We then carried out additional experiments to evaluate the process compatibility and scalability of the EF-SCN approach. The original EF-SCN system relied on a relatively high growth temperature (750 °C), which may raise questions regarding its compatibility with low-temperature processes. To demonstrate that the growth temperature is not a fundamental constraint of the approach, we introduced an alternative barrier material with a lower crystallization temperature and confirmed that single-centered nucleation can be achieved at a reduced growth temperature of 500 °C using a ZrO_2 barrier. This result indicates that EF-SCN is not intrinsically limited to the original high-temperature HfO_2 process and can be extended toward lower-temperature regimes through rational barrier engineering.

Beyond thermal adaptability, we verified high-density single-crystal growth, large-area uniformity at the centimeter scale, and successful single-crystal growth on amorphous SiO_2 substrates. These results collectively demonstrate that the EF-SCN method is not limited to specific growth conditions but can operate robustly across a wide range of process environments, indicating its strong potential for practical implementation beyond a conceptual framework.

(iii) Advanced applications

Finally, beyond the self-aligned ALD contact FETs reported in the original manuscript, we carried out additional demonstrations to realize advanced 2D TMD single-crystal architectures. By leveraging the nucleation-growth decoupling strategy, a unique capability of the EF-SCN system, we achieved the growth of a 10 µm-long line-shaped single-crystal MoS_2. A single FET defined on this long channel exhibited a high field-effect mobility of 117 $cm^2V^{-1}s^{-1}$, and the mobility distribution across 16 FET arrays showed a relative standard deviation (RSD) of 21.8%, outperforming previously reported MoS_2 FETs by selective growth.

Furthermore, to more effectively reduce the performance variation of conventional single-crystal MoS_2 FETs, we explored a one-pocket-multi-channel concept instead of the conventional one-pocket-one-channel approach. By integrating multiple FETs within a line-shaped single-crystal MoS_2, we achieved a significantly reduced mobility variation, with an RSD of 7.8%, corresponding to 65% reduction compared to single FET arrays.

These results demonstrate that the single-crystal scaling capability of EF-SCN provides a promising pathway to suppress device-to-device variation and can be further extended toward more advanced single-crystal device architectures.

In summary, this revision systematically reinforces the single-crystallinity, process compatibility, and application potential of the EF-SCN method. Through these efforts, we aim to clearly demonstrate that EF-SCN extends beyond conventional nucleation control strategies and can serve as a next-generation platform for 2D semiconductor growth based on deterministic spatial control, crystallographic alignment, and large-area single-crystal scalability.

We now proceed to provide detailed point-by-point responses to each reviewer's comment.

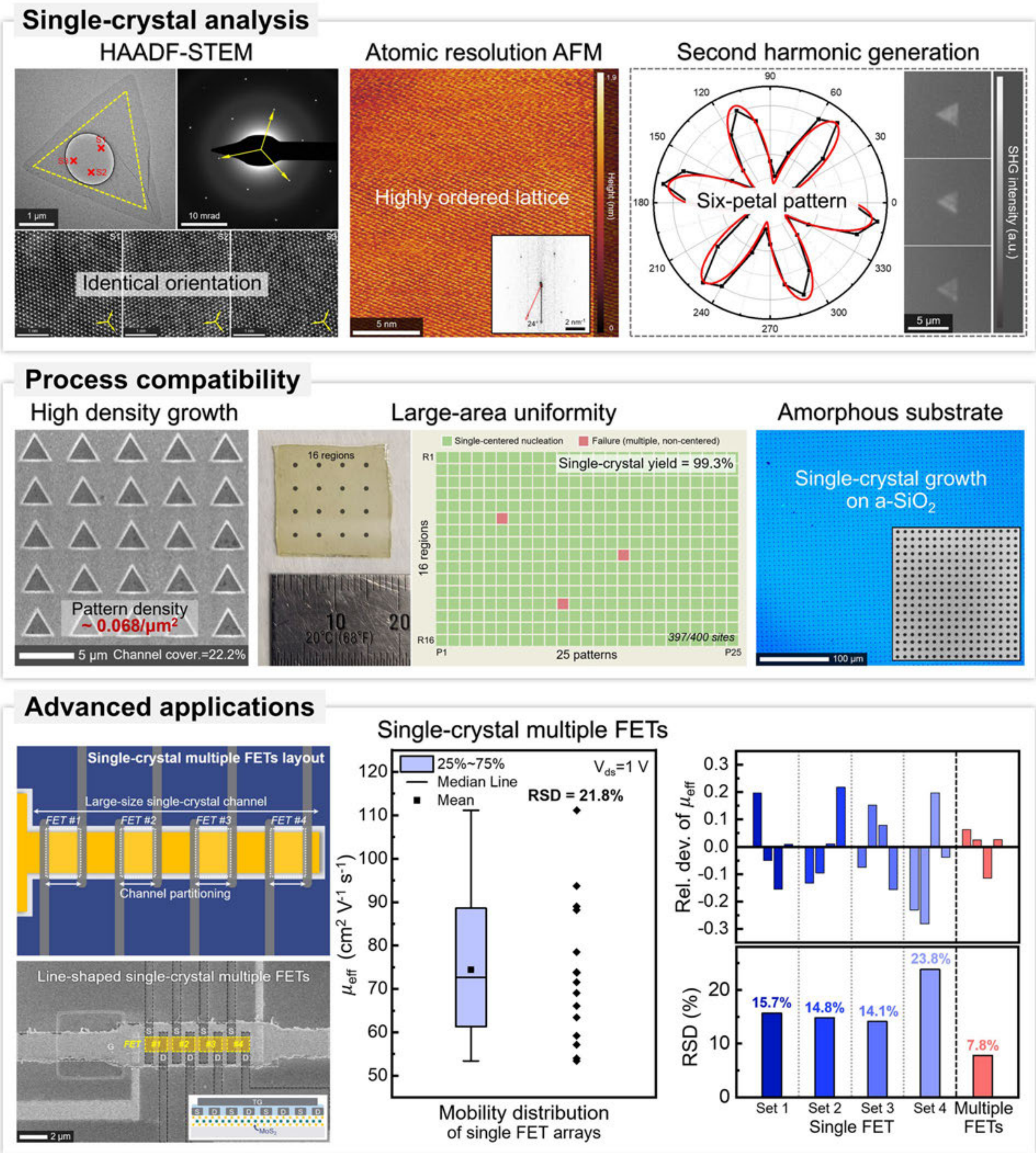

Fig. R1 | Overview of the key additional results in this revision. Single-crystal analysis, process compatibility, and advanced applications.

2. Point-by-Point Responses to Reviewer #1 Comments

(1) Although the EF-SCN mechanism is described using a continuum-based phenomenological model, nucleation is inherently a stochastic process. In this regard, the authors are encouraged to incorporate simulations such as kinetic Monte Carlo (kMC) to compare and validate the proposed growth model from a simulation perspective.

[Author reply]

We sincerely appreciate the reviewer's thoughtful suggestion and the opportunity to strengthen this part of the manuscript. We agree that the phenomenological description of the mechanism should be complemented by stochastic modeling. To provide this validation, we developed a hybrid diffusion-reaction process emulator that operates on the same triangular confinement geometry as the experiment. The emulator is structured as a reaction-diffusion operator-splitting cycle[1-3], coupling two steps: (i) a transport step for precursor and oxygen species, implemented using the on-lattice kinetic Monte Carlo (kMC) engine SPPARKS with Kawasaki exchange dynamics, and (ii) a rule-based reaction step formulated as an asynchronous stochastic cellular automaton.

This construction is in line with on-lattice TMD growth kMC approaches and with reaction-diffusion treatments[4-9]. The physical processes implemented in the reaction step are: precursor adsorption on the substrate; nucleation upon a contiguous adatom cluster reaching a critical size; anisotropic lateral growth; oxygen-mediated etching of MoS_2 and adatoms[10,11]; and partial oxygen resupply representing the gradual oxygen depletion at the HfO_2 boundary. Time and length in the emulator are treated as relative scales (cycle count and relative template scale of 1.0×); the stochastic rules for each process, numerical parameter values, and the Wulff reshape are summarized in the Supplementary Note 5.

(i) Emulation framework and process sequence

Fig. R2a visualizes this emulation framework. The orthogonal view in Fig. R2a(i) depicts the triangular growth template together with the physical processes incorporated in the emulator: precursor species are supplied from a gas-phase reservoir placed above the growth region, and oxygen species are supplied from a reservoir placed along the HfO_2 sidewalls. Within the template, the reactions proceed concurrently on the lattice. The top view in Fig. R2a(ii) schematically depicts the inward diffusion of oxygen from the HfO_2 sidewalls, where the resulting etching-flux is strongest near the boundary and weakens toward the template center. The precursor flux, in contrast, arrives uniformly from above. This geometry reproduces the experimental setup in which oxygen is emitted from the sidewall barrier while the precursor is delivered from the gas phase.

Fig. R2b traces the resulting process sequence under the reference EF-SCN condition. As precursor and oxygen species diffuse into distinct spatial distributions within the same triangular region, adatoms formed by adsorption are preferentially removed near the boundary, whereas adatoms near the center — where the etching-flux is weakest — stochastically persist and accumulate. When the accumulated adatom population reaches the critical cluster size, a nucleus forms and subsequently fills the template with lateral growth.

(ii) Nucleation regimes and emulation results

Depending on conditions such as template size, precursor adsorption, and lateral growth rate, the system exhibits zero, single, and multiple nucleation (Fig. R2b). In the single-nucleation regime, adatoms accumulate in the center region due to the strong etching effect at the boundary, leading to centered nucleation; the nucleus subsequently grows into a single-crystal via lateral growth. When the template size is smaller or the precursor adsorption is limited relative to the single nucleation condition, the adatom population becomes insufficient across the entire growth region, and nuclei fail to reach the critical size, resulting in zero nucleation. In contrast, when the template size is larger or the lateral growth rate is reduced, adatoms become widely distributed, leading to multiple nucleation sites and ultimately polycrystalline growth.

Fig. R2c presents simulation data illustrating the nucleation and growth behavior of MoS_2 over sequential loops (0 to 500). The simulation reveals single-centered nucleation at 120 loops, the expansion of the crystal via lateral growth at 360 loops, and the completion of single-crystal MoS_2 growth at the pattern center without undesired nucleation at 500 loops. Importantly, no privileged center site is imposed in advance; the centered nucleus emerges as a self-organized outcome of the local balance between precursor supply and oxygen etching. This emulation therefore provides the stochastic validation requested by the reviewer, reproducing the proposed EF-SCN mechanism — single-centered nucleation arising from this spatial balance — within the same confined geometry as the experiment.

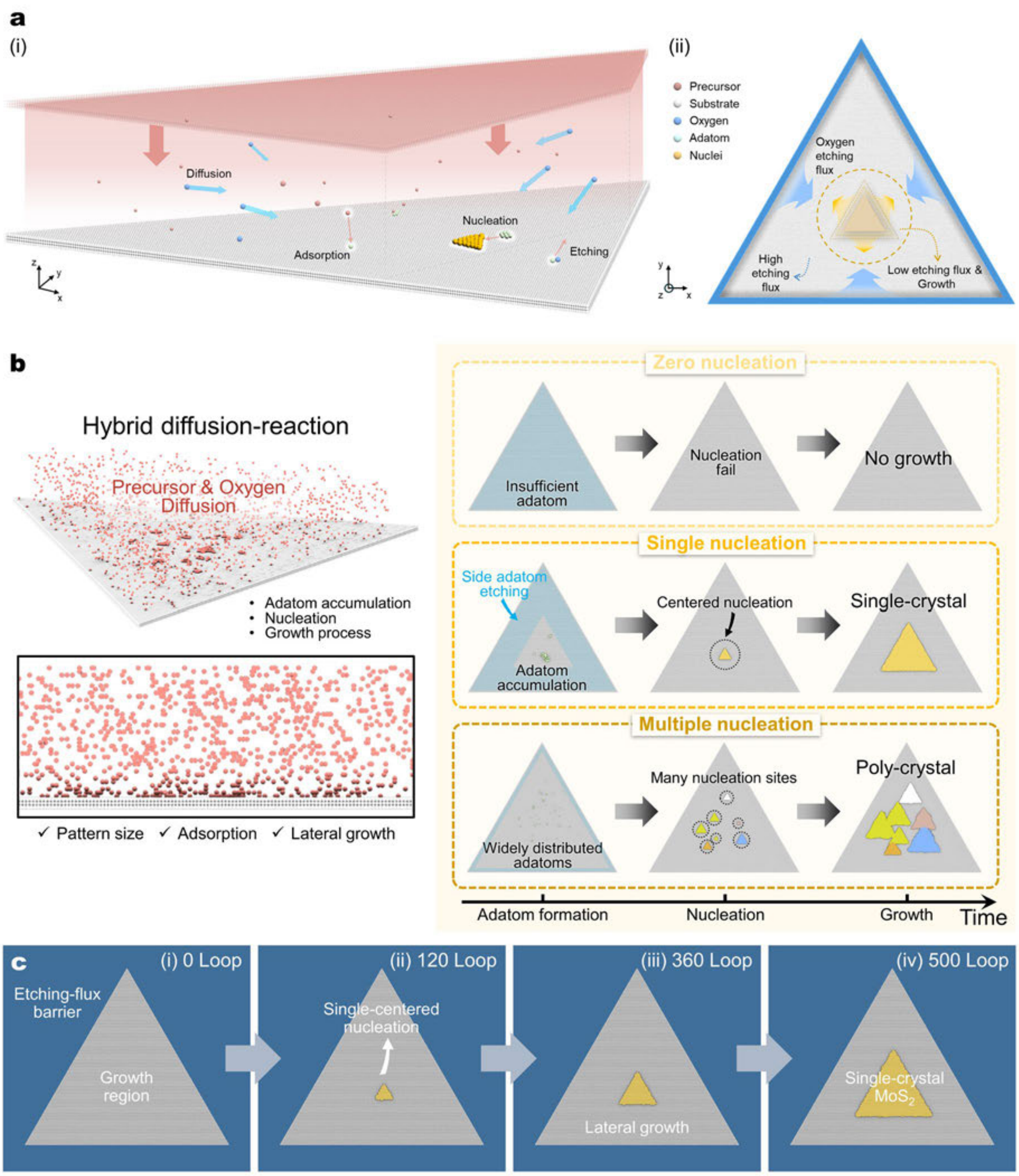

Fig. R2 | Hybrid diffusion-reaction process emulation of the EF-SCN nucleation regimes. a, Schematic of the emulation framework: orthogonal view of the triangular growth template with physical processes (i) and top view of the oxygen and precursor flux directions (ii). **b**, Time-evolution of the zero-, single-, and multiple-nucleation outcomes. **c**, Simulation images illustrating nucleation and growth behavior based on a hybrid diffusion-reaction emulation model, presented in a sequential loop.

[Action in Manuscript]

i) We have revised **Fig. 3 (p. 21)** and added **Extended Data Fig. 8 (p. 30)** to explain hybrid diffusion-reaction process emulation of EF-SCN system.

(p. 21) Fig. 3 | Nucleation design rule of 2D MoS_2 EF-SCN. f, The simulation images illustrating single-centered nucleation and lateral growth behavior based on a hybrid diffusion-reaction emulation model, presented in a sequential loop.

(p. 30) Extended Data Fig. 8 | Hybrid diffusion-reaction process emulation of the EF-SCN nucleation regimes. a, Schematic of the emulation framework: orthogonal view of the triangular growth template with physical processes (i) and top view of the oxygen and precursor flux directions (ii). b, Time-evolution of the zero-, single-, and multiple-nucleation outcomes.

ii) We have revised **manuscript (p. 9)** to demonstrate that the EF-SCN mechanism can be reproduced from a simulation perspective, which is a hybrid diffusion-reaction process emulation. Also, we added **method (p. 17)** and **Supplementary Note 5 (SI p. 29-33)** to provide a detailed description of the kMC-based emulation framework, including model assumptions, reaction rules, and parameter definitions.

(p. 9) "To further examine whether EF-SCN behavior can arise from spatially separated precursor supply and etching-flux, we performed kMC-based diffusion-reaction process emulation. In the framework schematized in Extended Data Fig. 8, diffusion and reaction steps were alternated using a cellular-automaton-based scheme[35-37], with a precursor reservoir above the growth region and an oxygen reservoir along the sidewalls to represent growth-species supply and HfO_2-derived oxygen etchants, respectively. ~"

(p. 17) "Hybrid diffusion-reaction process emulation was used to test whether spatially separated precursor supply and oxygen etching-flux can reproduce the zero-single-multiple nucleation behavior of EF-SCN. The emulation domain was defined as a simplified triangular growth template on a hexagonal lattice, with a precursor reservoir positioned above the template and an oxygen reservoir placed along the template boundary to mimic HfO_2-derived etching species. ~"

(SI p. 29-33) "To complement the nucleation-rate model described in Supplementary Note 2 and to interpret the stochastic growth sequence in the triangular template, we performed kMC-based hybrid diffusion-reaction process emulation. The emulation was used to explain how changes in experimental conditions lead to different nucleation outcomes based on local transport, adsorption, nucleation, lateral growth, and oxygen-mediated etching events. ~"

(2) Based on the proposed growth model, performing kMC simulations in which process parameters, such as the amount of etching flux and growth region size, are systematically varied to reproduce zero-single-multiple nucleation modes, along with a quantitative comparison of the conditions defining each regime, would further strengthen the logic and validity of the author's model.

[Author reply]

We are grateful for the reviewer's constructive recommendation to provide a more quantitative comparison of the conditions governing each nucleation regime through kMC-based parametric analysis. We expanded the manuscript to compare the zero, single, and multiple nucleation regimes across the main control variables of the model.

(i) Effect of process parameters on nucleation regimes

Direct single-parameter sweeps were performed along three control axes: the relative template scale (L/L_{ref}), the maximum lateral growth probability ($P_{\mathrm{grow,base}}$), and the adsorption probability (P_{ads}). Each axis is directly connected to the experimental parameters: L/L_{ref} corresponds to the patterned template size, $P_{\mathrm{grow,base}}$ reflects the lateral growth tendency governed by Mo supply, and P_{ads} represents precursor sticking, which sets the effective nucleation density and corresponds to the S supply condition used in the growth process[12]. Each condition was evaluated across 10 independent seeds.

Fig. R3a presents the fractions of zero, single, and multiple nucleation as a function of the relative template scale, lateral growth probability, and adsorption probability, while Fig. R3b shows the corresponding average number of nuclei. In all three sweeps, the reference EF-SCN condition consistently yields 90% single nucleation and 10% multiple nucleation, serving as a common baseline against which the effect of each parameter is evaluated.

Template scale (L/L_{ref}). As shown in Fig. R3a(i), decreasing L/L_{ref} to 0.5 relative to the reference condition shifts the distribution toward zero nucleation (90%) with only 10% single nucleation, whereas increasing it to 1.25 or above drives the system entirely into the multiple nucleation regime (100%). This systematic trend is independently corroborated by the average nucleus count in Fig. R3b(i), which equals unity at the reference condition and increases monotonically with L/L_{ref}.

Lateral growth probability ($P_{\mathrm{grow,base}}$). Decreasing $P_{\mathrm{grow,base}}$ from the reference condition progressively increases the fraction of multiple nucleation (Fig. R3a(ii)). For example, at $P_{\mathrm{grow,base}}$ = 0.05, the distribution shifts to 20% single and 80% multiple nucleation. The average nucleus count in Fig. R3b(ii) mirrors this trend, increasing steadily as $P_{\mathrm{grow,base}}$ is lowered.

Adsorption probability (P_{ads}). As shown in Fig. R3a(iii), decreasing P_{ads} from the reference condition shifts the distribution toward zero nucleation, whereas increasing P_{ads} promotes multiple nucleation — a trend further reflected in the average nucleus count in Fig. R3b(iii), which increases progressively with P_{ads}.

Taken together, these trends are consistent with experimental observations. Since L/L_{ref}, $P_{\mathrm{grow,base}}$, and P_{ads} correspond to the template size, Mo supply, and S supply, respectively, the simulation results map directly onto experimental growth conditions: small templates or low S supply favor zero nucleation, whereas larger templates, high S supply, or low Mo supply promote multiple nucleation. The reference condition, tuned to lie between these regimes, reproduces the experimentally observed single-centered nucleation.

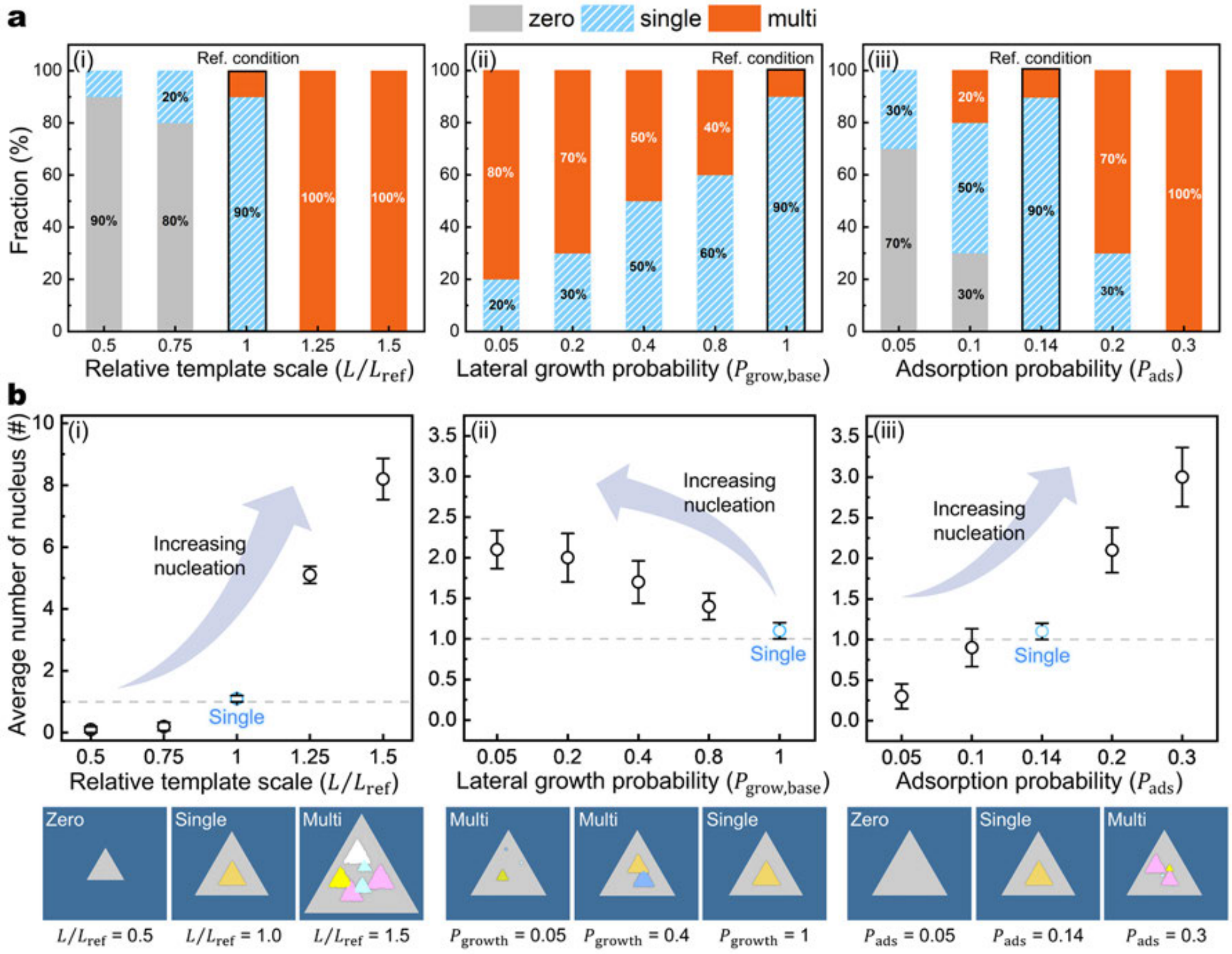

Fig. R3 | Parametric study of nucleation outcomes around the reference EF-SCN condition. a, Fractions of zero-, single-, and multiple-nucleation under three sweeps of relative template scale (i), maximum lateral growth probability (ii), and adsorption probability (iii). **b**, Average number of nuclei for the same three sweeps.

(ii) Mechanistic origin of single-centered nucleation

A. The nucleation-active region (NAR) and its template scale dependence

Building on this experimental agreement, we examined the spatial oxygen distribution and the locations of nuclei formation across the three sweeps to understand the underlying mechanism more analytically (Fig. R4).

The cumulative oxygen maps in Fig. R4a show that oxygen remains concentrated near the boundary and depleted toward the template center under all conditions. Fig. R4b visualizes the resulting nuclei, which are confined to the oxygen-depleted central region. We refer to this region as the nucleation-active region (NAR), within which the oxygen etching-flux is sufficiently weak for adatoms to survive and form a stable nucleus.

To make this analysis more quantitative, we define NAR as $\mathrm{NAR} = \pi r_{90}^2 P_{\mathrm{nuc}}$, where P_{nuc} is the fraction of seeds producing at least one nucleus and r_{90} is the smallest radius from the triangle centroid enclosing 90% of the pooled nucleus positions. The effective NAR radius is defined as $r_{\mathrm{eff}} = r_{90}\sqrt{P_{\mathrm{nuc}}}$ so that πr_{eff}^2 numerically equals NAR, and the complementary nucleation-suppressed region is defined as $\mathrm{NSR} = A_{\triangle} - \mathrm{NAR}$, where $A_{\triangle}$ is the triangle area.

The effective NAR radius and the NAR/NSR ratio in Fig. R4c and R4d show that the template size primarily governs the NAR. In a small template, the boundary oxygen source lies close to the center, resulting in a relatively high central etching-flux and preventing the formation of a stable NAR. In a larger template, the same boundary source is distributed over a wider area, leading to a relatively low central etching-flux and the emergence of a broad NAR. Consistent with this scaling, the average nucleus count increases from near zero for small templates to several for large templates (Fig. R3b(i)), and the average flake size peaks at the reference template size and decreases on either side (Fig. R5a).

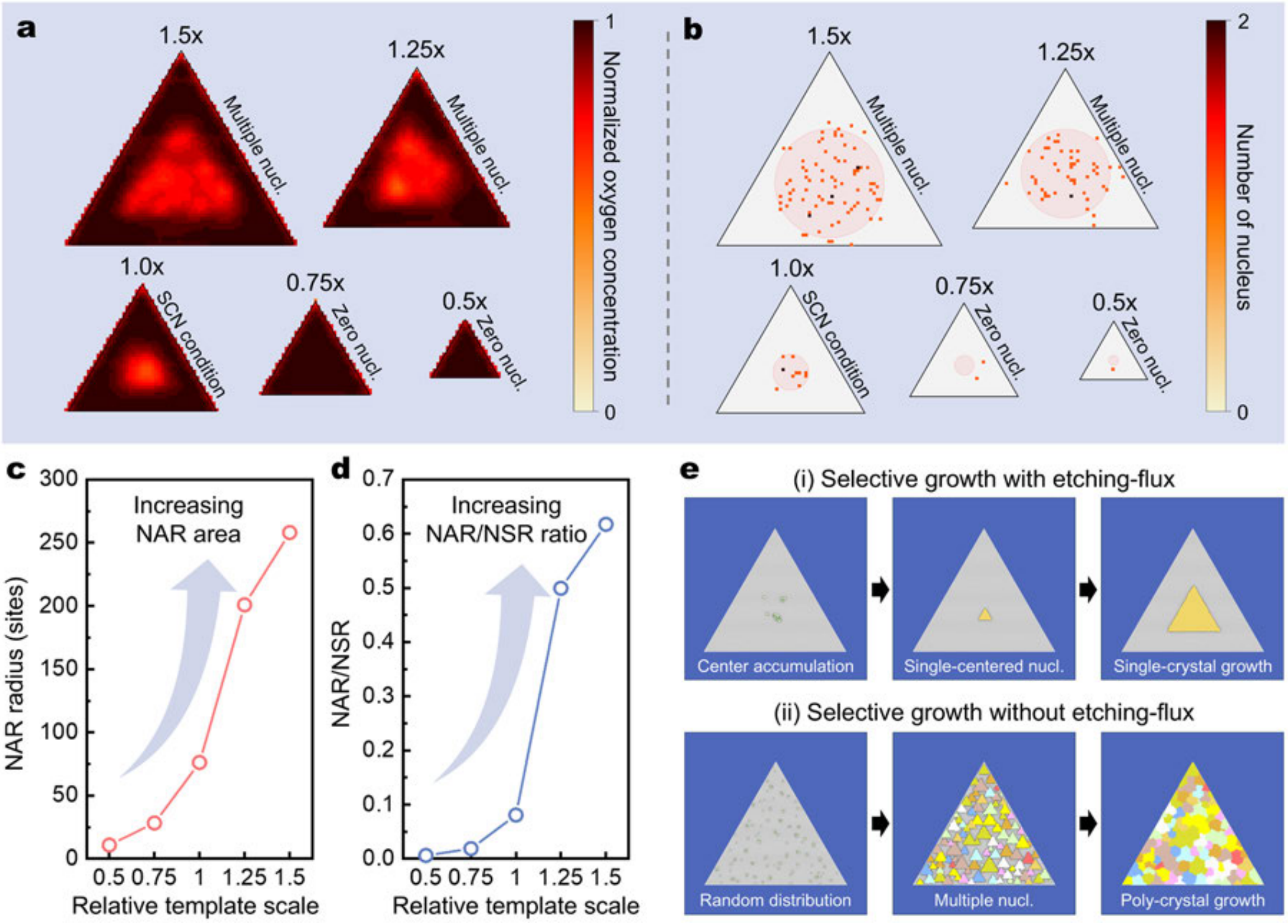

Fig. R4 | NAR/NSR analysis of the oxygen-flux origin of single-centered nucleation. a-d, Normalized cumulative oxygen concentration maps across relative template scales (**a**), Pooled nucleus positions with the effective NAR boundary (**b**), Effective NAR radius (r_{eff}) as a function of relative template scale (**c**), NAR/NSR ratio as a function of relative template scale (**d**). **e**, Reference EF-SCN condition with and without the boundary oxygen etching-flux under otherwise identical rules.

B. NAR occupancy: The role of lateral growth and adsorption

With the template held at the reference condition, $P_{\mathrm{grow,base}}$ and P_{ads} together control how the NAR is occupied. Lowering the $P_{\mathrm{grow,base}}$ raises the average nucleus count (Fig. R3b(ii)) and monotonically reduces the flake size (Fig. R5b). Under these conditions, the first nucleus cannot fully expand to fill the NAR before additional nuclei appear. Since these competing nuclei share the precursor and each expands slowly, smaller flakes are formed.

The P_{ads} sweep shows the same NAR-filling balance from both sides (Fig. R3b(iii) and Fig. R5c). At low adsorption, nucleation is delayed and the nucleus count stays near zero, so the post-nucleation growth time shortens and flakes remain small. At high adsorption, several clusters cross the critical nucleus size almost simultaneously and share the precursor, so each flake again remains smaller.

In summary, the template size first determines the size of the NAR, while adsorption and lateral growth together determine how it is occupied. Single-centered nucleation is achieved when a single nucleus grows fast enough to capture the entire NAR — a condition that requires sufficiently low nucleation density, an adequate lateral growth rate, and an appropriate template size.

Taken together, these trends are consistent with experimental observations: small templates or low S supply favor zero nucleation, whereas larger templates, high S supply, or low Mo supply promote multiple nucleation. The reference condition, tuned to lie between these regimes, reproduces the experimentally observed single-centered nucleation.

C. Control emulation: Role of the oxygen etching-flux

Finally, we isolated the direct role of the boundary oxygen flux through the on/off comparison in Fig. R4e. When the oxygen etching-flux is present, adatoms gradually accumulate in the center region, leading to single-centered nucleation and subsequent single-crystal growth. In contrast, when the oxygen source is removed, multiple and random nucleation events occur, resulting in polycrystalline growth. Therefore, the oxygen flux in the EF-SCN system serves as a key factor that effectively controls the nucleation position and enables single-centered nucleation.

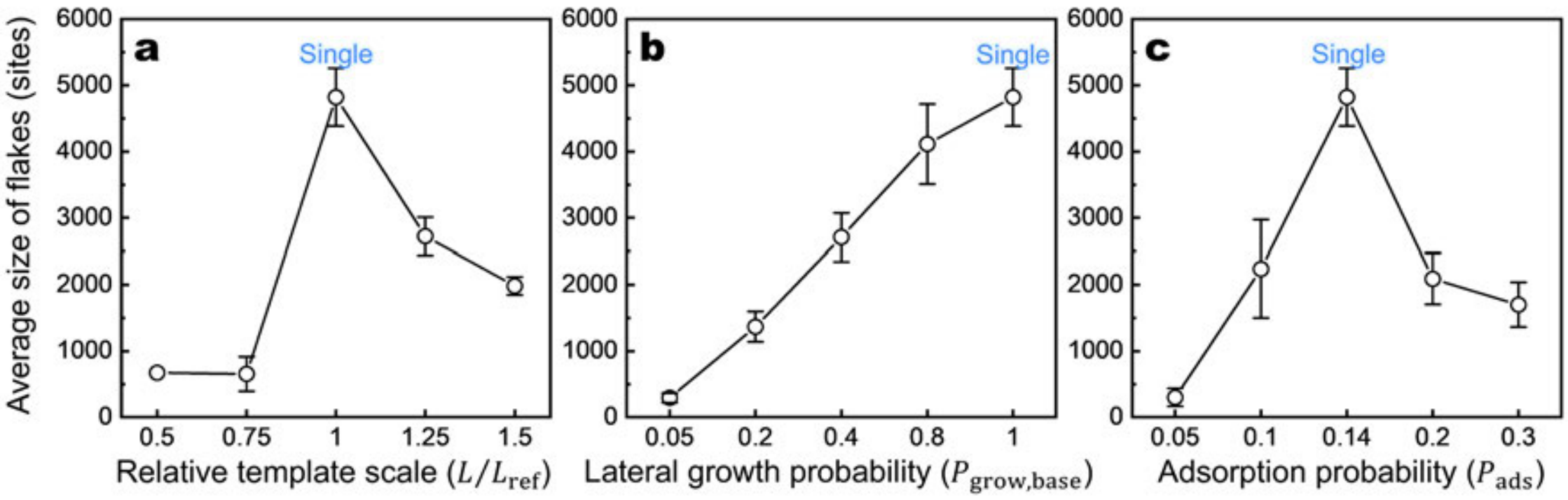

Fig. R5 | Average flake size across the three parameter sweeps. a, Dependence on relative template scale. **b**, Dependence on maximum lateral growth probability. **c**, Dependence on adsorption probability.

[Action in Manuscript]

i) We have added **Supplementary Fig. 14** and **15 (SI p. 15, 16)** to provide a systematic parametric analysis of nucleation behavior and to quantitatively demonstrate how key process variables govern the transition among zero, single, and multiple nucleation regimes.

(SI p. 15) Supplementary Fig. 14 | Parametric study of nucleation outcomes around the reference EF-SCN condition. a, Fractions of zero, single, and multiple nucleation under three sweeps of relative template scale (i), maximum lateral growth probability (ii), and adsorption probability (iii). b,c, Average number of nuclei (b) and average flake size (c) for the same three sweeps.

(SI p. 16) Supplementary Fig. 15 | NAR/NSR analysis of the oxygen-flux origin of single-centered nucleation. a-d, Normalized cumulative oxygen concentration maps across relative template scales (a), pooled nucleus positions with the effective NAR boundary (b), effective NAR radius (r_{eff}) as a function of relative template scale (c), NAR/NSR ratio as a function of relative template scale (d). e, Reference EF-SCN condition with and without the boundary oxygen etching-flux under otherwise identical rules.

ii) We have revised **manuscript (p. 10)** and added **Supplementary Note 5 (SI p. 29-33)** to explain the mechanistic origin of the observed nucleation regimes based on the kMC-based emulation and to define the relationship between process parameters and nucleation behavior within a physically grounded framework.

(p. 10) "To connect the emulation with the process trends, a compact parametric study was conducted around the single-nucleation condition. Supplementary Fig. 14 shows that larger templates and higher adsorption probabilities expand the nucleation probability and promote multiple nucleation, whereas smaller templates and lower adsorption probabilities favor zero nucleation; insufficient lateral growth also shifts the system toward the multi-nucleation regime. These trends indicate that efficient single-crystal growth requires both a nucleation density low enough to permit a single event and a lateral growth rate high enough for that event to fill the template. ~"

(SI p. 29-33) "To complement the nucleation-rate model described in Supplementary Note 2 and to interpret the stochastic growth sequence in the triangular template, we performed kMC-based hybrid diffusion-reaction process emulation. The emulation was used to explain how changes in experimental conditions lead to different nucleation outcomes based on local transport, adsorption, nucleation, lateral growth, and oxygen-mediated etching events. ~"

(3) In previous studies on confined growth of 2D TMDs (Nature 614, 2023), HfO_2 functioned as a growth region. In contrast, in this work, HfO_2 acts as both a barrier and a source of etching flux, as shown in Fig. 2, exhibiting a distinctly different behavior. Providing additional discussion on the mechanistic differences between these two cases would help clarify the growth behavior proposed by the authors.

[Author reply]

This comment raises a critical question that clearly distinguishes EF-SCN from 2D TMD confined growth. As the concept of an etching-flux barrier that releases oxygen is a newly introduced mechanism in selective TMD growth, it is necessary to explicitly clarify the growth behavior of TMD on HfO_2 surfaces.

(i) TMD growth on HfO_2 surface in prior studies

In previously reported studies on 2D TMD confined growth, selective growth of WSe_2 was achieved by using HfO_2 as the growth region and SiO_2 as the barrier region[13]. In addition, effective growth of MoS_2 on ALD-grown HfO_2 films has also been reported[14]. A common feature of these studies is the use of high growth temperature (700-900 °C) and powder CVD process employing oxide precursors. Under such high temperature conditions, oxygen can be sufficiently released from HfO_2. However, because oxide precursors are already oxygen-rich, the etching and nucleation-suppression effects from HfO_2-released oxygen are expected to be significantly reduced.

On the other hand, in edge nucleation processes at relatively low temperatures below 400 °C, the growth temperature of TMD is lower than the crystallization temperature of HfO_2, suggesting that HfO_2 cannot release sufficient oxygen[15]. Therefore, in conventional 2D TMD confined growth systems in which HfO_2 is used as the growth region, the oxygen released from HfO_2 does not provide a sufficient nucleation-etching effect. As a result, selectivity in such systems is instead governed by differences in TMD binding energy between the two surfaces.

(ii) Primary reason why HfO_2 can act as effective etching-flux barrier in our EF-SCN system

Unlike powder CVD processes based on oxide precursors, EF-SCN employs the MOCVD method using molybdenum hexacarbonyl (MHC) and diethyl sulfide (DES). As shown in Table R1, for powder CVD using a MoO_3 precursor, effective MoS_2 growth has been reported even when the oxygen concentration exceeds 1%[16-18]. In contrast, for MOCVD processes using MHC as the Mo precursor, even a small amount of oxygen (~ 0.025%) induces a measurable etching effect, and increasing the oxygen concentration to 0.04% results in complete etching, making MoS_2 growth impossible[19]. Therefore, the EF-SCN system is highly sensitive to oxygen flux due to the MOCVD process and enables precise control of nucleation positions through trace amounts of oxygen released from HfO_2.

Type	Precursor	Temperature	Ar flow	O_2 flow	O_2 / Ar+O_2	Ref
Powder CVD	**MoO_3**, S	910 °C	275 sccm	~ 8 sccm	**2.83 %**	[16]
	**MoO_3**, S	750~850 °C	100 sccm	~ 2 sccm	**1.96 %**	[17]
	**MoO_3**, S	625 °C	20 sccm	~ 0.2 sccm	**0.99 %**	[18]
MOCVD	**MHC**, DES	600 °C	400 sccm	~ 0.1 sccm	**0.025 %**	[19]

Table R1 | MoS_2 growth conditions with oxygen flow. Precursors, growth temperature, and oxygen fraction conditions for effective MoS_2 growth[16-19].

(iii) Valid growth conditions for achieving EF-SCN

To demonstrate that oxygen-sensitive conditions are essential for achieving the EF-SCN system, Fig. R6 analyzes the conditions under which HfO_2 can serve as an effective etching-flux barrier. Fig. R6a presents SEM images of MoS_2 MOCVD growth on HfO_2 films deposited by e-beam evaporation at growth temperatures of 600 and 700 °C. Consistent with the previous EF-SCN results, MoS_2 growth is completely suppressed on HfO_2 surfaces.

In contrast, when the DES flow is significantly increased from 5 sccm to 20 sccm (Fig. R6b(i) and R6b(ii)), or when the H_2 flow is increased from 0 sccm to 800 sccm (Fig. R6b(iii) and R6b(iv)), MoS_2 growth is observed even on HfO_2 surfaces. Furthermore, MoS_2 growth on HfO_2 is clearly observed after vacuum annealing at 1000 °C for 1 hour (Fig. R6c(i) and R6c(ii)) or H_2 annealing under the same conditions (Fig. R6c(iii) and R6c(iv)). These results indicate that oxygen released from HfO_2 loses its nucleation-suppression role if its effective concentration is insufficient to inhibit MoS_2 nucleation — in which case HfO_2 acts as a growth region rather than a barrier.

Fig. R6d schematically summarizes the growth conditions under which HfO_2 can serve as an etching-flux barrier. As shown in Fig. R6d(i), the EF-SCN system requires MOCVD conditions with low H_2 and low chalcogen flow, enabling the utilization of oxygen from HfO_2 for nucleation control. In contrast, when the H_2 flow is too high (Fig. R6d(ii)) or the chalcogen flow is significantly increased (Fig. R6d(iii)), the relative effect of oxygen is reduced due to the formation of a hydrogen-rich environment or an increased TMD nucleation rate, leading to reduced selectivity. As discussed above, when molybdenum oxide precursors are employed, the oxygen released from HfO_2 is unlikely to provide an effective etching effect on TMD growth (Fig. R6d(iv)). In addition, if the growth temperature is excessively high, oxygen may be

rapidly depleted (Fig. R6d(v)), whereas if the temperature is too low, insufficient oxygen is released (Fig. R6d(vi)). In both cases, HfO_2 is not expected to serve as an etching-flux barrier.

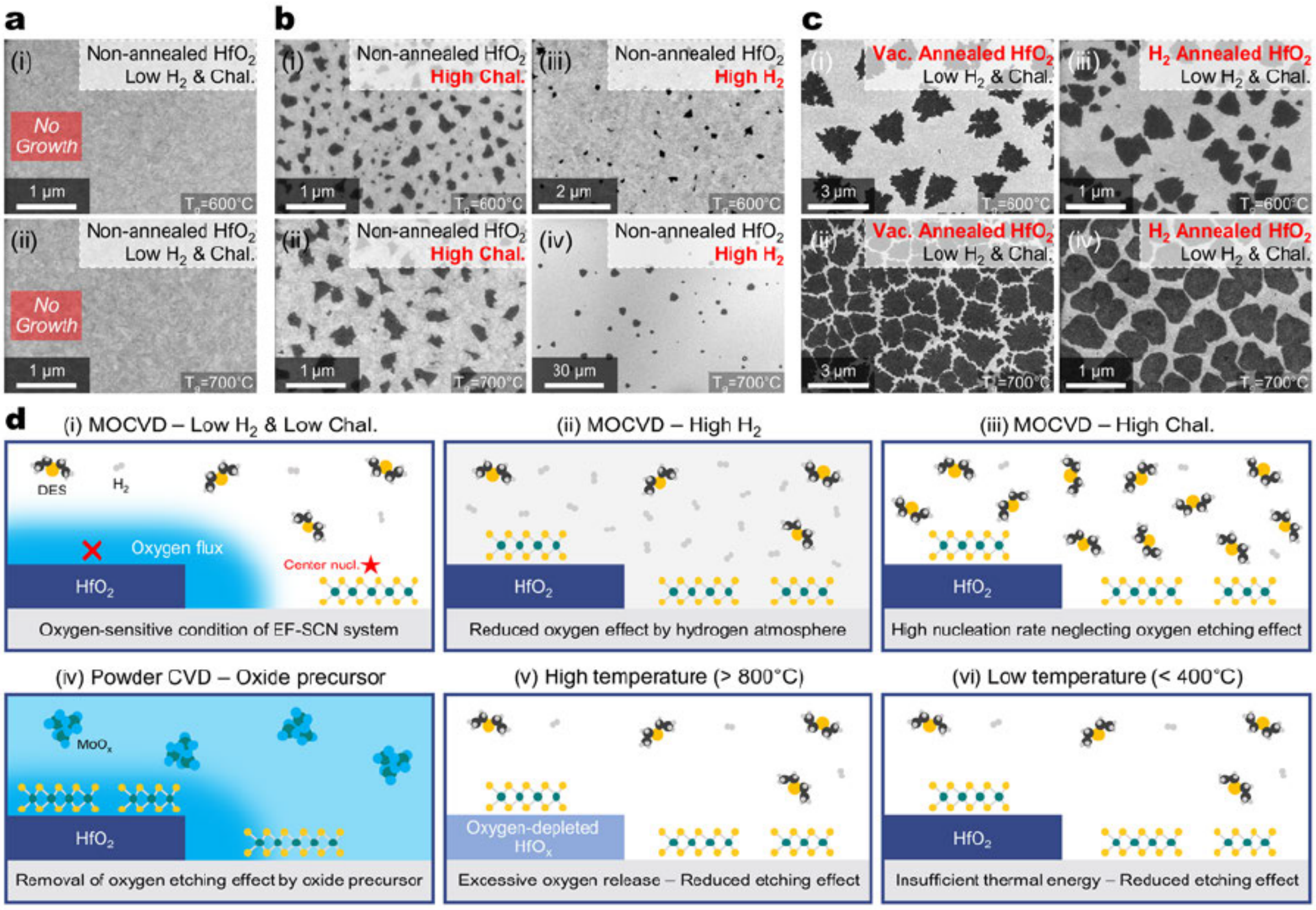

Fig. R6 | Valid conditions for HfO_2 to act as an etching-flux barrier. a-c, SEM images of MoS_2 grown on HfO_2 with low H_2 & low chalcogen condition (**a**), HfO_2 with high H_2 or high chalcogen condition (**b**), and annealed HfO_2 with low H_2 & low chalcogen condition (**c**). **d**, Schematic of MoS_2 growth on HfO_2 surface with different conditions.

(iv) Expanding the valid EF-SCN window via diversification of barrier materials

As shown in Fig. R6d, achieving nucleation positioning and single-crystal growth by EF-SCN requires specific growth conditions. In particular, maintaining a growth temperature at which sufficient oxygen can be released from the etching-flux barrier is critically important. Therefore, to improve the process window of EF-SCN, it is necessary to explore alternative barrier materials that can effectively release oxygen at lower temperatures.

[redacted]

[redacted]

[redacted]

[redacted]

[redacted]

[Action in Manuscript]

i) We have added **Extended Data Fig. 5 (p. 27)**, **Supplementary Fig. 5 (SI p. 6)**, and **Supplementary Table 2 (SI p. 18)** to verify the low-temperature applicability of EF-SCN and clarify the oxygen-sensitive process conditions required for HfO_2 to serve as an etching-flux barrier.

> **(p. 28) Extended Data Fig. 5** | Low-temperature EF-SCN and high density single-crystal growth. a,b, Schematic and SEM images showing single-crystal MoS_2 EF-SCN with high T_c barrier (HfO_2) on sapphire at 750 °C (a) and low T_c barrier (ZrO_2) on sapphire at 500 °C (b). Scale bars, 1 μm. c, SEM images of MoS_2 EF-SCN behaviors in (i) low, (ii) middle, and (iii) high pattern density substrates.
>
> **(SI p. 6) Supplementary Fig. 5** | Valid conditions for HfO_2 to act as an etching-flux barrier. a-c, SEM images of MoS_2 grown on HfO_2 with low H_2 & low chalcogen condition (a), HfO_2 with high H_2 or high chalcogen condition (b), and annealed HfO_2 with low H_2 & low chalcogen condition (c). d, Schematic of MoS_2 growth on HfO_2 surface with different conditions.
>
> **(SI p. 18) Supplementary Table 2** | MoS_2 growth conditions with oxygen flow. Precursors, growth temperature, and oxygen fraction conditions for effective MoS_2 growth[12-15].

ii) We have updated **manuscript (p. 6, 7)** and provided **Supplementary Note 1 (SI p. 21-22)** to explain why HfO_2 can serve as an oxygen-releasing etching-flux barrier in the EF-SCN system and to demonstrate low-temperature EF-SCN.

> **(p. 6)** "More detailed discussions on the oxygen-sensitive growth conditions under which HfO_2 serves as a valid etching-flux barrier are provided in Supplementary Note 1, along with Supplementary Figs. 4, 5, and Supplementary Table 2."
>
> **(p. 7)** "Therefore, we employed ZrO_2, which is known to have a lower crystallization temperature than HfO_2, as an alternative etching-flux barrier[33,34]. As shown in the SEM images in Extended Data Fig. 5b, the use of a ZrO_2 etching-flux barrier results in effective single-centered nucleation, rather than edge nucleation, even at a low MoS_2 MOCVD temperature of 500 °C. ~"
>
> **(SI p. 21-22)** "The HfO_2 barrier used in the EF-SCN system enables a new selective growth strategy by releasing an oxygen etching-flux, which induces deterministic nucleation at the center point of the growth region. Owing to the low thermal decomposition temperature of HfO_2, we confirmed that sufficient oxygen release occurs at 750 °C (Fig. 2b, c). ~"

(4) In this study, HfO_2 deposited by e-beam evaporation is used as the barrier. The authors are encouraged to discuss whether HfO_2 deposited by ALD could play a similar role as an oxygen source under EF-SCN conditions, and how differences in deposition methods might influence the proposed mechanism.

[Author reply]

This comment is considered highly important: the density, defect, and composition of oxide films can vary depending on the deposition method, and these variations can significantly influence the oxygen release behavior from HfO_2. In general, oxide films deposited by physical vapor deposition (PVD) are known to be more defective than those fabricated by atomic layer deposition (ALD). Accordingly, Fig. R8 compares the growth behavior of MoS_2 on HfO_2 films deposited by PVD (e-beam evaporation) and ALD.

As shown in Fig. R8a, MoS_2 growth is suppressed on the PVD-HfO_2 surface over a temperature range of 500-750 °C. In contrast, as shown in Fig. R8b, MoS_2 growth on ALD-HfO_2 is suppressed only within a narrower temperature range of 600-700 °C. This difference can be attributed to the lower defect density of ALD-HfO_2 compared to PVD-HfO_2, which results in reduced oxygen release during the crystallization process. Consequently, beyond employing different oxide materials as demonstrated in Fig. R7, these results suggest that the process window of EF-SCN can also be expanded by tailoring the deposition method of the etching-flux barrier.

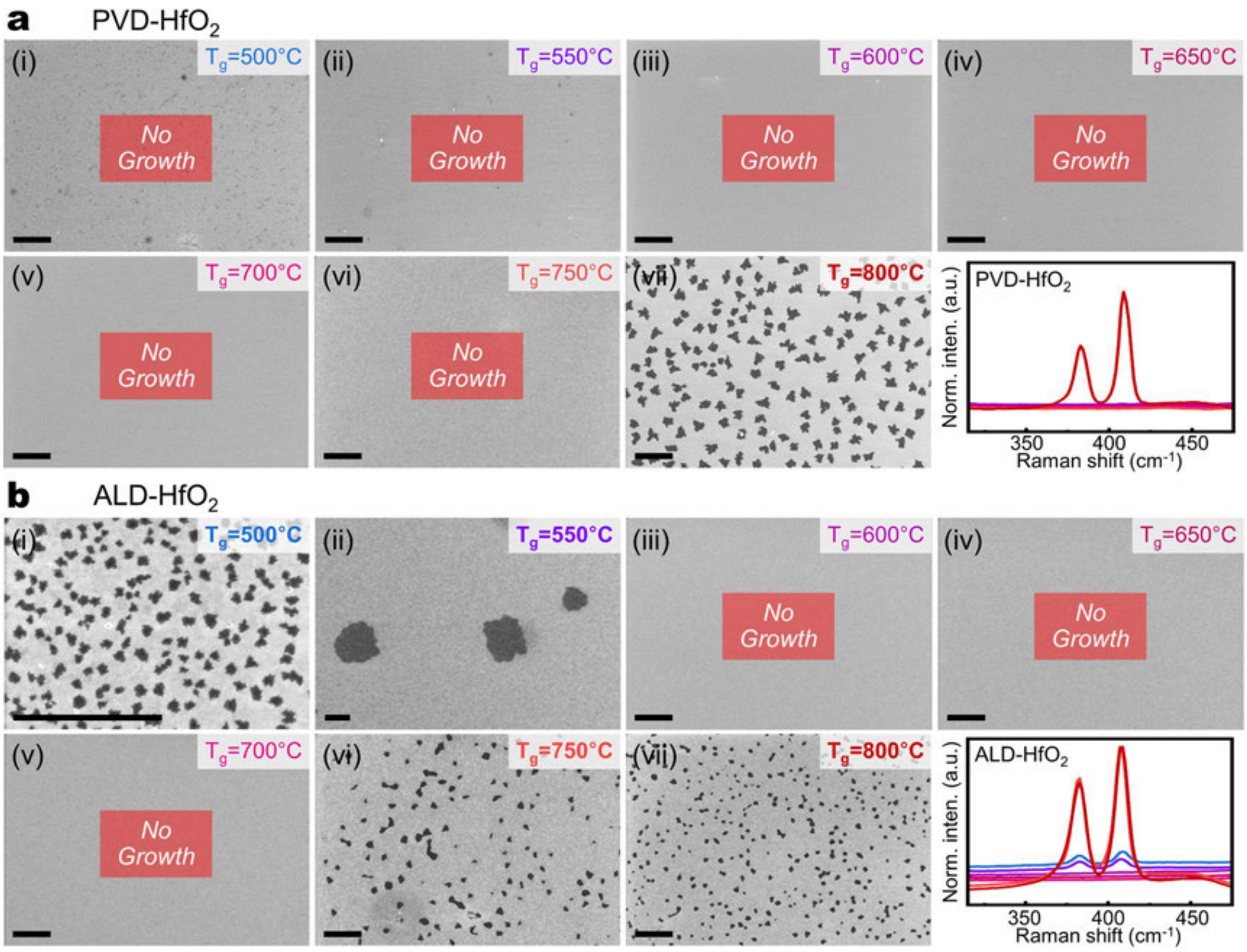

Fig. R8 | 2D MoS_2 MOCVD growth on HfO_2. a,b, SEM images and Raman spectra of MoS_2 grown on PVD-HfO_2 (a) and ALD-HfO_2 (b) with MOCVD temperature (500-800 °C). Scale bars, 3 μm.

[Action in Manuscript]

i) We have added **Extended Data Fig. 4 (p. 26)** to compare nucleation behavior across different oxide barriers and to verify that nucleation suppression is enabled by HfO_2 under EF-SCN conditions.

(p. 26) Extended Data Fig. 4 | 2D MoS_2 growth behaviors with a-HfO_2, a-SiO_2, and a-Al_2O_3 barrier. a,b, SEM images (a) and Raman spectra (b) of MoS_2 grown on various substrates (sapphire, a-Al_2O_3, a-SiO_2, PVD a-HfO_2, and ALD a-HfO_2) with MOCVD temperatures (500-800 °C). Scale bars, 3 μm.

ii) We have added **Supplementary Note 1 (SI p. 22)** to clarify how the validity of EF-SCN depends on barrier material types and deposition method.

(SI p. 22) "These valid conditions for EF-SCN can be expanded by using other types of barriers instead of HfO_2. As shown in Extended Data Fig. 5, when a ZrO_2 barrier with a lower crystallization temperature than HfO_2 is used, EF-SCN can be achieved even below 500 °C[23-27]. In addition, as shown by PVD-HfO_2 and ALD-HfO_2 in Extended Data Fig. 4a, the nucleation suppression effect can vary depending on the deposition method of the etching-flux barrier. ~"

(5) Beyond HfO_2, it would be valuable to examine whether other oxide barriers, such as Al_2O_3 or SiO_2, can induce nucleation control effects similar to those observed in EF-SCN. If differences arise, a comparative discussion on how variations in material properties, such as melting point, bonding energy, and surface defect characteristics, affect the nucleation behavior is suggested.

[Author reply]

Investigating which barrier materials enable effective single-centered nucleation is a critically important task for developing the EF-SCN system. To this end, we first examined the growth behavior of MoS_2-$MoSe_2$ lateral superlattices using amorphous HfO_2 (a-HfO_2), amorphous SiO_2 (a-SiO_2), and amorphous Al_2O_3 (a-Al_2O_3) as barrier materials, as discussed in Supplementary Table 1. As shown in the SEM image in Fig. R9a, when a-HfO_2 is used as the barrier, TMD growth is suppressed on the a-HfO_2 surface and nucleation is induced at the center of the growth region, following the typical EF-SCN behavior.

Material	Crystallinity	Annealing	Transition		Ref
Sapphire	Crystalline	1100~1500°C (N_2 & N_2+H_2 vacuum)	**Decomposition (>1100°C)**	**>1100°C**	(1)
	Crystalline	1100~1500°C (He & H_2 vacuum)	**Decomposition (>1300°C)**		(2), (3)
a-Al_2O_3	Amorphous (Sputter)	600~1200°C (Air)	**Crystallization (~900°C)**	**~900°C**	(4)
	Amorphous (ALD)	1000~1200°C (N_2 vacuum)	**Decomposition (~1150°C)**		(5)
	Amorphous (ALD)	800~1000°C (Vacuum)	**Decomposition (~900°C)**		(6)
a-SiO_2	Amorphous (Thermal)	-	**Decomposition (>800°C)**	**>800°C**	(7)
	Amorphous (Thermal)	1050~1100°C (Vacuum)	**Void formation (~1050°C)**		(8)
	Amorphous (Thermal)	760~850°C (Vacuum)	**Decomposition (>800°C)**		(9)
a-HfO_2	Amorphous (Plasma ALD)	400~600°C (N_2 vacuum)	**Decomposition (~500°C)**	**~500°C**	(10)
	Amorphous (Sputter)	200~600°C (Vacuum)	**Roughening (~600°C)**		(11)

Supplementary Table 1

In contrast, when a-SiO_2 is used as the barrier (Fig. R9b), MoS_2 exhibits low coverage on a-SiO_2 and high coverage on sapphire. This behavior is similar to previously reported confined growth results, suggesting that a-SiO_2 serves as a barrier due to differences in TMD binding energy rather than through an etching-flux effect[13]. Interestingly, when a-Al_2O_3 is employed as the barrier (Fig. R9c), full film growth is observed on a-Al_2O_3, while growth on sapphire initiates from the edge of the a-Al_2O_3, even at a high growth temperature of 750 °C. As indicated in Supplementary Table 1, both a-SiO_2 and a-Al_2O_3 have higher crystallization temperatures than a-HfO_2, implying that oxygen release from these materials is negligible. Therefore, the observed selective growth in Fig. R9b and R9c is attributed primarily to differences in TMD binding energy rather than the etching-flux effects.

In addition to the materials suggested by the reviewer, we further confirm that ZrO_2, which has a lower crystallization temperature than HfO_2, can serve as an effective barrier for EF-SCN even at temperatures below 500 °C (Fig. R7).

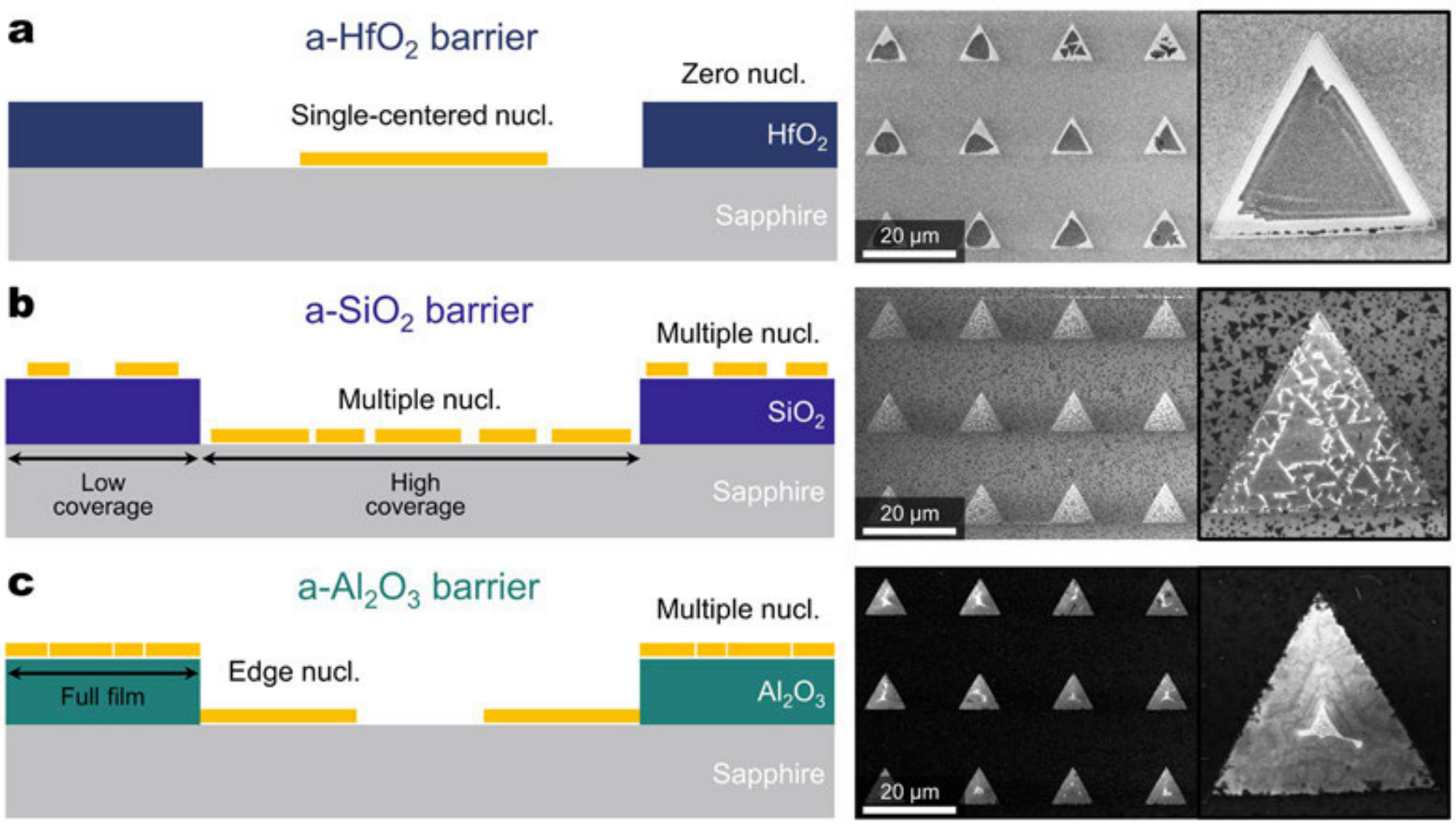

Fig. R9 | Selective growth behaviors in EF-SCN system with different barriers. a-c, Schematic and SEM images of MoS_2-$MoSe_2$ lateral superlattice growth with HfO_2 barrier (**a**), SiO_2 barrier (**b**), and Al_2O_3 barrier (**c**).

[Action in Manuscript]

i) We have added **Extended Data Fig. 4 (p. 26)** to compare nucleation behavior across different oxide barriers and to demonstrate that only HfO_2 enables deterministic nucleation of EF-SCN.

(p. 26) Extended Data Fig. 4 | 2D MoS_2 growth behaviors with a-HfO_2, a-SiO_2, and a-Al_2O_3 barrier. c-e, Schematic and SEM images of MoS_2-$MoSe_2$ lateral superlattice growth with a-HfO_2 barrier (c), a-SiO_2 barrier (d), and a-Al_2O_3 barrier (e).

ii) We have revised **manuscript (p. 6)** to clarify the mechanistic difference in nucleation behavior among oxide barriers, explaining why only HfO_2 enables deterministic nucleation.

(p. 6) "In addition, Extended Data Fig. 4c-e present the growth behavior of 2D TMD at 750 °C when a-HfO_2, a-SiO_2, and a-Al_2O_3 are used as barrier materials. Unlike a-HfO_2, where centered nucleation is induced by the etching-flux released from the barrier, a-SiO_2 exhibits random multiple nucleation within the growth region, while a-Al_2O_3 shows distinct edge nucleation. This indicates that a-SiO_2 and a-Al_2O_3 do not release sufficient oxygen during 2D TMD growth to induce an etching effect, whereas a-HfO_2 can suppress MoS_2 growth through etching-flux and enable spatially deterministic nucleation. Importantly, compared with previous reports of MoS_2 growth on HfO_2 surfaces, the MOCVD environment in EF-SCN was found to be more sensitive to oxygen-induced etching, which can be attributed to the use of non-oxide precursors[8,32]. More detailed discussions on the oxygen-sensitive growth conditions under which HfO_2 serves as a valid etching-flux barrier are provided in Supplementary Note 1, along with Supplementary Figs. 4, 5, and Supplementary Table 2."

(6) In Extended Data Fig. 10e, the epitaxial growth direction is parallel to the triangular barrier pattern direction. If the epitaxial growth direction does not coincide with the pattern orientation, it would be informative to discuss whether MoS_2 flakes follow the imposed pattern or retain their intrinsic epitaxial orientation, and to include the expected or observed growth behavior under such misalignment conditions.

[Author reply]

In the previous manuscript, the triangular barrier patterns were fabricated parallel to the epitaxial growth direction of MoS_2, which made it difficult to properly assess the effect of pattern orientation on single-crystal growth behavior. In response to this comment, we investigated the influence of barrier pattern orientation, as crystallographic alignment of TMD is critical for heterostructure integration. Fig. R10 presents the growth behavior when the orientation of the barrier pattern deviates from the epitaxial growth direction of MoS_2. Fig. R10a and R10b show a schematic and an SEM image of the case in which the barrier pattern is aligned parallel to the MoS_2 epitaxy direction, exhibiting clean edges of single-crystal MoS_2 flakes.

In contrast, Fig. R10c and R10d show the case with a 15° misalignment between the epitaxy direction and the barrier pattern, where the flakes are etched in a uniform direction, resulting in rough edges. Similarly, Fig. R10e and R10f (60° misalignment) also exhibit flakes with rough edges consistent with the same etching tendency. These results indicate that the orientation of MoS_2 flakes is not dictated by the geometry of the HfO_2 barrier pattern but rather follows the intrinsic epitaxial growth direction. Moreover, the consistent etching-induced faceting direction across flakes further supports their single-crystallinity.

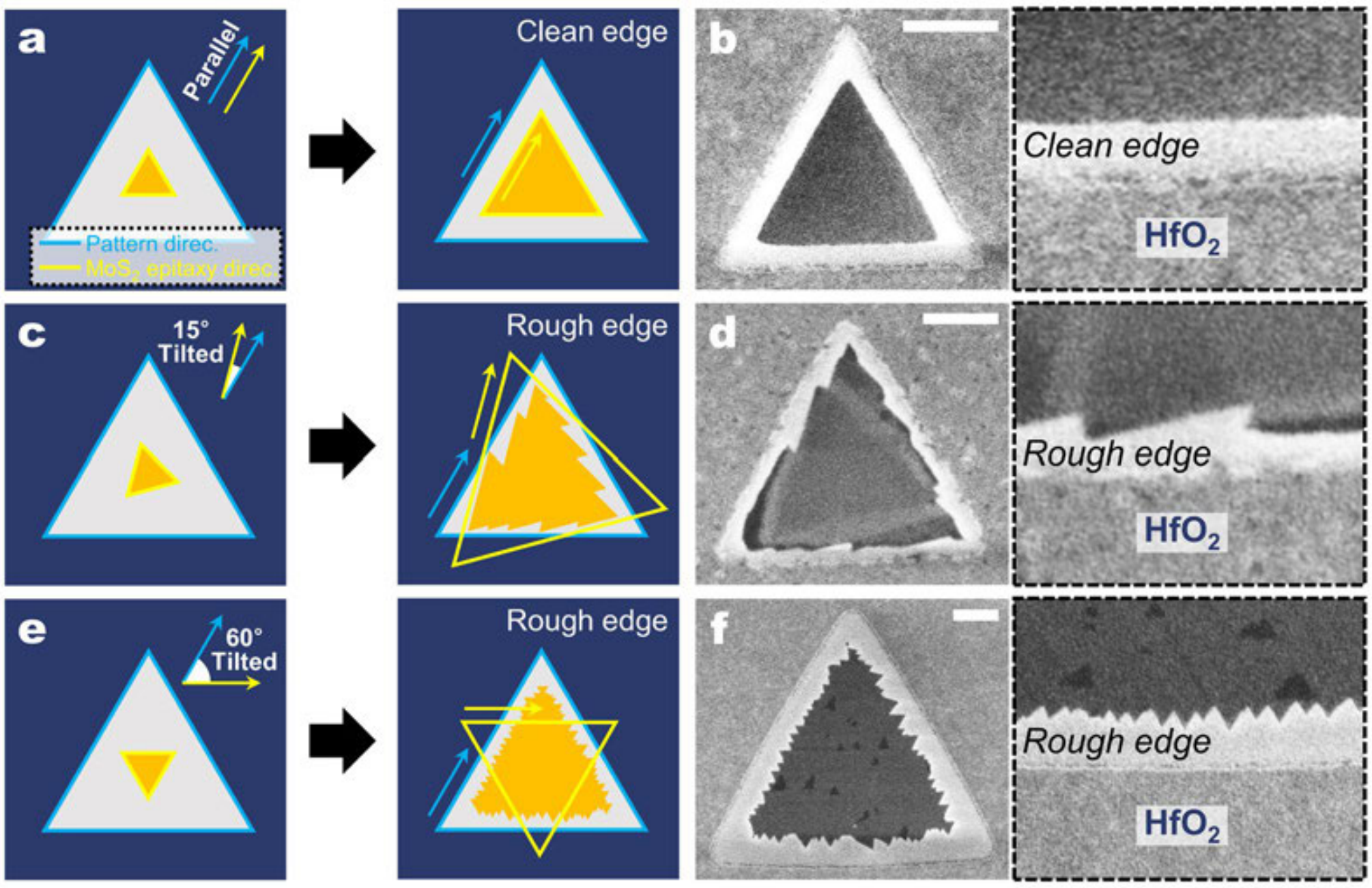

Fig. R10 | Discussion on the relationship between MoS_2 epitaxial growth direction and HfO_2 barrier pattern direction. a,**b**, Schematic (**a**) and SEM images (**b**) of MoS_2 growth when the epitaxial growth direction and the barrier pattern direction are parallel. **c-f**, Schematic and SEM images of MoS_2 growth at misalignment angles of 15° (**c**, **d**) and 60° (**e**, **f**) between the epitaxial growth direction and the barrier pattern direction. Scale bars, 1 μm.

[Action in Manuscript]

i) We have added **Supplementary Fig. 3 (SI p. 4)** to examine the effect of alignment between the epitaxial growth direction and the barrier pattern.

(SI p. 4) Supplementary Fig. 3 | Relationship between MoS_2 epitaxial growth direction and HfO_2 barrier pattern direction. a,b, Schematic (a) and SEM images (b) of MoS_2 growth when the epitaxial growth direction and the barrier pattern direction are parallel. c-f, Schematic and SEM images of MoS_2 growth at misalignment angles of 15° (c, d) and 60° (e, f) between the epitaxial growth direction and the barrier pattern direction. Scale bars, 1 μm.

ii) We have revised **manuscript (p. 5)** to clarify that the crystallographic orientation of MoS_2 is governed by the epitaxial growth direction rather than the barrier pattern geometry.

(p. 5) "Furthermore, when the barrier pattern direction is misaligned with the epitaxial growth direction, rough edges aligned with the intrinsic crystallographic orientation are observed near the barrier, instead of the clean triangular flake typical of EF-SCN (Supplementary Fig. 3). These results indicate that the orientation of triangular single-crystal MoS_2 in EF-SCN is determined by the epitaxial growth direction rather than the barrier pattern geometry. ~"

(7) In the regime where many nucleation events occur due to increased pattern size, analyzing the evolution of nucleation distributions as a function of pattern size and growth conditions is expected to be particularly informative. The authors are encouraged to experimentally explore this regime and to include a discussion on the observed trends and underlying mechanisms.

[Author reply]

Our previous manuscript has a limitation in that EF-SCN was discussed based on only a relatively small number of flakes. Therefore, this comment provides an important opportunity to address and improve this aspect. Fig. R11 demonstrates the EF-SCN behavior in large-size line patterns. SEM images were used to analyze the selective growth of MoS_2 in 20 μm and 40 μm line patterns under three different coverage conditions (85.7%, 67.6%, and 31.9%) (Fig. R11a). As the growth coverage decreases, the saturated coverage within the pattern is reduced, while the critical length increases significantly (Fig. R11b and R11c).

Notably, in the 20 μm patterns, zero nucleation occurs inside the pattern under the lowest coverage condition. This suggests that oxygen released from HfO_2 can influence regions up to 10 μm. Based on the nucleation distribution in large-size patterns, the effective pattern size for nucleation control by the etching-flux effect is on the order of 20 μm. Furthermore, it is expected that larger single-crystals can be achieved by increasing the lateral growth rate of MoS_2.

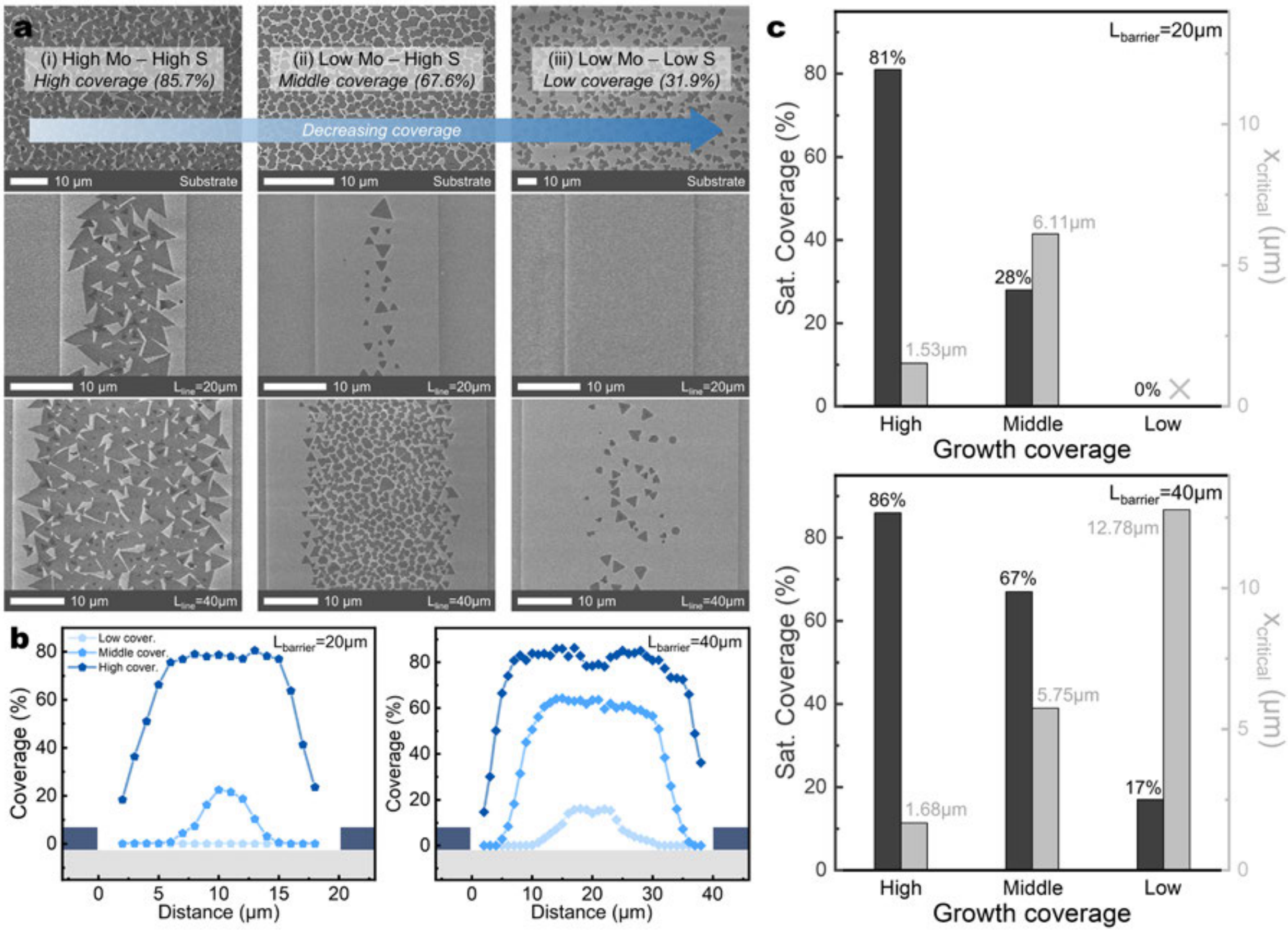

Fig. R11 | MoS_2 growth behaviors in a large-size pattern. a,**b**, SEM images (**a**) and coverage graphs (**b**) of MoS_2 growth in linear etching-flux barrier pattern with different growth conditions. **c**, Bar graphs of saturated growth coverage (dark gray) and critical length (light gray) of the results in (**a**).

[Action in Manuscript]

i) We have added **Supplementary Fig. 13 (SI p. 14)** to demonstrate the growth behavior in large-size linear patterns under varying growth conditions.

(SI p. 14) Supplementary Fig. 13 | MoS_2 growth behaviors in large-size pattern. a,b, SEM images (a) and coverage graphs (b) of MoS_2 growth in linear etching-flux barrier pattern with different growth conditions. c, Bar graphs of saturated growth coverage (dark gray) and critical length (light gray) of the results in (a).

ii) We have added **Supplementary Note 4 (SI p. 28)** to explain the EF-SCN behavior in large-size linear patterns.

(SI p. 28) "Supplementary Fig. 13 presents the growth behavior observed under varying growth coverage conditions in linear etching-flux barrier patterns with line widths of 20 and 40 μm. Across both pattern widths, a decrease in growth coverage is accompanied by a progressive narrowing of the NAR, an increase in critical length, and a reduction in intra-pattern saturation coverage, consistent with trends established in small-size triangular pattern geometries. ~"

3. Point-by-Point Responses to Reviewer #2, #3 Comments

The authors present a strategy utilizing "etching-flux mediation" to spatially program the nucleation of 2D semiconductors at the center of patterned trenches. The concept of "geometric confinement" for wafer-scale single-domain growth was established by Kim et al. in 2023. That work solved the fundamental kinetic challenge of merging nuclei. In 2024, they also demonstrated the growth of single-crystal TMDs at 385 °C, enabling Back-End-of-Line compatible Monolithic 3D integration. In contrast, the current work relies on high temperature growth (700 °C−800 °C) to activate the etching mechanism. In the context of the semiconductor roadmap, abandoning the low temperature progress achieved in 2024 to return to high temperature processing—merely to achieve "center nucleation"—seems to diverge from the industry's critical trajectory toward low-thermal-budget integration. Therefore, the scientific significance of this work should be clarified further.

[Author reply]

We sincerely thank the reviewers for their insightful comments regarding the scientific significance and technological relevance of the EF-SCN approach within the broader context of TMD selective growth and semiconductor integration. This revision provides an important opportunity to further clarify the distinct scientific contribution and practical implications of this work.

As noted by the reviewers, previous studies demonstrated grain-boundary-free TMD growth using confinement structures (2023) and subsequently realized CFET integration through low-temperature TMD growth at 385 °C (2024). These studies established important progress in confinement-assisted single-crystal growth and low-thermal-budget integration.

In contrast, the present work addresses a fundamentally different scientific challenge: whether nucleation in 2D TMD growth can be transformed from a stochastic process into a deterministic and spatially programmable process through spatial control of the local growth environment during synthesis. Importantly, the scientific objective of the present work is not simply to realize center nucleation itself, but to establish deterministic and spatially programmable nucleation as a distinct growth regime for 2D TMD systems.

The EF-SCN mechanism introduces an additional degree of freedom beyond conventional geometric confinement by utilizing etching-flux-mediated growth kinetics to create a spatially biased nucleation landscape with a substantially enhanced nucleation probability at the center of the growth region. Furthermore, this spatially programmed nucleation behavior enables subsequent extension toward aligned lateral heterostructures and enlarged single-crystal domains, which are difficult to achieve using conventional confinement-based growth approaches.

We understand the reviewer's concern regarding process compatibility, particularly because the original manuscript primarily presented growth results at 750 °C. However, the EF-SCN mechanism itself is not fundamentally limited by temperature. Rather, the originally presented process conditions were selected to most clearly demonstrate the underlying etching-flux-mediated nucleation mechanism. In response to the reviewer's insightful industry-oriented perspective regarding thermal-budget compatibility, we conducted additional experiments during this revision to engineer the EF-SCN growth process toward lower-temperature operation. These new results demonstrate that deterministic single-crystal growth through EF-SCN can also be achieved under substantially reduced thermal budgets while preserving its nucleation-programming capability.

		Seed growth [29-32]	Confined growth [13,15]		EF-SCN [Our work]	
	Selectivity origin	Surface difference (Nucleation promoter)	Surface difference (Nucleation inhibitor)		Etching-flux	
A	TMD growth temperature (°C)	650-750	750 [13]	385 [15]	750	500 [Fig. R7]
A	TMD growth method	Powder CVD or Sulfurization	Powder CVD		MOCVD	
A	Mobility ($cm^2V^{-1}s^{-1}$)	5-50	62 [13]	56 [15]	117 [Fig. R12]	
B	**Nucleation initiation**	**Not spatially deterministic**	**Not spatially deterministic**		**Deterministic** at center	
B	**Effective single-crystal size (μm)**	**Limited single domain**	**1-2** [13]	**0.2-0.5** [15]	**7 (Tri.)** [Fig. R7]	**10 (Line)** [Fig. R12]
B	**Orientation alignment**	**Not controlled**	**Not controlled**		**Possible** [Fig. R14, R21]	
B	**Advanced applications** beyond basic FET	-	**Valleytronics device** [13] with single-crystal *vertical heterostructure*		**Self-aligned ALD contact FETs** [Fig. R14] with *aligned lateral heterostructure*	
B			**CFET** [15] with single-crystal *3D integration*		**Single-crystal multiple FETs** [Fig. R12] with *large-size single-crystal*	

Table R2 | Benchmark table of 2D monolayer MoS_2 area-selective growth. Comparison between seed growth[29-32], confined growth[13,15], and EF-SCN method.

To systematically clarify the scientific distinctions and technological advantages of EF-SCN relative to other selective growth strategies[13,15,29-32], we constructed the benchmark comparison summarized in Table R2. The table compares representative monolayer MoS_2 area-selective growth methods, including seed growth, confined growth, and EF-SCN, using consistent metrics for process compatibility, device performance, and achievable application scope. Through this comparison, we aim to clarify that EF-SCN extends beyond simple nucleation-site positioning and enables capabilities that are difficult to achieve using conventional selective-growth approaches.

A. Process compatibility and device-level validation

Most reported seed-growth approaches employ high growth temperatures in the range of 650-750 °C, whereas confined growth based on edge nucleation has demonstrated effective single-crystal growth at a low temperature of 385 °C. As pointed out by the reviewers, the EF-SCN results in our previous report required relatively high temperatures (~750 °C) to generate sufficient etching-flux.

To address this concern, we performed additional experiments using a ZrO_2 barrier and demonstrated deterministic single-crystal MoS_2 growth at a reduced temperature of 500 °C (Fig. R7). This result confirms that EF-SCN is not intrinsically tied to the original 750 °C HfO_2 barrier, but can be extended to lower-temperature regimes through rational barrier engineering. The exploratory multi-stacked barrier result further suggests room for future thermal-budget reduction. Importantly, these additions clarify that the significance of EF-SCN lies not merely in center nucleation at a specific temperature, but in establishing etching-flux-mediated deterministic nucleation control as a programmable growth strategy for 2D TMD.

In addition, while most previously reported 2D TMD area-selective growth studies relied on powder CVD processes, EF-SCN is implemented using MOCVD, which provides a well-controlled and scalable growth environment. To further validate the process compatibility and robustness of EF-SCN, we additionally achieve high-density single-crystal growth (Fig. R15), establish a viable process window for deterministic single-crystal growth (Fig. R16), and demonstrate large-area uniform single-crystal growth, including growth on amorphous SiO_2 substrates (Fig. R17).

In terms of device performance, single-crystal MoS_2 obtained by seed growth and confined growth typically exhibits field-effect mobilities of approximately 50-60 $cm^2V^{-1}s^{-1}$. The EF-SCN method likewise enables high-quality single-crystal growth, yielding a maximum field-effect mobility of 117 $cm^2V^{-1}s^{-1}$ extracted from FET arrays (Fig. R12).

B. Advanced single-crystal device capabilities enabled by EF-SCN

Conventional 2D TMD area-selective growth methods generally exhibit distributed nucleation behavior across the growth region, limiting the achievable single-crystal size to approximately 1 μm. In contrast, EF-SCN utilizes etching-flux-mediated growth kinetics to strongly localize nucleation near the center of the growth region, enabling enlarged single-crystal growth reaching 7 μm in triangular patterns and 10 μm in linear patterns (Fig. R7 and R12).

This single-crystal scaling capability enables integration of multiple FETs within a single-crystal channel, rather than forming an individual FET inside each isolated growth region. The resulting multiple-FET structures exhibited a 65% reduction in mobility variation, with the RSD decreasing from 21.8% for conventional single-FET arrays to 7.8% (Fig. R12), suggesting the potential of EF-SCN for low-variation and high-density device architectures.

In addition, conventional seed-growth and confined-growth strategies do not inherently provide deterministic control over both nucleation position and crystallographic orientation simultaneously. In contrast, EF-SCN enables simultaneous control of nucleation position and orientation, thereby enabling advanced single-crystal architectures based on spatially programmed in-plane growth.

Using aligned 2D MoS_2-$MoSe_2$ lateral heterostructures, we further demonstrate self-aligned ALD contact devices, providing a platform for spatially controlled strain engineering, doping modulation, and advanced contact processing in 2D TMD channels (Fig. R14).

[Action in Manuscript]

i) We have added **Supplementary Table 4 (SI p. 20)** to benchmark the EF-SCN method against conventional seed growth and confined growth approaches for 2D monolayer MoS_2 ASG.

(SI p. 20) Supplementary Table 4 | Benchmark table of 2D monolayer MoS_2 ASG. Comparison between seed growth[16-19], confined growth[20,21], and EF-SCN method.

ii) We have introduced **Supplementary Note 6 (SI p. 34)** to discuss the novelty and advantages of EF-SCN compared with conventional 2D TMD ASG methods, particularly in terms of process compatibility and advanced single-crystal device implementation.

(SI p. 34) "Conventional 2D TMD ASG has been explored primarily through two approaches: seed growth[16-19] and confined growth[20,21]. In this note, we elaborate on the novelty and advantages of EF-SCN in terms of process compatibility and advanced single-crystal device implementation, based on the benchmarking presented in Supplementary Table 4. ~"

(1) What is the specific, quantifiable advantage of "Center Nucleation" over the low temperature "Edge Nucleation" strategy? If edge-nucleated crystals are already single-crystalline and BEOL-compatible, why should the industry adopt a high temperature (>700 °C), oxygen-mediated process?

[Author reply]

Clarifying the advantages of the EF-SCN approach over conventional selective growth methods is the most critical task of this revision. To address this comment, we performed additional experiments to demonstrate (i) Low-temperature extensibility of EF-SCN through barrier engineering and (ii) Exclusive demonstration of advanced single-crystal devices.

(i) Low-temperature extensibility of EF-SCN through barrier engineering

The key principle of EF-SCN lies in utilizing the intrinsic etching-flux released from the barrier to deterministically localize the nucleation site at the center of the growth region, thereby enabling effective single-crystal growth of 2D TMD. In our previous manuscript, a high growth temperature (750 °C) was primarily required to induce sufficient oxygen release from the HfO_2 barrier. Therefore, we expected that EF-SCN could be extended to lower temperatures by employing alternative barrier materials capable of releasing oxygen at reduced temperatures.

[redacted]

[redacted]

[redacted]

[redacted]

(ii) Exclusive demonstration of advanced single-crystal devices

BEOL compatibility of channel growth in semiconductor integration processes is clearly a critical issue. On the other hand, major chip makers such as Samsung and TSMC continue to regard high-temperature FEOL channel-first integration as an important strategy and have reported the realization of silicon- and MoS_2-based GAA-FETs at 600-800 °C temperature[33-37]. The rationale for adopting channel-first integration lies in its ability to achieve device architectures and performance that are difficult to realize with low-temperature BEOL-compatible processes. In this context, EF-SCN presents a unique and differentiated approach that enables advanced single-crystal devices that are difficult to achieve with conventional 2D TMD selective growth methods.

A. Single-crystal multiple FETs with large-size single-crystal growth

In the 2D TMD confined growth method, the nucleation probability is identical across all terrace sites within the growth region. As a result, as the pattern size increases, undesired nucleation cannot be suppressed, leading to a significant reduction in single-crystal yield[13]. In particular, for low-temperature edge growth, nucleation probability is also uniform along all edge sites, and the reduced lateral growth rate at low temperatures further limits single-crystal growth to small pattern sizes of the 500 nm scale[15]. Therefore, confined growth inherently suffers from a fundamental limitation in achievable single-crystal size.

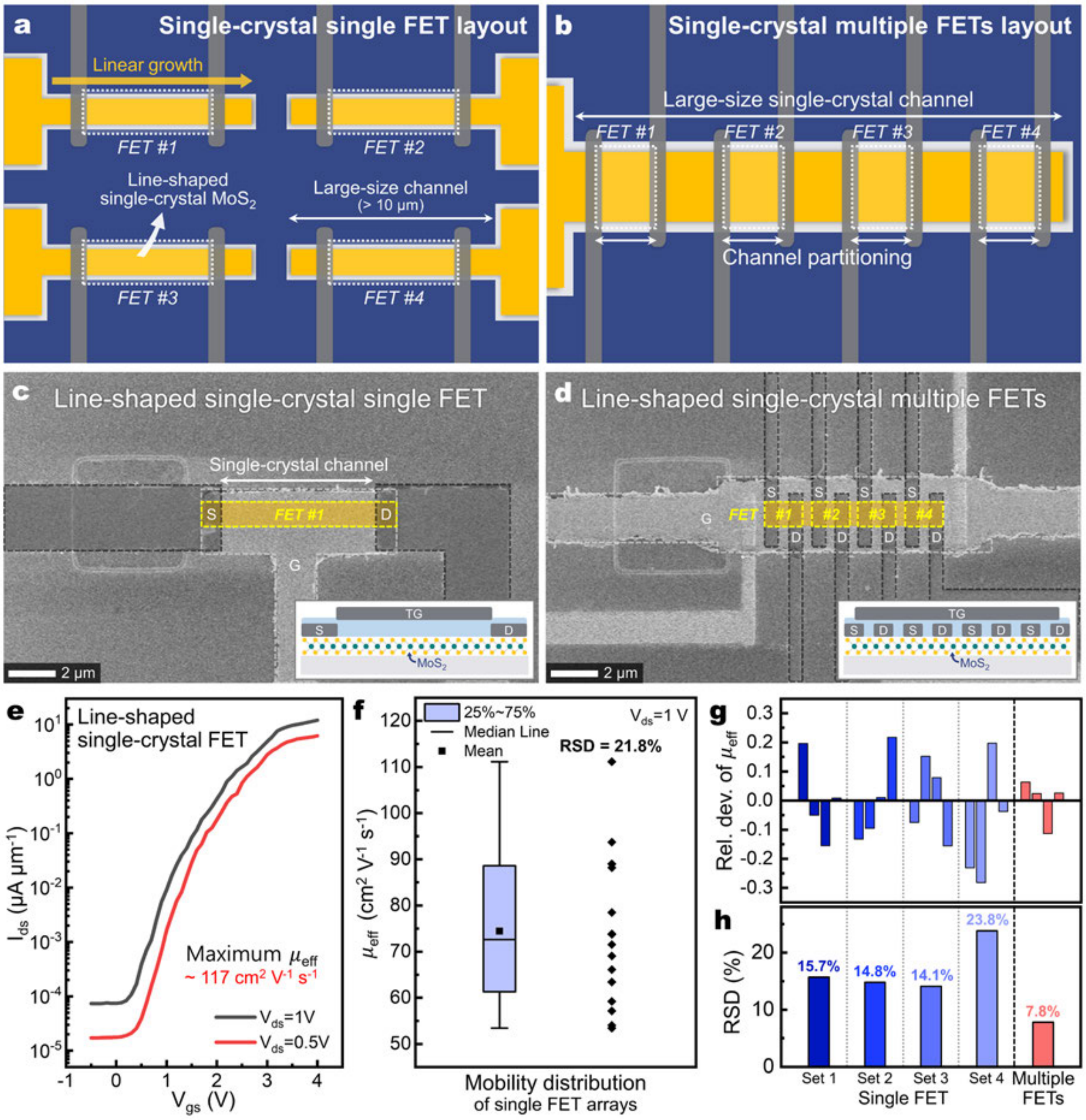

Fig. R12 | Device performance of line-shaped single-crystal single FET and multiple FETs by nucleation-growth decoupling method. a,**b**, Schematic illustrating single-crystal single FET (**a**) and single-crystal multiple FETs (**b**) layouts. **c**,**d**, SEM images of single-crystal MoS_2 single FET (**c**) and multiple FETs (**d**). **e**, Transfer curves of line-shaped single-crystal single FET. **f**, Statistical distribution and box plot of field-effect mobility for single FET arrays. **g**,**h**, Relative mobility deviation (**g**) and relative standard deviation (**h**) of single FET arrays and multiple FETs.

Scaling of single-crystal size, which is difficult to achieve with conventional selective growth methods, is expected to play a critical role in the practical utilization of 2D TMD devices, particularly in terms of device-to-device variation. Rather than fabricating a single device within a single channel, as in the typical one-pocket-one-channel system, integrating multiple FETs within a large-size single-crystal based on a one-pocket-multi-channel concept is expected to reduce performance variation. From this perspective, single-crystal scaling directly impacts device integration, and large-area single-crystals are expected to be essential for the industrial adoption of 2D TMD.

EF-SCN enables deterministic nucleation at the center of the growth region while effectively suppressing undesired nucleation, thereby allowing large-size single-crystal growth, including triangular flakes of 7 μm and linear flakes of 10 μm, which are not achievable with conventional confined growth. Leveraging this advantage, we demonstrate the integration of multiple FETs within a single-crystal MoS_2 channel.

Fig. R12a and R12b illustrate the concepts of typical single-crystal single FET arrays and single-crystal multiple FETs with large-size line-shaped MoS_2 channel. As shown in the SEM images in Fig. R12c and R12d, single FET arrays were fabricated within individual channels (Fig. R12c), while multiple FETs were integrated within a single channel (Fig. R12d). In the case of the single-crystal single FET, an excellent field-effect mobility of 117 $cm^2V^{-1}s^{-1}$ was observed, as shown in Fig. R12e, exceeding the previously reported values by confined growth (56-62 $cm^2V^{-1}s^{-1}$)[13,15]. Statistical analysis across 16 single FET arrays yields a mean mobility of 74 $cm^2V^{-1}s^{-1}$ and mobility RSD of 21.8% (Fig. R12f), comparable to performance variations of confined growth[13].

Importantly, the single-crystal multiple FETs exhibit improved uniformity: RSD values for the four sets of single FETs ranged from 14.1 to 23.8% (21.8% overall), while the multiple FETs yielded a substantially reduced RSD of 7.8% (Fig. R12g and R12h), corresponding to a 65% improvement in mobility uniformity. This finding indicates that integrating multiple devices within a single channel is a more effective strategy for minimizing device performance variation than the typical single FET in one channel approach.

Collectively, these results highlight the potential of EF-SCN's single-crystal scaling capability for realizing low-variation, high-density 2D FET arrays within an enlarged single-crystal channel. As illustrated in Fig. R13, this capability provides an architectural basis for extending single-crystal 2D FET integration from individual devices toward standard-cell-level layouts, where multiple transistors can share a common single-crystal foundation. Although logic-circuit operation is beyond the scope of the present work, the experimentally demonstrated multiple FETs integration within a single-crystal MoS_2 channel provides an experimental basis for exploring future scaling from 2D FET arrays toward standard-cell and block-level logic integration. In addition, because EF-SCN enables the formation of elongated single-crystal channels that can be partitioned into multiple FETs, this strategy may also be relevant to string-like transistor architectures, such as NAND flash strings, where multiple transistor elements are arranged along an extended channel direction.

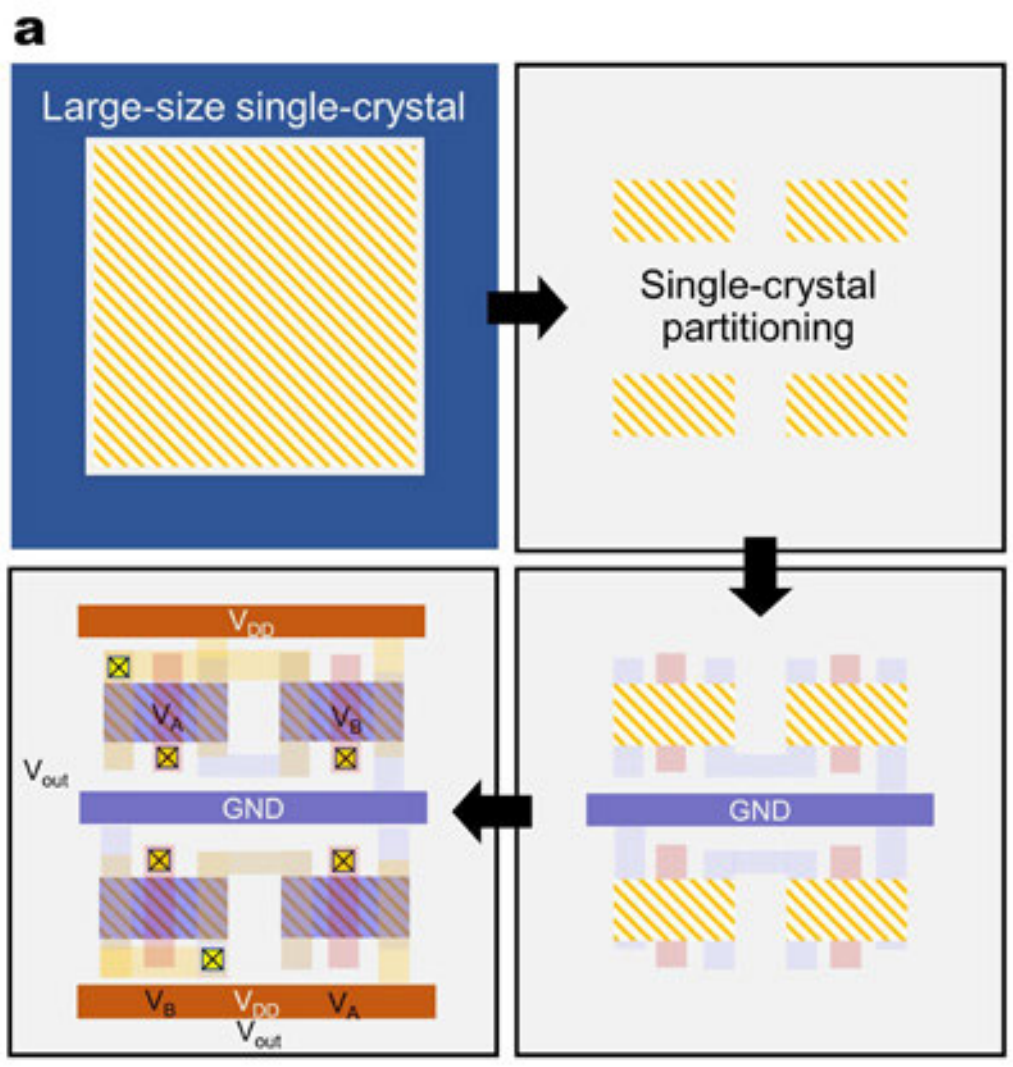

Fig. R13 | Large-size single-crystal partitioning for multiple FETs integration and logic implementation. a, Schematic illustrating a large-size single-crystal domain and its division into multiple channels via single-crystal partitioning, followed by the formation of multiple FETs and their interconnection for logic operation.

B. Self-aligned ALD contact devices with self-aligned lateral heterostructure

Conventional 2D TMD selective growth methods enable the formation of a single-crystal within a predefined region; however, there is no established approach to control the lattice orientation. In contrast, the EF-SCN approach allows precise control over the nucleation initiation site as well as crystallographic alignment via epitaxy, enabling functionalities beyond those of a homogeneous material, including strain, doping, and contact engineering in the vicinity of a single-crystal channel. While post-treatment processing can be used to introduce such functionalities, 2D TMD is highly sensitive to external forces, and these processes may induce unintended damage to the channel region. In contrast, EF-SCN enables the formation of aligned 2D lateral heterostructures without channel damage through gas-phase precursor control during the MOCVD process, providing a clear technological distinction and novelty.

Fig. R14 and Main Fig. 4 demonstrate self-aligned ALD contact devices enabled by self-aligned lateral heterostructures formed via EF-SCN. As shown in the SEM image in Fig. R14a, although slight misalignment in crystal orientation is observed due to the low epitaxial growth temperature, large-size single-crystal MoS_2-$MoSe_2$ lateral heterostructures exceeding 5 µm can be grown with overall alignment within the patterned region. Such spatially programmed growth enables new contact engineering strategies based on strain modulation in the 2D TMD channel. In MoS_2-$MoSe_2$ lateral heterostructures, strain induced by lattice mismatch enhances precursor adsorption on the $MoSe_2$ region during the ALD process[38]. Therefore, by applying Ru AS-ALD to aligned MoS_2-$MoSe_2$ structures, a self-aligned ALD contact 2D TMD device can be realized without additional processing steps (Fig. R14b-e). These results demonstrate that EF-SCN-based growth provides a fundamentally new pathway for engineering the channel surroundings, which is not achievable with conventional selective growth approaches.

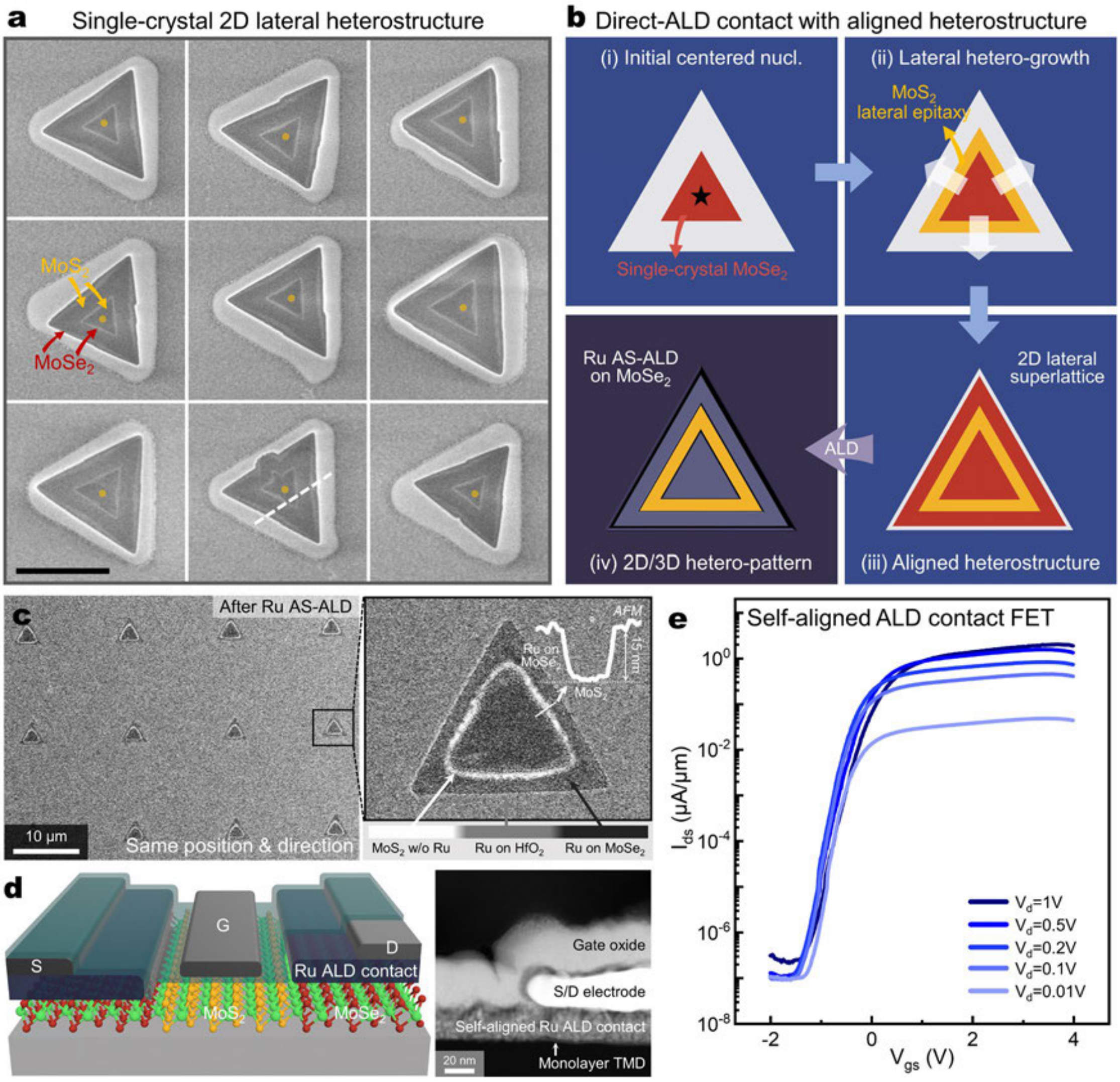

Fig. R14 | Self-aligned ALD contact device with self-aligned lateral heterostructure by EF-SCN. a, SEM images of aligned single-crystal MoS_2-$MoSe_2$ lateral superlattice. Scale bars, 5 µm. **b-d**, Schematic, SEM images, and HAADF-STEM image of Ru AS-ALD on aligned single-crystal 2D heterostructures. **e**, Transfer curve of self-aligned ALD contact FET.

[Action in Manuscript]

i) We have revised **Fig. 4 (p. 22)** and added **Extended Data Fig. 5,9,10 (p. 27,31,32)** to demonstrate the low-temperature applicability of EF-SCN, the realization of single-crystal multiple FETs based on nucleation-growth decoupling, and the implementation of self-aligned ALD contact FETs.

(p. 22) Fig. 4 | 2D TMD single-crystal architectures based on EF-SCN method. a, Schematic of nucleation-growth decoupling strategy for line-shaped single-crystal 2D MoS_2 growth. b, SEM image of line-shaped single-crystal MoS_2 single FET. c, Transfer curve of the line-shaped single FET. d, Statistical distribution and box plot of field-effect mobility for single FET arrays. e-g, Schematic (e, g) and SEM images (f) of Ru AS-ALD on aligned 2D MoS_2-$MoSe_2$ lateral heterostructure for self-aligned ALD contact. The inset graph shows the AFM height profile along the white line. h, i, HAADF-STEM images (h) and transfer curve (i) of self-aligned ALD contact FET.

(p. 27) Extended Data Fig. 5 | Low-temperature EF-SCN and high-density single-crystal growth. a,b, Schematic and SEM images showing single-crystal MoS_2 EF-SCN with high T_c barrier (HfO_2) on sapphire at 750 °C (a) and low T_c barrier (ZrO_2) on sapphire at 500 °C (b). Scale bars, 1 μm.

(p. 31) Extended Data Fig. 9 | Nucleation-growth decoupling strategy and single-crystal multiple FETs. a,b, SEM images showing line-shaped single-crystal MoS_2 growth via nucleation-growth decoupling strategy in initial stage (a) and fully growth stage (b). c,d, Schematic (c) and NCM phase mapping image by AFM (d) of stripe 2D MoS_2-$MoSe_2$ lateral heterostructure. e,f, Schematic illustrating single-crystal single FET (e) and single-crystal multiple FETs (f) layouts. g, SEM image of single-crystal MoS_2 multiple FETs. h,i, Relative mobility deviation (h) and relative standard deviation (i) of single FET arrays and multiple FETs.

(p. 32) Extended Data Fig. 10 | Alignment of 2D TMD lateral heterostructure for self-aligned ALD contact FET. a-d, Schematic (a) and SEM images (b-d) of aligned 2D MoS_2-$MoSe_2$ lateral heterostructure by EF-SCN method. e, Schematic of self-aligned ALD contact FET with Ru AS-ALD on 2D MoS_2-$MoSe_2$ lateral heterostructure.

ii) We have revised **manuscript (p. 7)** to explain that EF-SCN can be extended to low-temperature growth by engineering the etching-flux barrier materials with lower crystallization temperatures.

(p. 7) "As shown in the SEM images in Extended Data Fig. 5b, the use of a ZrO_2 etching-flux barrier results in effective single-centered nucleation, rather than edge nucleation, even at a low MoS_2 MOCVD temperature of 500 °C. These results suggest that selecting barrier materials with lower crystallization temperatures can enable EF-SCN at further reduced growth temperatures. ~"

iii) We have improved **manuscript (p. 10-11)** to demonstrate superior electrical performance of single-crystal single FET arrays and low-variation single-crystal multiple FETs.

(p. 10-11) "Importantly, a high field-effect mobility of 117 $cm^2V^{-1}s^{-1}$ was achieved, significantly surpassing previously reported values for selectively grown MoS_2 FETs, indicating the excellent electrical properties of single-crystal MoS_2 by EF-SCN[8,9]. In Fig. 4d, the mobility distribution across a total of 16 single FET arrays reveals an average mobility of 74 $cm^2V^{-1}s^{-1}$ with a RSD of 21.8%, demonstrating a performance variation comparable to that of previous 2D TMD single-crystal FETs. ~", "In the case of single-crystal single FETs, the RSD values for each set were 15.7%, 14.8%, 14.1%, and 23.8% (21.8% overall), whereas the single-crystal multiple FETs exhibited a significantly lower RSD of 7.8%. This corresponds to a 65% reduction in mobility variation compared to the single FET arrays, indicating that fabricating multiple channels within a single-crystal is more advantageous in terms of variation than defining a single channel in one single-crystal. ~"

iv) We have reworked **manuscript (p. 11-12)** to illustrate the self-aligned ALD contact FETs enabled by spatially aligned 2D MoS_2-$MoSe_2$ lateral heterostructures.

(p. 11-12) "Fig. 4e-f present a schematic and SEM images illustrating the fabrication of self-aligned ALD contact FETs. When Ru ALD is conducted on the 2D MoS_2-$MoSe_2$ lateral heterostructures with precisely controlled position and orientation via the EF-SCN approach, selective deposition occurs on the $MoSe_2$ region due to the local strain distribution (Fig. 4e, g). As shown in Fig. 4f, a well-aligned 2D/3D hybrid structure is achieved at the array level, and AFM measurements confirm the formation of 15 nm-thick Ru film."

(2) The proposed growth strategy exhibits extremely low nucleation density (~one nucleus per tens of square micrometers) and poses a risk of nucleation failure, which may be harmful than grain boundaries during large-area integration. What's more, slight variations in oxygen flow dynamics across a wafer could lead to over-etching or under-etching. Thus, what is the specific relationship between the growth outcomes and oxygen partial pressure? Furthermore, what is the wafer-scale uniformity of this method?

[Author reply]

This comment raises important questions regarding the scalability and practical applicability of the EF-SCN approach. To address this comment, it is essential to achieve integration capability.

(i) Effective single-crystal growth in high-density patterns

From the perspective of device integration, it is critical to evaluate whether the density of a single-crystal can be increased in the EF-SCN system. To this end, we analyzed the growth behavior as a function of pattern density. In the EF-SCN method, nucleation is initiated at the center of each pattern, followed by lateral growth that fills the growth region with a single-crystal channel (Fig. R15a). Fig. R15b shows SEM images of EF-SCN for three different pattern densities (0.009/μm^2, 0.025/μm^2, and 0.068/μm^2) with a growth region size of 3 μm. The corresponding DES flow rates were 3, 3.5, and 4 sccm, respectively. Notably, even at the highest pattern density (Fig. R15b(iii)), complete single-crystal growth was achieved at all sites.

As shown in Fig. R15c, nucleation failure can occur under low nucleation density conditions. This issue is not unique to EF-SCN, but is also observed in conventional selective growth methods. A straightforward solution is to increase the nucleation density through appropriate growth conditions (Fig. R15d). As demonstrated in Fig. R15b(iii), sufficiently high DES flow enables high single-crystal density and filling yield comparable to those achieved in the confined growth method.

It should be noted that excessive DES flow may induce multiple nucleation events even within a 3 μm growth region, indicating an inherent trade-off. However, as shown in Fig. R16, valid single-crystal growth is maintained within a DES flow window of 3-7 sccm for 3 μm patterns. Therefore, further optimization within this process window is expected to enable even higher single-crystal growth density.

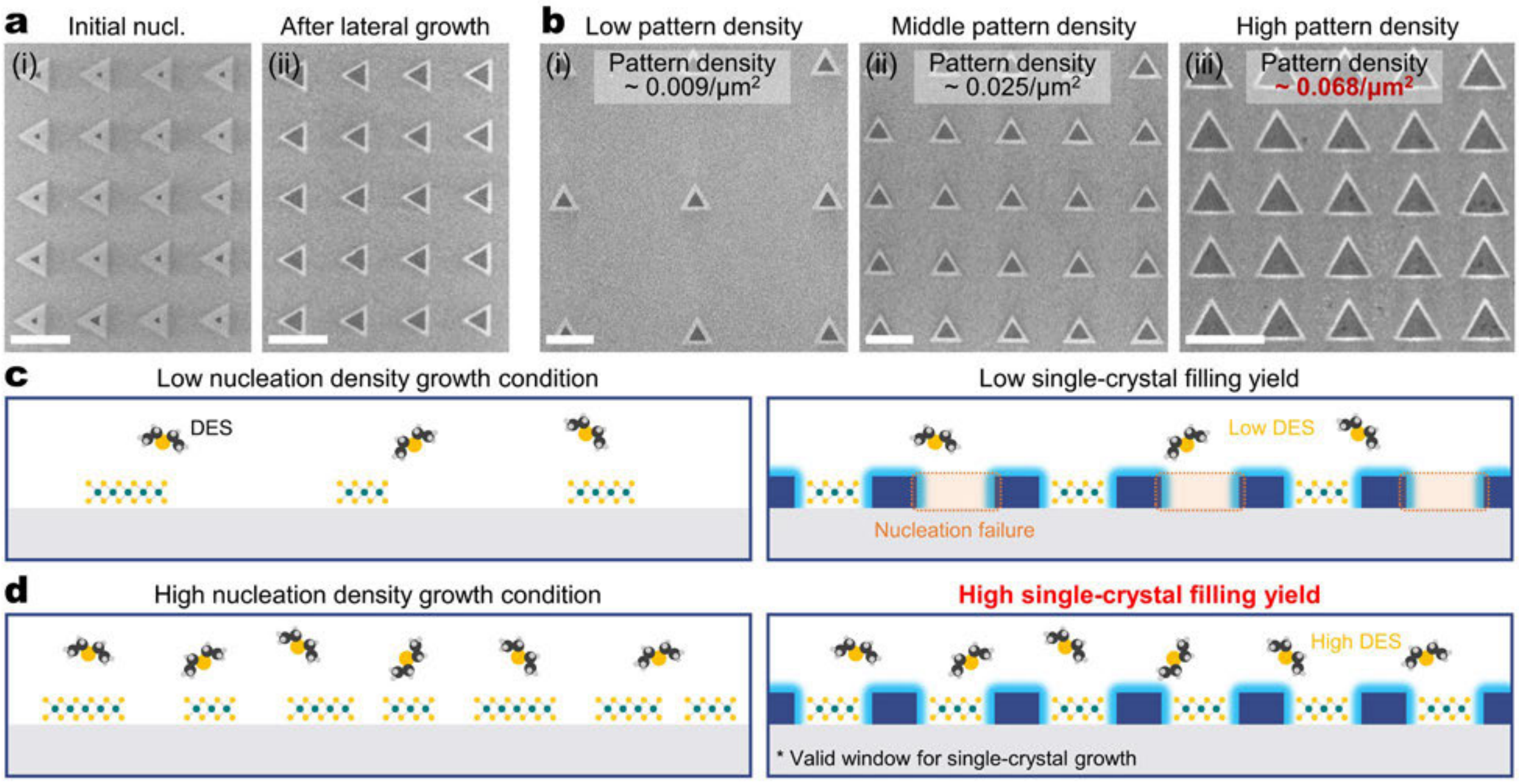

Fig. R15 | High-density single-crystal growth by EF-SCN. a, Initial single-centered nucleation and lateral growth behavior with high-density pattern. **b**, MoS_2 EF-SCN behaviors in (i) low, (ii) middle, and (iii) high pattern density substrates. Scale bars, 5 μm. **c**,**d**, Schematic of MoS_2 single-crystal filling yield in EF-SCN system with low nucleation density condition (**c**) and high nucleation density condition (**d**).

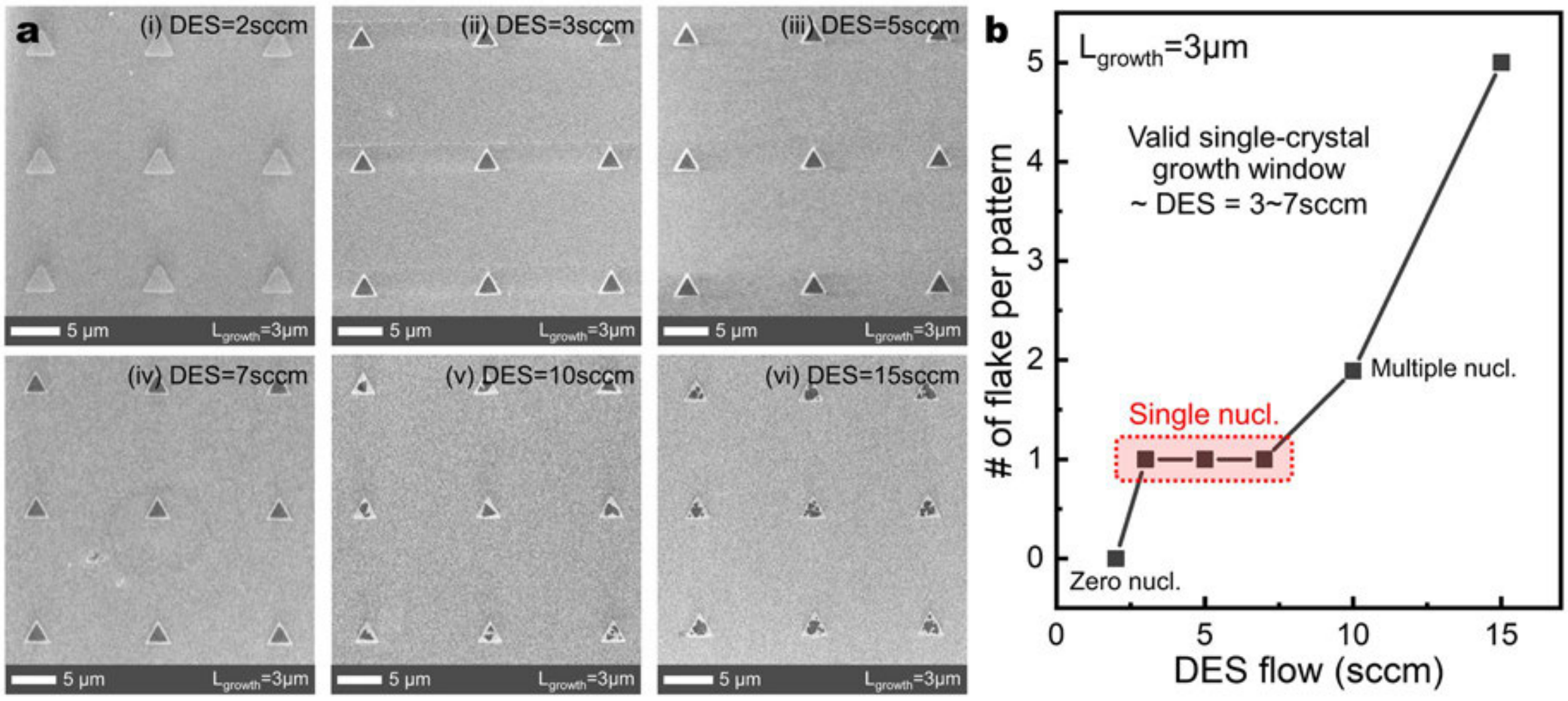

Fig. R16 | Valid process window for single-crystal MoS_2 growth. a, SEM images showing MoS_2 EF-SCN behavior with DES flow (2-15 sccm). **b**, Plot of the number of MoS_2 flakes per pattern as a function of DES flow.

(ii) Large-scale uniformity of EF-SCN

To evaluate the scalability of EF-SCN across large areas, we fabricated 25 patterns at 16 regions on a 2 cm miscut sapphire substrate, resulting in a total of 400 sites. The resulting MoS_2 growth behavior on the substrate is shown in Fig. R17a-c. SEM analysis (Fig. R17b) shows that single-centered nucleation is observed at 397 out of 400 sites. This corresponds to a single-crystal yield of 99.3%, which demonstrates the strong potential of EF-SCN for large-scale applications (Fig. R17c).

Fig. R17d presents OM and SEM images showing EF-SCN growth behavior over a large area exceeding 400 μm on an amorphous SiO_2 substrate using an HfO_2 barrier. Even on an amorphous substrate, uniform single-crystal growth was achieved across more than 2500 sites. The enlarged SEM images of 9 randomly selected sites confirm the formation of a monolayer MoS_2 film with bilayer MoS_2 flakes. Since the substrate is amorphous SiO_2, the crystallographic orientation cannot be controlled, resulting in different orientations of the bilayer flakes between the 9 sites. However, within each site, the bilayer flakes exhibit consistent orientation, indicating that the underlying monolayer MoS_2 film is a single-crystal.

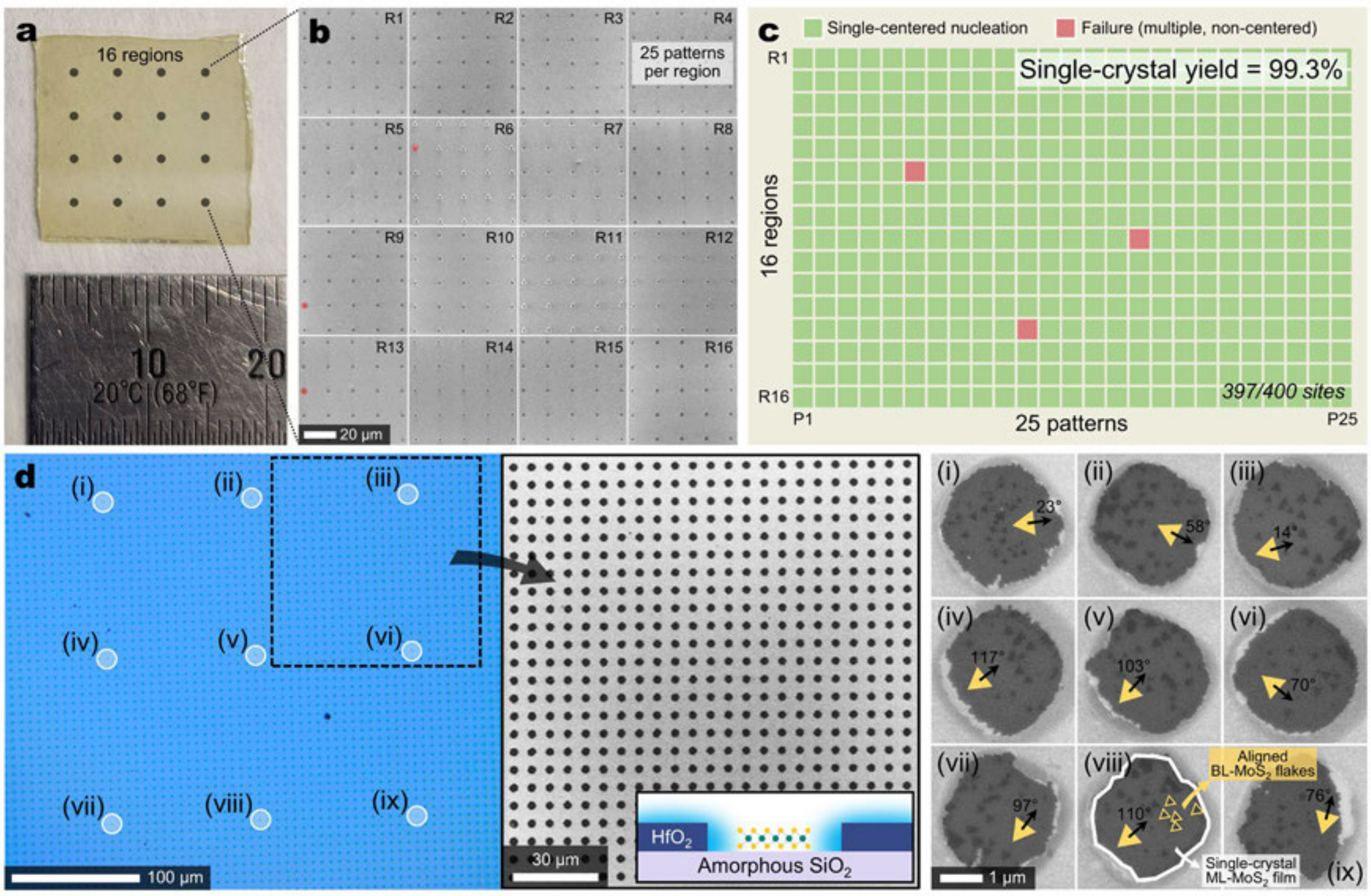

Fig. R17 | Large-scale single-crystal growth of EF-SCN. a-c, OM image (**a**), SEM images (**b**), and single-crystal mapping image (**c**) of large-scale MoS_2 EF-SCN consisting of 16 regions, each containing 25 patterns (a total of 400 sites). **d**, OM image and SEM images showing the uniformity of single-crystal MoS_2 EF-SCN growth on an amorphous SiO_2 substrate.

(iii) Unintentional etching effect by oxygen flux from barrier

The effect of oxygen released from the etching-flux barrier on the TMD channel and the substrate are discussed in detail in the response to 3rd comment.

[Action in Manuscript]

i) We have revised **Fig. 2,3 (p. 20,21)** and added **Extended Data Fig. 5 (p. 27)** and **Supplementary Fig. 7,9 (SI p. 8,10)** to demonstrate the process compatibility of EF-SCN, including high-density single-crystal growth, large-scale uniformity, and valid single-crystal growth window.

(p. 20) Fig. 2 | Etching-flux barrier mechanism and process compatibility of EF-SCN system. d, OM image and SEM images showing single-crystal MoS_2 growth by EF-SCN on amorphous SiO_2 substrate. e-g, OM image (e), SEM images (f), and single-crystal mapping (g) of large-scale MoS_2 EF-SCN consisting of 16 regions, each containing 25 patterns (a total of 400 sites).

(p. 21) Fig. 3 | Nucleation design rule of 2D MoS_2 EF-SCN. e, The average number of MoS_2 flakes per pattern as function of DES flow at L_{growth}=3 µm.

(p. 27) Extended Data Fig. 5 | Low-temperature EF-SCN and high-density single-crystal growth. c, SEM images of MoS_2 EF-SCN behaviors in (i) low, (ii) middle, and (iii) high pattern density substrates. Scale bars, 5 µm.

(SI p. 8) Supplementary Fig. 7 | 2D MoS_2 EF-SCN on amorphous substrate. a, SEM images of 2D MoS_2 EF-SCN on amorphous SiO_2 substrate, corresponding to the enlarged views of Fig. 2d (i-ix). Small bilayer MoS_2 flakes (dark contrast) are observed on the monolayer MoS_2 surface, exhibiting a consistent crystallographic orientation within each pattern site.

(SI p. 10) Supplementary Fig. 9 | Valid DES flow window for single-crystal MoS_2 growth. a, SEM images showing MoS_2 EF-SCN behavior with DES flow (2-15 sccm).

ii) We have updated **manuscript (p. 7)** to describe the scalability of EF-SCN in high-density patterns.

(p. 7) "As shown in Extended Data Fig. 5c, effective single-crystal growth is achieved across a wide range of pattern densities (0.009–0.068 patterns/µm^2). Notably, single-crystal growth is maintained even at pattern densities more than seven times higher than that in Extended Data Fig. 5c(i)."

iii) We have revised **manuscript (p. 8)** to demonstrate large-scale uniformity of single-crystal growth by EF-SCN.

(p. 8) "Importantly, Fig. 2e-g demonstrate the single-crystal yield and uniformity at a large-scale. A total of 400 sites were fabricated across 16 regions on a 2 cm substrate (25 patterns per region), achieving a single-crystal yield exceeding 99%. These results indicate that EF-SCN provides strong compatibility with semiconductor processing and scalability toward integration."

iv) We have improved **manuscript (p. 9)** to define the DES flow window for valid single-crystal MoS_2 growth.

(p. 9) "Fig. 3e shows the nucleation behavior as a function of DES flow (2-15 sccm) for L_{growth} = 3 µm. As observed in the SEM images in Supplementary Fig. 9, zero nucleation occurs at 2 sccm, while multiple nucleation is observed at 10-15 sccm. A DES flow range of 3-7 sccm is identified as an effective window for single-crystal MoS_2 growth."

(3) Continuous oxygen flux would induce unintentional etching, how does this affect the intrinsic material quality?

(5) Does the high temperature oxygen anneal degrade the surface roughness or interface trap density of the underlying dielectric? A roughened interface due to etching would render this method unsuitable for high-performance logic. Please provide more discussion.

(8) Can this oxygen-mediated strategy support vertical heterostructure growth without destroying the underlying TMD layer?

[Author reply]

Unlike other 2D TMD selective growth methods, EF-SCN uses an oxygen etching-flux released from the barrier; therefore, it is necessary to examine how this oxygen affects the single-crystal quality and subsequent growth behavior.

(i) Finite oxygen release in the etching-flux barrier

The HfO_2 etching-flux barrier employed in this study is expected to release oxygen during its crystallization process, which is thermally activated under MoS_2 growth conditions. However, as crystallization progresses, the oxygen reservoir becomes gradually depleted, implying that the total oxygen flux is intrinsically finite. As evidenced by the XPS data in Main Fig. 2c, the oxygen content in HfO_2 decreases rapidly within the first 10 minutes at 750 °C and subsequently reaches a saturated state. This observation indicates that the oxygen flux is most pronounced during the initial centered nucleation stage and gradually diminishes thereafter. Consequently, the resulting single-crystal film is not expected to experience unintentional and excessive etching effects.

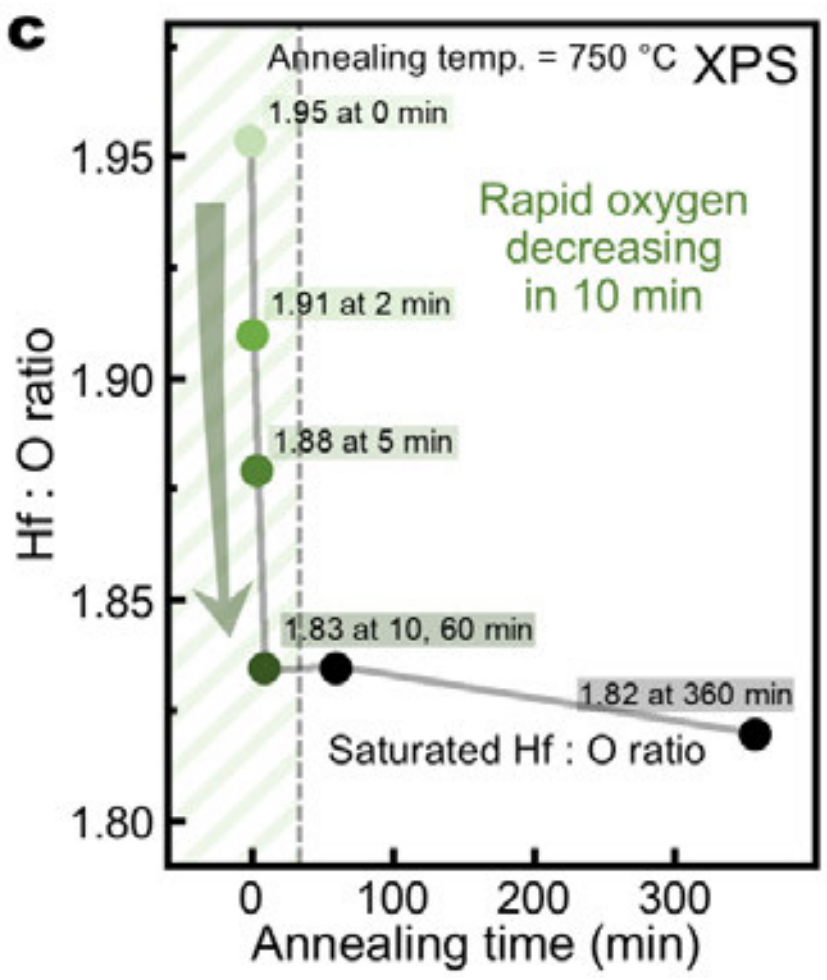

Main Fig. 2c

Fig. R18a provides experimental evidence for the time-dependent attenuation of the etching effect from the etching-flux barrier. As observed in the SEM images, once a single-crystal monolayer MoS_2 film has fully formed, subsequent bilayer formations no longer occur at the central region but instead emerge at random locations on the monolayer surface. This clearly demonstrates that the oxygen flux released from HfO_2 becomes insufficient to enforce spatial selectivity of nucleation over time.

Fig. R18b presents HAADF-STEM images of EF-SCN single-crystal MoS_2 transferred onto a TEM grid. Atomic-scale analysis reveals the presence of sulfur vacancy sites, manifested as reduced contrast at chalcogen positions. Importantly, the observed defect density is comparable to that reported for conventional CVD-grown MoS_2, suggesting that oxygen from the etching-flux barrier does not significantly degrade the MoS_2 film quality[16,39,40]. Moreover, severe oxygen exposure could induce molybdenum vacancy sites; however, no molybdenum vacancies are observed in the HAADF-STEM analysis, indicating that the crystalline quality remains comparable to that of standard CVD-grown MoS_2. A more detailed discussion of single-crystal quality is provided in the response to the 4th comment.

(ii) Potential impact of oxygen on the underlying dielectric film

The amount of oxygen released from the etching-flux barrier in the EF-SCN system is expected to be extremely low compared to other studies in which oxygen is introduced into the gas phase for 2D TMD growth control. In fact, MoS_2 MOCVD research using oxygen gas reported that even an oxygen fraction of 0.04% can completely suppress MoS_2 growth[19]. In contrast, under the EF-SCN system, nucleation occurs at the center of the growth region, implying that the effective oxygen partial pressure is substantially lower than that reported in such studies. This suggests that the oxygen level is sufficiently low to avoid detrimental effects on the channel region.

The impact of this trace oxygen flux on the underlying dielectric film is expected to be minimal. In particular, for crystalline substrates such as sapphire, it is unlikely that low oxygen exposure would induce measurable surface degradation, such as increased roughness. This is further supported by the AFM data in Extended Data Fig. 1b, where no spatial variation in surface height is observed within the growth region.

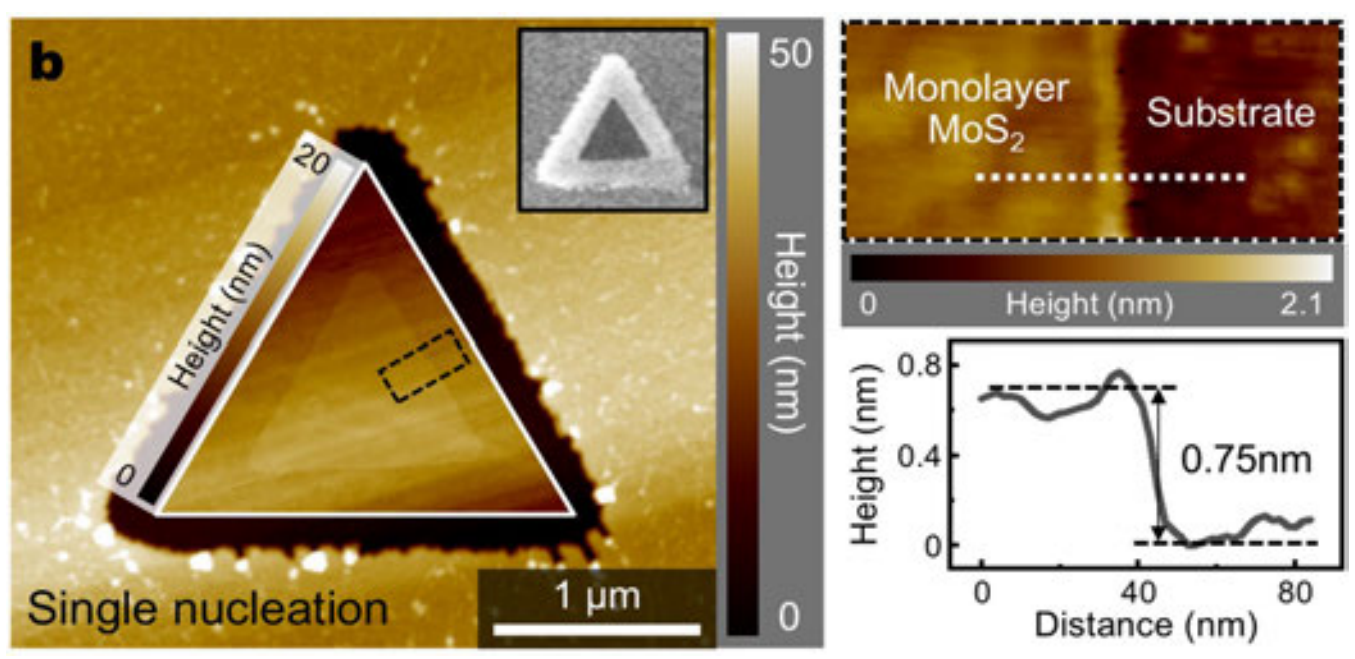

Extended Data Fig. 1b

Nevertheless, as the reviewer correctly noted, when the underlying dielectric is intended for device operation, even subtle modifications must be carefully examined. To address this point, we employed amorphous SiO_x, which is more susceptible to oxygen-induced changes than crystalline sapphire. As shown in Fig. R18c, MoS_2 was grown on amorphous SiO_x substrates using both oxygen-free MOCVD and EF-SCN with a ZrO_2 barrier. After growth, the

MoS_2 films were removed via a transfer process, and XPS measurements were performed on the exposed SiO_x surface. For the MOCVD sample without the etching-flux barrier, we measured the SiO_x surface where MoS_2 had previously been present. In the case of the EF-SCN sample with the barrier, measurements were performed at a location 10 µm away from the barrier edge.

As shown in the Si 2p XPS spectra in Fig. R18c, binding energy peaks are observed at 103.5eV without the barrier and at 103.6eV with the barrier. In addition, the O 1s XPS spectra exhibits an identical peak position at 532.65eV in both cases. These results indicate that the oxygen released from the etching-flux barrier does not have a detrimental effect on the underlying SiO_x surface. Furthermore, XPS analysis of the SiO_x film reveals atomic ratios of $SiO_{1.506}$ without the barrier and $SiO_{1.502}$ with the barrier, confirming that the films are essentially identical.

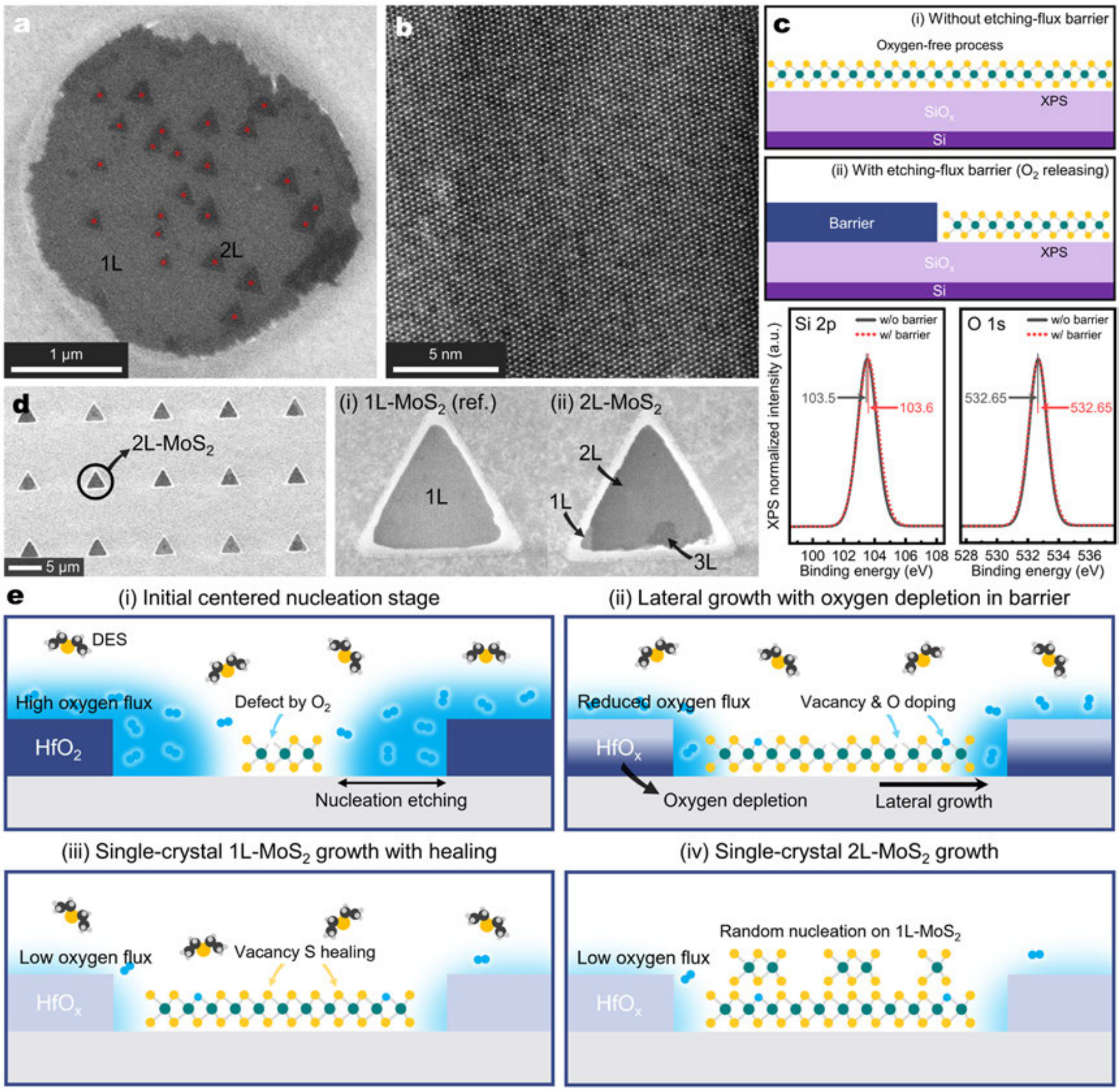

Fig. R18 | Impact of oxygen released from etching-flux barrier on MoS_2 growth and adjacent regions. a, SEM image showing nucleation of 2L-MoS_2 flakes at random positions on 1L-MoS_2 basal plane. **b**, Representative HAADF-STEM image of MoS_2 by EF-SCN. **c**, Schematic and XPS analysis comparing the quality of underlying dielectric layer (amorphous SiO_x) between conventional MoS_2 MOCVD without barrier and EF-SCN with barrier. **d**, SEM images of 2L-MoS_2 growth by EF-SCN. **e**, Process schematic depicting centered nucleation, oxygen depletion in barrier, defect formation/healing, and vertical growth.

(iii) 2D vertical growth potential in EF-SCN

As shown in Fig. R18a and R18b, for monolayer single-crystal growth, the effect of oxygen release diminishes over time; therefore, degradation of the single-crystal quality is not expected. Instead of demonstrating vertical heterostructure growth using the EF-SCN approach, we simply examined the growth behavior of bilayer MoS_2 to evaluate the feasibility of 2D vertical growth. Fig. R18d presents SEM images of bilayer MoS_2 growth via the EF-SCN method. Based on SEM contrast, both fully bilayer MoS_2 and partially bilayer MoS_2 regions are observed. The enlarged SEM images further suggest that 2D vertical growth is achievable within the EF-SCN system. To effectively realized 2D vertical heterostructures, precise control of both the lateral growth rate and the duration of oxygen release from the HfO_2 barrier will be required.

(iv) Time-dependent 2D TMD growth scenario in EF-SCN system

Fig. R18e presents a schematic that more clearly illustrates the growth behavior of EF-SCN. At the initial stage of MoS_2 growth, a high oxygen flux released from HfO_2 induces a strong nucleation-etching effect, driving selective nucleation at the center — the point farthest from the barrier (Fig. R18e(i)).

As growth proceeds, the MoS_2 nucleated at the center fills the growth region through lateral growth, while oxygen depletion begins in the HfO_2 barrier (Fig. R18e(ii)). As a result, the oxygen flux gradually decreases, weakening its nucleation-suppression capability. As noted by the reviewers, during this stage, oxygen may induce processes such as sulfur vacancy formation or oxygen doping, which could influence the film quality.

With further time, only a trace amount of oxygen is released from HfO_x. At this stage, the oxygen flux remains sufficient to suppress MoS_2 nucleation on the barrier but is no longer strong enough to control nucleation positions within the growth region (Fig. R18e(iii)). In addition, a continuous supply of MOCVD precursors facilitates partial healing of defect sites.

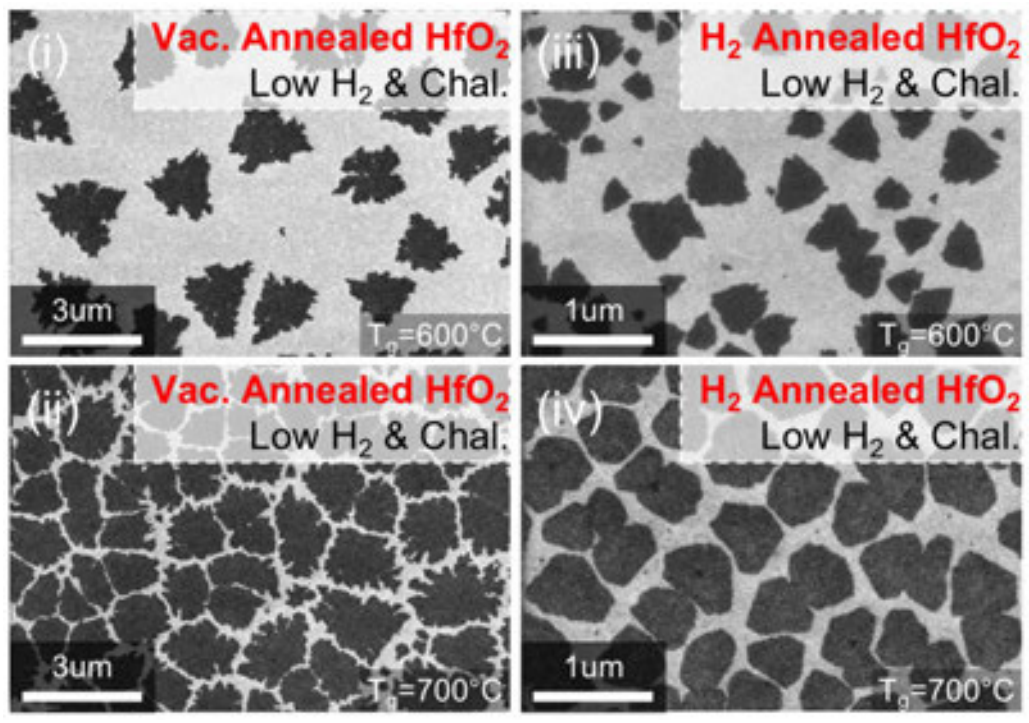

Fig. R6c

At the final stage, as the oxygen flux from HfO_x becomes insufficient to maintain spatial control, bilayer MoS_2 flakes nucleate at random locations on the monolayer MoS_2 surface (Fig. R18e(iv)). Ultimately, after a sufficiently long duration, HfO_x may completely lose its ability to release oxygen, which could lead to undesired growth on the barrier. However, as shown in Fig. R6c, MoS_2 growth on HfO_x requires extreme conditions such as thermal annealing at 1000 °C for 1 hour. Therefore, considering the typical EF-SCN growth duration and conditions, the selectivity window of the HfO_2 barrier is expected to be sufficiently wide.

[Action in Manuscript]

i) We have added **Extended Data Fig. 2 (p. 24)** and **Supplementary Fig. 6,7,8 (SI p. 7,8,9)** to verify the oxygen-flux behavior and its impact on the channel quality and underlying substrate.

(p. 24) Extended Data Fig. 2 | TEM and atomic resolution AFM measurements of single-crystal MoS_2 flake by EF-SCN. a, TEM image of single-crystal MoS_2 flake fully suspended over a TEM grid hole. b, Diffraction pattern image obtained from the single-crystal in a. c, HAADF-STEM images acquired at S1, S2, and S3 positions marked in a, showing consistent atomic lattice orientation across the flake.

(SI p. 7) Supplementary Fig. 6 | Oxygen flux and nucleation rate in EF-SCN system. e, Process schematic depicting centered nucleation, oxygen depletion in barrier, defect formation/healing, and vertical growth.

(SI p. 8) Supplementary Fig. 7 | 2D MoS_2 EF-SCN on amorphous substrate. a, SEM images of 2D MoS_2 EF-SCN on amorphous SiO_2 substrate, corresponding to the enlarged views of Fig. 2d (i-ix). Small bilayer MoS_2 flakes (dark contrast) are observed on the monolayer MoS_2 surface, exhibiting a consistent crystallographic orientation within each pattern site.

(SI p. 9) Supplementary Fig. 8 | Impact of oxygen released from an etching-flux barrier on underlying substrate. a,b, Schematic (a) and XPS analysis (b) comparing the quality of underlying dielectric layer (amorphous SiO_x) between conventional MoS_2 MOCVD without barrier and EF-SCN with barrier.

ii) We have added **Supplementary Note 3 (SI p. 26-27)** to discuss oxygen depletion from the etching-flux barrier and evaluate its influence on the MoS_2 quality and the underlying dielectric layer.

(SI p. 26-27) "As observed in the SEM images, once a single-crystal monolayer MoS_2 film has fully formed, subsequent bilayer nucleation no longer occurs at the central region but instead emerges at random locations on the monolayer surface. This clearly demonstrates that the oxygen flux released from HfO_2 becomes insufficient to enforce spatial selectivity of nucleation over time. ~", "Atomic-scale analysis reveals the presence of sulfur vacancy sites, manifested as reduced contrast at chalcogen positions. Importantly, the observed defect density is comparable to that reported for conventional CVD-grown MoS_2, suggesting that oxygen from the etching-flux barrier does not significantly degrade the MoS_2 film quality[28-30]. ~", "As shown in the Si 2p XPS spectra in Supplementary Fig. 8b, binding energy peaks are observed at 103.5eV without the barrier and at 103.6eV with the barrier. In addition, the O 1s XPS spectra exhibit an identical peak position at 532.65eV in both cases. These results indicate that the oxygen released from the etching-flux barrier does not have a detrimental effect on the underlying SiO_x surface. ~"

(4) On a flat substrate, the single-crystallinity of TMDs could be easily confirmed by the triangular or hexagonal geometries due to the kinetic Wulff criterion (ACS Nano 2016, 10, 4330). However, in this space-confined strategy, the final triangular morphology of the TMD domains could be determined by anisotropic etching. Therefore, the authors should provide more effective characterizations for the single-crystallinity of the as-grown arrays, like TEM?

[Author reply]

This comment raises an important question regarding whether the flakes formed via EF-SCN are indeed single-crystal. Therefore, we regarded this comment as a critical task in the revision process and performed rigorous characterizations. Since previous grain boundary observation by AFM is insufficient to verify single-crystallinity, we additionally conducted a comprehensive analysis using TEM, atomic-resolution AFM, second-harmonic generation (SHG), faceting of flake edges, and bilayer flake orientation alignment.

(i) TEM

Fig. R19 presents TEM analysis results of a single-crystal MoS_2 flake grown by EF-SCN. After selective growth of MoS_2, a PMMA layer was coated, and the flakes were transferred onto a quantifoil TEM grid via a wet-transfer process, followed by PMMA removal. The optical microscopy (OM) image in Fig. R19a shows MoS_2 flakes transferred onto the TEM grid; except for one flake, the others were folded or torn. Fig. R19b shows a low-magnification TEM image of a flake successfully transferred across a hole, and Fig. R19c presents the corresponding diffraction pattern. In the case of polycrystalline MoS_2 films containing grain boundaries, additional spots or ring patterns are typically observed. However, Fig. R19c exhibits only a hexagonal spot pattern, strongly indicating that the flake is a single-crystal MoS_2.

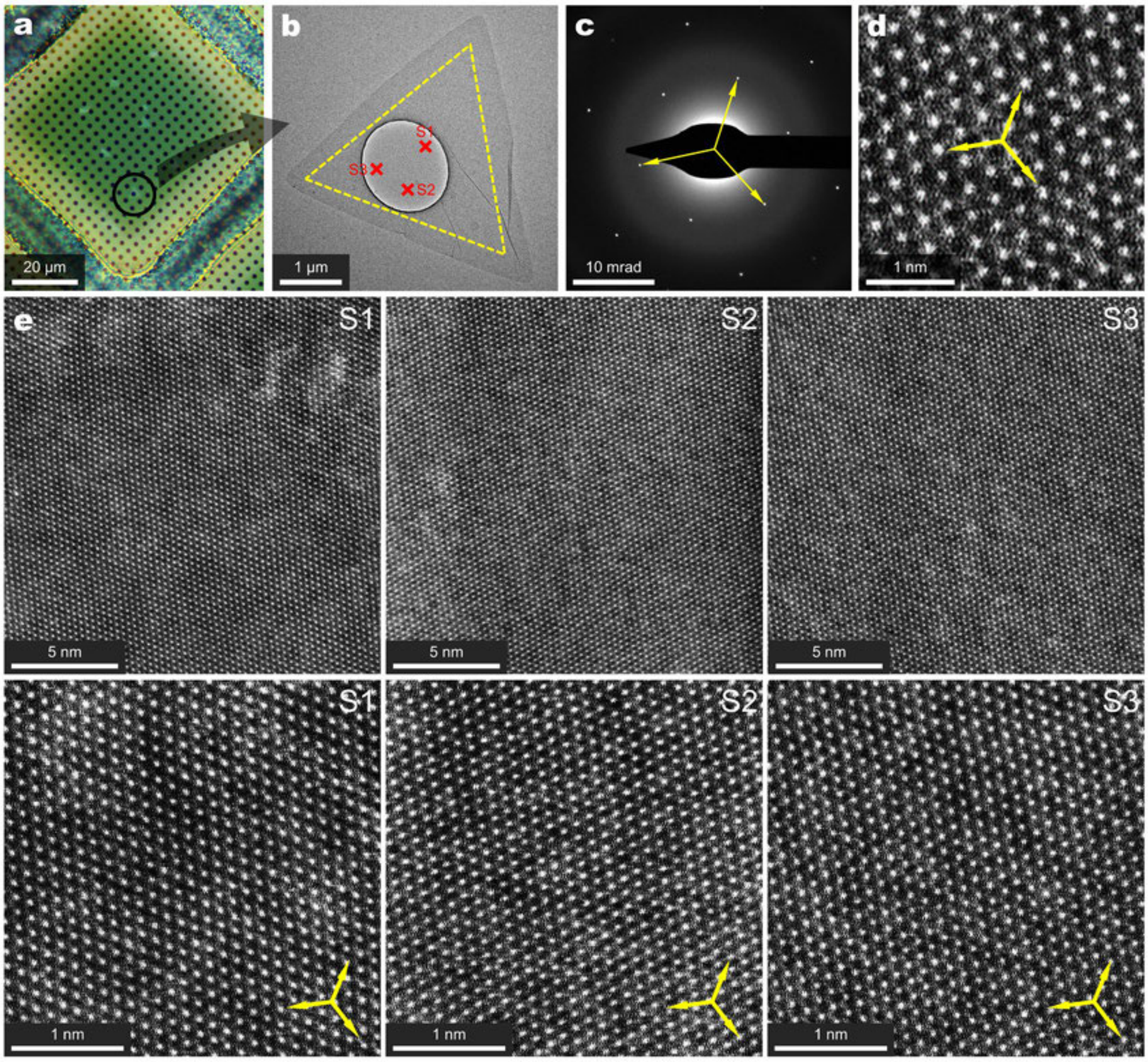

Fig. R19 | Structural TEM characterization of single-crystal MoS_2 flake by EF-SCN. a, OM image of EF-SCN single-crystal MoS_2 flakes transferred onto a quantifoil TEM grid. **b**, TEM image of single-crystal MoS_2 flake fully suspended over a hole. **c**,**d**, Diffraction pattern (**c**) and corresponding HAADF-STEM image (**d**) obtained from the single-crystal in **b**. **e**, HAADF-STEM images acquired at the S1, S2, and S3 positions marked in **b**, showing consistent atomic lattice orientation across the flake.

Fig. R19d shows an HAADF-STEM image corresponding to Fig. R19b, where the hexagonal lattice structure of monolayer 2H-phase MoS_2 is clearly observed, with its orientation consistent with the diffraction pattern in Fig. R19c. Fig. R19e presents HAADF-STEM images acquired at three positions (S1, S2, and S3) within the flake, confirming that the atomic lattice orientation is uniform across all regions, which directly supports the single-crystal nature of the flake. Furthermore, comparable surface quality is observed at the flake center (S1), edge (S2), and corner (S3), indicating that spatial variations in flake quality due to oxygen from the etching-flux barrier can be ruled out.

Based on these TEM analyses, it can be conclusively determined that the MoS_2 flake grown by the EF-SCN method is single-crystalline. In addition, the triangular morphology is not a result of anisotropic etching but rather originates from epitaxial growth aligned with the barrier pattern orientation, confirming the formation of well-defined triangular single-crystal MoS_2 flakes.

(ii) Atomic resolution AFM

Fig. R20 presents atomic resolution AFM analysis of a MoS_2 flake grown by EF-SCN. Unlike TEM measurements, which require transfer onto a grid, AFM measurements were performed directly after growth without additional processing. Fig. R20a shows an SEM image of single-crystal MoS_2 flakes grown within a 3 μm pattern, and Fig. R20b provides a low-magnification AFM image of the black dotted region indicated in Fig. R20a. This image clearly delineates the HfO_2 barrier and the MoS_2 region formed at the center of the growth area.

Fig. R20c shows a representative atomic resolution AFM image acquired from the MoS_2 region in Fig. R20b. A well-defined periodic hexagonal lattice, characteristic of MoS_2, is clearly observed across the entire scanned area. Considering previously reported atomic resolution AFM and conductive AFM results for MoS_2, the uniform contrast and continuous lattice observed in Fig. R20c indicate high crystallinity and a low defect density[41].

Fig. R20d presents atomic resolution AFM images obtained from three sites within Fig. R20b. The insets show the corresponding FFT patterns, where clear hexagonal symmetry consistent with MoS_2 is observed. The angles extracted from the FFT patterns at the three locations fall within a range of 22-24°, which is within the typical error margin arising from tip drift during AFM measurements. Therefore, these results provide strong evidence that the flake in Fig. R20b is single-crystal across its entire area.

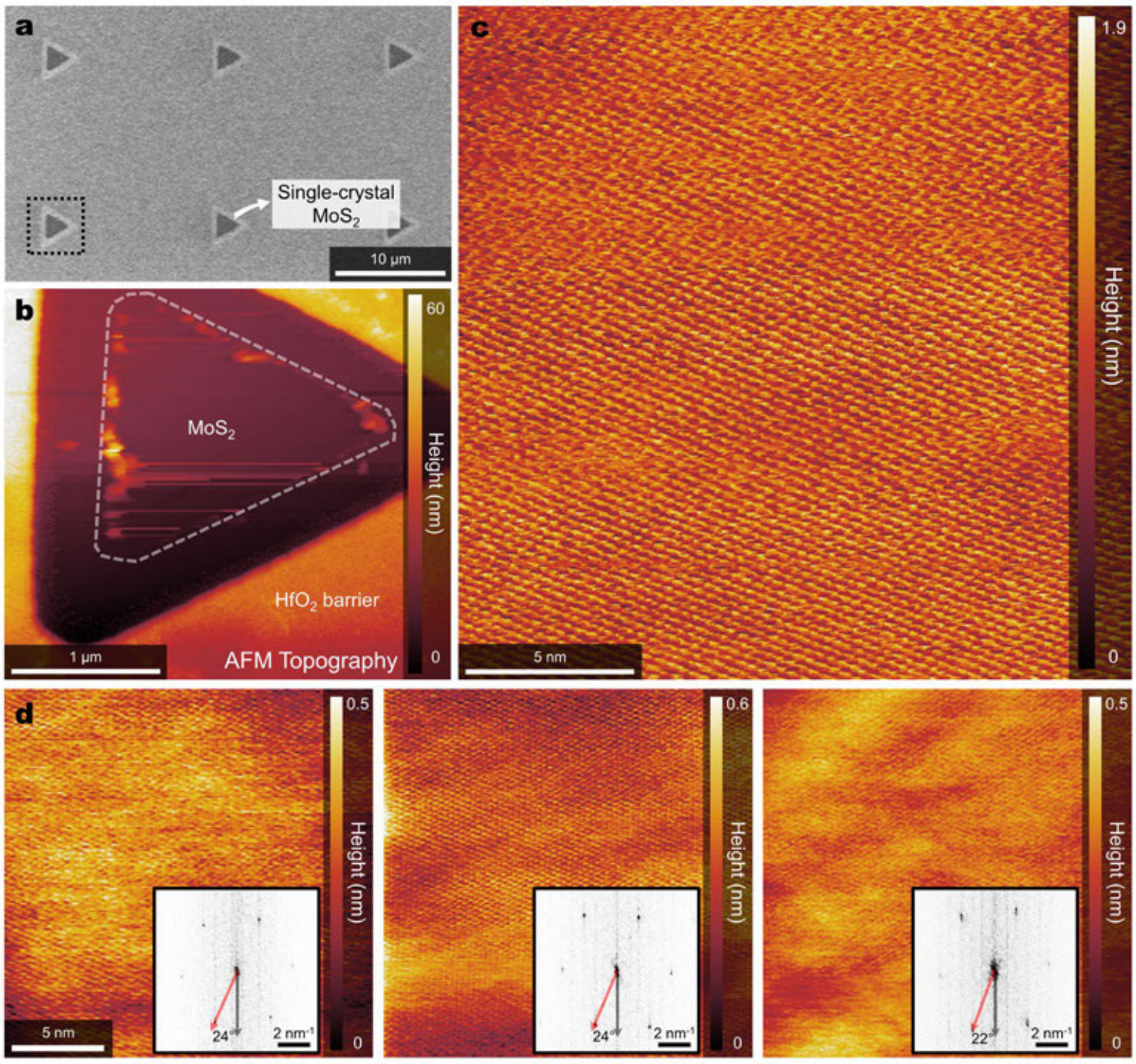

Fig. R20 | Structural AFM characterization of single-crystal MoS_2 flake by EF-SCN. a,**b**, SEM (**a**) and AFM (**b**) images of EF-SCN single-crystal MoS_2 flake. **c**,**d**, Atomic scale high resolution AFM images of single-crystal MoS_2 flake in **b** with FFT image.

(iii) SHG

Fig. R21 presents a rigorous analysis of MoS_2 flakes grown by the EF-SCN method using second-harmonic generation (SHG). Under a low DES growth condition (3 sccm) that promotes single nucleation, the SEM image (Fig. R21a) clearly shows the formation of a single-crystal MoS_2 flake within each pattern. The corresponding SHG mapping image (Fig. R21b) exhibits uniform SHG intensity across the entire flake area, indicating the absence of grain boundaries and thus confirming its single-crystal nature. Furthermore, as shown in Fig. R21c, angle-resolved SHG measurements reveal a well-defined six-petal pattern with clear six-fold symmetry, providing critical evidence that the MoS_2 flakes in Fig. R21a possess single-crystal SHG characteristics.

In contrast, under a high DES growth condition (15 sccm) that induces multiple nucleation, SEM images (Fig. R21d) show multiple MoS_2 flakes within each pattern. The corresponding SHG mapping image (Fig. R21e) displays regions of significantly reduced SHG intensity, indicative of grain boundaries and thus confirming the polycrystalline nature of the MoS_2. In addition, the angle-resolved SHG measurement in Fig. R21f shows an isotropic SHG intensity distribution, lacking the distinct six-petal pattern. This behavior can be attributed to the averaging of SHG signals from multiple domains with different orientations, leading to the loss of directional anisotropy. These results clearly demonstrate that the growth region in Fig. R21d consists of polycrystalline MoS_2.

Furthermore, angle-resolved SHG measurements were performed at the array level, as shown in Fig. R21g and R21h. All six randomly measured flakes exhibit clear six-petal SHG patterns, confirming their single-crystal nature. The SHG patterns of these flakes show similar crystallographic orientations within a range of 57-63°. The slight variation in orientation is attributed to the relatively low epitaxial growth temperature of 750 °C, which may limit the degree of alignment compared to higher temperature epitaxial growth. Nevertheless, the SHG analysis confirms that MoS_2 flakes grown via EF-SCN are unequivocally single-crystalline and exhibit consistent crystallographic orientation.

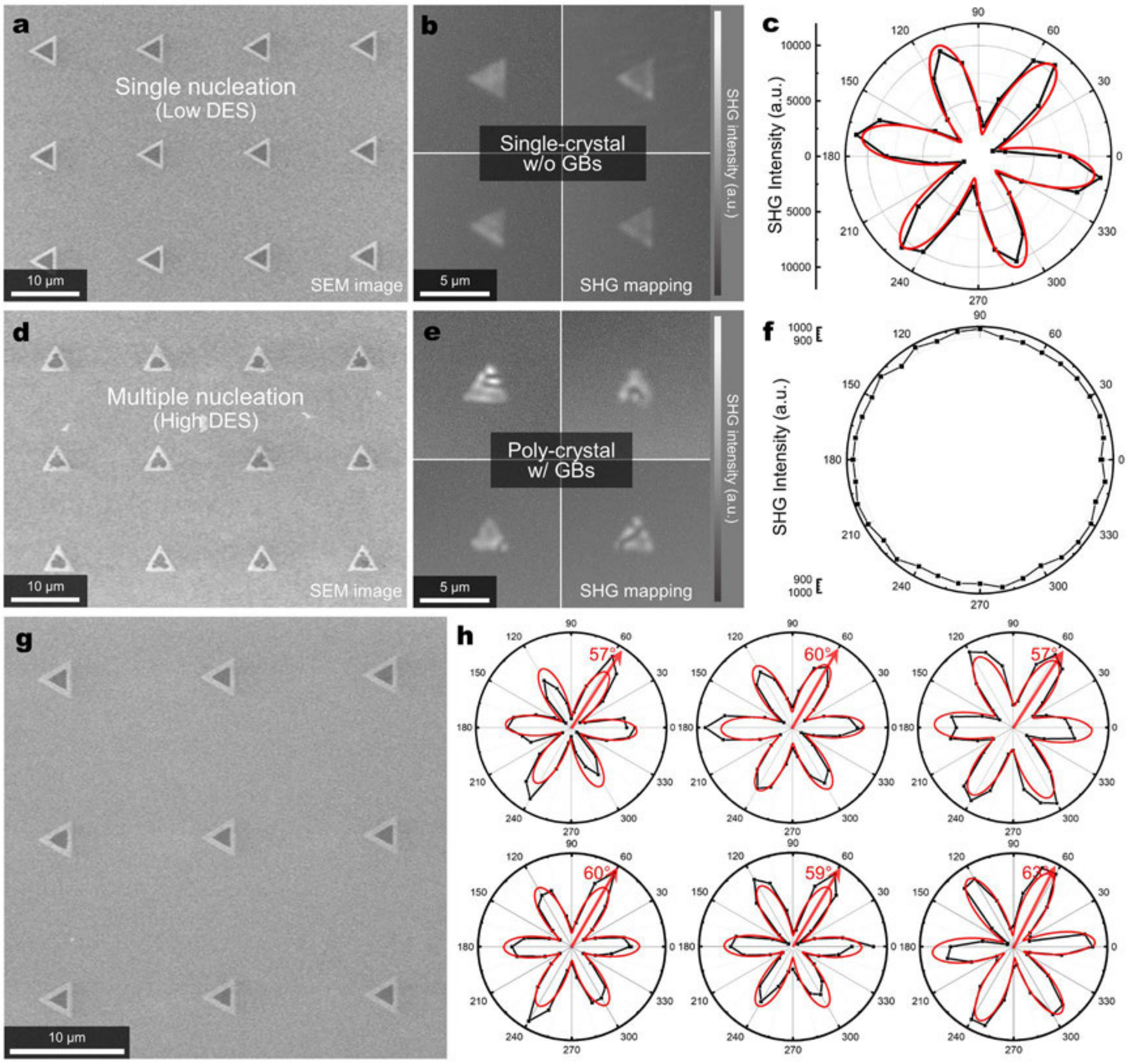

Fig. R21 | SHG mapping and angle-resolved SHG patterns of single-crystal MoS_2 flake by EF-SCN. a-c, SEM image (**a**), SHG mapping image (**b**), and angle-resolved SHG patterns (**c**) of single-crystal MoS_2 flakes with low DES condition. **d-f**, SEM image (**d**), SHG mapping image (**e**), and angle-resolved SHG patterns (**f**) of poly-crystal MoS_2 flakes with high DES condition. **g**,**h**, SEM image (**g**) and corresponding angle-resolved SHG patterns (**h**) of single-crystal MoS_2.

(iv) Faceting of flake edges

If the growth time is prolonged such that oxygen release from HfO_2 is sufficiently reduced, the etching effect acting on the MoS_2 flake edges would be substantially weakened. In this case, as noted by the reviewer, the flake shape may follow the pattern geometry due to anisotropic etching (Extended Data Fig. 1e). However, in most EF-SCN results, including those shown in Fig. R19-21, it is clearly observed that the flake morphology is consistent with the intrinsic crystallographic orientation of MoS_2. In addition, Fig. R10 examines the growth behavior when the MoS_2 epitaxial growth direction is not aligned with the barrier pattern orientation.

As shown in Fig. R10a and R10b, when the epitaxial growth direction and the barrier pattern direction are aligned, triangular MoS_2 flakes with clean edges are formed. This is a characteristic feature consistently observed in the single-nucleation regime of EF-SCN. In contrast, when the epitaxial direction and the barrier pattern are misaligned by 15° or 60°, the crystallographic orientation of the TMD follows epitaxial growth, while the etching process is influenced by the barrier pattern direction (Fig. R10c-f). Since the two directions are not aligned, the flake edges are no longer clean; instead, they consist of rough edges composed of facets corresponding to the MoS_2 crystallographic directions. Therefore, the triangular flakes with clean edges observed in typical EF-SCN results can be interpreted as well-aligned single-crystal MoS_2. Even in cases where rough edges are formed due to misalignment, the fact that the facet orientations remain consistent within a entire flake strongly suggests that these flakes are also single-crystalline.

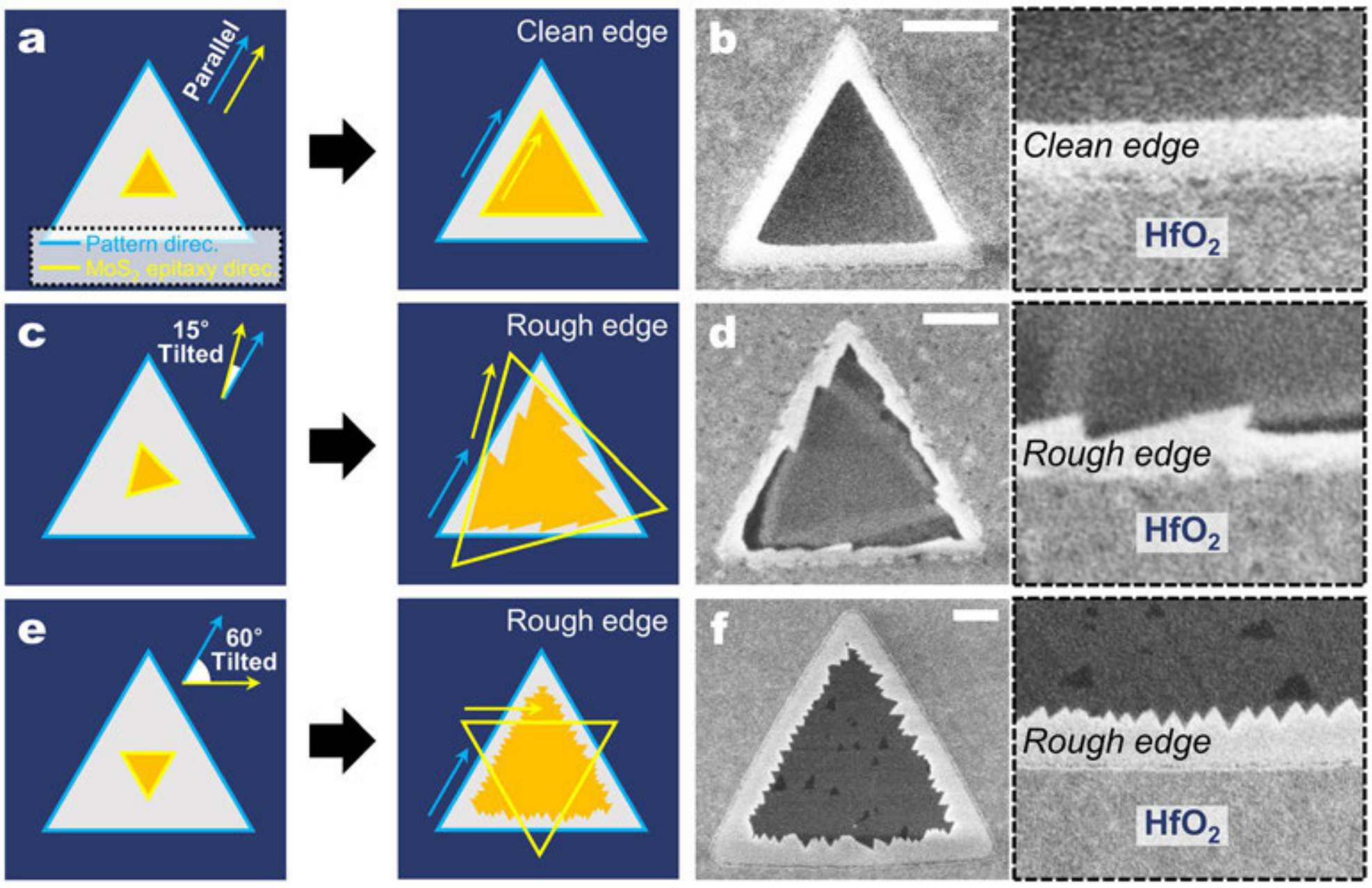

Fig. R10 | Discussion on the relationship between MoS_2 epitaxial growth direction and HfO_2 barrier pattern direction. a,**b**, Schematic (**a**) and SEM images (**b**) of MoS_2 growth when the epitaxial growth direction and the barrier pattern direction are parallel. **c**-**f**, Schematic and SEM images of MoS_2 growth at misalignment angles of 15° (**c**, **d**) and 60° (**e**, **f**) between the epitaxial growth direction and the barrier pattern direction. Scale bars, 1 μm.

(v) Bilayer flake orientation alignment

In layer-by-layer growth of MoS_2, it is generally known that bilayer MoS_2 flakes grow with their crystallographic orientation aligned to that of the underlying monolayer MoS_2. As shown in Fig. R18a, when single-crystal MoS_2 and subsequent bilayer flakes are grown via EF-SCN on an amorphous SiO_2 substrate, the bilayer flakes within a given pattern exhibit identical orientations. This indicates that the monolayer MoS_2 formed by EF-SCN is a single-crystal, and consequently, the bilayer flakes are also aligned in a single crystallographic direction.

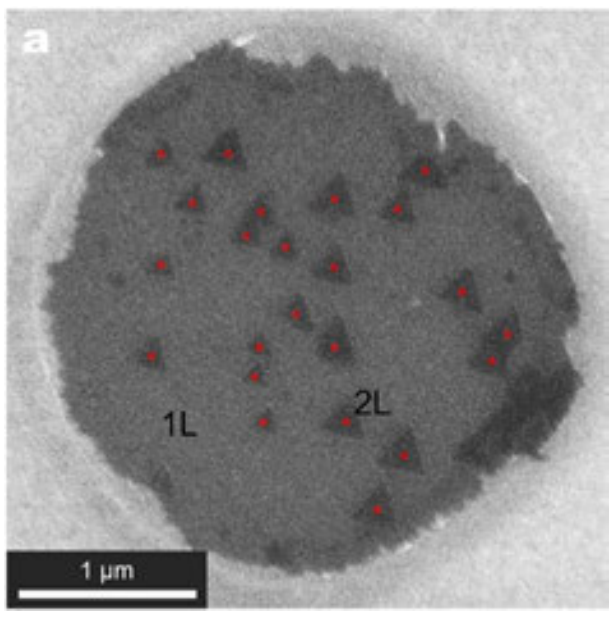

Fig. R18a

[Action in Manuscript]

i) We have added **Extended Data Fig. 2,3 (p. 24, 25)** and **Supplementary Fig. 3,7 (SI p. 4,8)** to verify the single-crystallinity of MoS_2 flake grown by EF-SCN method.

(p. 24) Extended Data Fig. 2 | TEM and atomic resolution AFM measurements of single-crystal MoS_2 flake by EF-SCN. a, TEM image of single-crystal MoS_2 flake fully suspended over a TEM grid hole. b, Diffraction pattern image obtained from the single-crystal in a. c, HAADF-STEM images acquired at S1, S2, and S3 positions marked in a, showing consistent atomic lattice orientation across the flake. d, Representative atomic resolution AFM image of single-crystal MoS_2 flake by EF-SCN. e, AFM and corresponding FFT pattern images of single-crystal MoS_2.

(p. 25) Extended Data Fig. 3 | SHG mapping and angle-resolved SHG pattern of single-crystal MoS_2 flake by EF-SCN. a-c, SEM image (a), SHG mapping image (b), and angle-resolved SHG patterns (c) of single-crystal MoS_2 flakes with low DES condition. d-f, SEM image (d), SHG mapping image (e), and angle-resolved SHG patterns (f) of poly-crystal MoS_2 flakes with high DES condition. g,h, SEM image (g) and corresponding angle-resolved SHG patterns (h) of single-crystal MoS_2.

(SI p. 4) Supplementary Fig. 3 | Relationship between MoS_2 epitaxial growth direction and HfO_2 barrier pattern direction. a,b, Schematic (a) and SEM images (b) of MoS_2 growth when the epitaxial growth direction and the barrier pattern direction are parallel. c-f, Schematic and SEM images of MoS_2 growth at misalignment angles of 15° (c, d) and 60° (e, f) between the epitaxial growth direction and the barrier pattern direction. Scale bars, 1 μm.

(SI p. 8) Supplementary Fig. 7 | 2D MoS_2 EF-SCN on amorphous substrate. a, SEM images of 2D MoS_2 EF-SCN on amorphous SiO_2 substrate, corresponding to the enlarged views of Fig. 2d (i-ix). Small bilayer MoS_2 flakes (dark contrast) are observed on the monolayer MoS_2 surface, exhibiting a consistent crystallographic orientation within each pattern site.

ii) We have modified **manuscript (p. 4-5, 7)** to provide comprehensive structural characterizations confirming the single-crystallinity of MoS_2 flake grown by EF-SCN method.

(p. 4-5) "The diffraction pattern obtained from a MoS_2 flake suspended over a TEM grid hole (Extended Data Fig. 2a) exhibits a typical hexagonal spot pattern of single-crystal MoS_2, rather than multiple spots or ring patterns (Extended Data Fig. 2b). Furthermore, as shown in Extended Data Fig. 2c, high-angle annular dark-field scanning transmission electron microscopy (HAADF-STEM) images acquired at the center (S1), edge (S2), and corner (S3) positions of the flake reveal consistent crystallographic orientation across all regions, aligned with the diffraction pattern. These results confirm that the entire flake region has a consistent crystallographic orientation, demonstrating its single-crystal nature. In addition, Extended Data Fig. 2d, e present atomic resolution AFM measurements, where a well-defined periodic hexagonal lattice characteristic of MoS_2 is clearly observed across the entire scanned area. AFM and corresponding fast Fourier transform (FFT) analyses performed at three arbitrary locations within a flake confirm identical crystallographic orientation. Together, these TEM and atomic resolution AFM results verify that the MoS_2 flakes grown by EF-SCN are single-crystalline and possess high structural quality.", "Extended Data Fig. 3 shows second-harmonic generation (SHG) analyses of single-crystal MoS_2 grown by EF-SCN. In the case of single nucleation, a single triangular flake is observed in SEM image, and the absence of grain boundaries results in uniform SHG intensity mapping, along with a well-defined six-petal pattern in angle-resolved SHG, characteristic of single-crystal MoS_2 (Extended Data Fig. 3a-c). In contrast, under multiple nucleation conditions, multiple domains and irregular flake morphologies are observed in SEM image, accompanies by significant intensity drop at grain boundaries in SHG mapping. The coexistence of domains with different orientations results in an isotropic SHG intensity distribution rather than a six-petal pattern (Extended Data Fig. 3d-f). The barrier patterns in EF-SCN are aligned with the epitaxial growth direction of MoS_2 on miscut sapphire. Accordingly, SHG measurements at six arbitrary positions within the single-crystal MoS_2 array consistently exhibit six-petal patterns with similar crystallographic orientations (Extended Data Fig. 3g, h). The small angular variations ($\pm$3°) is attributed to slight misalignment arising from epitaxial growth at a relatively low temperature (750 °C). Furthermore, when the barrier pattern direction is misaligned with the epitaxial growth direction, rough edges aligned with the intrinsic crystallographic orientation are observed near the barrier, instead of the clean triangular flake typical of EF-SCN (Supplementary Fig. 3). These results indicate that the orientation of triangular single-crystal MoS_2 in EF-SCN is determined by the epitaxial growth direction rather than the barrier pattern geometry."

(p. 7) "Fig. 2d shows optical microscope (OM) and SEM images of uniform single-crystal growth on an amorphous SiO_2 substrate over a 400 μm area. As shown in the SEM images in Supplementary Fig. 7, bilayer MoS_2 flakes (dark contrast) grown at nine random pattern sites in Fig. 2d exhibit consistent orientation, indicating that the underlying monolayer MoS_2 is single-crystalline. However, due to the amorphous substrate, the crystal orientations across different sites are random."

(6) To evaluate the reliability of this method, what is the statistical performance distribution of FET devices fabricated on the large-area TMD single-crystal arrays?

[Author reply]

To verify the reliability of single-crystal MoS_2 growth by EF-SCN, we assessed device-to-device performance variation using FETs fabricated from line-shaped single-crystal MoS_2 arrays. As illustrated in the schematic of Fig. R12a, we fabricated single-crystal single FETs, in which one device was constructed per channel. The devices were fabricated using 10 μm long single-crystal MoS_2 crystals grown at arbitrary locations on an 8 mm × 8 mm miscut sapphire substrate, as depicted in the SEM image of Fig. R12c. The average channel length and width were 6.8 μm and 0.83 μm, respectively, and the cross-sectional structure of the device is presented in the inset schematic.

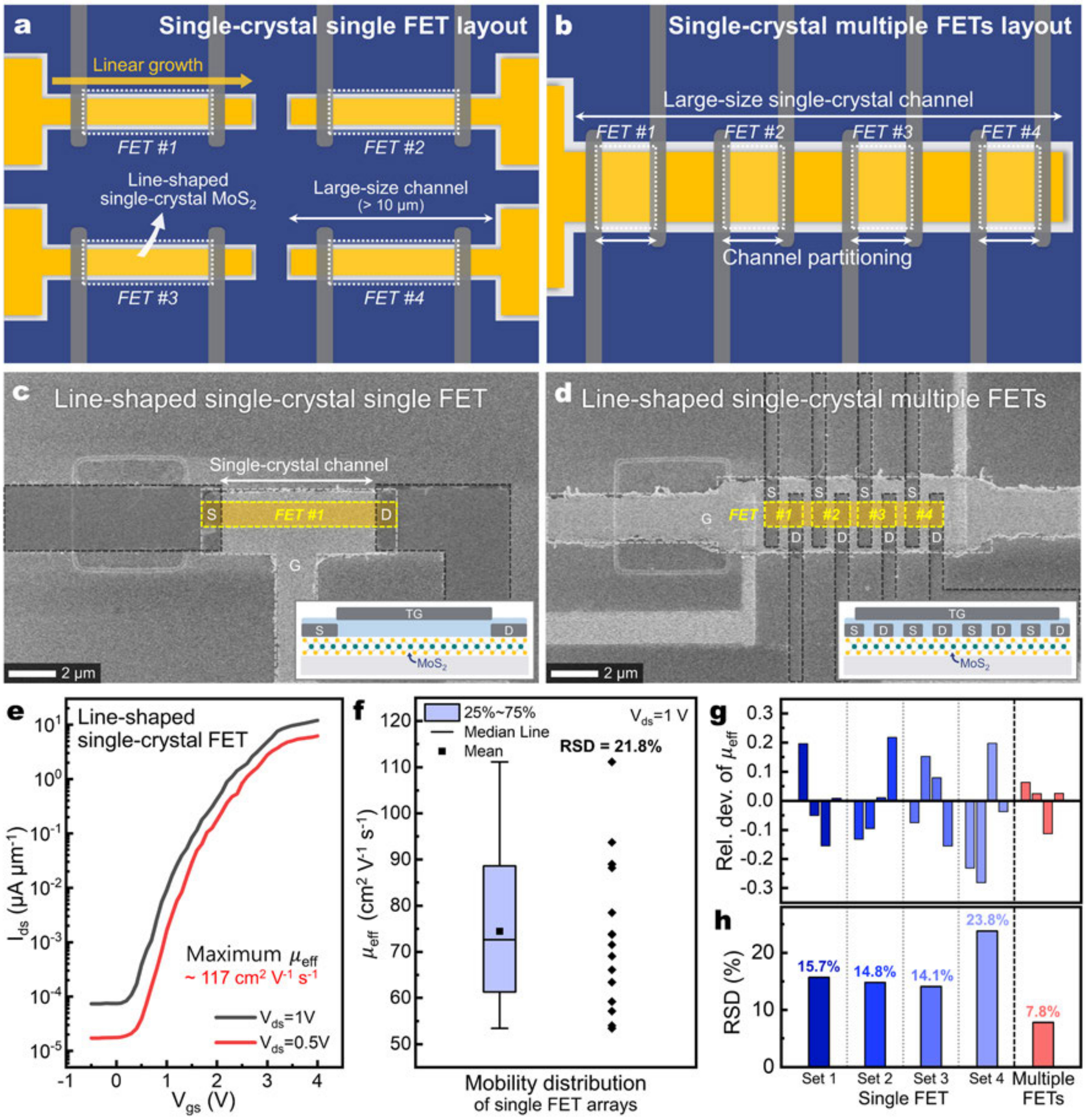

Fig. R12 | Device performance of line-shaped single-crystal single FET and multiple FETs by nucleation-growth decoupling method. a,**b**, Schematic illustrating single-crystal single FET (**a**) and single-crystal multiple FETs (**b**) layouts. **c**,**d**, SEM images of single-crystal MoS_2 single FET (**c**) and multiple FETs (**d**). **e**, Transfer curves of line-shaped single-crystal single FET. **f**, Statistical distribution and box plot of field-effect mobility for single FET arrays. **g**,**h**, Relative mobility deviation (**g**) and relative standard deviation (**h**) of single FET arrays and multiple FETs.

The electrical properties of the fabricated devices are summarized in Fig. R12e, f. Fig. R12e shows the transfer curves of the best-performing device measured at V_{ds} = 1 V and V_{ds} = 0.5 V. The device showed a maximum field-effect mobility of 117 $cm^2V^{-1}s^{-1}$ at V_{ds} = 0.5 V, which surpasses the performance range of previously reported MoS_2 FETs based on confined growth approaches (56-62 $cm^2V^{-1}s^{-1}$)[13,15]. At V_{ds} = 1 V, an average field-effect mobility of 74 $cm^2V^{-1}s^{-1}$ and an RSD of 21.8% were obtained across 16 devices (Fig. R12f), demonstrating a level of variation across devices comparable to previously reported 2D TMD single-crystal FETs (24.18%)[13].

Beyond this, we propose a strategy to further reduce device performance variation by leveraging the line-shaped single-crystals enabled by the nucleation-growth decoupling strategy. Specifically, the single-crystal multiple FET architecture (Fig. R12b) employs multiple FETs integrated within a single channel. We anticipated that devices fabricated using partitioned channels originating from a single-crystal would exhibit lower channel-induced performance variation compared to those fabricated from separate single-crystal channels. To this end, four devices with a channel length of 0.31 µm and width of 0.87 µm were fabricated using a 10 µm long MoS_2 single crystal, as shown in Fig. R12d.

Fig. R12g and R12h summarize the relative deviation and RSD of field-effect mobility across the 16 single FETs (four sets) and the 4 multiple FETs. The analysis revealed that the single-crystal single FETs showed RSDs of 15.7%, 14.8%, 14.1%, and 23.8% across the four sets (21.8% overall), whereas the single-crystal multiple FETs exhibited a markedly reduced RSD of 7.8%. Such a 65% enhancement in mobility uniformity demonstrates that utilizing a single-crystal partitioned into multiple channels, rather than employing individual single-crystals as separate channels for each device, is a more effective strategy for reducing variation across devices.

Collectively, these results highlight the potential of EF-SCN's single-crystal scaling capability for realizing low-variation, high-density 2D FET arrays within an enlarged single-crystal channel. As illustrated in Fig. R13, this capability provides an architectural basis for extending single-crystal 2D FET integration from individual devices toward standard-cell-level layouts, where multiple transistors can share a common single-crystal foundation. Although logic-circuit operation is beyond the scope of the present work, the experimentally demonstrated multiple FETs integration within a single-crystal MoS_2 channel provides an experimental basis for exploring future scaling from 2D FET arrays toward standard-cell and block-level logic integration. In addition, because EF-SCN enables the formation of elongated single-crystal channels that can be partitioned into multiple FETs, this strategy may also be relevant to string-like transistor architectures, such as NAND flash strings, where multiple transistor elements are arranged along an extended channel direction.

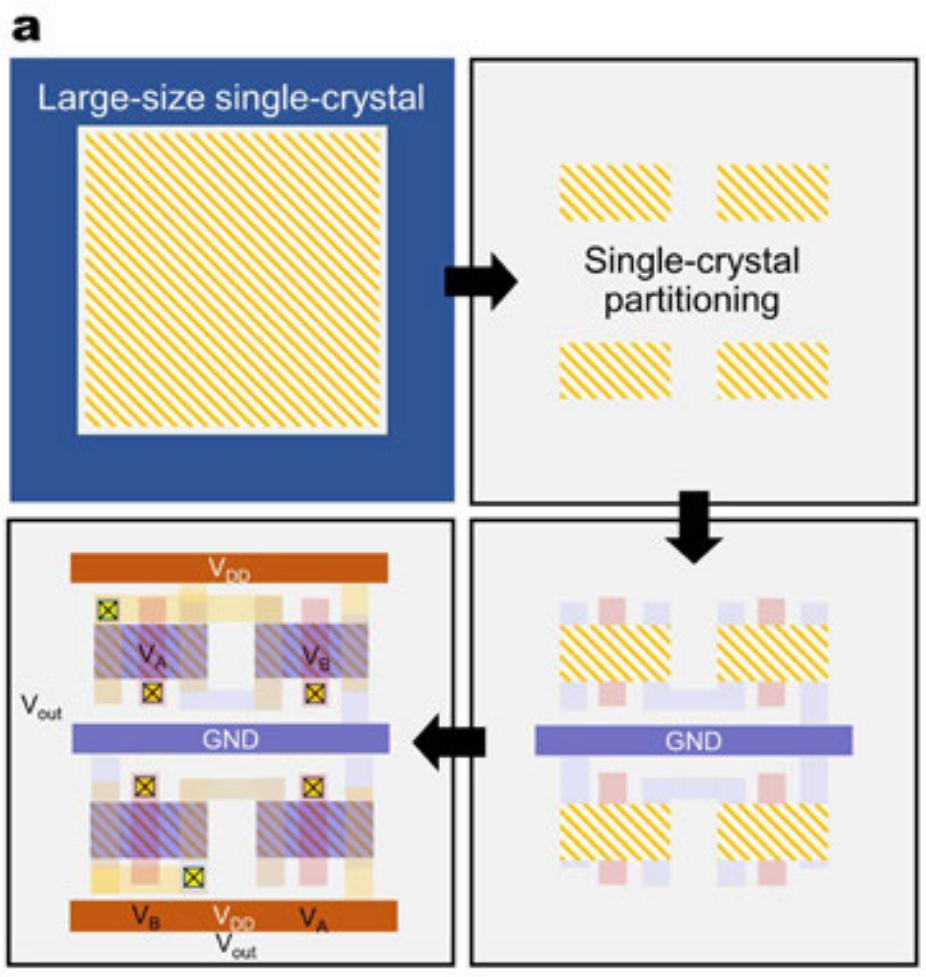

Fig. R13 | Large-size single-crystal partitioning for multiple FETs integration and logic implementation. a, Schematic illustrating a large-size single-crystal domain and its division into multiple channels via single-crystal partitioning, followed by the formation of multiple FETs and their interconnection for logic operation.

[Action in Manuscript]

i) We have revised **Fig. 4 (p. 22)** and added **Extended Data Fig. 9 (p. 31)** to present the statistical performance distribution of single-crystal single FET arrays and single-crystal multiple FETs.

(p. 22) Fig. 4 | 2D TMD single-crystal architectures based on EF-SCN method. a, Schematic of nucleation-growth decoupling strategy for line-shaped single-crystal 2D MoS_2 growth. b, SEM image of line-shaped single-crystal MoS_2 single FET. c, Transfer curve of the line-shaped single FET. d, Statistical distribution and box plot of field-effect mobility for single FET arrays. e-g, Schematic (e, g) and SEM images (f) of Ru AS-ALD on aligned 2D MoS_2-$MoSe_2$ lateral heterostructure for self-aligned ALD contact. The inset graph shows the AFM height profile along the white line. h, i, HAADF-STEM images (h) and transfer curve (i) of self-aligned ALD contact FET.

(p. 31) Extended Data Fig. 9 | Nucleation-growth decoupling strategy and single-crystal multiple FETs. a,b, SEM images showing line-shaped single-crystal MoS_2 growth via nucleation-growth decoupling strategy in initial stage (a) and fully growth stage (b). c,d, Schematic (c) and NCM phase mapping image by AFM (d) of stripe 2D MoS_2-$MoSe_2$

lateral heterostructure. e,f, Schematic illustrating single-crystal single FET (e) and single-crystal multiple FETs (f) layouts. g, SEM image of single-crystal MoS_2 multiple FETs. h,i, Relative mobility deviation (h) and relative standard deviation (i) of single FET arrays and multiple FETs.

ii) We have revised **manuscript (p. 10-11)** to demonstrate superior electrical performance of single-crystal single FET arrays and low-variation single-crystal multiple FETs.

(p. 10-11) "Importantly, a high field-effect mobility of 117 $cm^2V^{-1}s^{-1}$ was achieved, significantly surpassing previously reported values for selectively grown MoS_2 FETs, indicating the excellent electrical properties of single-crystal MoS_2 by EF-SCN[8,9]. In Fig. 4d, the mobility distribution across a total of 16 single FET arrays reveals an average mobility of 74 $cm^2V^{-1}s^{-1}$ with a RSD of 21.8%, demonstrating a performance variation comparable to that of previous 2D TMD single-crystal FETs. ~", "In the case of single-crystal single FETs, the RSD values for each set were 15.7%, 14.8%, 14.1%, and 23.8% (21.8% overall), whereas the single-crystal multiple FETs exhibited a significantly lower RSD of 7.8%. This corresponds to a 65% reduction in mobility variation compared to the single FET arrays, indicating that fabricating multiple channels within a single-crystal is more advantageous in terms of variation than defining a single channel in one single-crystal. ~"

(7) Regarding the spatially programmed lateral heterostructures, what is the atomic sharpness of the interface?

[Author reply]

The MOCVD conditions for MoS_2-$MoSe_2$ lateral heterostructure growth in the EF-SCN system closely resemble those reported in a prior *Nature Communications* study on 2D lateral superlattice AS-ALD[38]. As shown in Fig. R22a, a HAADF-STEM image of the MoS_2-$MoSe_2$ lateral superlattice reveals a sharp interface of less than 2 nm at a line width of ~10 nm. Given the use of comparable growth parameters – including diethyl sulfide (DES), dimethyl selenide (DMSe), molybdenum hexacarbonyl (MHC), and a growth temperature of 700-750 °C – the EF-SCN system provides a viable basis for achieving sharp heterointerfaces.

However, the presence of oxygen released from the etching-flux barrier may inherently degrade the interface quality compared to the prior study. As shown in Fig. R22b, the currently achieved aligned lateral heterostructures exhibit a line width of ~100 nm. Therefore, realizing narrower features with improved interface sharpness will require more refined growth control, including precise tuning of the DES/DMSe supply and oxygen release from the etching-flux barrier.

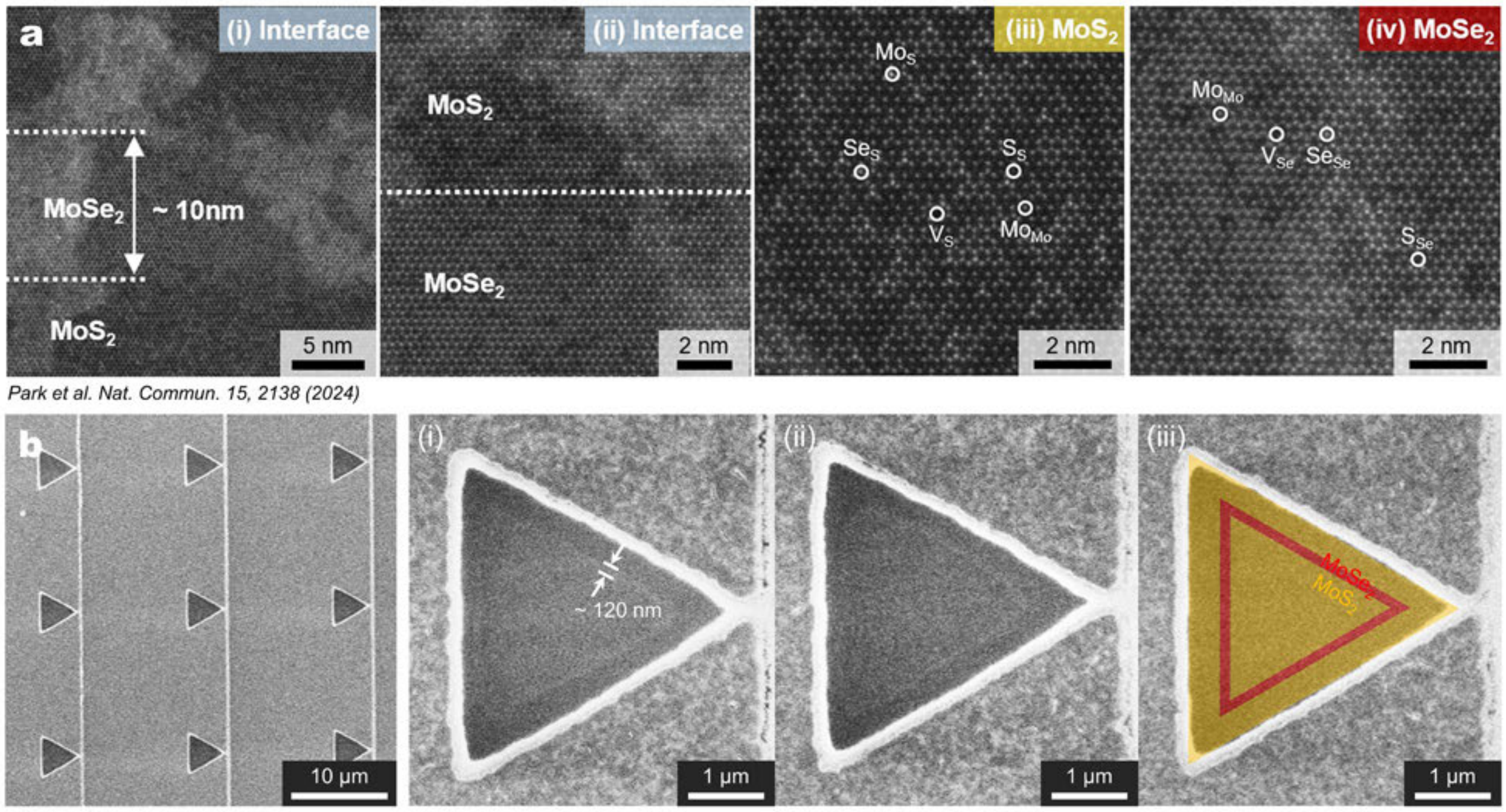

Fig. R22 | Interface quality of 2D MoS_2-$MoSe_2$ lateral heterostructure. a, HAADF-STEM images of MoS_2-$MoSe_2$ lateral heterostructure. **b**, SEM images of aligned MoS_2-$MoSe_2$ lateral superlattices grown by EF-SCN.

[Action in Manuscript]

i) We have added **Extended Data Fig. 10 (p. 32)** to demonstrate the well-aligned MoS_2-$MoSe_2$ lateral heterostructure.

(p. 32) Extended Data Fig. 10 | Alignment of 2D TMD lateral heterostructure for self-aligned ALD contact FET. a-d, Schematic (a) and SEM images (b-d) of aligned 2D MoS_2-$MoSe_2$ lateral heterostructure by EF-SCN method. e, Schematic of self-aligned ALD contact FET with Ru AS-ALD on 2D MoS_2-$MoSe_2$ lateral heterostructure.

ii) We have revised **manuscript (p. 11)** to explain the achievable line width of lateral heterostructure under the EF-SCN system.

(p. 11) "As demonstrated in Extended Data Fig. 10a-d, the EF-SCN system affords exceptional controllability over lateral heterostructure alignment. We successfully synthesized MoS_2-$MoSe_2$ lateral superlattices spanning a wide dimensional range, from 120 nm line widths (Extended Data Fig. 10c) to large-area structures exceeding 5 μm that remain well-aligned within the patterned region (Extended Data Fig. 10d). Such spatially programmed growth enables new contact engineering strategies through strain modulation in the 2D TMD channel."

4. Point-by-Point Responses to Reviewer #4, #5 Comments

Area-selective growth has been widely developed in 2D and nanoscale electronics for precise control of high-quality materials with ideal device performance. Single crystal epitaxial growth of 2D materials has also become very important in further improvement of high-quality materials without twin boundaries and alloys. The research of ASG strategies such as selective chemical vapor deposition in 2D growth is meaningful in the study of 2D channel layers for mass production. This manuscript contributes meaningfully to that direction by introducing an etching-flux mediated single-nucleation method. Oxygen-flux induced by HfO_2 has been applied in the restriction of MoS_2 growth near the nucleation barrier. MoS_2-$MoSe_2$ heterostructures and double-aligned 2D/3D hetero-patterns are realized. The approach yields line-shaped MoS_2, exhibiting excellent electrical performance with a maximum mobility of 72 $cm^2V^{-1}s^{-1}$ and an on/off ratio above 10^8. In addition, a model has been proposed to describe the oxygen flux, oxygen concentration, and MoS_2 nucleation rate within the growth region, which further elaborate the etching behavior of EF-SCN. In my evaluation, the current version of the manuscript shows moderate level of novelty since both oxygen etching behavior and selective growth methods have already been realized in previous studies. Some underlying mechanisms remain unclear that may require further evidence for more convincing conclusions. Below are the list of technical comments that should be addressed before further consideration of this manuscript.

[Author reply]

We sincerely thank the reviewers for their insightful comments regarding the novelty and mechanistic interpretation of the EF-SCN approach. We appreciate the reviewer's perspective that both oxygen-assisted etching and area-selective growth strategies have previously been investigated in 2D TMD systems. In this revision, we therefore focused on clarifying the distinct nucleation-control mechanism of EF-SCN and the unique capabilities enabled by this growth mode.

To systematically compare EF-SCN with representative selective-growth approaches[13,15,29-32], we constructed the benchmark analysis summarized in Table R2. The comparison evaluates seed growth, confined growth, and EF-SCN using consistent metrics including process compatibility, device performance, and achievable application scope. Through this analysis, we aim to clarify that EF-SCN introduces a distinct nucleation-control regime beyond conventional area-selective growth approaches.

		Seed growth [29-32]	Confined growth [13,15]		EF-SCN [Our work]	
A	Selectivity origin	Surface difference (Nucleation promoter)	Surface difference (Nucleation inhibitor)		Etching-flux	
B	TMD growth temperature (°C)	650-750	750 [13]	385 [15]	750	500 [Fig. R7]
	TMD growth method	Powder CVD or Sulfurization	Powder CVD		MOCVD	
	Mobility ($cm^2V^{-1}s^{-1}$)	5-50	62 [13]	56 [15]	117 [Fig. R12]	
C	**Nucleation initiation**	**Not spatially deterministic**	**Not spatially deterministic**		**Deterministic at center**	
	**Effective single-crystal size (μm)**	**Limited single domain**	**1-2** [13]	**0.2-0.5** [15]	**7 (Tri.)** [Fig. R7]	**10 (Line)** [Fig. R12]
	**Orientation alignment**	**Not controlled**	**Not controlled**		**Possible** [Fig. R14, R21]	
	**Advanced applications** beyond basic FET	-	**Valleytronics device** [13] with single-crystal *vertical heterostructure*		**Self-aligned ALD contact FETs** [Fig. R14] with *aligned lateral heterostructure*	
			**CFET** [15] with single-crystal *3D integration*		**Single-crystal multiple FETs** [Fig. R12] with *large-size single-crystal*	

Table R2 | Benchmark table of 2D monolayer MoS_2 area-selective growth. Comparison between seed growth[29-32], confined growth[13,15], and EF-SCN method.

A. Selectivity origin

Conventional selective-growth approaches primarily rely on intrinsic surface differences between heterogeneous substrates to achieve selectivity. In seed growth, nucleation preferentially occurs near predefined nucleation-promoting regions such as noble metals or Mo precursor sites. In confined growth, differences in binding energy between the barrier and growth regions are utilized to suppress growth outside the confined region while allowing growth within the designated area.

However, in both approaches, the nucleation probability remains broadly distributed across energetically similar edge or terrace sites within the growth region, making deterministic localization of nucleation challenging. In contrast, EF-SCN does not primarily rely on heterogeneous surface selectivity. Instead, the etching-flux creates a spatially biased nucleation landscape within the confined region, substantially enhancing the nucleation probability near the center of the growth region while reducing off-center nucleation events. This mechanistic distinction enables deterministic center nucleation together with crystallographic alignment control.

This distinction is schematically illustrated in Fig. R23. As shown in Fig. R23a, conventional oxygen-assisted growth primarily utilizes oxygen etching to control nucleation density and grain size[16-19], but does not provide deterministic spatial control over nucleation position. Confined growth (Fig. R23b) relies on conventional selective-growth mechanisms based on binding-energy differences between growth and barrier regions[13]. In contrast, EF-SCN (Fig. R23c) utilizes etching-flux-mediated spatial modulation of nucleation probability, enabling deterministic control over both nucleation position and crystallographic orientation. This capability establishes a new pathway toward spatially programmed growth of single-crystal 2D TMD systems.

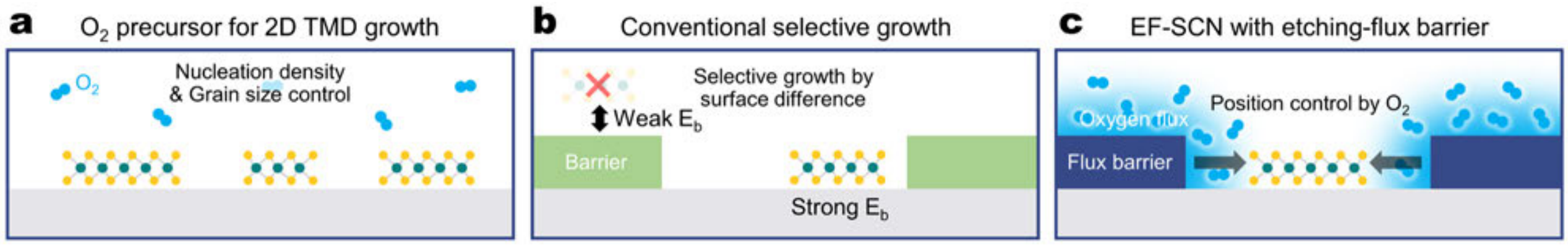

Fig. R23 | Microscale spatial control of nucleation sites based on oxygen flux in EF-SCN. a-c, Schematic illustrating 2D TMD growth using oxygen precursor (a), conventional selective growth of 2D TMD (b), and EF-SCN system using etching-flux barrier for deterministic nucleation (c).

B. Process compatibility of EF-SCN method

Most reported seed-growth approaches employ high growth temperatures in the range of 650-750 °C, whereas confined growth based on edge nucleation has demonstrated effective single-crystal growth at 385 °C. However, the EF-SCN results in our previous report required relatively high temperatures (~750 °C) to generate sufficient etching-flux.

To address this concern, we performed additional experiments and identified a low-temperature etching-flux regime that enables deterministic single-crystal MoS_2 growth even at 500 °C (Fig. R7). These results demonstrate that the EF-SCN mechanism is not fundamentally restricted to high-temperature operation and can be extended toward low-thermal-budget processes.

In addition, while most previously reported 2D TMD area-selective growth studies relied on powder CVD processes, EF-SCN is implemented using MOCVD, which provides a well-controlled and scalable growth environment. To further validate the process compatibility and robustness of EF-SCN, we additionally demonstrate a valid process window for deterministic single-crystal growth (Fig. R16) and large-area uniform single-crystal growth on amorphous substrates (Fig. R17).

C. Advanced single-crystal device capabilities enabled by EF-SCN

i) Single-crystal multiple FETs with large-size single-crystal growth

Conventional 2D TMD ASG methods exhibit uniform nucleation probability throughout the growth region, limiting small-size single-crystal growth of 1 μm. In contrast, the EF-SCN approach effectively suppresses undesired nucleation by using the etching-flux, enabling large-size single-crystal growth reaching 7 μm in triangular patterns and 10 μm in linear patterns (Fig. R7 and R12). This single-crystal scaling capability enables the integration of multiple FETs within a single-crystal channel, rather than merely realizing a single FET within a channel.

Fig. R12a and R12b illustrate the concepts of typical single-crystal single FET arrays and single-crystal multiple FETs with large-size line-shaped MoS_2 channel. As shown in the SEM images in Fig. R12c and R12d, single FET arrays were fabricated within individual channels (Fig. R12c), while multiple FETs were integrated within a single channel (Fig. R12d). In the case of the single-crystal single FET, an excellent field-effect mobility of 117 $cm^2V^{-1}s^{-1}$ was observed, as shown in Fig. R12e, exceeding the previously reported values by confined growth (56-62 $cm^2V^{-1}s^{-1}$)[13,15]. Statistical analysis across 16 single FET arrays yields a mean mobility of 74 $cm^2V^{-1}s^{-1}$ and mobility RSD of 21.8% (Fig. R12f), comparable to performance variations of confined growth[13].

Importantly, the single-crystal multiple FETs exhibit improved uniformity: RSD values for the four sets of single FETs ranged from 14.1 to 23.8% (21.8% overall), while the multiple FETs yielded a substantially reduced RSD of 7.8% (Fig. R12g and R12h), corresponding to a 65% improvement in mobility uniformity. This finding indicates that integrating multiple devices within a single channel is a more effective strategy for minimizing device performance variation than the typical single FET in one channel approach.

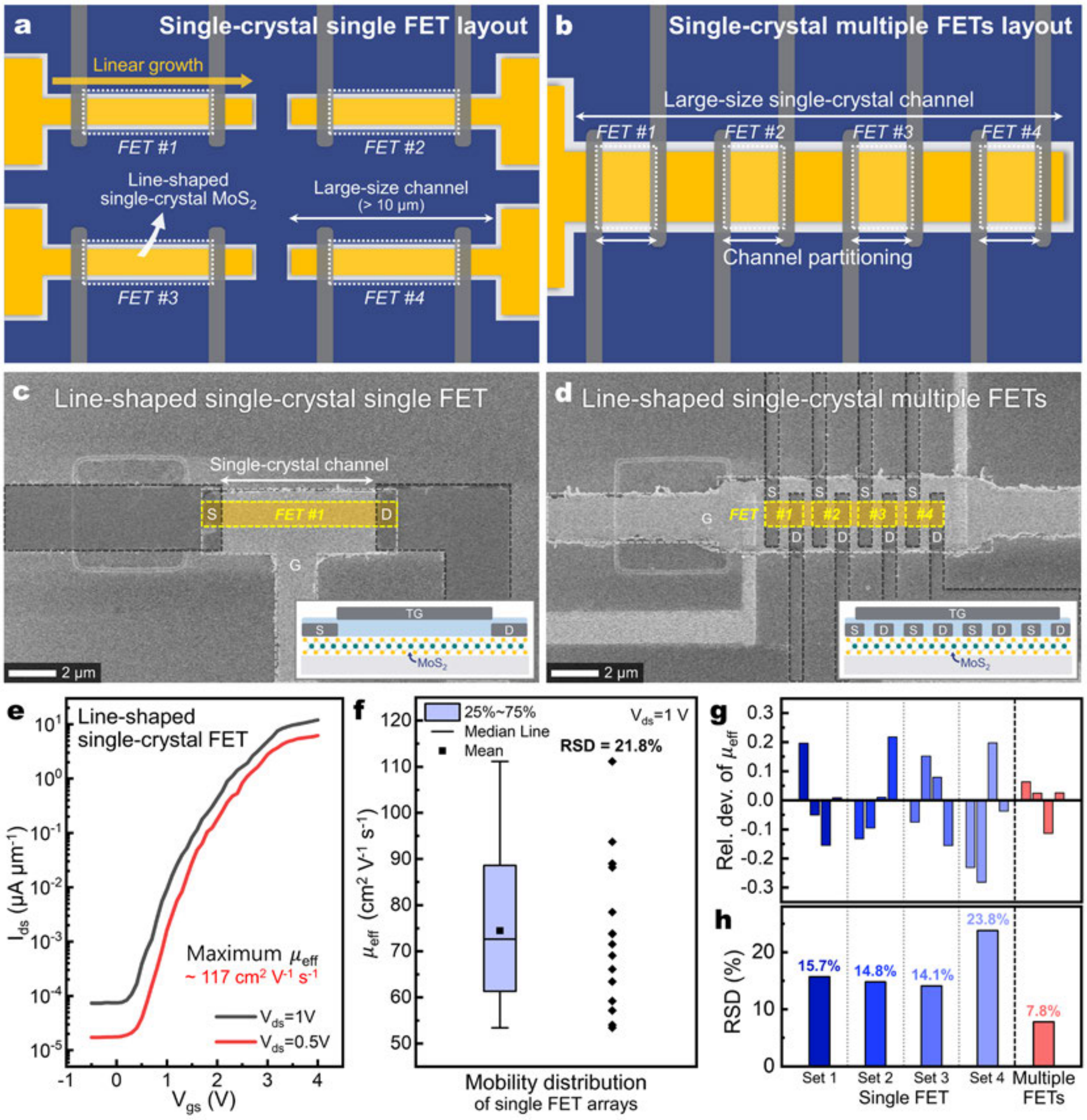

Fig. R12 | Device performance of line-shaped single-crystal single FET and multiple FETs by nucleation-growth decoupling method. a,**b**, Schematic illustrating single-crystal single FET (**a**) and single-crystal multiple FETs (**b**) layouts. **c**,**d**, SEM images of single-crystal MoS_2 single FET (**c**) and multiple FETs (**d**). **e**, Transfer curves of line-shaped single-crystal single FET. **f**, Statistical distribution and box plot of field-effect mobility for single FET arrays. **g**,**h**, Relative mobility deviation (**g**) and relative standard deviation (**h**) of single FET arrays and multiple FETs.

Collectively, these results highlight the potential of EF-SCN's single-crystal scaling capability for realizing low-variation, high-density 2D FET arrays within an enlarged single-crystal channel. As illustrated in Fig. R13, this capability provides an architectural basis for extending single-crystal 2D FET integration from individual devices toward standard-cell-level layouts, where multiple transistors can share a common single-crystal foundation. Although logic-circuit operation is beyond the scope of the present work, the experimentally demonstrated multiple FETs integration within a single-crystal MoS_2 channel provides an experimental basis for exploring future scaling from 2D FET arrays toward standard-cell and block-level logic integration. In addition, because EF-SCN enables the formation of elongated single-crystal channels that can be partitioned into multiple FETs, this strategy may also be relevant to string-like transistor architectures, such as NAND flash strings, where multiple transistor elements are arranged along an extended channel direction.

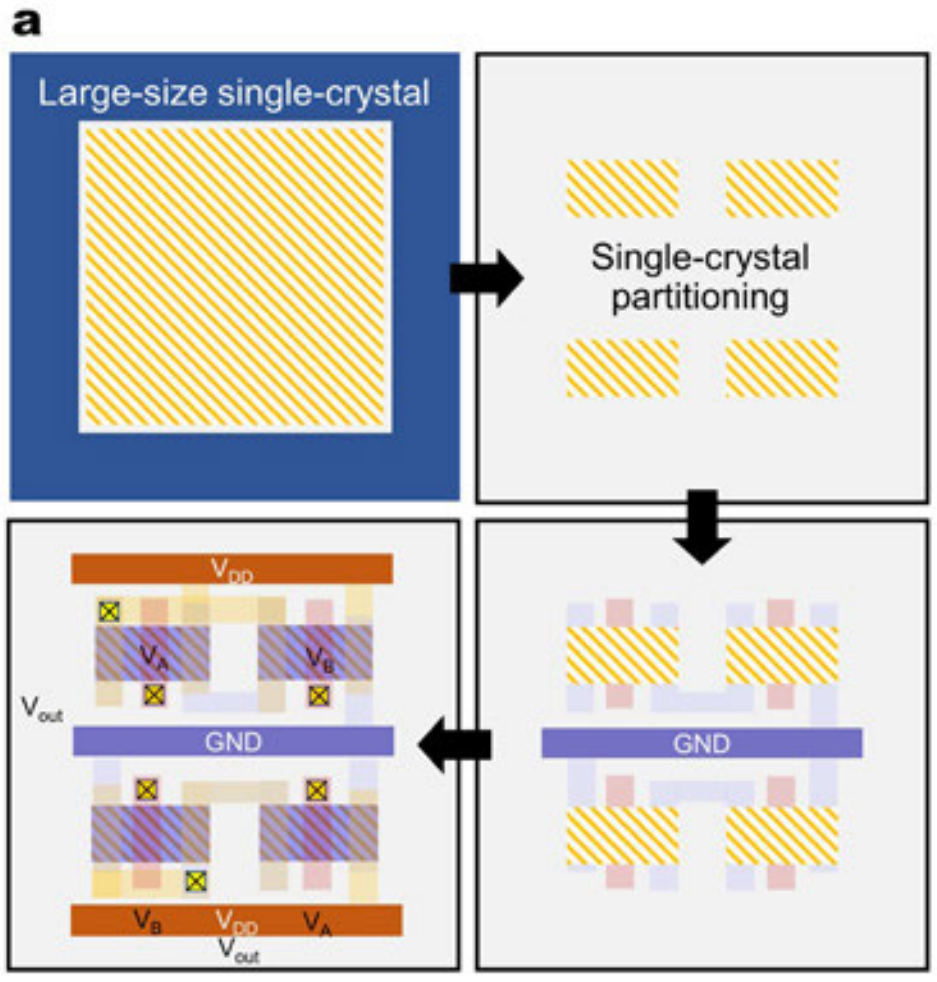

Fig. R13 | Large-size single-crystal partitioning for multiple FETs integration and logic implementation. a, Schematic illustrating a large-size single-crystal domain and its division into multiple channels via single-crystal partitioning, followed by the formation of multiple FETs and their interconnection for logic operation.

ii) Self-aligned ALD contact devices with self-aligned lateral heterostructure

Since conventional seed growth and confined growth strategies cannot fundamentally control the nucleation onset position and crystallographic orientation, previous reports have mainly demonstrated basic FETs, CFETs, and valleytronic devices that do not require deterministic nucleation. In contrast, EF-SCN simultaneously controls both the nucleation position and orientation, enabling the realization of advanced single-crystal architectures based on spatially programmed in-plane growth. Using the aligned 2D MoS_2-$MoSe_2$ lateral heterostructures, self-aligned ALD contact devices can be realized, providing a platform for spatially controlled strain modulation, doping engineering, and advanced contact processing in 2D TMD channels (Fig. R14).

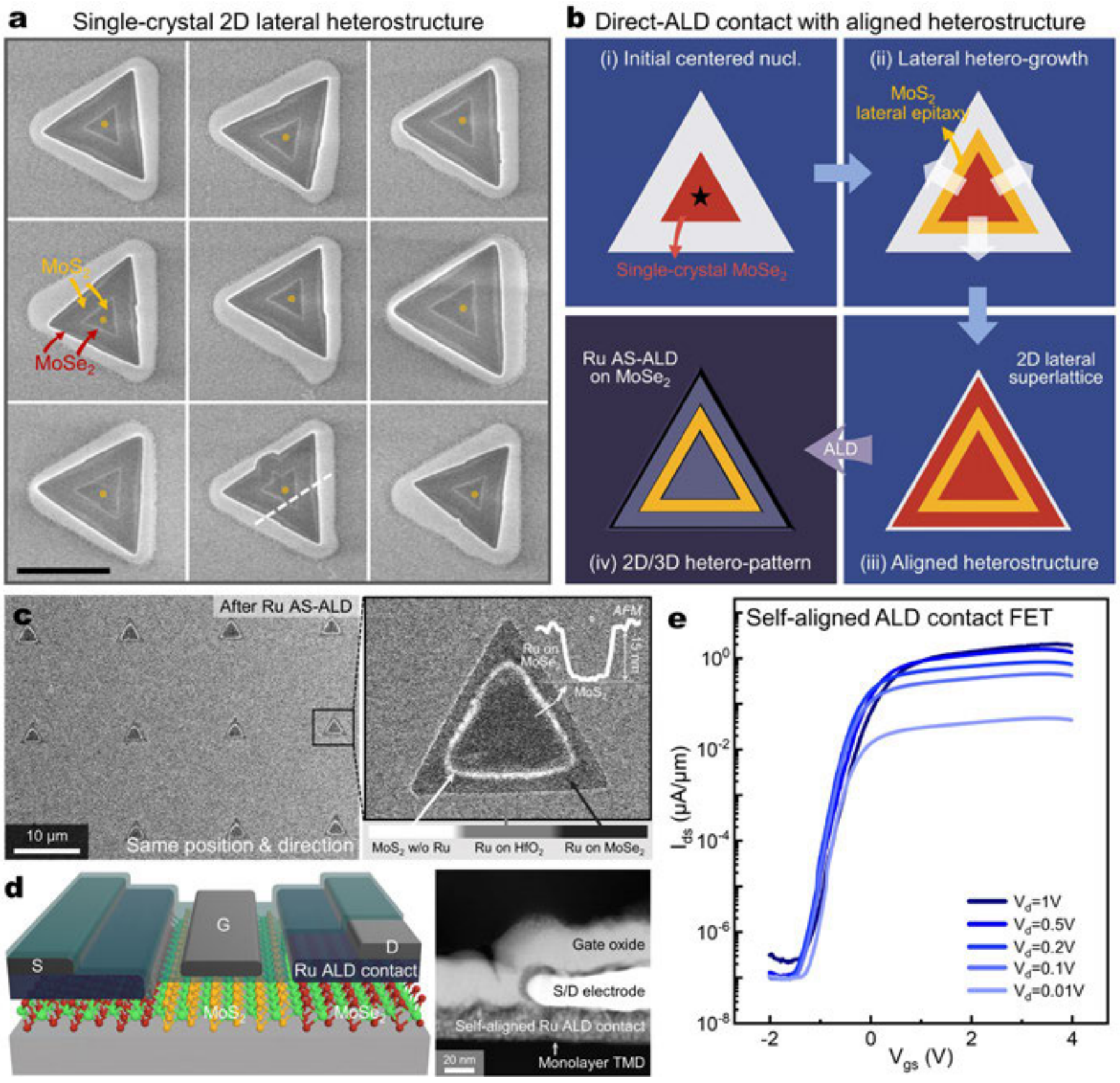

Fig. R14 | Self-aligned ALD contact device with self-aligned lateral heterostructure by EF-SCN. a, SEM images of aligned single-crystal MoS_2-$MoSe_2$ lateral superlattice. Scale bars, 5 µm. **b**-**d**, Schematic, SEM images, and HAADF-STEM image of Ru AS-ALD on aligned single-crystal 2D heterostructures. **e**, Transfer curve of self-aligned ALD contact FET.

[Action in Manuscript]

i) We have revised **Fig. 4 (p. 22)** and added **Extended Data Fig. 9,10 (p. 31,32), Supplementary Table 4 (SI p. 20)** to demonstrate the realization of single-crystal multiple FETs based on nucleation-growth decoupling, the implementation of self-aligned ALD contact FETs, and a benchmark of the EF-SCN method against conventional seed growth and confined growth approaches for 2D monolayer MoS_2 ASG, respectively.

(p. 22) Fig. 4 | 2D TMD single-crystal architectures based on EF-SCN method. a, Schematic of nucleation-growth decoupling strategy for line-shaped single-crystal 2D MoS_2 growth. b, SEM image of line-shaped single-crystal MoS_2 single FET. c, Transfer curve of the line-shaped single FET. d, Statistical distribution and box plot of field-effect mobility for single FET arrays. e-g, Schematic (e, g) and SEM images (f) of Ru AS-ALD on aligned 2D MoS_2-$MoSe_2$ lateral heterostructure for self-aligned ALD contact. The inset graph shows the AFM height profile along the white line. h, i, HAADF-STEM images (h) and transfer curve (i) of self-aligned ALD contact FET.

(p. 31) Extended Data Fig. 9 | Nucleation-growth decoupling strategy and single-crystal multiple FETs. a,b, SEM images showing line-shaped single-crystal MoS_2 growth via nucleation-growth decoupling strategy in initial stage (a) and fully growth stage (b). c,d, Schematic (c) and NCM phase mapping image by AFM (d) of stripe 2D MoS_2-$MoSe_2$ lateral heterostructure. e,f, Schematic illustrating single-crystal single FET (e) and single-crystal multiple FETs (f) layouts. g, SEM image of single-crystal MoS_2 multiple FETs. h,i, Relative mobility deviation (h) and relative standard deviation (i) of single FET arrays and multiple FETs.

(p. 32) Extended Data Fig. 10 | Alignment of 2D TMD lateral heterostructure for self-aligned ALD contact FET. a-d, Schematic (a) and SEM images (b-d) of aligned 2D MoS_2-$MoSe_2$ lateral heterostructure by EF-SCN method. e, Schematic of self-aligned ALD contact FET with Ru AS-ALD on 2D MoS_2-$MoSe_2$ lateral heterostructure.

(SI p. 20) Supplementary Table 4 | Benchmark table of 2D monolayer MoS_2 ASG. Comparison between seed growth[16-19], confined growth[20,21], and EF-SCN method.

ii) We have improved **manuscript (p. 10-11)** to demonstrate superior electrical performance of single-crystal single FET arrays and low-variation single-crystal multiple FETs.

(p. 10-11) "Importantly, a high field-effect mobility of 117 $cm^2V^{-1}s^{-1}$ was achieved, significantly surpassing previously reported values for selectively grown MoS_2 FETs, indicating the excellent electrical properties of single-crystal MoS_2 by EF-SCN[8,9]. In Fig. 4d, the mobility distribution across a total of 16 single FET arrays reveals an average mobility of 74 $cm^2V^{-1}s^{-1}$ with a RSD of 21.8%, demonstrating a performance variation comparable to that of previous 2D TMD single-crystal FETs. ~", "In the case of single-crystal single FETs, the RSD values for each set were 15.7%, 14.8%, 14.1%, and 23.8% (21.8% overall), whereas the single-crystal multiple FETs exhibited a significantly lower RSD of 7.8%. This corresponds to a 65% reduction in mobility variation compared to the single FET arrays, indicating that fabricating multiple channels within a single-crystal is more advantageous in terms of variation than defining a single channel in one single-crystal. ~"

iii) We have reworked **manuscript (p. 11-12)** to illustrate the self-aligned ALD contact FETs enabled by spatially aligned 2D MoS_2-$MoSe_2$ lateral heterostructures.

(p. 11-12) "Fig. 4e-f present a schematic and SEM images illustrating the fabrication of self-aligned ALD contact FETs. When Ru ALD is conducted on the 2D MoS_2-$MoSe_2$ lateral heterostructures with precisely controlled position and orientation via the EF-SCN approach, selective deposition occurs on the $MoSe_2$ region due to the local strain distribution (Fig. 4e, g). As shown in Fig. 4f, a well-aligned 2D/3D hybrid structure is achieved at the array level, and AFM measurements confirm the formation of 15 nm-thick Ru film."

iv) We have introduced **Supplementary Note 6 (SI p. 34)** to discuss the novelty and advantages of EF-SCN compared with conventional 2D TMD ASG methods, particularly in terms of process compatibility and advanced single-crystal device implementation.

(SI p. 34) "Conventional 2D TMD ASG has been explored primarily through two approaches: seed growth[16-19] and confined growth[20,21]. In this note, we elaborate on the novelty and advantages of EF-SCN in terms of process compatibility and advanced single-crystal device implementation, based on the benchmarking presented in Supplementary Table 4. ~"

(1) One of the main conclusions in the manuscript is the single nucleation achieved under medium L_{growth} and middle R_{growth}. However, it is important to differentiate between single nucleation and multiple nucleation "film" within the triangular region in the nucleation site. Current results only show AFM height profiles to identify the grain boundary in multiple nucleation images. For polycrystalline films, the height profile can also be smooth if the grain boundary is in atomic scale. Would the oxygen etching-flux actually perform etching after the multiple nucleation film growth? Therefore, further evidence should be provided in order to clarify whether it is really single crystal through single nucleation growth.

(4) It seems that the triangle orientation is randomly distributed in multiple nucleation scheme. It would be important to know whether the triangle orientation is uniform or not in single nucleation condition across the nucleation sites. This can also help to clarify whether it is single nucleation or multiple nucleation to form the film first then etched away by the oxygen flux.

(6) For the fully filled crystal line in Extended Data Fig. 10, it would also be important to use methods other than AFM to further confirmation of the single crystal MoS_2. Methods such as TEM would provide more definitive structural validation.

This comment raises an important question regarding whether the flakes formed via EF-SCN are indeed single-crystal. Therefore, we regarded this comment as a critical task in the revision process and performed rigorous characterizations. Since previous grain boundary observation by AFM is insufficient to verify single-crystallinity, we additionally conducted a comprehensive analysis using TEM, atomic-resolution AFM, second-harmonic generation (SHG), faceting of flake edges, and bilayer flake orientation alignment.

(i) TEM

Fig. R19 presents TEM analysis results of a single-crystal MoS_2 flake grown by EF-SCN. After selective growth of MoS_2, a PMMA layer was coated, and the flakes were transferred onto a quantifoil TEM grid via a wet-transfer process, followed by PMMA removal. The optical microscopy (OM) image in Fig. R19a shows MoS_2 flakes transferred onto the TEM grid; except for one flake, the others were folded or torn. Fig. R19b shows a low-magnification TEM image of a flake successfully transferred across a hole, and Fig. R19c presents the corresponding diffraction pattern. In the case of polycrystalline MoS_2 films containing grain boundaries, additional spots or ring patterns are typically observed. However, Fig. R19c exhibits only a hexagonal spot pattern, strongly indicating that the flake is a single-crystal MoS_2.

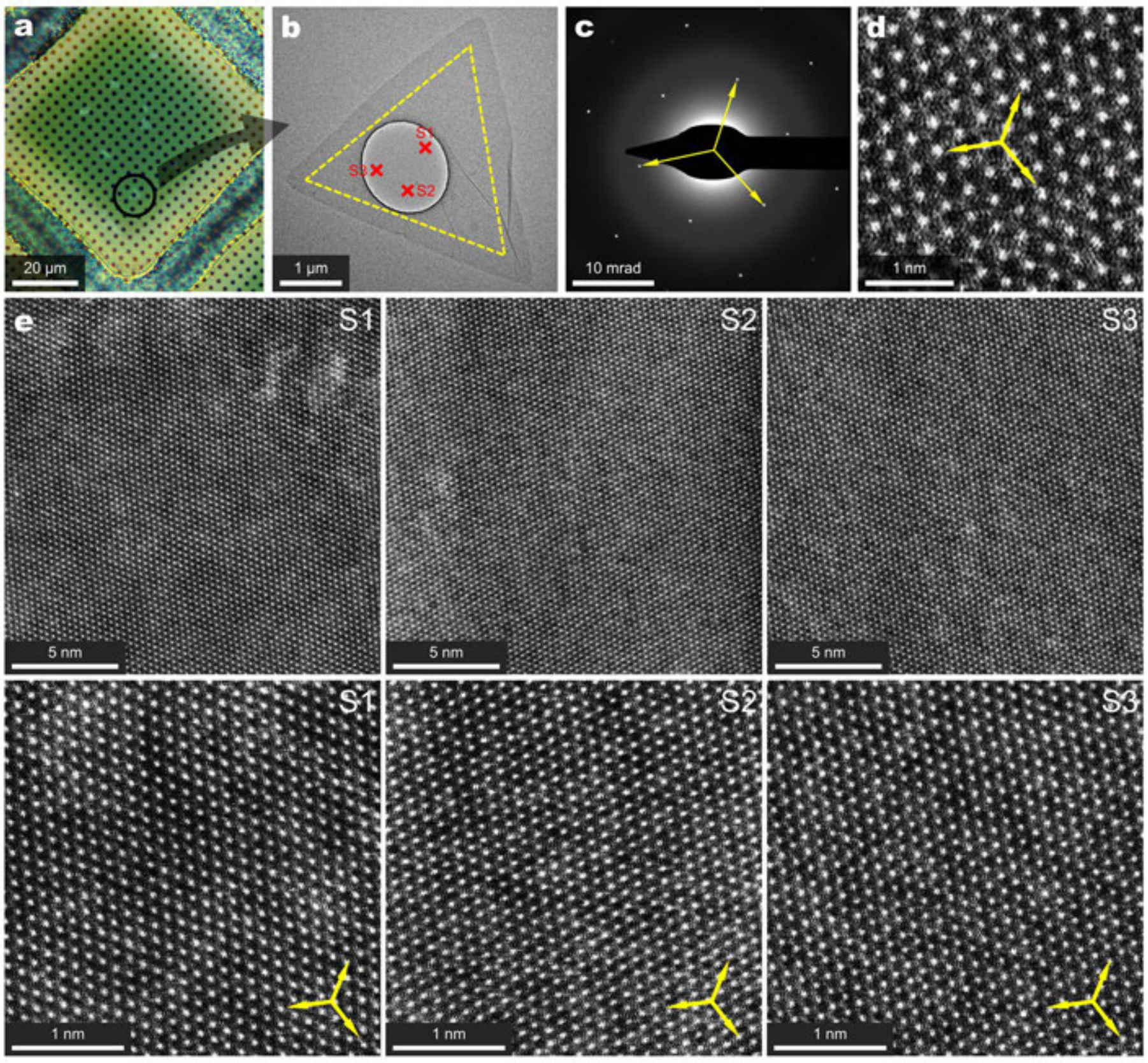

Fig. R19 | Structural TEM characterization of single-crystal MoS_2 flake by EF-SCN. a, OM image of EF-SCN single-crystal MoS_2 flakes transferred onto a quantifoil TEM grid. **b**, TEM image of single-crystal MoS_2 flake fully suspended over a hole. **c**,**d**, Diffraction pattern (**c**) and corresponding HAADF-STEM image (**d**) obtained from the single-crystal in **b**. **e**, HAADF-STEM images acquired at the S1, S2, and S3 positions marked in **b**, showing consistent atomic lattice orientation across the flake.

Fig. R19d shows an HAADF-STEM image corresponding to Fig. R19b, where the hexagonal lattice structure of monolayer 2H-phase MoS_2 is clearly observed, with its orientation consistent with the diffraction pattern in Fig. R19c. Fig. R19e presents HAADF-STEM images acquired at three positions (S1, S2, and S3) within the flake, confirming that the atomic lattice orientation is uniform across all regions, which directly supports the single-crystal nature of the flake. Furthermore, comparable surface quality is observed at the flake center (S1), edge (S2), and corner (S3), indicating that spatial variations in flake quality due to oxygen from the etching-flux barrier can be ruled out.

Based on these TEM analyses, it can be conclusively determined that the MoS_2 flake grown by the EF-SCN method is single-crystalline. In addition, the triangular morphology is not a result of anisotropic etching but rather originates from epitaxial growth aligned with the barrier pattern orientation, confirming the formation of well-defined triangular single-crystal MoS_2 flakes.

(ii) Atomic resolution AFM

Fig. R20 presents atomic resolution AFM analysis of a MoS_2 flake grown by EF-SCN. Unlike TEM measurements, which require transfer onto a grid, AFM measurements were performed directly after growth without additional processing. Fig. R20a shows an SEM image of single-crystal MoS_2 flakes grown within a 3 μm pattern, and Fig. R20b provides a low-magnification AFM image of the black dotted region indicated in Fig. R20a. This image clearly delineates the HfO_2 barrier and the MoS_2 region formed at the center of the growth area.

Fig. R20c shows a representative atomic resolution AFM image acquired from the MoS_2 region in Fig. R20b. A well-defined periodic hexagonal lattice, characteristic of MoS_2, is clearly observed across the entire scanned area. Considering previously reported atomic resolution AFM and conductive AFM results for MoS_2, the uniform contrast and continuous lattice observed in Fig. R20c indicate high crystallinity and a low defect density[41].

Fig. R20d presents atomic resolution AFM images obtained from three sites within Fig. R20b. The insets show the corresponding FFT patterns, where clear hexagonal symmetry consistent with MoS_2 is observed. The angles extracted from the FFT patterns at the three locations fall within a range of 22-24°, which is within the typical error margin arising from tip drift during AFM measurements. Therefore, these results provide strong evidence that the flake in Fig. R20b is single-crystal across its entire area.

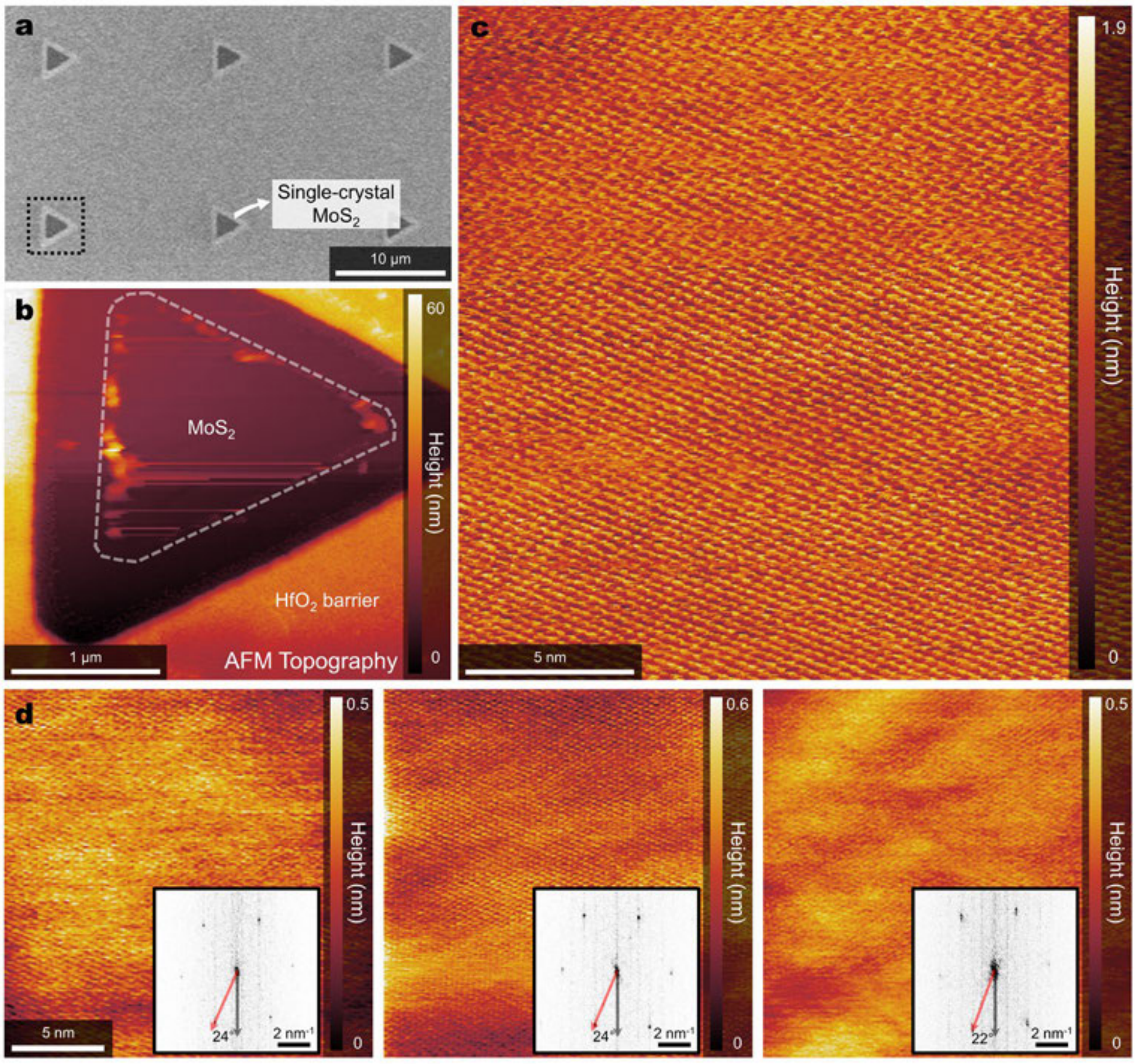

Fig. R20 | Structural AFM characterization of single-crystal MoS_2 flake by EF-SCN. a,**b**, SEM (**a**) and AFM (**b**) images of EF-SCN single-crystal MoS_2 flake. **c**,**d**, Atomic scale high resolution AFM images of single-crystal MoS_2 flake in **b** with FFT image.

(iii) SHG

Fig. R21 presents a rigorous analysis of MoS_2 flakes grown by the EF-SCN method using second-harmonic generation (SHG). Under a low DES growth condition (3 sccm) that promotes single nucleation, the SEM image (Fig. R21a) clearly shows the formation of a single-crystal MoS_2 flake within each pattern. The corresponding SHG mapping image (Fig. R21b) exhibits uniform SHG intensity across the entire flake area, indicating the absence of grain boundaries and thus confirming its single-crystal nature. Furthermore, as shown in Fig. R21c, angle-resolved SHG measurements reveal a well-defined six-petal pattern with clear six-fold symmetry, providing critical evidence that the MoS_2 flakes in Fig. R21a possess single-crystal SHG characteristics.

In contrast, under a high DES growth condition (15 sccm) that induces multiple nucleation, SEM images (Fig. R21d) show multiple MoS_2 flakes within each pattern. The corresponding SHG mapping image (Fig. R21e) displays regions of significantly reduced SHG intensity, indicative of grain boundaries and thus confirming the polycrystalline nature of the MoS_2. In addition, the angle-resolved SHG measurement in Fig. R21f shows an isotropic SHG intensity distribution, lacking the distinct six-petal pattern. This behavior can be attributed to the averaging of SHG signals from multiple domains with different orientations, leading to the loss of directional anisotropy. These results clearly demonstrate that the growth region in Fig. R21d consists of polycrystalline MoS_2.

Furthermore, angle-resolved SHG measurements were performed at the array level, as shown in Fig. R21g and R21h. All six randomly measured flakes exhibit clear six-petal SHG patterns, confirming their single-crystal nature. The SHG patterns of these flakes show similar crystallographic orientations within a range of 57-63°. The slight variation in orientation is attributed to the relatively low epitaxial growth temperature of 750 °C, which may limit the degree of alignment compared to higher temperature epitaxial growth. Nevertheless, the SHG analysis confirms that MoS_2 flakes grown via EF-SCN are unequivocally single-crystalline and exhibit consistent crystallographic orientation.

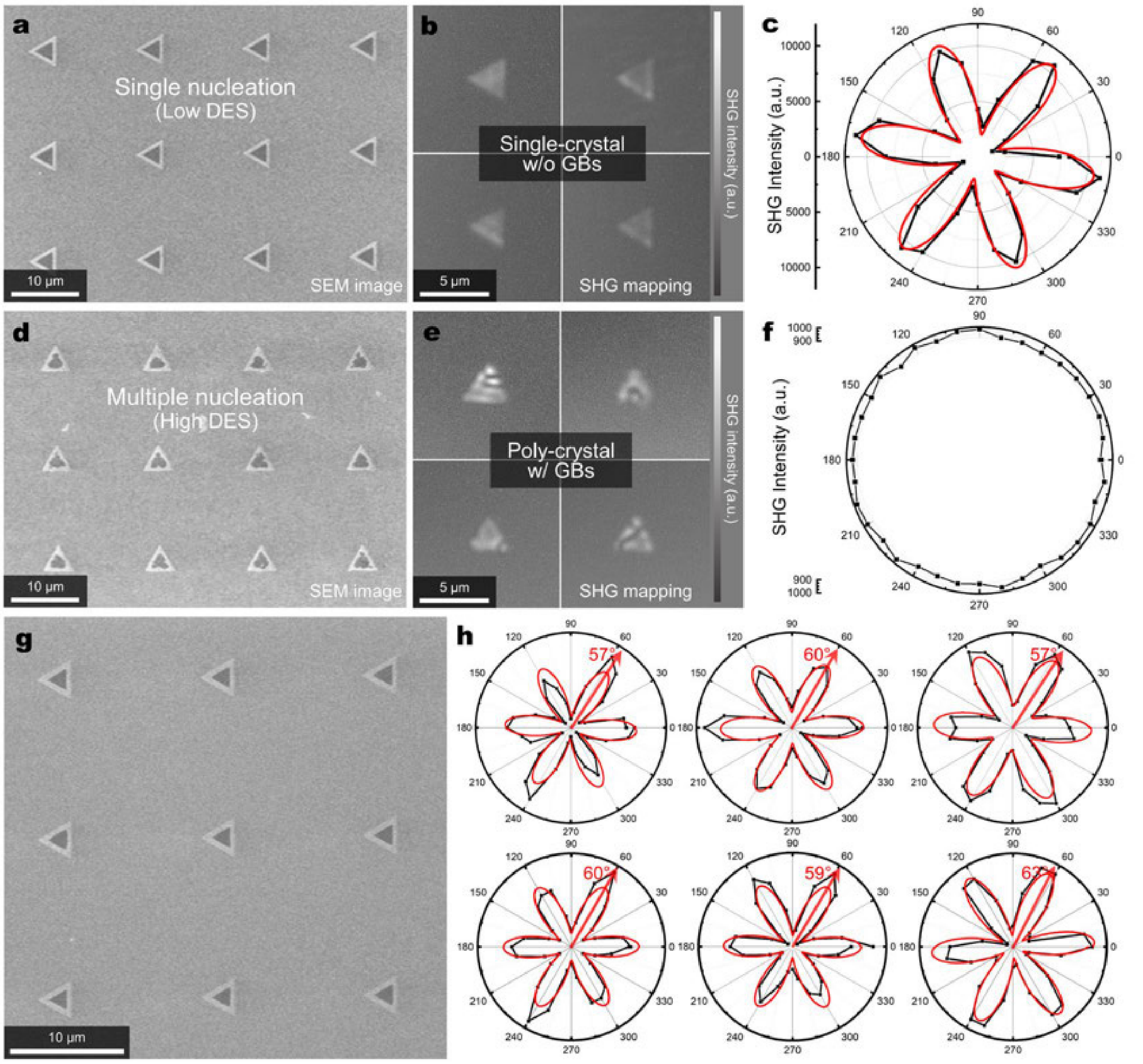

Fig. R21 | SHG mapping and angle-resolved SHG patterns of single-crystal MoS_2 flake by EF-SCN. a-c, SEM image (**a**), SHG mapping image (**b**), and angle-resolved SHG patterns (**c**) of single-crystal MoS_2 flakes with low DES condition. **d-f**, SEM image (**d**), SHG mapping image (**e**), and angle-resolved SHG patterns (**f**) of poly-crystal MoS_2 flakes with high DES condition. **g**,**h**, SEM image (**g**) and corresponding angle-resolved SHG patterns (**h**) of single-crystal MoS_2.

(iv) Faceting of flake edges

If the growth time is prolonged such that oxygen release from HfO_2 is sufficiently reduced, the etching effect acting on the MoS_2 flake edges would be substantially weakened. In this case, as noted by the reviewer, the flake shape may follow the pattern geometry due to anisotropic etching (Extended Data Fig. 1e). However, in most EF-SCN results, including those shown in Fig. R19-21, it is clearly observed that the flake morphology is consistent with the intrinsic crystallographic orientation of MoS_2. In addition, Fig. R10 examines the growth behavior when the MoS_2 epitaxial growth direction is not aligned with the barrier pattern orientation.

As shown in Fig. R10a and R10b, when the epitaxial growth direction and the barrier pattern direction are aligned, triangular MoS_2 flakes with clean edges are formed. This is a characteristic feature consistently observed in the single-nucleation regime of EF-SCN. In contrast, when the epitaxial direction and the barrier pattern are misaligned by 15° or 60°, the crystallographic orientation of the TMD follows epitaxial growth, while the etching process is influenced by the barrier pattern direction (Fig. R10c-f). Since the two directions are not aligned, the flake edges are no longer clean; instead, they consist of rough edges composed of facets corresponding to the MoS_2 crystallographic directions. Therefore, the triangular flakes with clean edges observed in typical EF-SCN results can be interpreted as well-aligned single-crystal MoS_2. Even in cases where rough edges are formed due to misalignment, the fact that the facet orientations remain consistent within a entire flake strongly suggests that these flakes are also single-crystalline.

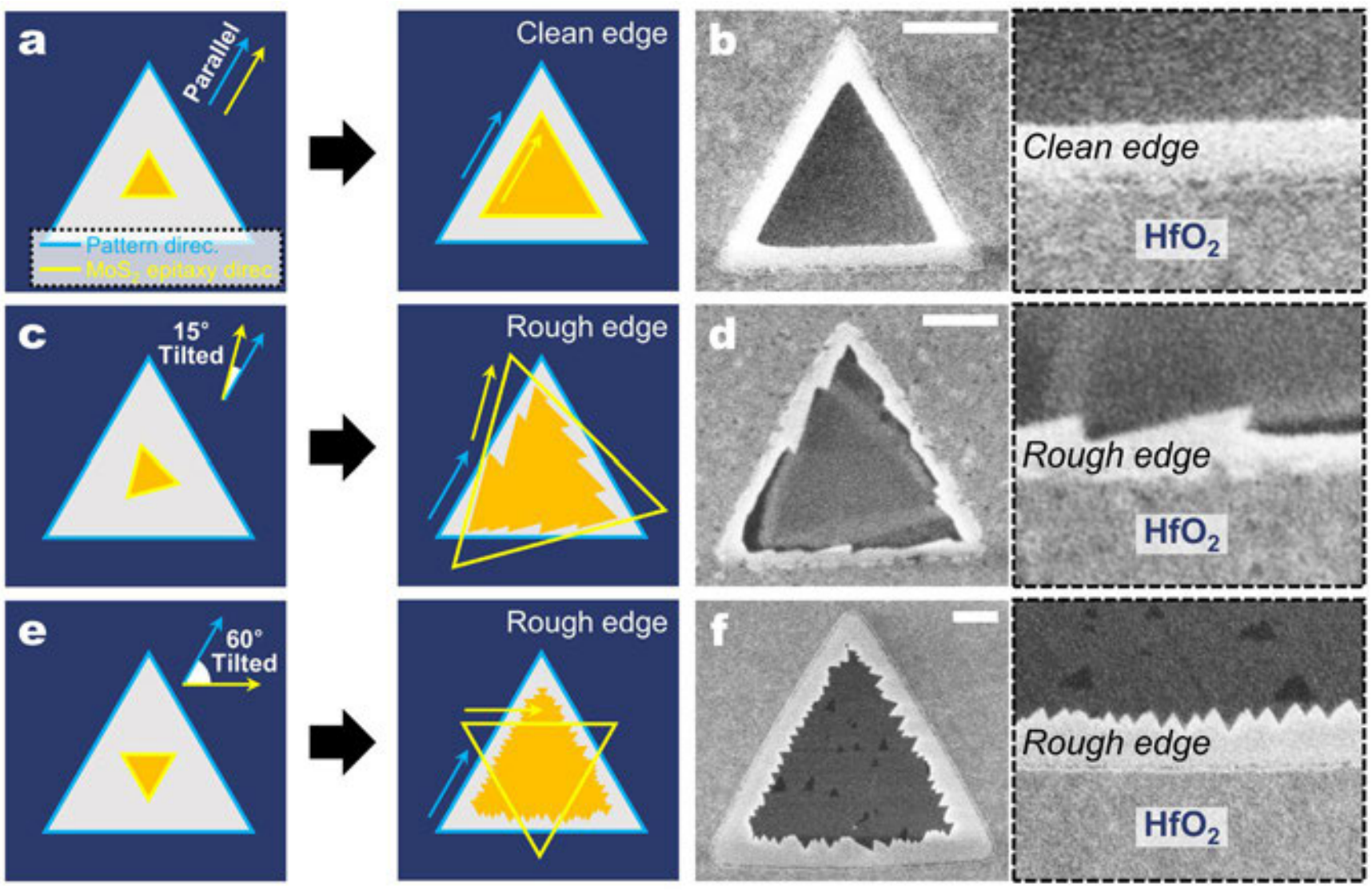

Fig. R10 | Discussion on the relationship between MoS_2 epitaxial growth direction and HfO_2 barrier pattern direction. a,**b**, Schematic (**a**) and SEM images (**b**) of MoS_2 growth when the epitaxial growth direction and the barrier pattern direction are parallel. **c**-**f**, Schematic and SEM images of MoS_2 growth at misalignment angles of 15° (**c**, **d**) and 60° (**e**, **f**) between the epitaxial growth direction and the barrier pattern direction. Scale bars, 1 μm.

(v) Single-crystal growth on an amorphous substrate

We acknowledge the reviewer's concern that, in epitaxial growth systems, multiple nucleation events at different sites could still yield an apparently single-crystalline film if all nuclei share the same crystallographic orientation dictated by the substrate. To address this, we performed EF-SCN on an amorphous SiO_2 substrate, where epitaxial alignment is absent, thereby providing an unambiguous test of whether single-centered nucleation — rather than orientation-averaging through epitaxy — is responsible for the observed single crystallinity.

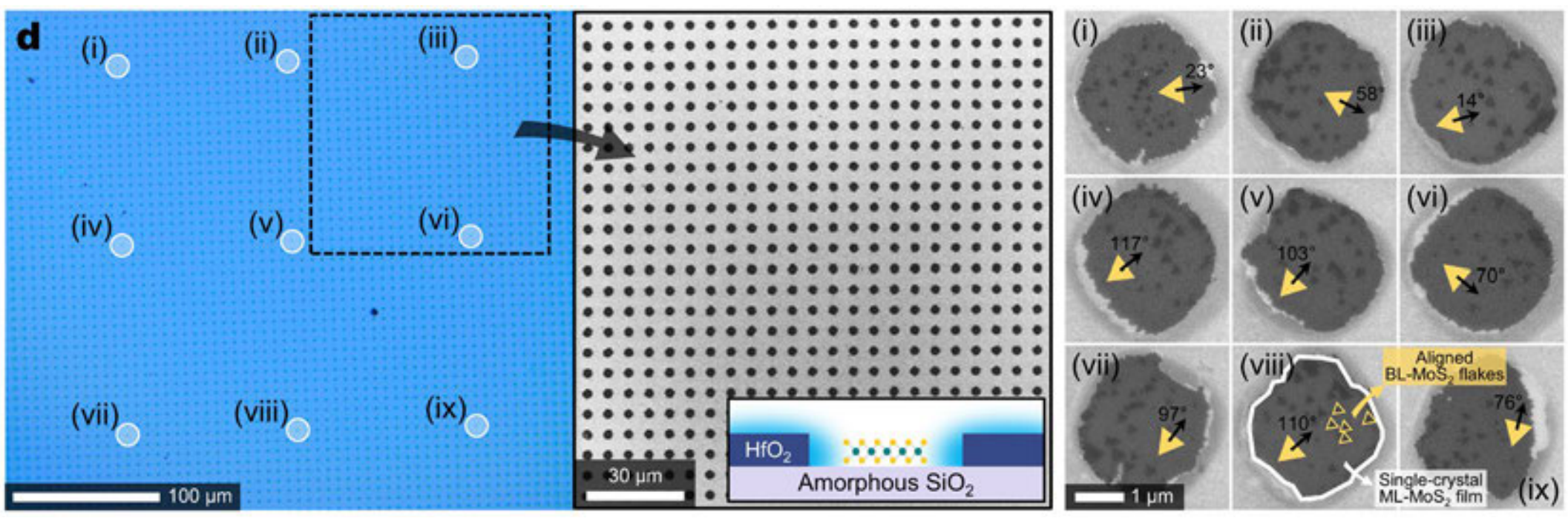

Fig. R17 | Large-scale single-crystal growth of EF-SCN. d, OM image and SEM images showing the uniformity of single-crystal MoS_2 EF-SCN growth on an amorphous SiO_2 substrate.

As shown in Fig. R17d, EF-SCN growth on an amorphous SiO_2 substrate using an HfO_2 barrier achieved uniform single-crystal growth across more than 2500 sites over a large area exceeding 400 μm. Enlarged SEM images of 9 randomly selected sites confirm the formation of a monolayer MoS_2 film with bilayer MoS_2 flakes. Critically, since the substrate is amorphous, the crystallographic orientation of the monolayer film varies from site to site — ruling out any substrate-mediated orientation alignment. Nevertheless, within each individual site, the bilayer flakes exhibit a consistent crystallographic orientation, demonstrating that the underlying monolayer MoS_2 is a single-crystal. This result provides direct evidence that the single-crystallinity observed in EF-SCN originates from single-centered nucleation driven by the oxygen etching-flux, rather than from epitaxial coalescence of multi-nucleated domains.

[Action in Manuscript]

i) We have added **Extended Data Fig. 2,3 (p. 24, 25)** and **Supplementary Fig. 3,7 (SI p. 4,8)** to verify the single-crystallinity of MoS_2 flake grown by EF-SCN method.

(p. 24) Extended Data Fig. 2 | TEM and atomic resolution AFM measurements of single-crystal MoS_2 flake by EF-SCN. a, TEM image of single-crystal MoS_2 flake fully suspended over a TEM grid hole. b, Diffraction pattern image obtained from the single-crystal in a. c, HAADF-STEM images acquired at S1, S2, and S3 positions marked in a, showing consistent atomic lattice orientation across the flake. d, Representative atomic resolution AFM image of single-crystal MoS_2 flake by EF-SCN. e, AFM and corresponding FFT pattern images of single-crystal MoS_2.

(p. 25) Extended Data Fig. 3 | SHG mapping and angle-resolved SHG pattern of single-crystal MoS_2 flake by EF-SCN. a-c, SEM image (a), SHG mapping image (b), and angle-resolved SHG patterns (c) of single-crystal MoS_2 flakes with low DES condition. d-f, SEM image (d), SHG mapping image (e), and angle-resolved SHG patterns (f) of poly-crystal MoS_2 flakes with high DES condition. g,h, SEM image (g) and corresponding angle-resolved SHG patterns (h) of single-crystal MoS_2.

(SI p. 4) Supplementary Fig. 3 | Relationship between MoS_2 epitaxial growth direction and HfO_2 barrier pattern direction. a,b, Schematic (a) and SEM images (b) of MoS_2 growth when the epitaxial growth direction and the barrier pattern direction are parallel. c-f, Schematic and SEM images of MoS_2 growth at misalignment angles of 15° (c, d) and 60° (e, f) between the epitaxial growth direction and the barrier pattern direction. Scale bars, 1 μm.

(SI p. 8) Supplementary Fig. 7 | 2D MoS_2 EF-SCN on amorphous substrate. a, SEM images of 2D MoS_2 EF-SCN on amorphous SiO_2 substrate, corresponding to the enlarged views of Fig. 2d (i-ix). Small bilayer MoS_2 flakes (dark contrast) are observed on the monolayer MoS_2 surface, exhibiting a consistent crystallographic orientation within each pattern site.

ii) We have modified **manuscript (p. 4-5, 7)** to provide comprehensive structural characterizations confirming the single-crystallinity of MoS_2 flake grown by EF-SCN method.

(p. 4-5) "The diffraction pattern obtained from a MoS_2 flake suspended over a TEM grid hole (Extended Data Fig. 2a) exhibits a typical hexagonal spot pattern of single-crystal MoS_2, rather than multiple spots or ring patterns (Extended Data Fig. 2b). Furthermore, as shown in Extended Data Fig. 2c, high-angle annular dark-field scanning transmission electron microscopy (HAADF-STEM) images acquired at the center (S1), edge (S2), and corner (S3) positions of the flake reveal consistent crystallographic orientation across all regions, aligned with the diffraction pattern. These results confirm that the entire flake region has a consistent crystallographic orientation, demonstrating its single-crystal nature. In addition, Extended Data Fig. 2d, e present atomic resolution AFM measurements, where a well-defined periodic hexagonal lattice characteristic of MoS_2 is clearly observed across the entire scanned area. AFM and corresponding fast Fourier transform (FFT) analyses performed at three arbitrary locations within a flake confirm identical crystallographic orientation.", "Extended Data Fig. 3 shows second-harmonic generation (SHG) analyses of single-crystal MoS_2 grown by EF-SCN. In the case of single nucleation, a single triangular flake is observed in SEM image, and the absence of grain boundaries results in uniform SHG intensity mapping, along with a well-defined six-petal pattern in angle-resolved SHG, characteristic of single-crystal MoS_2 (Extended Data Fig. 3a-c). In contrast, under multiple nucleation conditions, multiple domains and irregular flake morphologies are observed in SEM image, accompanies by significant intensity drop at grain boundaries in SHG mapping. The coexistence of domains with different orientations results in an isotropic SHG intensity distribution rather than a six-petal pattern (Extended Data Fig. 3d-f). The barrier patterns in EF-SCN are aligned with the epitaxial growth direction of MoS_2 on miscut sapphire. Accordingly, SHG measurements at six arbitrary positions within the single-crystal MoS_2 array consistently exhibit six-petal patterns with similar crystallographic orientations (Extended Data Fig. 3g, h).", "Furthermore, when the barrier pattern direction is misaligned with the epitaxial growth direction, rough edges aligned with the intrinsic crystallographic orientation are observed near the barrier, instead of the clean triangular flake typical of EF-SCN (Supplementary Fig. 3). These results indicate that the orientation of triangular single-crystal MoS_2 in EF-SCN is determined by the epitaxial growth direction rather than the barrier pattern geometry."

(p. 7) "Fig. 2d shows optical microscope (OM) and SEM images of uniform single-crystal growth on an amorphous SiO_2 substrate over a 400 μm area. As shown in the SEM images in Supplementary Fig. 7, bilayer MoS_2 flakes (dark contrast) grown at nine random pattern sites in Fig. 2d exhibit consistent orientation, indicating that the underlying monolayer MoS_2 is single-crystalline. However, due to the amorphous substrate, the crystal orientations across different sites are random."

(2) For practical application, the process window is very important. It shows in Fig. 3b and Fig. 3c that only when L_{growth} = 3 μm and DES is 1 sccm, the claimed single nucleation can be effectively achieved. I would suggest to briefly describe whether it is possible to further enlarge the process window for larger tolerance. In addition, it is shown in Fig. 3f that the critical length becomes almost 0.6 μm when L_{growth} = 3 μm, which is different from what the authors stated "as L_{growth} increases, the critical length remains nearly constant."

[Author reply]

This comment raises an important question regarding the process window of the EF-SCN approach for practical applications. Accordingly, we conducted additional experiments to evaluate the scalability of the process window not only with respect to the DES flow raised by the reviewers, but also in terms of growth temperature and the feasibility of large-area synthesis.

(i) DES flow window for valid single-crystal growth

We first conducted a more detailed analysis of the range over which reliable single-crystal growth can be achieved at a fixed pattern size. Fig. R16 presents SEM images showing the MoS_2 nucleation behavior at a pattern size of 3 μm under varying DES flow, along with a quantitative evaluation of the number of flakes within each pattern. As described in Main Fig. 3a, increasing the DES flow expands the nucleation-active region (NAR), resulting in a systematic transition in nucleation behavior from zero to single and eventually to multiple nucleation. This trend is consistently observed in the additional analysis in Fig. R16. For the 3 μm pattern, zero nucleation occurs at a DES flow of 2 sccm, while a single flake is consistently formed within each pattern in the 3-7 sccm range. In contrast, when the DES flow exceeds 10 sccm, multiple nucleation is observed. These observations indicate that the DES flow range for single-crystal growth is 3-7 sccm, corresponding to an effective process window of approximately ±40% relative to the central value.

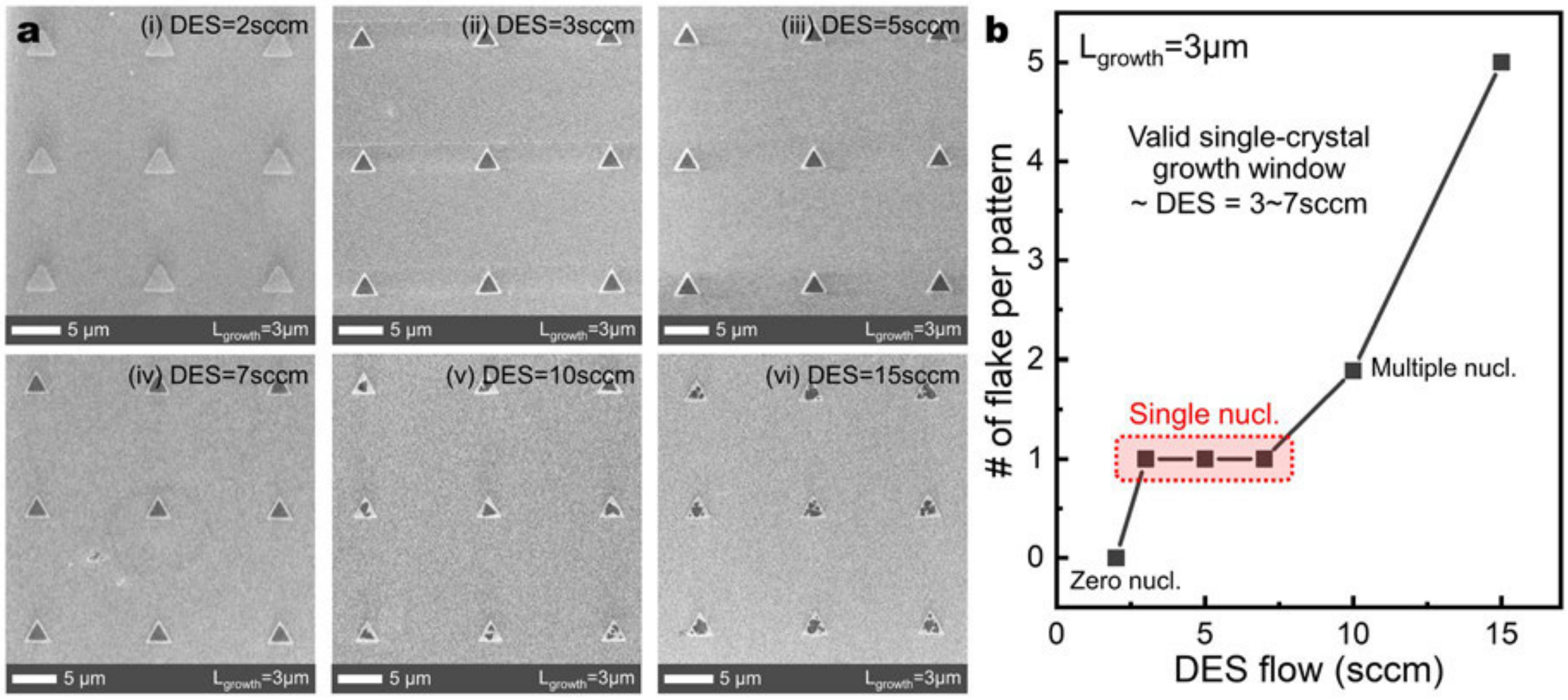

Fig. R16 | Valid process window for single-crystal MoS_2 growth. a, SEM images showing MoS_2 EF-SCN behavior with DES flow (2-15 sccm). **b**, Plot of the number of MoS_2 flakes per pattern as a function of DES flow.

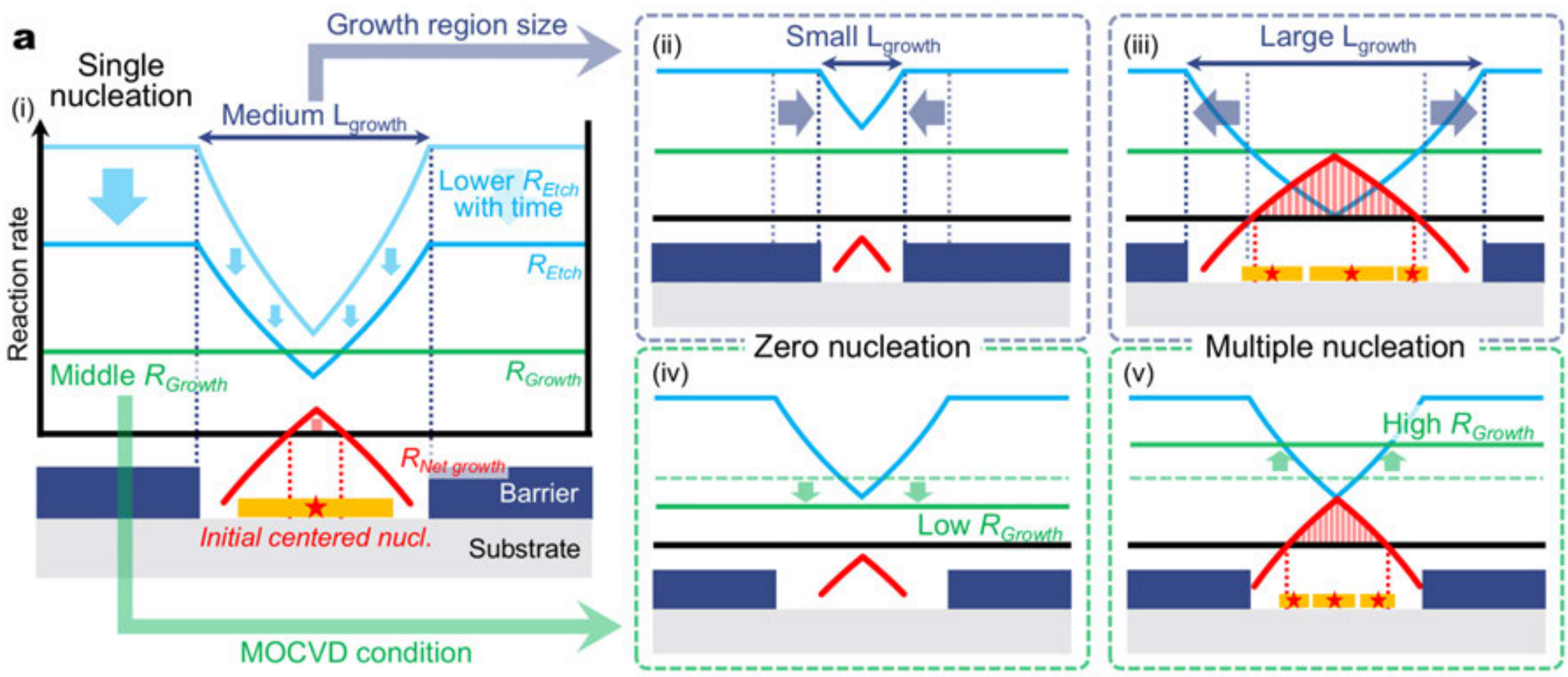

Main Fig. 3a

Turning to the second point noted by the reviewers, Extended Data Fig. 7d (Fig. 3f in the previous manuscript) indicates a critical length of ~0.6 μm at a growth region size of 3 μm, which differs from the values obtained for other pattern sizes. However, we conclude that the critical length is nearly independent of the growth region size, as this deviation arises from the statistical methodology used in data processing. Extended Data Fig. 7a serves as the basis for the data shown in Extended Data Fig. 7d. It presents a contour map constructed by defining concentric triangular unit areas for each pattern size, and counting the number of MoS_2 flakes with centers located within each region. For larger growth region sizes (5-9 μm), nucleation is absent only in the outermost unit region, leading to a consistent critical length of ~0.3 μm. In contrast, for the 3 μm pattern, nucleation is not observed in the outermost and second outermost regions, resulting in an overestimated critical length of ~0.6 μm. This deviation originates from the discrete nature of the nucleation counting method. However, the key point of Extended Data Fig. 7d is that the critical length is governed by the distance from the oxygen-releasing barrier, as shown in Main Fig. 3a(i)-(iii), and thus should remain invariant with respect to pattern size. Therefore, the consistent critical lengths obtained for larger pattern sizes (5, 7, and 9 μm) are sufficient to support this interpretation.

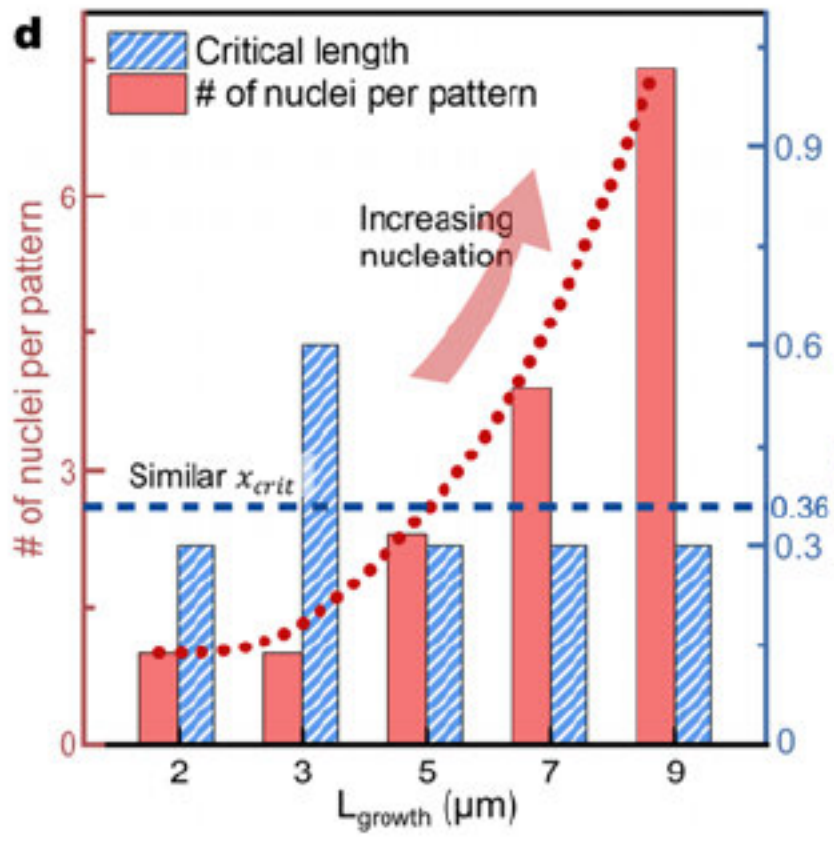

Extended Data Fig. 7d

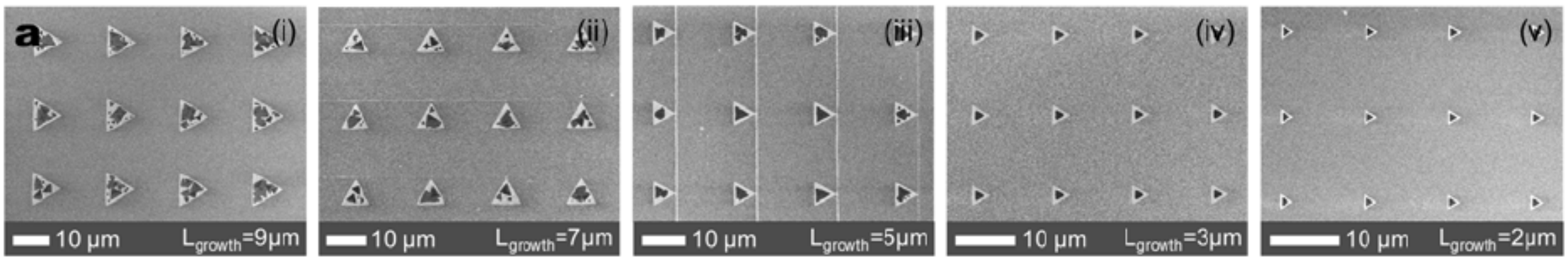

Extended Data Fig. 7a

(ii) Growth temperature window for valid single-crystal growth

In addition to the DES flow window raised by the reviewers, we further evaluated the scalability of the process window with respect to growth temperature. The key principle of EF-SCN lies in utilizing the intrinsic etching-flux released from the barrier to deterministically localize the nucleation site at the center of the growth region, thereby enabling controlled single-crystal growth of 2D TMD. In our previous manuscript, a high growth temperature (750 °C) was required to induce sufficient oxygen release from the HfO_2 barrier. Therefore, we hypothesized that EF-SCN could be extended to lower temperatures by employing alternative barrier materials capable of releasing oxygen at reduced temperatures.

[redacted]

[redacted]

[redacted]

[redacted]

(iii) Large-scale growth with valid single-crystal growth

To evaluate the scalability of EF-SCN across large areas, we fabricated 25 patterns at 16 regions on a 2 cm miscut sapphire substrate, resulting in a total of 400 sites. The resulting MoS_2 growth behavior on the substrate is shown in Fig. R17a-c. SEM analysis (Fig. R17b) shows that single-centered nucleation is observed at 397 out of 400 sites. This corresponds to a single-crystal yield of 99.3%, which demonstrates the strong potential of EF-SCN for large-scale applications (Fig. R17c).

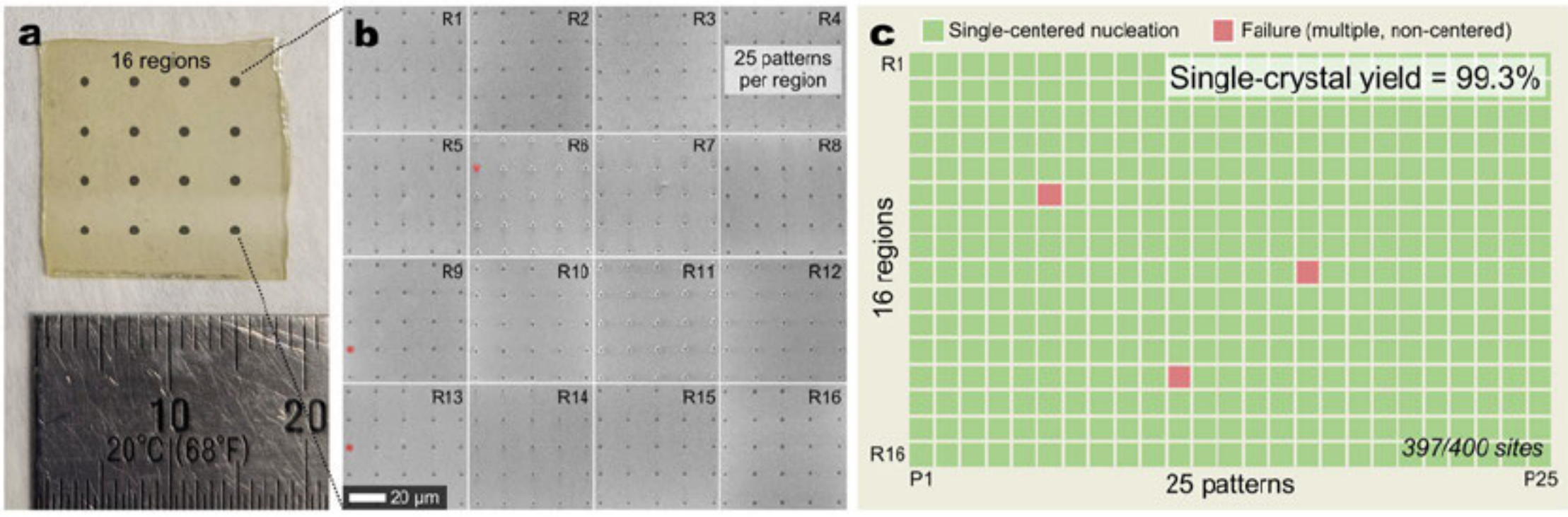

Fig. R17 | Large-scale single-crystal growth of EF-SCN. a-c, OM image (**a**), SEM images (**b**), and single-crystal mapping image (**c**) of large-scale MoS_2 EF-SCN consisting of 16 regions, each containing 25 patterns (a total of 400 sites).

[Action in Manuscript]

i) We have revised **Fig. 2,3 (p. 20,21)** and added **Extended Data Fig. 5 (p. 27)** and **Supplementary Fig. 9 (SI p. 10)** to demonstrate the process compatibility of EF-SCN, including low-temperature EF-SCN, large-scale uniformity, and valid single-crystal growth window.

(p. 20) Fig. 2 | Etching-flux barrier mechanism and process compatibility of EF-SCN system. e-g, OM image (e), SEM images (f), and single-crystal mapping (g) of large-scale MoS_2 EF-SCN consisting of 16 regions, each containing 25 patterns (a total of 400 sites).

(p. 21) Fig. 3 | Nucleation design rule of 2D MoS_2 EF-SCN. e, The average number of MoS_2 flakes per pattern as function of DES flow at L_{growth}=3 μm.

(p. 27) Extended Data Fig. 5 | Low-temperature EF-SCN and high-density single-crystal growth. c, SEM images of MoS_2 EF-SCN behaviors in (i) low, (ii) middle, and (iii) high pattern density substrates. Scale bars, 5 μm.
(SI p. 10) Supplementary Fig. 9 | Valid DES flow window for single-crystal MoS_2 growth. a, SEM images showing MoS_2 EF-SCN behavior with DES flow (2-15 sccm).

ii) We have updated **manuscript (p. 7)** to describe the low-temperature EF-SCN by using ZrO_2 barrier.
(p. 7) "Therefore, we employed ZrO_2, which is known to have a lower crystallization temperature than HfO_2, as an alternative etching-flux barrier[33,34]. As shown in the SEM images in Extended Data Fig. 5b, the use of a ZrO_2 etching-flux barrier results in effective single-centered nucleation, rather than edge nucleation, even at a low MoS_2 MOCVD temperature of 500 °C. ~"

iii) We have revised **manuscript (p. 8)** to demonstrate large-scale uniformity of single-crystal growth by EF-SCN.
(p. 8) "Importantly, Fig. 2e-g demonstrate the single-crystal yield and uniformity at a large-scale. A total of 400 sites were fabricated across 16 regions on a 2 cm substrate (25 patterns per region), achieving a single-crystal yield exceeding 99%. These results indicate that EF-SCN provides strong compatibility with semiconductor processing and scalability toward integration."

iv) We have improved **manuscript (p. 9)** to define the DES flow window for valid single-crystal MoS_2 growth.
(p. 9) "Fig. 3e shows the nucleation behavior as a function of DES flow (2-15 sccm) for L_{growth} = 3 μm. As observed in the SEM images in Supplementary Fig. 9, zero nucleation occurs at 2 sccm, while multiple nucleation is observed at 10-15 sccm. A DES flow range of 3-7 sccm is identified as an effective window for single-crystal MoS_2 growth."

(3) In the nucleation probability profiles shown in Fig. 3. Error bar should be indicated and well-defined in the statistical results.

[Author reply]

Main Fig. 3d presents the probabilities of single and centered nucleation as a function of growth region size, derived from the data shown in Extended Data Fig. 7a-c. The single nucleation probability is defined as the fraction of patterns (out of 12) containing exactly one flake, whereas the centered nucleation probability corresponds to the fraction of flakes located within the central unit region, as illustrated in Extended Data Fig. 7b. In essence, the original dataset is based on a simplified analysis in which the pattern area is partitioned into discrete regions to evaluate nucleation probability and critical size. Consequently, incorporating statistical analysis with error bars into the existing dataset is not straightforward.

To overcome this limitation, we performed an additional statistical analysis of nucleation positions using the data in Extended Data Fig. 7a. Fig. R24 shows the distribution of distances between the pattern center and the incenter of MoS_2 flakes across different growth region sizes. Unlike the previous segmented-area approach, we determined the positions of all individual flakes and directly quantified their distances from the pattern center. This enables a more rigorous comparison of nucleation position distributions as a function of growth region size and chalcogen conditions.

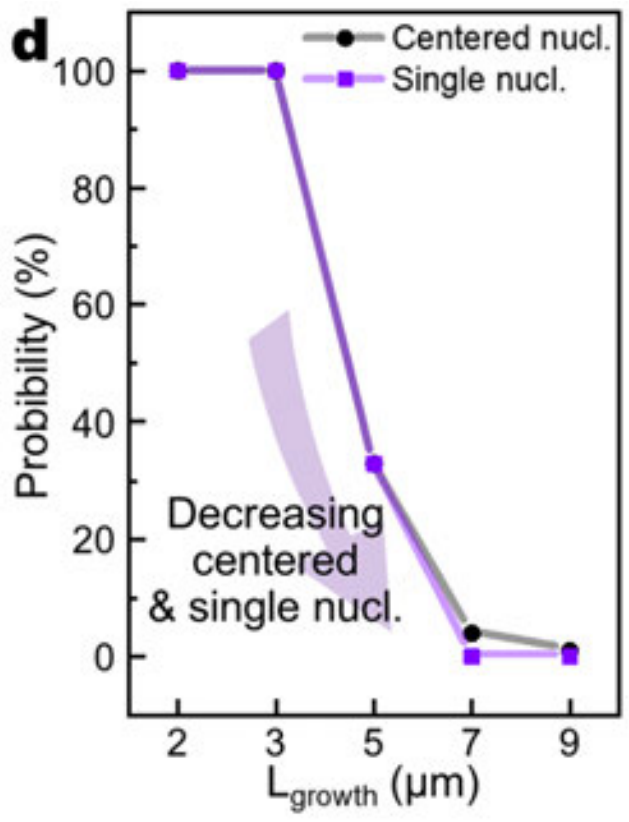

Main Fig. 3d

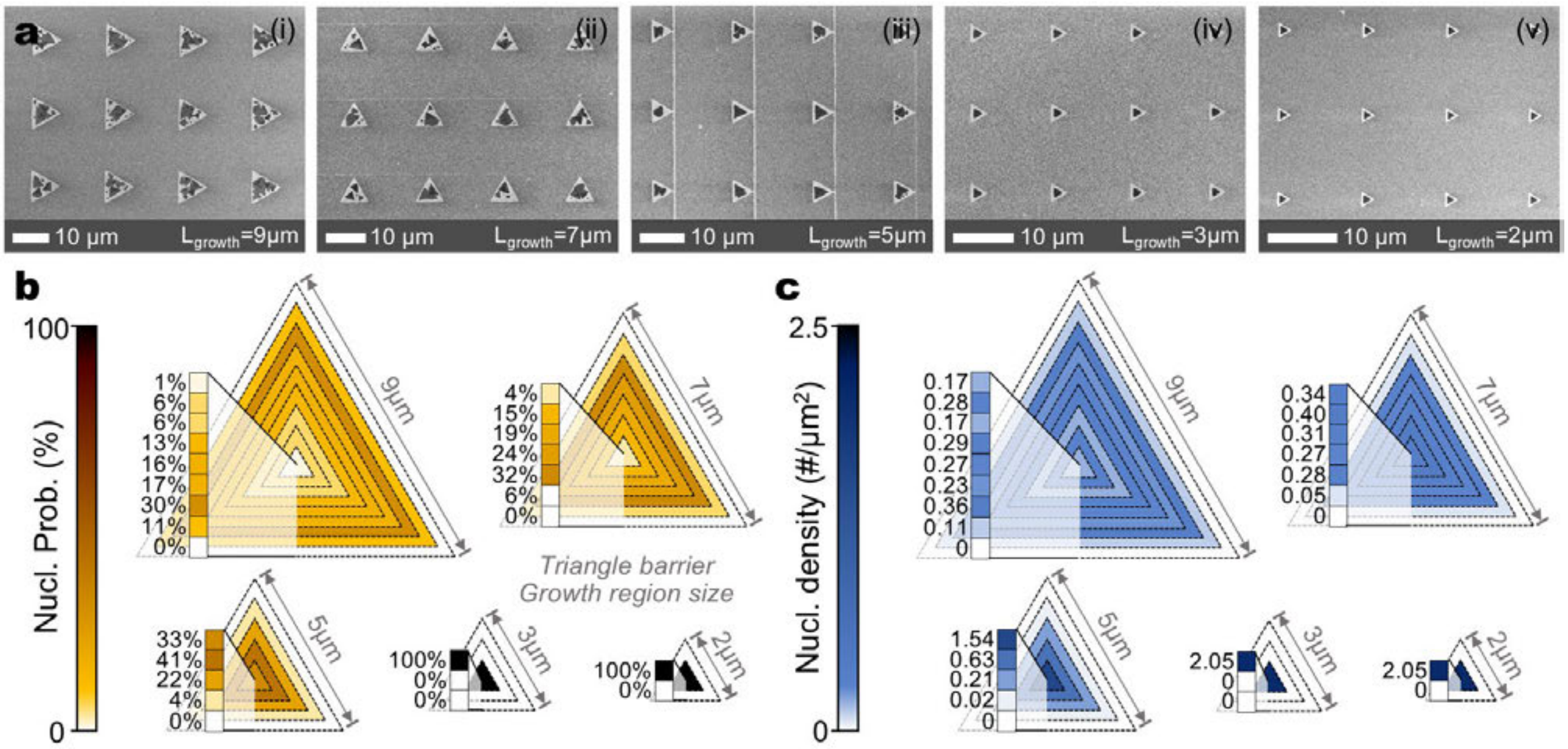

Extended Data Fig. 7a-c

The high chalcogen condition in Fig. R24a shows that, as the growth region size increases, both the mean distance from the pattern center and the spread of the distribution increase systematically. At a 3 μm pattern, the mean distance is ~0.05 μm, indicating strong localization of nucleation near the center with minimal dispersion. As the pattern size increases to 5 μm, the mean distance rises to ~0.6 μm, accompanied by an expansion of the interquartile range (IQR) to 0.3–0.9 μm. For larger growth region sizes of 7 μm and 9 μm, the mean distances further increase to ~1.4 μm and ~2.2 μm, with corresponding IQR ranges of 0.9–2.0 μm and 1.6–3.0 μm. These results indicate that nucleation progressively shifts away from the center while becoming more spatially heterogeneous with increasing growth region size.

Under low chalcogen conditions in Fig. R24b, both the mean distance and the spread of the distribution are smaller than those observed under high chalcogen conditions, suggesting that reduced chalcogen flow promotes more centralized nucleation. However, the total number of flakes is significantly lower under these conditions (e.g., 26 and 27 flakes at 7 μm and 9 μm, respectively, compared to 47 and 89 under high chalcogen conditions). As a result, statistical robustness is limited, which likely obscures any clear trend in dispersion with increasing growth region size.

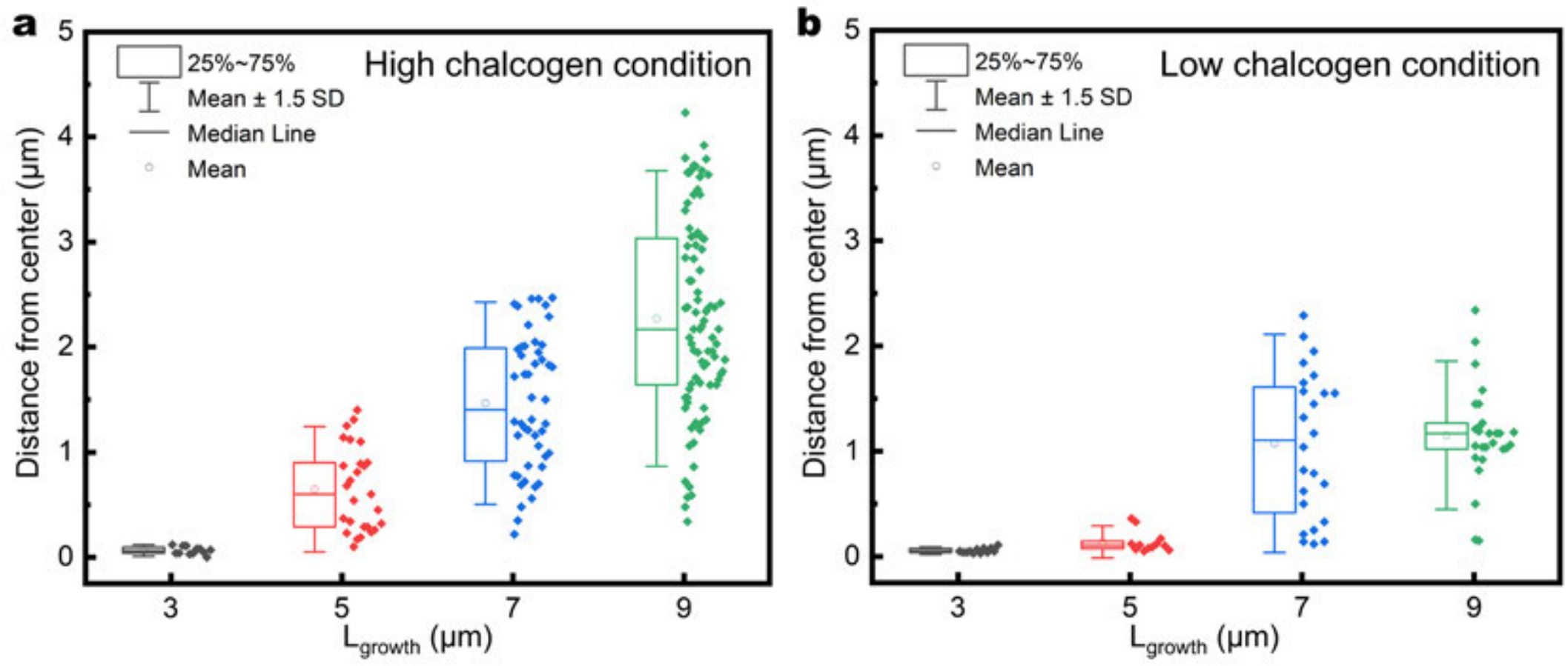

Fig. R24 | Statistical analysis of nucleation positions in the EF-SCN system. a,**b**, Box plots showing distribution of distances from pattern center to the incenter of MoS_2 flakes as a function of growth region size.

[Action in Manuscript]

i) We have modified **Extended Data Fig. 7 (p. 29)** to explain the distance distribution of MoS_2 flake from pattern center as a function of growth region size with error bars.

(p. 29) Extended Data Fig. 7 | 2D MoS_2 nucleation behavior with growth region size. e, Box plots showing distribution of distances from pattern center to the incenter of MoS_2 flakes as a function of growth region size.

ii) We have revised **manuscript (p. 9)** to describe the statistical analysis of MoS_2 nucleation behavior in EF-SCN system.

(p. 9) "Extended Data Fig. 7e shows the distribution of distances between the pattern center and the incenter of MoS_2 flakes across different growth region sizes. As the growth region size increases, both the mean distance from the pattern center and the spread of the distribution increase systematically. This result suggests that as L_{growth} increases, the NAR expands, leading to an increase in both the number and spatial distribution of nucleation events."

(5) Miscut sapphire substrates have been used for the epitaxial growth of 2D TMD including MoS_2. As mentioned in the above comment, the triangle orientation is randomly distributed in multiple nucleation scheme. It may be critical to understand whether the terrace of the substrate under specific miscut angle and EBL patterned angle are aligned.

[Author reply]

As noted by the reviewers, a random distribution of flake orientations is observed in the multiple nucleation regime. This behavior originates from the degradation of epitaxial alignment with increasing chalcogen supply, as illustrated in Fig. R25. Under low chalcogen conditions, the orientation remains well aligned due to epitaxial growth (Fig. R25a), whereas increasing the chalcogen supply leads to a loss of directional control (Fig. R25b). Within the EF-SCN system, low chalcogen conditions are favorable for single-crystal growth and concurrently support strong epitaxial alignment. In contrast, high chalcogen conditions promote multiple nucleation and are associated with reduced overall orientation alignment. Consequently, the triangular orientations of TMD flakes exhibit a random distribution under these conditions.

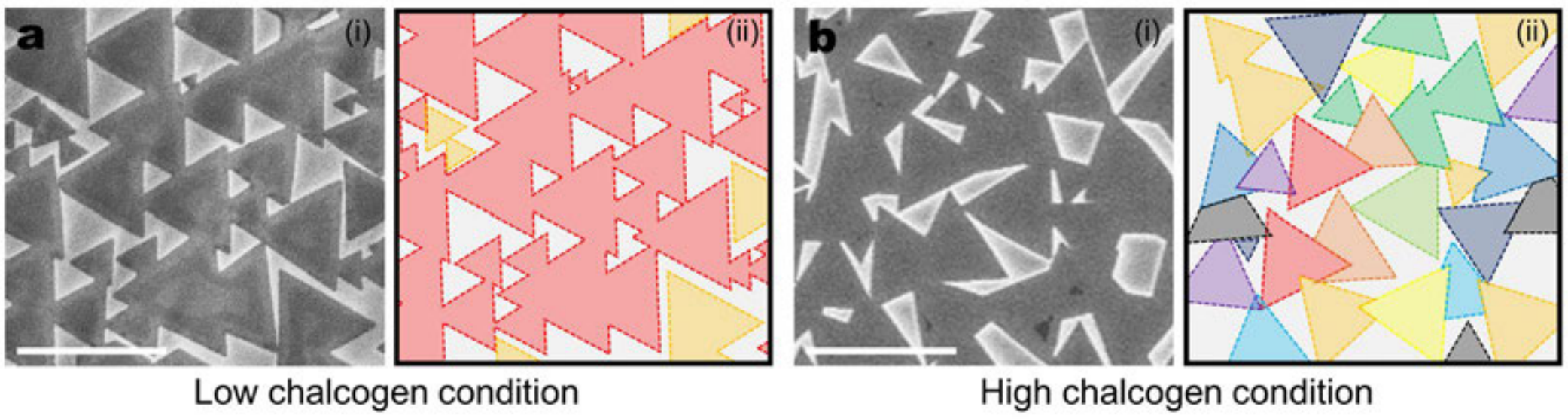

Fig. R25 | Epitaxial growth behavior on miscut sapphire with growth conditions. a,**b**, SEM images and schematic showing growth behavior of MoS_2-$MoSe_2$ heterostructure and MoS_2 flakes under low DES (**a**) and high DES (**b**) conditions. Scale bars, 5 µm.

Turning to pattern orientation, in the previous manuscript, the triangular barrier patterns were aligned parallel to the epitaxial growth direction of MoS_2, which precluded a systematic assessment of the effect of pattern orientation on single-crystal growth behavior. To address this, we conducted additional investigations to examine how the alignment between the epitaxial growth direction and the barrier pattern affects the crystallographic orientation of MoS_2.

Fig. R10 presents the growth behavior when the barrier pattern orientation deviates from the epitaxial growth direction of MoS_2. Fig. R10a and R10b show a schematic and an SEM image of the case in which the barrier pattern is aligned parallel to the epitaxial direction, exhibiting clean edges of single-crystal MoS_2 flakes. In contrast, Fig. R10c and R10d show a 15° misalignment, where the flakes are preferentially etched along a single direction, resulting in rough edges. Similarly, Fig. R10e and R10f (60° misalignment) exhibit flakes with rough edges consistent with the same etching tendency. These results indicate that the orientation of MoS_2 flakes is not dictated by the geometry of the HfO_2 barrier pattern but instead follows the intrinsic epitaxial growth direction. Moreover, the consistent etching-induced faceting direction across the flakes further supports their single-crystallinity.

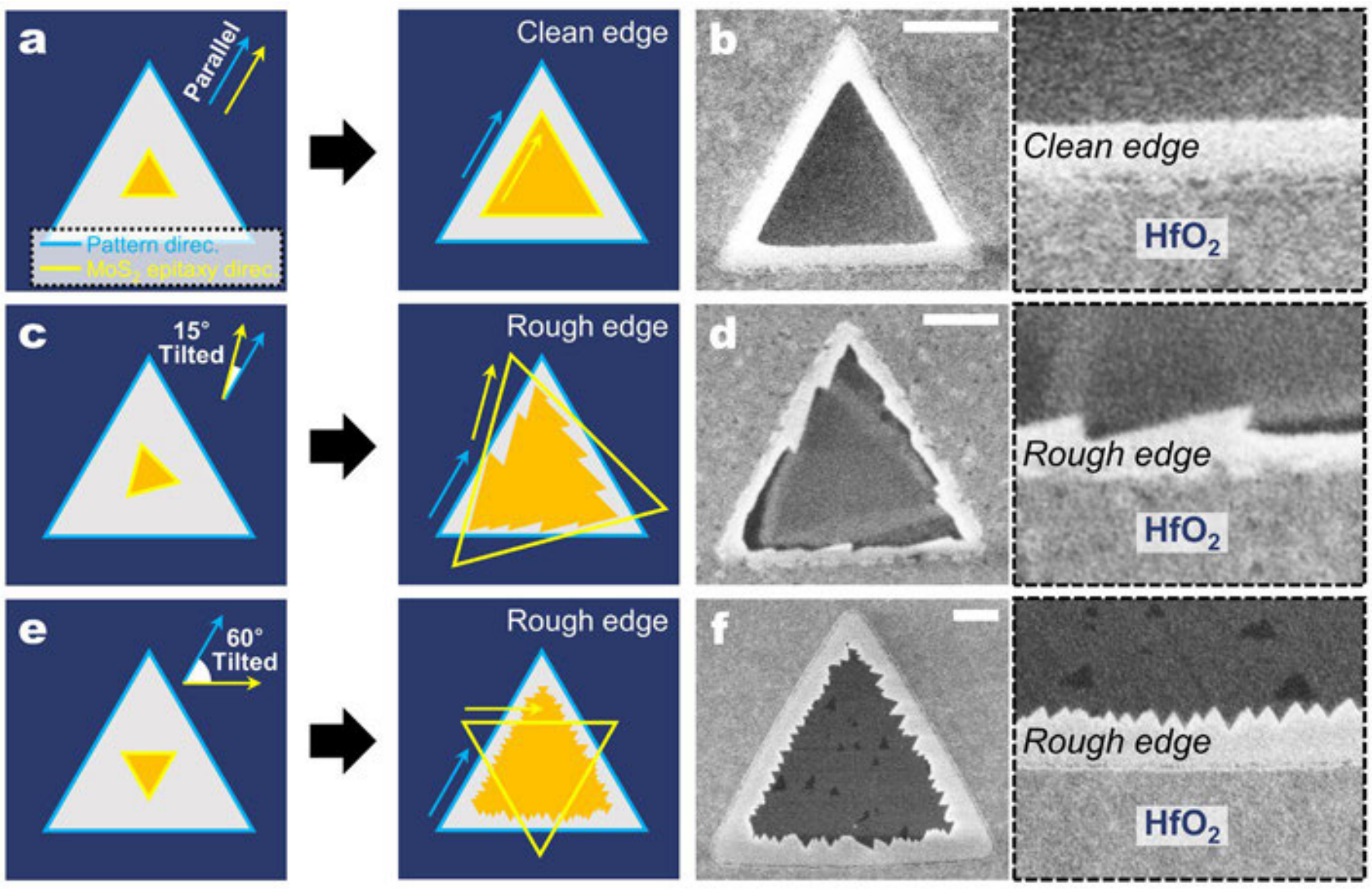

Fig. R10 | Discussion on the relationship between MoS_2 epitaxial growth direction and HfO_2 barrier pattern direction. a,**b**, Schematic (**a**) and SEM images (**b**) of MoS_2 growth when the epitaxial growth direction and the barrier pattern direction are parallel. **c**-**f**, Schematic and SEM images of MoS_2 growth at misalignment angles of 15° (**c**, **d**) and 60° (**e**, **f**) between the epitaxial growth direction and the barrier pattern direction. Scale bars, 1 µm.

[Action in Manuscript]

i) We have added **Supplementary Fig. 3 (SI p. 4)** to examine the effect of alignment between the epitaxial growth direction and the barrier pattern.

(SI p. 4) Supplementary Fig. 3 | Relationship between MoS_2 epitaxial growth direction and HfO_2 barrier pattern direction. a,b, Schematic (a) and SEM images (b) of MoS_2 growth when the epitaxial growth direction and the barrier pattern direction are parallel. c-f, Schematic and SEM images of MoS_2 growth at misalignment angles of 15° (c, d) and 60° (e, f) between the epitaxial growth direction and the barrier pattern direction. Scale bars, 1 μm.

ii) We have revised **manuscript (p. 5)** to clarify that the crystallographic orientation of MoS_2 is governed by the epitaxial growth direction rather than the barrier pattern geometry.

(p. 5) "Furthermore, when the barrier pattern direction is misaligned with the epitaxial growth direction, rough edges aligned with the intrinsic crystallographic orientation are observed near the barrier, instead of the clean triangular flake typical of EF-SCN (Supplementary Fig. 3). These results indicate that the orientation of triangular single-crystal MoS_2 in EF-SCN is determined by the epitaxial growth direction rather than the barrier pattern geometry. ~"

5. References

1 Mitchell, J. A. *et al.* Parallel simulation via SPPARKS of on-lattice kinetic and Metropolis Monte Carlo models for materials processing. *Modelling Simul. Mater. Sci. Eng.* **31**, 055001 (2023).

2 Kawasaki, K. Diffusion constants near the critical point for time-dependent Ising models. I. *Phys. Rev.* **145**, 224–230 (1966).

3 Chopard, B. & Droz, M. *Cellular Automata Modeling of Physical Systems* (Cambridge Univ. Press, 1998).

4 Nie, Y. *et al.* A kinetic Monte Carlo simulation method of van der Waals epitaxy for atomistic nucleation–growth processes of transition metal dichalcogenides. *Sci. Rep.* **7**, 2977 (2017).

5 Aldana, S. *et al.* Understanding substrate effects on MoS_2 growth: a kinetic Monte Carlo approach. *Adv. Mater. Interfaces* **11**, 2400209 (2024).

6 Lampoudi, S., Gillespie, D. T. & Petzold, L. R. The multinomial simulation algorithm for discrete stochastic simulation of reaction–diffusion systems. *J. Chem. Phys.* **130**, 094104 (2009).

7 Choi, T., Maurya, M. R., Tartakovsky, D. M. & Subramaniam, S. Stochastic operator-splitting method for reaction–diffusion systems. *J. Chem. Phys.* **137**, 184102 (2012).

8 Hellander, A., Lawson, M. J., Drawert, B. & Petzold, L. Local error estimates for adaptive simulation of the reaction–diffusion master equation via operator splitting. *J. Comput. Phys.* **266**, 89–100 (2014).

9 Hepburn, I. *et al.* Accurate reaction–diffusion operator splitting on tetrahedral meshes for parallel stochastic molecular simulations. *J. Chem. Phys.* **145**, 054118 (2016).

10 Åhlgren, E. H. *et al.* Atomic-scale oxygen-mediated etching of 2D MoS_2 and $MoTe_2$. *Adv. Mater. Interfaces* **9**, 2200987 (2022).

11 Farigliano, L. M., Paredes-Olivera, P. A. & Patrito, E. M. Initial steps of oxidative etching of MoS_2 basal plane induced by O_2. *J. Phys. Chem. C* **124**, 13177–13186 (2020).

12 Venables, J. A. Rate equation approaches to thin film nucleation kinetics. *Philos. Mag.* **27**, 697–738 (1973).

13 Kim, K. S. *et al.* Non-epitaxial single-crystal 2D material growth by geometric confinement. *Nature* **614**, 88–94 (2023).

14 Zhao, M. *et al.* Growth of atomically thin MoS_2 flakes on high-κ substrates by chemical vapor deposition. *J. Mater. Sci.* **53**, 4262–4273 (2018).

15 Kim, K. S. *et al.* Growth-based monolithic 3D integration of single-crystal 2D semiconductors. *Nature* **636**, 615–621 (2024).

16 Zhang, X. *et al.* Low-defect density monolayer MoS_2 wafer by oxygen-assisted growth-repair strategy. *Adv. Sci.* **11**, 2408640 (2024).

17 Chen, W. *et al.* Oxygen-assisted chemical vapor deposition growth of large single-crystal and high-quality monolayer MoS_2. *J. Am. Chem. Soc.* **137**, 15632–15635 (2015).

18 Shen, P.-C. *et al.* Healing of donor defect states in monolayer molybdenum disulfide using oxygen-incorporated chemical vapour deposition. *Nat. Electron.* **5**, 28–36 (2022).

19 Kim, T. S. *et al.* Gas-phase alkali metal-assisted MOCVD growth of 2D transition metal dichalcogenides for large-scale precise nucleation control. *Small* **18**, 2106368 (2022).

20 Zhang, X.-Y. *et al.* Temperature-dependent HfO_2/Si interface structural evolution and its mechanism. *Nanoscale Res. Lett.* **14**, 83 (2019).

21 Vinod, A., Rathore, M. S., Rao, N. S. Effects of annealing on quality and stoichiometry of HfO_2 thin films grown by RF magnetron sputtering. *Vacuum* **155**, 339–344 (2018).

22 Hsain, H. A. *et al.* Compositional dependence of crystallization temperatures and phase evolution in hafnia–zirconia $(Hf_xZr_{1-x})O_2$ thin films. *Appl. Phys. Lett.* **116**, 192901 (2020).

23 Murdzek, J. A., George, S. M. Effect of crystallinity on thermal atomic layer etching of hafnium oxide, zirconium oxide, and hafnium zirconium oxide. *J. Vac. Sci. Technol. A* **38**, 022608 (2020).

24 Ushakov, S. V. *et al.* Crystallization in hafnia- and zirconia-based systems. *Phys. Status Solidi B* **241**, 2268–2278 (2004).

25 Ushakov, S. V., Brown, C. E., Navrotsky, A. Effect of La and Y on crystallization temperatures of hafnia and zirconia. *J. Mater. Res.* **19**, 693–700 (2004).

26 Kim, H., McIntyre, P. C., Saraswat, K. C. Microstructural evolution of ZrO_2–HfO_2 nanolaminate structures grown by atomic layer deposition. *J. Mater. Res.* **19**, 643–650 (2004).

27 Mudavakkat, V. H. *et al.* Structure, morphology and optical properties of nanocrystalline yttrium oxide (Y_2O_3) thin films. *Opt. Mater.* **34**, 893–900 (2012).

28 Cho, M.-H. *et al.* Structural transition of crystalline Y_2O_3 film on Si(111) with substrate temperature. *Thin Solid Films* **349**, 266–269 (1999).

29 Han, G. H. *et al.* Seeded growth of highly crystalline molybdenum disulphide monolayers at controlled locations. *Nat. Commun.* **6**, 6128 (2015).

30 Li, Y. *et al.* Site-specific positioning and patterning of MoS_2 monolayers: The role of Au seeding. *ACS Nano* **12**, 8970–8976 (2018).

31 Ryu, B. *et al.* A facile approach for site-selective and large-area growth of MoS_2 through heterogeneous nucleation. *Appl. Surf. Sci.* **607**, 155066 (2023).

32 Li, S. *et al.* Wafer-scale and deterministic patterned growth of monolayer MoS_2 via vapor–liquid–solid method. *Nanoscale* **11**, 16122–16129 (2019).

33 Chu, C.-L *et al.* Stacked SiGe nanosheets p-FET for sub-3 nm logic applications. *Sci. Rep.* **13**, 9433 (2023).

34 Zhang, Q. *et al.* Optimization of structure and electrical characteristics for four-layer vertically-stacked horizontal gate-all-around Si nanosheets devices. *Nanomaterials* **11**, 646 (2021).

35 Loo, R. *et al.* Epitaxial Si/SiGe multi-stacks: From stacked nano-sheet to fork-sheet and CFET devices. *ECS J. Solid State Sci. Technol.* **14**, 015003 (2025).

36 Chung, Y.-Y. *et al.* Monolayer-MoS_2 stacked nanosheet channel with C-type metal contact. *IEDM Tech. Dig.* (2023).

37 Kim, C. *et al.* Transfer-free 2D CMOS multi-bridge channel FET. *IEDM Tech. Dig.* (2024).

38 Park, J. *et al.* Area-selective atomic layer deposition on 2D monolayer lateral superlattices. *Nat. Commun.* **15**, 2138 (2024).

39 Lee, K. *et al.* STEM image analysis based on deep learning: Identification of vacancy defects and polymorphs of MoS_2. *Nano Lett.* **22**, 4677–4685 (2022).

40 van der Zande, A. M. *et al.* Grains and grain boundaries in highly crystalline monolayer molybdenum disulphide. *Nat. Mater.* **12**, 554–561 (2013).

41 Sumaiya, S. A., Liu, J., Baykara, M. Z. True atomic-resolution surface imaging and manipulation under ambient conditions via conductive atomic force microscopy. *ACS Nano* **16**, 20086–20093 (2022).

Response Letter

Table of Contents

1. Overall Response to the Editor and Reviewers …………...……………………………………………… 2

2. Point-by-Point Responses to Reviewer #1 Comments …………...……………………………………… 3

3. Point-by-Point Responses to Reviewer #2, #3 Comments ……………………………………………….. 6

4. Minor Corrections in the Manuscript ……………………………………………………………….. 15

5. References ………………………………………………………………………………………...... 16

1. Overall Response to the Editor and Reviewers

We sincerely thank the Editor and the Reviewers for their careful and constructive evaluation of our revised manuscript, "Etching-flux mediated single-centered nucleation for spatially programmed growth of 2D semiconductors". We are especially grateful for the Editor's positive assessment that the manuscript may in principle be considered for publication, provided that the remaining concerns are fully addressed, particularly the need for an additional large-scale array demonstration and further technical clarification.

In response, we have undertaken a focused and comprehensive revision of the manuscript. We performed new experiments to evaluate the nucleation-suppression capability of the barrier, large-scale device-to-device uniformity, low-temperature scalability, and hetero-interface quality of the EF-SCN system. Furthermore, we reorganized the manuscript and Extended Data Figures to present the more important contents. We believe that these advances directly address the points raised by the Editor and Reviewers and further strengthen the experimental validation and mechanistic foundation of the manuscript. The principal additional demonstrations are summarized below, followed by detailed point-by-point responses in the subsequent sections.

Representative responses for large-scale array demonstrations

I. Large-area device uniformity

To demonstrate the large-area device-to-device uniformity of EF-SCN device, we fabricated and electrically characterized 50 back-gated MoS_2 FETs across a 2 cm substrate. The array exhibited an on-current relative standard deviation (RSD) of 14.66%, and a field-effect mobility RSD of 10.45%. All of these results exhibited variations below 15%, comparable to the best reported values for single-crystal 2D TMD devices. This demonstration verifies that EF-SCN is a highly reproducible single-crystal growth strategy capable of achieving large-area device-to-device uniformity.

II. Large-area low-temperature growth

To address the concern regarding the practical significance of the low-temperature process, we optimized EF-SCN process with ZrO_2 barrier. Well-defined single-centered nucleation was achieved at 430 °C, while 10 μm single-crystal MoS_2 domains were demonstrated by low-temperature EF-SCN. Consistent selective growth and single-crystalline MoS_2 formation were further confirmed at representative locations across a 2 cm substrate. These results show that the EF-SCN behavior—suppression of edge nucleation and localization of nucleation near the pattern center—can be retained at substantially reduced growth temperature.

In addition to these two main demonstrations, we systematically examined the thickness- and time-dependent durability of the etching effect by HfO_2 barrier, measured HAADF-STEM analysis of the MoS_2-$MoSe_2$ lateral heterointerface, and clarified the correspondence between the experimental parameters and the hybrid diffusion-reaction emulation.

2. Point-by-Point Responses to Reviewer #1 Comments

(1) The revised manuscript now includes many important additional datasets. Given the significance of some of the newly added results, the authors may consider reorganizing the figure structure. Some of the newly added data appear more central to the main message than the current Extended Data Fig. 1. Therefore, the authors may consider moving relatively less critical panels from Extended Data Fig. 1 to the Supplementary Information and placing some of the more important newly added datasets in the Extended Data figures.

[Author reply]

We sincerely thank the reviewer for the careful and thoughtful comments regarding the figure organization. As the reviewer pointed out, the SEM images and PL spectra presented in Extended Data Fig. 1 of the previous manuscript have relatively lower importance. In contrast, datasets such as Supplementary Fig. 5, which demonstrate the valid operating conditions of the EF-SCN system, are more essential for effectively conveying the main message of this work.

Accordingly, we have comprehensively reorganized the Extended Data Figures and Supplementary Figures by incorporating the reviewer's suggestions, along with the newly added data included in this 2nd revision process.

[Action in Manuscript]

i) We have reorganized Extended Data Figs. 1, 3, 4, and 9 according to the significance of the data and transferred less essential data to the Supplementary Figures.

(p. 16) Extended Data Fig. 1 | Characterizations of single-crystal MoS_2 flake by EF-SCN. a, AFM image of MoS_2 single-centered nucleation with the corresponding SEM inset image and height profile. **b**, Photoluminescence (PL) mapping image of a MoS_2 EF-SCN array. **c**, TEM image of a single-crystal MoS_2 flake fully suspended over a TEM grid hole. **d**, Diffraction pattern obtained from the flake in **c**. **e**, HAADF-STEM images acquired at S1, S2, and S3 positions marked in **c**, showing consistent atomic lattice orientation across the flake. **f**, Representative atomic-resolution AFM image of single-crystal MoS_2 flake by EF-SCN. **g**, AFM images and corresponding FFT patterns of single-crystal MoS_2.

(p. 17) Extended Data Fig. 3 | Valid conditions for HfO_2 to act as an etching-flux barrier. a–**c**, SEM images of MoS_2 grown on HfO_2 under low-H_2 and low-chalcogen conditions (**a**), under high-H_2 or high-chalcogen conditions (**b**), and on annealed HfO_2 under low-H_2 and low-chalcogen conditions (**c**). **d**, SEM images of MoS_2 nucleation as a function of HfO_2 thickness and in-situ pre-annealing time. Scale bars, 500 nm. **e**, Corresponding graph of MoS_2 coverage extracted from the SEM images in **d**. **f**, Schematic of MoS_2 growth on an HfO_2 surface with different conditions.

(p. 17) Extended Data Fig. 4 | Low-temperature EF-SCN using a ZrO_2 barrier. a,**b**, SEM images of single-crystal MoS_2 grown at 430 °C by low-temperature MOCVD. The ZrO_2 barrier suppresses MoS_2 nucleation at the pattern edge by the etching-flux, resulting in well-defined single-centered nucleation within the amorphous SiO_2 growth region. **c**,**d**, SEM images demonstrating effective EF-SCN at 500 °C in 10 μm patterns, showing both large single-crystal MoS_2 flake (**c**) and a continuous film (**d**) growth. **e**, Wafer-scale demonstration of low-temperature EF-SCN on a 2 cm substrate. The photograph, together with OM and SEM images collected from eight representative locations, confirms uniform single-crystal growth across the substrate. Black scale bars, 500 μm; white scale bars, 50 μm.

(p. 18) Extended Data Fig. 9 | Electrical characteristics and device-to-device uniformity of single-crystal MoS_2 BG-FET arrays by EF-SCN. a, Photograph of the chip containing the MoS_2 BG-FET arrays. **b**,**c**, OM images of the device arrays shown in **a** at higher magnifications. **d**, Transfer curves of 50 MoS_2 BG-FETs measured at V_{ds} = 1 V. **e**,**f**, Distribution of the on-current density (**e**) and relative deviations of the field-effect mobility (**f**) for 50 devices.

(2) In Figure 3, kMC is introduced for the first time. The authors may consider briefly defining the acronym as kinetic Monte Carlo at its first appearance. In addition, although the detailed description appears to be provided in the Supplementary Information, a short summary in the main text explaining how the kMC results support the experimental nucleation-control data would help readers better connect the simulation with the experimental observations.

[Author reply]

We appreciate the reviewer's helpful suggestion. As recommended, we now define the acronym at its first appearance in the main text. In addition, we have revised the main text so that each experimental control parameter is explicitly matched to its emulation parameter: L_{growth} to the template size, the MHC flow to the lateral growth probability, and the DES flow to the precursor adsorption probability, which sets the nucleation density.

[Action in Manuscript]

i) We have revised the manuscript to define the kMC acronym at its first appearance.

(p. 7) "To further examine whether spatially separated precursor supply and etching-flux can generate EF-SCN behavior, we performed kinetic Monte Carlo (kMC)-based hybrid diffusion-reaction emulation."

ii) We have revised the manuscript to match each experimental control parameter with its emulation parameter.

(p. 7) "In Extended Data Fig. 7, precursor species are supplied from above and HfO_2-derived oxygen etchants from the sidewalls, with adsorption, nucleation, lateral growth, and etching implemented as minimal reaction rules through a cellular-automaton-based scheme[35-37]. Using template size, lateral-growth probability, and adsorption probability as counterparts to L_{growth}, MHC flow, and DES flow, the emulation reproduced the zero-, single-, and multiple-nucleation regimes."

(3) The authors may also consider adding the simulation results as a GIF or other dynamic visualization in the Supplementary Information. This could help readers more intuitively understand the temporal evolution of the simulated nucleation process and further improve the clarity of the mechanistic explanation.

[Author reply]

We are grateful to the reviewer for this constructive suggestion. As recommended, we have added Supplementary Videos, which presents the temporal evolution of the emulated nucleation process for the zero-, single-, and multiple-nucleation regimes side by side.

In the video, each frame advances one diffusion-reaction cycle (cycles 0–500), the three panels correspond to relative template scales of 0.5×, 1.0×, and 1.5× shown on a common physical scale, and a synchronized cycle bar indicates the current cycle. The video can intuitively visualize how a single nucleus emerges near the template center and expands laterally under the single-nucleation condition, whereas no stable nucleus survives in the zero-nucleation regime and several nuclei form and coalesce in the multiple-nucleation regime.

[Action in Manuscript]

i) We have added Supplementary Videos 1 and 2 to more effectively illustrate the temporal evolution of nucleation in EF-SCN. The corresponding descriptions have been added to the manuscript and Supplementary Note 7.

(p. 7) "Under the single-nucleation condition, Fig. 3f shows center nucleation followed by lateral growth, consistent with a center-localized NAR; the temporal evolution is shown in Supplementary Videos 1 and 2."

(SI p. 22) Supplementary Video 1 | Time-evolution of the size-dependent nucleation regimes in the kMC-based hybrid diffusion-reaction process emulation. a–**c**, Representative emulation results for the zero (**a**), single (**b**), and multiple-nucleation (**c**) regimes at relative template scales of 0.5×, 1.0×, and 1.5× respectively. Each frame advances one diffusion-reaction cycle (cycles 0–500); all panels share a common physical scale and a synchronized cycle clock.

(SI p. 22) Supplementary Video 2 | Time-evolution of centered nucleation and shape-programmed growth in the kMC-based hybrid diffusion-reaction process emulation. a,**b**, Centered nucleation and subsequent lateral growth in triangular (**a**) and square (**b**) growth regions. The two regions were size-matched according to their inscribed-circle dimensions. **c**, Line-shaped growth through nucleation-growth decoupling in a template comprising a large nucleation zone and a narrow only growth zone. The square nucleation zone has the same lateral dimensions as the square growth region in **b**. A nucleus formed near the center of the nucleation zone expands into the only growth zone, where nucleation is suppressed but lateral growth is allowed.

(SI p. 38) "III. Time-evolution emulation of nucleation and growth in EF-SCN system. Supplementary Video 1 presents the time evolution of the size-dependent zero-, single-, and multiple-nucleation regimes in the kMC-based hybrid diffusion-reaction process emulation. The three panels show representative results at relative template scales of 0.5×, 1.0×, and 1.5×, respectively, using a common physical scale and synchronized diffusion-reaction cycles from 0 to 500. In the smallest template, the boundary-derived oxygen etching-flux suppresses stable nucleus formation throughout the growth region, resulting in zero nucleation. At the reference template scale, a stable nucleus forms near the center and subsequently expands laterally to fill the template, reproducing the single-centered nucleation sequence. In the largest template, the expanded center-localized NAR accommodates multiple stable nuclei, which grow simultaneously and ultimately produce the multiple-nucleation regime. This temporal evolution directly visualizes how template size regulates the spatial extent of the NAR and determines the zero-single-multiple nucleation transition. Supplementary Video 2 demonstrates centered nucleation and subsequent shape-programmed growth in growth regions with different geometries. Supplementary Video 2a, b show the temporal evolution of nucleation and lateral growth in triangular and square growth regions that are size-matched according to their inscribed-circle dimensions. In both geometries, nucleation initially occurs near the center, followed by lateral growth conforming to the template boundary, indicating that center-localized nucleation is not restricted to triangular patterns. Supplementary Video 2c further illustrates nucleation-growth decoupling in a line-shaped template consisting of a large nucleation zone connected to a narrow only-growth zone. A nucleus formed near the center of the nucleation zone expands into the only-growth zone, where additional nucleation is suppressed while lateral growth remains allowed. Consequently, a continuous line-shaped domain is formed from a single nucleation event. Together, these videos provide a time-resolved visualization of the EF-SCN design rule and demonstrate how spatial regulation of nucleation and lateral growth enables both deterministic nucleation control and programmable single-crystal geometries."

3. Point-by-Point Responses to Reviewer #2, #3 Comments

(1) The authors mention that the HfO_2 barrier eventually exhibits a "loss of oxygen-releasing ability". If the EF-SCN mechanism fundamentally relies on the lateral etching flux provided by HfO_2, the total available oxygen is finite and strictly governed by the volume/thickness of the barrier layer. A quantitative physical model or experimental data correlating the barrier thickness with its oxygen-releasing capacity and duration is required. It is important to understand the growth mechanism.

[Author reply]

This comment raises an important question regarding the oxygen-releasing capacity of the EF-SCN system. As the reviewer pointed out, the amount of oxygen released from the etching-flux barrier is inherently finite. Therefore, understanding the nucleation-suppression effect with barrier thickness and annealing time is essential for establishing the underlying mechanism. Following the reviewer's suggestion, we experimentally investigated the etching effect of the barrier in EF-SCN system.

Fig. R1 presents the MoS_2 growth behavior on the HfO_2 surface as a function of the in-situ pre-annealing time and HfO_2 thickness. The growth temperature and growth duration were fixed at 750 °C and 30 min, respectively. After ramping the temperature to 750 °C, an in-situ pre-annealing step was introduced prior to the precursor injection for MoS_2 growth, allowing the duration of the oxygen-mediated nucleation-suppression effect to be evaluated at different HfO_2 thicknesses.

As shown in the SEM images in Fig. R1a, no MoS_2 nucleation was observed on all HfO_2 surfaces with thicknesses ranging from 5 to 50 nm when the pre-annealing time was between 0 and 1 h. In contrast, MoS_2 growth was observed on the 5 nm HfO_2 surface after 3 h of pre-annealing, and on the 5–20 nm HfO_2 surfaces after 6 h of pre-annealing. These results indicate that the nucleation-suppression effect diminishes as pre-annealing time increases or HfO_2 thickness decreases, consistent with the progressive depletion of a finite oxygen reservoir within the HfO_2 barrier.

Fig. R1b shows the MoS_2 coverage extracted from the SEM images in Fig. R1a. Considering that the EF-SCN process is typically completed within 1 h, HfO_2 barriers with a thickness of 5 nm or greater are expected to retain sufficient nucleation-suppression capacity throughout the whole EF-SCN process for single-crystal growth.

As the reviewer correctly pointed out, the nucleation-etching effect of the barrier is indeed finite. Our results confirm that the oxygen reservoir is gradually depleted with decreasing HfO_2 thickness and increasing pre-annealing time, leading to undesired MoS_2 growth on the HfO_2 surface. Nevertheless, because the EF-SCN process is typically completed within 1 h, the finite nucleation-suppression capacity of the etching-flux barrier is unlikely to impose practical limitations under the process conditions employed in this work.

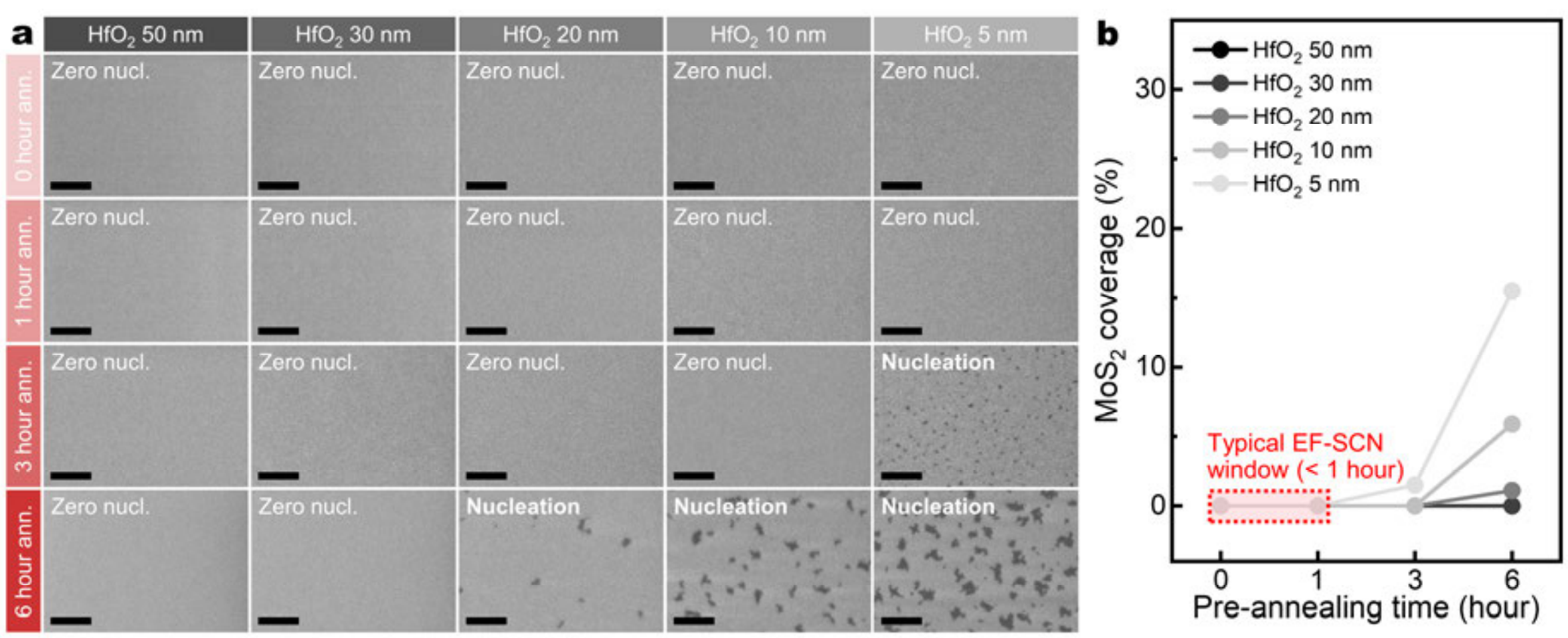

Fig. R1 | Effect of HfO_2 thickness and in-situ pre-annealing time on the nucleation-suppression ability of the HfO_2 barrier. a, SEM images of MoS_2 nucleation as a function of HfO_2 thickness and in-situ pre-annealing time. Scale bars, 500 nm. **b**, Corresponding MoS_2 coverage graph extracted from the SEM images in **a**. No MoS_2 nucleation is observed after growth following up to 1 hour of pre-annealing, demonstrating retention of the oxygen-mediated nucleation-suppression effect over the typical EF-SCN process window.

[Action in Manuscript]

i) We have revised Extended Data Fig. 3 and Supplementary Note 2 to demonstrate how the HfO_2 thickness and pre-annealing time affect its nucleation-suppression capability.

(p. 17) Extended Data Fig. 3 | Valid conditions for HfO_2 to act as an etching-flux barrier. a–c, SEM images of MoS_2 grown on HfO_2 under low-H_2 and low-chalcogen conditions (**a**), under high-H_2 or high-chalcogen conditions

(**b**), and on annealed HfO_2 under low-H_2 and low-chalcogen conditions (**c**). **d**, SEM images of MoS_2 nucleation as a function of HfO_2 thickness and in-situ pre-annealing time. Scale bars, 500 nm. **e**, Corresponding graph of MoS_2 coverage extracted from the SEM images in **d**. **f**, Schematic of MoS_2 growth on an HfO_2 surface with different conditions.

(SI p. 25) "Extended Data Fig. 3d, e present the MoS_2 growth behavior on the HfO_2 surface as a function of the in-situ pre-annealing time and HfO_2 thickness. The growth temperature and growth duration were fixed at 750 °C and 30 min, respectively. After ramping the temperature to 750 °C, an in-situ pre-annealing step was introduced prior to the precursor injection for MoS_2 growth, allowing the duration of the oxygen-mediated nucleation-suppression effect to be evaluated different HfO_2 thicknesses. As shown in the SEM images in Extended Data Fig. 3d, no MoS_2 nucleation was observed on all HfO_2 surfaces with thicknesses ranging from 5 to 50 nm when the pre-annealing time was between 0 and 1 h. In contrast, MoS_2 growth was observed on the 5 nm HfO_2 surface after 3 h of pre-annealing, and on the 5–20 nm HfO_2 surfaces after 6 h of pre-annealing. These results indicate that a longer pre-annealing time and a thinner HfO_2 thickness accelerate the depletion of oxygen in the HfO_2 barrier, thereby weakening its ability to suppress MoS_2 nucleation. Extended Data Fig. 3e shows the MoS_2 coverage extracted from the SEM images in Extended Data Fig. 3d. Considering that the EF-SCN process is typically completed within 1 h, HfO_2 barriers with a thickness of 5 nm or greater are expected to retain sufficient nucleation-suppression capacity throughout the whole EF-SCN process for single-crystal growth."

(2) To demonstrate the high reliability and low variation, the authors fabricated "multiple FETs" on a single MoS_2 stripe and claimed a low RSD of 7.8%. This statistical approach is highly misleading. Devices fabricated by partitioning the same single-crystal stripe naturally share identical qualities. This only proves that a single crystal is locally uniform. They need to demonstrate the reliability and uniformity of the EF-SCN growth method across a large-area array. Furthermore, the variation observed among the single FETs from different sites does not currently reflect a very high level of uniformity.

[Author reply]

We appreciate the reviewer's comment regarding the interpretation of the multiple-FETs configuration. The previously reported mobility RSD of 7.8%, obtained from multiple FETs partitioned within a single-crystal MoS_2, specifically evaluates device-to-device consistency within a common crystalline domain. This result is relevant to the intended device concept because it demonstrates that increasing the single-crystal dimension enables multiple devices with highly consistent electrical characteristics to be integrated within the same crystal.

However, we agree that evaluation across a large-area array is required to assess the uniformity of the EF-SCN process. To extend the statistical analysis from the within-crystal scale to the substrate scale, we therefore fabricated and characterized 50 independent MoS_2 FETs distributed across a 2 cm substrate. The revised manuscript now presents these two datasets as complementary assessments: the multiple-FETs configuration evaluates consistency within an individual single-crystal, whereas the 50-device array evaluates uniformity across a large-area substrate.

According to our survey of 11 reported studies, the device-to-device uniformity of polycrystalline/coalesced film-based devices exhibits a broad mobility RSD distribution, ranging from 8.8% to 29.7%[1-3], while the on-current RSD was reported to be approximately 9.1%[2]. For single-crystal epitaxial films, the mobility RSD was generally around 15%[4-6], with the best-performing studies achieving values as low as 8%[7-9]. The corresponding on-current RSD was reported to be in the range of 16–20%[4,8,9].

In the case of confined growth, which is most closely related to this work, the reported mobility RSD ranges from 11.4% to 24.9%, while the on-current RSD ranges from 12.9% to 25.2%[10,11]. Taken together, these results indicate that the mobility RSD of 21.8% reported in the previous manuscript falls within the typical range of single-crystal TMD device by selective growth. Nevertheless, since this RSD value was obtained from only 16 devices fabricated on a 1 cm substrate, we further performed a large-scale device demonstration to validate the uniformity of the EF-SCN growth process.

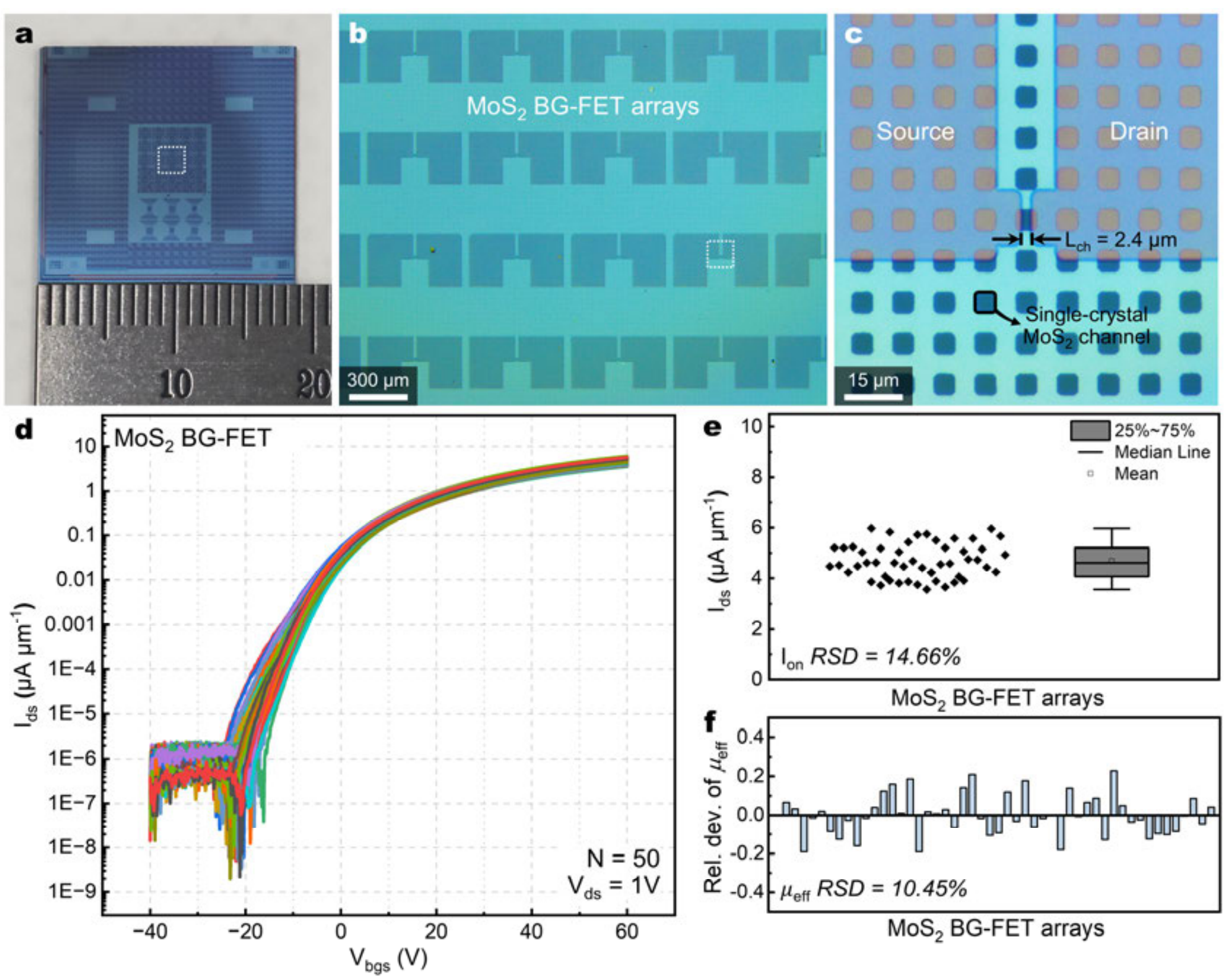

Fig. R2 | Electrical characteristics and device-to-device uniformity of single-crystal MoS_2 BG-FET arrays by EF-SCN. **a**, Photograph of the chip containing the MoS_2 BG-FET arrays. **b**,**c**, OM images of the device arrays shown in **a** at higher magnifications. **d**, Transfer curves of 50 MoS_2 BG-FETs measured at V_{ds} = 1 V. **e**,**f**, Distribution of the on-current density (**e**) and relative deviations of the field-effect mobility (**f**) for 50 devices.

Fig. R2a–c present a photograph and optical microscope (OM) images of back-gated field-effect transistor (BG-FET) arrays fabricated from single-crystal MoS_2 on a 2 cm SiO_2 substrate by EF-SCN. Fig. R2d shows the transfer curves of 50 devices from the MoS_2 BG-FET arrays, measured at V_{ds} = 1 V. Fig. R2e, f show the distribution of the on-current density and the relative deviation of the field-effect mobility, respectively. The 50 devices exhibited an on-current RSD of 14.66% (mean 4.69 μA μm^{-1}) and a field-effect mobility RSD of 10.45% (mean 8.56 $cm^2 V^{-1} s^{-1}$).

The RSD values obtained from the EF-SCN device arrays in Fig. R2d–f are comparable to those reported for confined growth systems (on-current RSD 12.9–25.2%; mobility RSD 11.4–24.9%)[10,11], demonstrating that the EF-SCN strategy achieves growth and device-to-device uniformity. Furthermore, considering that device performance variation is influenced not only by TMD's crystallinity but also by the subsequent device fabrication processes, achieving mobility and on-current RSDs below 15% across a 2 cm substrate strongly suggests that the EF-SCN system possesses a high degree of reproducibility and uniformity.

[Action in Manuscript]

i) We have added Extended Data Fig. 9 to demonstrate the large-area uniformity of the EF-SCN device array and described the related results in the manuscript, methods, and Supplementary Note 8.

(p. 8) "Additionally, to evaluate the large-area uniformity of the EF-SCN system, we analyzed the performance of 50 back-gated FETs (BG-FETs) fabricated using single-crystal MoS_2 channels grown across a 2 cm substrate. As shown in Extended Data Fig. 9, the devices exhibited RSDs of 10.45% in mobility and 14.66% in on-current density, supporting the superior uniformity of the EF-SCN system over a large area. Further details are provided in Supplementary Note 8."

(p. 15) "**Methods – Device fabrication and electrical characterization.** A back-gated FET array was fabricated using photolithography. Source and drain electrodes were formed by thermal evaporation of a 40 nm Au layer. For encapsulation, a 1 nm Si seed layer was first deposited by electron beam evaporation, and a 10 nm HfO_2 layer was subsequently deposited by PEALD. ~. Electrical measurements of the back-gated FET array were performed at room temperature under vacuum using a semiconductor analyzer (B1500A, Keysight), while those of the other devices were performed at room temperature under ambient conditions using a semiconductor analyzer (4200A-SCS, Keithley).

(p. 18) Extended Data Fig. 9 | Electrical characteristics and device-to-device uniformity of single-crystal MoS_2 BG-FET arrays by EF-SCN. a, Photograph of the chip containing the MoS_2 BG-FET arrays. **b**,**c**, OM images of the device arrays shown in **a** at higher magnifications. **d**, Transfer curves of 50 MoS_2 BG-FETs measured at V_{ds} = 1 V. **e**,**f**, Distribution of the on-current density (**e**) and relative deviations of the field-effect mobility (**f**) for 50 devices.

(SI p. 39) "**Supplementary Note 8. Large-scale device uniformity of EF-SCN method.** I. Device performance uniformity in previously reported TMD growth methods**.** To establish a basis for evaluating the uniformity of single-crystal MoS_2 grown by EF-SCN, we benchmarked previously reported studies. Based on a survey of 11 publications, the device-to-device uniformity of polycrystalline/coalesced film-based devices showed a broad mobility RSD distribution ranging from 8.8% to 29.7%[45-47], with an on-current RSD of 9.1%[46]. For single-crystal epitaxial films, mobility RSD was generally around 15%[48-50], with the best-performing cases reported as low as 8%[51-53]. On-current RSD for these films was reported in the range of 16–20%[48,52,53]. For confined-growth approaches, which are most closely related to this work, reported mobility RSD ranged from 11.4% to 24.9%, and on-current RSD ranged from 12.9% to 25.2%[20,21]. The mobility RSD of 21.8% reported in the main text (Fig. 4), obtained from 16 devices on a 1 cm substrate, also falls within this typical range for confined-growth systems. However, we further conducted the following experiment to verify the uniformity of the EF-SCN growth method at a larger scale. II. Uniformity of MoS_2 grown by EF-SCN on a 2 cm substrate. Extended Data Fig. 9a–c present a photograph and optical microscope (OM) images of back-gated field-effect transistor (BG-FET) arrays fabricated from single-crystal MoS_2 on a 2 cm SiO_2 substrate by EF-SCN. Each device had a channel length of 2.4 μm and a width of 5 μm. Source and drain electrodes were formed by depositing 40 nm of Au, followed by sequential deposition of a 1 nm Si seed layer and 10 nm HfO_2 for encapsulation. Extended Data Fig. 9d shows the transfer characteristics of the 50 devices measured at V_{ds} = 1 V. These devices exhibited an on-current RSD of 14.66% (mean 4.69 μA μm^{-1}) and a mobility RSD of 10.45% (mean 8.56 $cm^2 V^{-1} s^{-1}$) (Extended Data Fig. 9e, f). Both RSD values are comparable to those reported for confined-growth-based studies discussed above (on-current RSD 12.9–25.2%; mobility RSD 11.4–24.9%)[20,21], demonstrating that the EF-SCN growth method achieves excellent uniformity even at larger scales."

(3) The authors introduced a ZrO_2 barrier to achieve single-centered nucleation at low temperature. However, the intrinsic crystal quality appears to degrade significantly. Consequently, these results fail to convincingly demonstrate the superiority of this oxygen-mediated process over the previously established low-temperature "edge nucleation" methods.

[Author reply]

We appreciate the reviewer's concern regarding the crystal quality of MoS_2 obtained under the low-temperature conditions presented in the previous revision. As crystal quality encompasses several aspects, we used crystal morphology, a representative indirect indicator of it, as the criterion for improvement. To this end, we optimized the low-temperature MOCVD conditions and obtained MoS_2 crystals with well-defined triangular morphology and increased lateral dimensions, while preserving single-centered nucleation. Fig. R3 presents these expanded low-temperature EF-SCN results obtained using a ZrO_2 barrier on an amorphous SiO_2 substrate.

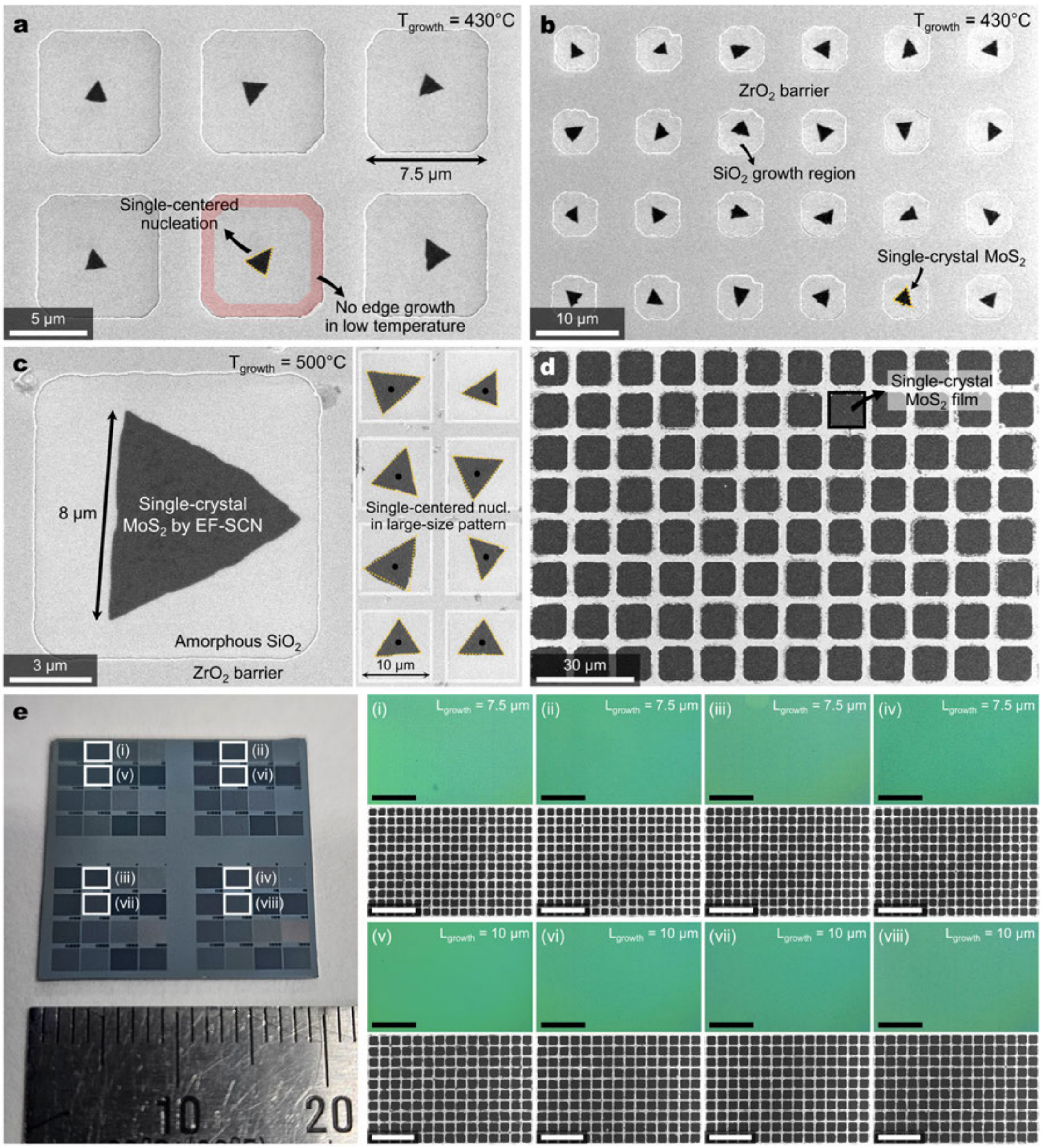

Fig. R3 | Low-temperature EF-SCN using a ZrO_2 barrier. a,**b**, SEM images of single-crystal MoS_2 grown at 430 °C by low-temperature MOCVD. The ZrO_2 barrier suppresses MoS_2 nucleation at the pattern edge by etching-flux, resulting in well-defined single-centered nucleation within the amorphous SiO_2 growth region. **c**,**d**, SEM images demonstrating effective EF-SCN at 500 °C in 10 µm patterns, showing both large single-crystal MoS_2 flake (**c**) and continuous film (**d**) growth. **e**, Wafer-scale demonstration of low-temperature EF-SCN on a 2 cm substrate. The photograph, together with OM and SEM images collected from eight representative locations, confirms uniform single-crystal growth across the substrate. Black scale bars, 500 µm; white scale bars, 50 µm.

Fig. R3a, b show SEM images of MoS_2 grown by EF-SCN at 430 °C. Well-defined triangular single-crystal MoS_2 flakes originating near the pattern center are observed even in relatively large patterns with lateral dimensions of 7.5 and 5 μm. Notably, although edge nucleation is generally favored under low-temperature growth conditions, the EF-SCN system effectively suppresses nucleation near the pattern boundary and preserves single-centered nucleation.

Fig. R3c, d present large single-crystal growth achieved by EF-SCN at 500 °C. Even within a large 10 μm pattern, centered nucleation of a large triangular single-crystal MoS_2 flake with a size of 8 μm is clearly observed (Fig. R3c). With increasing the growth time, the 10 μm region becomes fully filled with a single-crystalline MoS_2 film (Fig. R3d).

In previously reported low-temperature edge growth method, increasing the pattern size also increases the total edge length and the number of potential edge-nucleation sites. This generally raises the probability of multiple nucleation events and can limit the size of an individual single-crystal. In contrast, EF-SCN suppresses nucleation near the pattern boundary and localizes the surviving nucleation event toward the pattern center. Therefore, the present results demonstrate center-localized single-crystal growth in patterns as large as 10 μm under low-temperature conditions.

Fig. R3e demonstrates the large-area uniformity of low-temperature EF-SCN. The photograph shows the result of MoS_2 MOCVD performed on a 2 cm SiO_2 substrate patterned with ZrO_2 barrier. OM and SEM measurements were conducted at 8 locations across the substrate, and uniform single-crystalline MoS_2 film and selective growth behavior were consistently observed at all locations.

Together, these results more clearly establish the capability and scalability of low-temperature EF-SCN. Under the optimized conditions, we achieved well-defined center-localized MoS_2 growth at 430 °C, large single-crystal formation within patterns as large as 10 μm at 500 °C, and consistent selective-growth behavior across representative locations on a 2 cm substrate. The improved crystal morphology and increased lateral dimensions directly address the aspect of growth quality underlying the reviewer's concern. More importantly, these results demonstrate that the defining behavior of EF-SCN, suppression of edge nucleation and localization of nucleation near the pattern center, is retained at reduced growth temperatures and extended to larger growth patterns.

[Action in Manuscript]

i) We have added Extended Data Fig. 4 to demonstrate advanced low-temperature EF-SCN and revised the manuscript and methods accordingly.

(p. 6) "Extended Data Fig. 4a, b show SEM images of MoS_2 grown by EF-SCN at 430 °C on a SiO_2 substrate patterned with a ZrO_2 barrier. Despite the low-temperature process, uniform centered-nucleation of well-defined triangular single-crystalline MoS_2 domains is observed at the array level. Extended Data Fig. 4c, d demonstrate the growth of large single-crystal at 500 °C, including an 8 μm single-crystal MoS_2 flake and single-crystalline MoS_2 films fully filling the 10 μm regions. Furthermore, Extended Data Fig. 4e demonstrates the large-area uniformity of low-temperature EF-SCN. The photograph and the corresponding OM and SEM images show uniform selective growth and single-crystalline MoS_2 films across a 2 cm substrate. These results indicate that EF-SCN provides compatibility with low-temperature semiconductor processing and scalability toward large-area integration. Additional discussions on oxygen depletion from the etching-flux barrier and its impact are provided in Supplementary Note 4, along with Supplementary Fig. 9."

(p. 13) "**Methods – Etching-flux barrier pattern fabrication.** For the large-scale demonstration, photolithography was used to fabricate the barrier patterns. AZ 5214E photoresist (Merck Performance Materials) was spin-coated at 4,000 rpm and baked at 115 °C for 60 s. The photoresist was then exposed for 10–14 s using a mask aligner (Midas MDA-600S) and developed in AZ 300 MIF solution (Merck Performance Materials) for 45 s. Subsequently, the barrier material was deposited using an electron-beam evaporator, followed by lift-off in dimethyl sulfoxide (DMSO, Sigma-Aldrich)."

(p. 17) Extended Data Fig. 4 | Low-temperature EF-SCN using a ZrO_2 barrier. a,**b**, SEM images of single-crystal MoS_2 grown at 430 °C by low-temperature MOCVD. The ZrO_2 barrier suppresses MoS_2 nucleation at the pattern edge by the etching-flux, resulting in well-defined single-centered nucleation within the amorphous SiO_2 growth region. **c**,**d**, SEM images demonstrating effective EF-SCN at 500 °C in 10 μm patterns, showing both large single-crystal MoS_2 flake (**c**) and a continuous film (**d**) growth. **e**, Wafer-scale demonstration of low-temperature EF-SCN on a 2 cm substrate. The photograph, together with OM and SEM images collected from eight representative locations, confirms uniform single-crystal growth across the substrate. Black scale bars, 500 μm; white scale bars, 50 μm.

(4) Based on the current characterizations, the proposed method cannot clearly demonstrate an atomically sharp interface for the lateral heterostructures. In fact, the dynamic growth environment combined with continuous oxygen etching raises concerns about potential interdiffusion or alloying at the junction, which may yield inferior interface sharpness.

[Author reply]

Because the EF-SCN approach relies on an etching-flux released from the barrier, it is important to determine whether this chemical flux compromises the junction quality of 2D lateral heterostructures. During the 1st revision stage, we had already demonstrated through HAADF-STEM and atomic-resolution AFM that the in-plane oxygen etching-flux over the growth region primarily affects whether nucleation occurs and does not significantly degrade the 2D TMD itself.

However, as the reviewer noted, lateral heterostructures may be more susceptible to interface roughening, interdiffusion, or alloying by etching gas. We therefore conducted additional HAADF-STEM analyses of the interfacial region in lateral heterostructures fabricated by EF-SCN.

As shown in the SEM image in Fig. R4a, we demonstrated the spatially aligned 2D MoS_2-$MoSe_2$ lateral heterostructure by EF-SCN strategy. After growing the 2D lateral superlattice with an HfO_2 etching-flux barrier and transferring the flake onto a TEM grid, we conducted HAADF-STEM characterization. Fig. R4b reveals a clean junction within the MoS_2-$MoSe_2$ lateral heterostructure. To evaluate the interfacial quality more closely, two interface regions were analyzed at higher magnification (Fig. R4c, d). Both regions exhibited an extremely narrow intermixing width of less than 2 nm.

These observations indicate that the previously raised concerns regarding interface roughening, interdiffusion, and alloying are not significant, and that the resulting interface quality is comparable to that of other reported 2D lateral heterostructures[12-15]. This further confirms that the etching-flux in the EF-SCN system primarily governs the spatial position of nucleation without substantially degrading the quality of 2D TMD material itself.

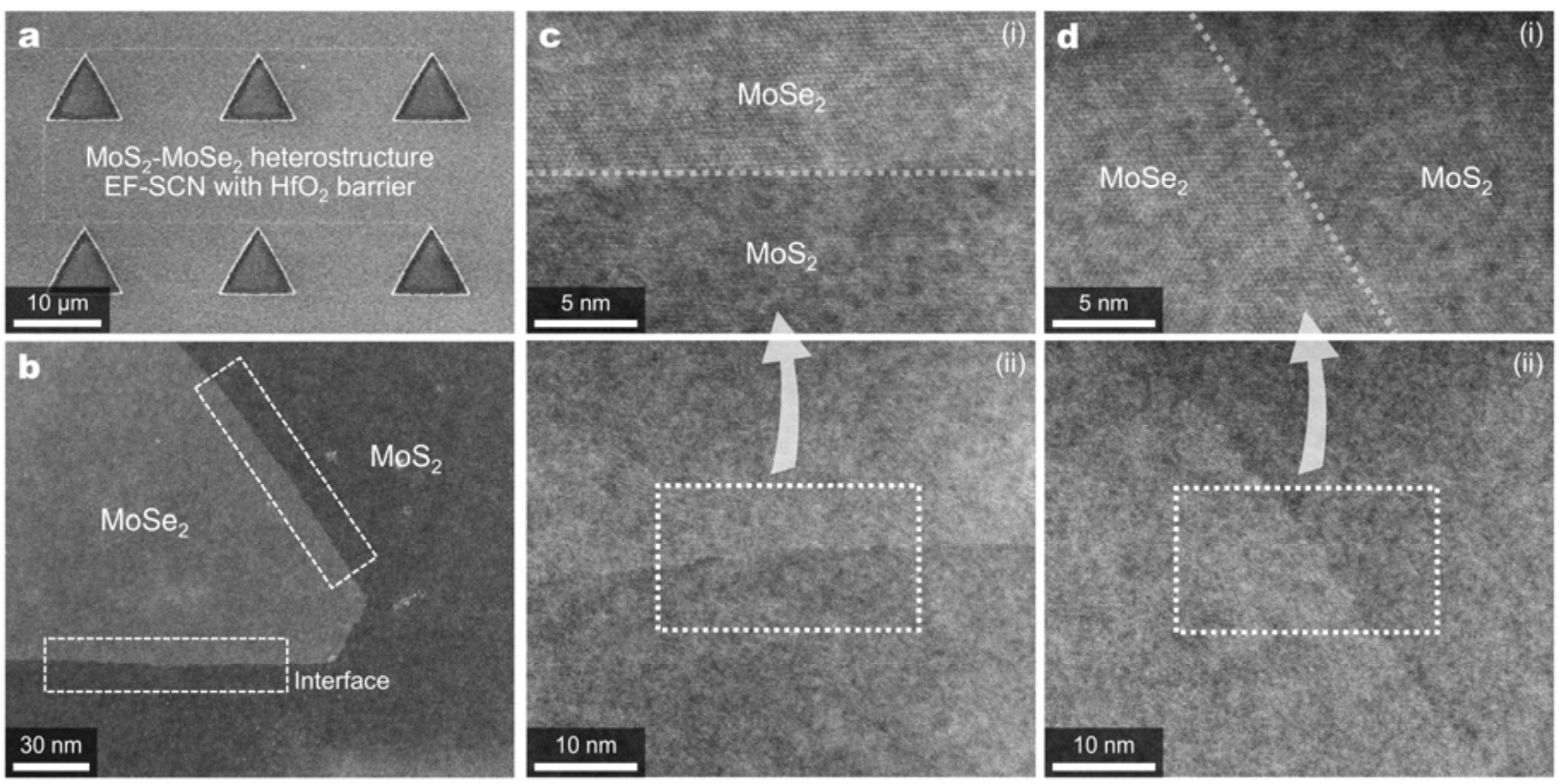

Fig. R4 | Atomic-scale characterization of the MoS_2-$MoSe_2$ lateral heterostructure interface. a, SEM image of the MoS_2-$MoSe_2$ lateral heterostructure grown by EF-SCN method, where the inner region is $MoSe_2$ and the outer region is MoS_2. **b–d**, HAADF-STEM images of the MoS_2-$MoSe_2$ lateral heterostructure interface. High-magnification images reveal an atomically sharp interface with an intermixing width of approximately 1–2 nm.

[Action in Manuscript]

i) We have revised Extended Data Fig. 10 to demonstrate the interfacial quality of the 2D lateral heterostructure by EF-SCN and added the related description to the manuscript.

(p. 8) "We successfully synthesized 2D MoS_2-$MoSe_2$ lateral superlattices with precise positional and orientational alignment at the array level. As shown in the HAADF-STEM image in Extended Data Fig. 10e, these lateral heterostructures exhibit sharp interfaces with an intermixing width of less than 2 nm."

(p. 18) Extended Data Fig. 10 | Alignment of 2D TMD lateral heterostructure for self-aligned ALD contact FET. a–d, Schematic (**a**) and SEM images (**b–d**) of aligned 2D MoS_2-$MoSe_2$ lateral heterostructure by the EF-SCN method. **e**, HAADF-STEM images of the MoS_2-$MoSe_2$ lateral heterostructure interface with an intermixing width of approximately 1–2 nm. **f**, Schematic of self-aligned ALD contact FET with Ru AS-ALD on 2D MoS_2-$MoSe_2$ lateral heterostructure.

(5) Looking at the broader implications of this work, the concepts of oxygen-assisted etching and geometrically confined selective growth of 2D materials have been previously established. While utilizing an amorphous oxide barrier as a localized, solid-state oxygen source is more like a "process optimization" rather than a fundamental breakthrough in underlying mechanisms. The authors must explicitly articulate the fundamental scientific novelty that elevates this work. What entirely new physical insight does this mechanism reveal that justifies its publication in Nature?

[Author reply]

As the reviewer correctly pointed out, we agree that EF-SCN may appear to be a process optimization that combines oxygen-assisted growth with confined growth. Indeed, EF-SCN is also a technique for growing single-crystal 2D TMD on the basis of selective growth, and we fully acknowledge that it employs oxygen-mediated nucleation control.

However, the central message of this work is not the simple combination or optimization of existing processes, but the introduction of a deterministic nucleation framework that redefines the fundamental principle of selective growth. The scientific novelty of EF-SCN lies not merely in the resulting growth of single-crystal, but in its implementation of a mechanistically distinct framework for establishing selectivity and physically controlling the spatial position of nucleation. To clarify this point, we summarize the scientific significance of EF-SCN from the following three perspectives.

(i) A distinct physical mechanism: Deterministic single-centered nucleation

The fundamental novelty of EF-SCN is that it spatially regulates the nucleation probability within the growth region through a chemical flux, thereby enabling deterministic single nucleation.

Most selective-growth approaches, including selective epitaxial growth (SEG), area-selective atomic layer deposition (AS-ALD), and confined growth, establish selectivity through differences in reaction rate or binding energy between growth and non-growth regions. However, once the depositing species enter the growth region, the precise nucleation position remains intrinsically stochastic.

This behavior is generally acceptable for amorphous deposition, where numerous uniformly distributed nucleation events are desirable. In crystalline systems, however, the entire growth region remains a potential nucleation domain, fundamentally constraining selective single-crystal growth. Therefore, a single-crystal can form only when lateral growth covers the designated region before additional nucleation occurs, imposing stringent limitations on pattern dimensions and process conditions.

Conventional barrier-based systems cannot eliminate this limitation because the entire growth region remains nucleation-active. Edge- or corner-directed approaches likewise do not guarantee single-point nucleation because a closed geometry generally contains multiple equivalent edges or corners. Seed-assisted and vapor-liquid-solid (VLS) approaches can define the growth position more explicitly, but seed materials such as Au, Pt, Mo, or MoO_x may remain after growth and affect the material or device properties.

In contrast, EF-SCN uses a lateral chemical flux released from the barrier to create a spatially non-uniform nucleation probability distribution without a seed material. Therefore, its novelty lies not merely in producing a single-crystal, but in introducing a new physical framework in which the position of selective nucleation is deterministically defined.

(ii) A selective growth mechanism unique to 2D materials: Van der Waals surface for spatial control

EF-SCN does not merely use oxygen-assisted growth to reduce the nucleation density. Rather, it exploits the distinctive crystallographic characteristics of 2D TMD to spatially control the nucleation position.

Conventional selective growth, including confined growth of 2D TMD, is primarily governed by binding energy differences between growth and non-growth surfaces. In contrast, EF-SCN represents a distinctive selective growth approach that uses a lateral chemical-flux field to control the nucleation position within the growth region. This capability originates from the van der Waals surface, which is a unique crystallographic property of 2D materials.

Since 2D nuclei are not strongly anchored to specific surface dangling bonds, their formation and survival remain highly sensitive to the local chemical environment. Consequently, in materials such as 2D TMD, the nucleation position can be more effectively controlled by the lateral etching-flux field. Importantly, EF-SCN does not merely regulate nucleation density using an etching gas; rather, it achieves deterministic nucleation by spatially controlling the position of nuclei on the micrometer scale. This means that EF-SCN constitutes a new selective growth strategy that fully exploits the distinctive crystallographic characteristics of 2D TMD.

(iii) EF-SCN process scalability: Single-crystal size scaling

Deterministic nucleation also alleviates the size limitations of conventional confined growth. In previous approaches, multiple nucleation events cannot be eliminated in principle, and large single-crystal can be obtained when lateral growth proceeds substantially faster than subsequent nucleation.

EF-SCN instead directs nucleation to the geometrical center, which is uniquely defined within the pattern. Therefore, the deterministic growth mechanism can be preserved as the growth region increases in size. Whereas confined growth studies have generally demonstrated effective single-crystal growth at approximately the 1 μm scale, EF-SCN realizes

single-crystal channels as large as 10 μm, even below 500 °C.

As the reviewer noted in comment #2, devices partitioned from the same crystal are naturally expected to exhibit similar intrinsic properties. Therefore, increasing the single-crystal dimension provides not only a larger crystal but also a greater area for integrating devices within a common crystalline domain. Increasing the linear dimension from 1 μm to 10 μm, for example, yields a hundredfold increase in the available integration area. Thus, EF-SCN provides a practical route toward larger-scale integration with reduced device-to-device variation.

We fully appreciate the reviewer's concern that EF-SCN could be viewed as a process optimization with limited fundamental novelty. Indeed, when evaluated solely by the final growth outcome, it may appear to be another selective growth method for producing single-crystal. However, the essential distinction lies in the physical origin of selectivity. EF-SCN establishes a chemical flux field within the growth region and converts it into spatially deterministic nucleation, a mechanism that is inaccessible to conventional binding energy-based selective growth. By addressing key challenges such as large-area device uniformity and low-temperature growth during this revision, EF-SCN demonstrates complementary spatial programmability at the device-pattern level, distinct from conventional epitaxial growth for single-crystalline film.

We anticipate that this concept may extend beyond 2D TMD and provide a general direction for spatially controlling single nucleation in selective growth systems where crystallinity is critical. Hopefully, we believe that EF-SCN represents not merely a process optimization, but a conceptual and scientific advance that meets the level of fundamental insight.

[Action in Manuscript]

i) We have revised the abstract and introduction to more logically and explicitly clarify that EF-SCN is not merely a process optimization, but establishes a new physical framework for selective growth with unique advantages.

(p. 2) "Nucleation control is fundamental to semiconductor deposition, governing when, where and how crystalline materials form. Conventional strategies can regulate nucleation density and, through area-selective growth, confine deposition to designated regions[1-13]. However, they generally do not determine where an individual nucleus forms within the growth regions, leaving even single-nucleation events spatially stochastic and limiting deterministic construction of crystalline materials. Here we report etching-flux mediated single-centered nucleation (EF-SCN) of two-dimensional (2D) semiconductors to deterministically localize a single nucleation event. The etching-flux released from the barrier suppresses nuclei near the pattern boundary, leaving a single surviving nucleus at the pattern center, while systematic experiments elucidate the mechanism and establish nucleation design rules for zero, single, and multiple nucleation regimes. EF-SCN enables single-crystal MoS_2 growth at the 10 μm scale, field-effect mobilities of up to 117 $cm^2 V^{-1} s^{-1}$, and large-area uniformity with process compatibility. This in-plane chemical-flux strategy realizes spatially programmed growth, demonstrating nucleation-growth decoupling for line-shaped single crystal, multiple transistors integrated within a large common crystal, and aligned 2D lateral heterostructures for self-aligned contacts. These capabilities open a path towards advanced 2D electronic integration and expand semiconductor deposition from controlling where materials grow to controlling where and how crystals can begin to form."

(p. 2–3) "To address these limitations, we developed an etching-flux mediated single-centered nucleation (EF-SCN) strategy that introduces a new paradigm for nucleation control in ASG systems. Unlike conventional 2D TMD ASG, which relies solely on out-of-plane selectivity through heterogeneous surface systems with different surface binding energies, EF-SCN achieves in-plane spatial control of nucleation by creating a non-uniform nucleation probability within the growth region. Oxygen released from an oxide barrier produces a lateral etching-flux that suppresses nucleation, defining the nucleation-active region at the geometric center of each pattern. This flux-driven mechanism enables deterministic nucleation and establishes a new physical framework in which in-plane chemical flux regulates spatial nucleation probability.

From a practical standpoint, EF-SCN also provides substantial advantages and novel device configurations. The deterministic nucleation at a center point enables effective nucleation suppression and single-crystal size scaling, producing single-crystal MoS_2 at the 10 μm scale. For process compatibility, EF-SCN achieves a single-crystal yield exceeding 99% and uniform selectivity over a large area, and can be applied to low-temperature growth at 430 °C. The EF-SCN-grown single-crystal MoS_2 exhibits a high field-effect mobility of 117 $cm^2 V^{-1} s^{-1}$, while a large single crystal realizes the integration of multiple field-effect transistors (FETs) within a single channel with a mobility relative standard deviation (RSD) of 7.8%. Moreover, precise positional and orientational alignment of 2D lateral heterostructures enables a distinctive demonstration of self-aligned ALD contact FETs. Collectively, EF-SCN establishes a new selective-growth framework that achieves deterministic nucleation and spatially programmed growth, providing a pathway toward functional single-crystal architectures and advanced 2D electronic integration."

4. Minor Corrections in the Manuscript

In this section, we briefly summarize minor corrections to the manuscript that are not addressed in the point-by-point responses.

i. Three authors have been added. **(p. 1)**

ii. The manuscript has been substantially condensed to comply with the Nature publication format, while preserving its overall organization and content.
- Abstract, from 280 to 200 words
- Introduction, from 630 to 480 words
- 1st section, from 1090 to 610 words
- 2nd section, from 1380 to 970 words
- 3rd section, from 1350 to 770 words
- 4th section, from 1130 to 1020 words
- Conclusion, from 330 to 210 words

iii. The contents removed from the main text during the above condensation have been incorporated into Supplementary Notes 1 and 5.
(SI p. 23) "**Supplementary Note 1. Rigorous verifications of single-crystallinity for EF-SCN MoS_2.** We performed various characterizations to confirm whether the MoS_2 flakes grown by EF-SCN method were indeed single-crystalline. As shown in Supplementary Fig. 2d, e, the characteristics of single and multiple nucleation can be readily distinguished by SEM images. To further confirm that the triangular MoS_2 flake observed in Fig. 1 is a single crystal, more rigorous characterizations were performed. Extended Data Fig. 1c–e present transmission electron microscopy (TEM) analyses of a MoS_2 flake grown by the EF-SCN method. The diffraction pattern obtained from a MoS_2 flake suspended over a TEM grid hole (Extended Data Fig. 1c) exhibits a typical hexagonal spot pattern of single-crystal MoS_2, rather than multiple spots or ring patterns (Extended Data Fig. 1d). ~. Extended Data Fig. 2 shows second-harmonic generation (SHG) analyses of single-crystal MoS_2 grown by EF-SCN. In the case of single nucleation, a single triangular flake is observed in the SEM image, and the absence of grain boundaries results in uniform SHG intensity mapping, along with a well-defined six-petal pattern in angle-resolved SHG, characteristic of single-crystal MoS_2 (Extended Data Fig. 2a–c). In contrast, under multiple nucleation conditions, multiple domains and irregular flake morphologies are observed in the SEM image, accompanied by a significant intensity drop at grain boundaries in SHG mapping. The coexistence of domains with different orientations results in an isotropic SHG intensity distribution rather than a six-petal pattern (Extended Data Fig. 2d–f)."
(SI p. 32) "**Supplementary Note 5. Additional discussions of L_{growth} and growth conditions.** Extended Data Fig. 6d displays the critical length (blue hatched bar) and the number of nuclei per pattern (red filled bar) as functions of L_{growth}. Even as L_{growth} increases, the critical length remains nearly constant, because the position where the net nucleation rate becomes zero does not change, as illustrated in Fig. 3a (i) and (iii). ~. Supplementary Fig. 11 shows the threshold chalcogen flow required for MoS_2 nucleation as a function of L_{growth}. The graph reveals that a smaller L_{growth} requires a higher chalcogen flow for nucleation to occur, indicating that the DES flow critically controls R_{Growth} control. Supplementary Fig. 12 shows the nucleation and growth behavior under low and high DES flow conditions (3 and 5 sccm), and low and high MHC conditions (2.5 and 4 sccm). Under high DES conditions, the number of flakes is consistently about twice that observed under low DES conditions, suggesting that DES flow is the limiting factor governing nucleation density and rate in the EF-SCN system."

iv. To improve clarity, we have divided the main text into four sections and added the following subheadings:
- Etching-flux mediated single-centered nucleation
- EF-SCN mechanism and process compatibility
- Nucleation design rule
- Spatially programmed single-crystal architectures

v. We have corrected minor errors in the legends of Figures

vi. We have separated and rewritten the Acknowledgements and Funding sections. **(p. 15–16)**

5. References

1 Dodda, A. et al. Active pixel sensor matrix based on monolayer MoS_2 phototransistor array. *Nat. Mater.* **21**, 1379–1387 (2022).

2 Seol, M. et al. High-throughput growth of wafer-scale monolayer transition metal dichalcogenide via vertical Ostwald ripening. *Adv. Mater.* **32**, 2003542 (2020).

3 Smithe, K. K. H. et al. Low variability in synthetic monolayer MoS_2 devices. *ACS Nano* **11**, 8456–8463 (2017).

4 Liu, L. et al. Uniform nucleation and epitaxy of bilayer molybdenum disulfide on sapphire. *Nature* **605**, 69–75 (2022).

5 Huang, L.-Y. et al. Wafer-scale single-crystal WSe_2 monolayers using substrate-passivation-driven epitaxy. *ACS Nano* **20**, 2104–2113 (2026).

6 Li, C. et al. Revisiting the epitaxial growth mechanism of 2D TMDC single crystals. *Adv. Mater.* **36**, 2404923 (2024).

7 Fu, W. et al. Evidence of air-induced surface transformation of atomic step-engineered sapphire in relation to epitaxial growth of 2D semiconductors. *Nat. Commun.* **16**, 8488 (2025).

8 Zou, X. et al. Robust epitaxy of single-crystal transition-metal dichalcogenides on lanthanum-passivated sapphire. *Science* **390**, eaea0849 (2025).

9 Li, T. et al. Epitaxial growth of wafer-scale molybdenum disulfide semiconductor single crystals on sapphire. *Nat. Nanotechnol.* **16**, 1201–1207 (2021).

10 Kim, K. S. et al. Non-epitaxial single-crystal 2D material growth by geometric confinement. *Nature* **614**, 88–94 (2023).

11 Kim, K. S. et al. Growth-based monolithic 3D integration of single-crystal 2D semiconductors. *Nature* **636**, 615–620 (2024).

12 Park, J. et al. Area-selective atomic layer deposition on 2D monolayer lateral superlattices. *Nat. Commun.* **15**, 2138 (2024).

13 Sahoo, P. K., Memaran, S., Xin, Y., Balicas, L. & Gutierrez, H. R. One-pot growth of two-dimensional lateral heterostructures via sequential edge-epitaxy. *Nature* **553**, 63-67 (2018).

14 Gong, Y. et al. Vertical and in-plane heterostructures from WS_2/MoS_2 monolayers. *Nat. Mater.* **13**, 1135-1142 (2014).

15 Huang, C. et al. Lateral heterojunctions within monolayer $MoSe_2$-WSe_2 semiconductors. *Nat. Mater.* **13**, 1096-1101 (2014).
